# Long Paths in First Passage Percolation on the Complete Graph II. Global Branching Dynamics

**DOI:** 10.1007/s10955-020-02585-1

**Published:** 2020-08-06

**Authors:** Maren Eckhoff, Jesse Goodman, Remco van der Hofstad, Francesca R. Nardi

**Affiliations:** 1 Department of Mathematical Sciences, University of Bath, Bath, BA2 7AY UK; 2 Department of Statistics, University of Auckland, Private Bag 92019, Auckland, 1142 New Zealand; 3 Department of Mathematics and Computer Science, Eindhoven University of Technology, P.O. Box 513, 5600 MB Eindhoven, The Netherlands

**Keywords:** First-passage percolation, Stochastic mean-field model of distance, Strong disorder, Continuous-time branching processes, Invasion percolation

## Abstract

We study the random geometry of first passage percolation on the complete graph equipped with independent and identically distributed positive edge weights. We consider the case where the lower extreme values of the edge weights are highly separated. This model exhibits strong disorder and a crossover between local and global scales. Local neighborhoods are related to invasion percolation that display self-organised criticality. Globally, the edges with relevant edge weights form a barely supercritical Erdős–Rényi random graph that can be described by branching processes. This near-critical behaviour gives rise to optimal paths that are considerably longer than logarithmic in the number of vertices, interpolating between random graph and minimal spanning tree path lengths. Crucial to our approach is the quantification of the extreme-value behavior of small edge weights in terms of a sequence of parameters $(s_n)_{n\ge 1}$ that characterises the different universality classes for first passage percolation on the complete graph. We investigate the case where $s_n\rightarrow \infty$ with $s_n=o(n^{1/3})$, which corresponds to the barely supercritical setting. We identify the scaling limit of the weight of the optimal path between two vertices, and we prove that the number of edges in this path obeys a central limit theorem with mean approximately $s_n\log {(n/s_n^3)}$ and variance $s_n^2\log {(n/s_n^3)}$. Remarkably, our proof also applies to *n*-dependent edge weights of the form $E^{s_n}$, where *E* is an exponential random variable with mean 1, thus settling a conjecture of Bhamidi et al. (Weak disorder asymptotics in the stochastic meanfield model of distance. Ann Appl Probab 22(1):29–69, 2012). The proof relies on a decomposition of the smallest-weight tree into an initial part following invasion percolation dynamics, and a main part following branching process dynamics. The initial part has been studied in Eckhoff et al. (Long paths in first passage percolation on the complete graph I. Local PWIT dynamics. Electron. J. Probab. 25:1–45, 2020. 10.1214/20-EJP484); the current paper focuses on the global branching dynamics.

## Model and Summary of Results

In this paper, we study first passage percolation on the complete graph equipped with independent and identically distributed positive and continuous edge weights. In contrast to earlier work [11, 12, 16, 20, 27], we consider the case where the extreme values of the edge weights are highly separated.

We start by introducing first passage percolation (FPP). Given a graph ${\mathcal {G}}=(V({\mathcal {G}}),E({\mathcal {G}}))$, let $(Y_e^{\scriptscriptstyle ({\mathcal {G}})})_{e\in E({\mathcal {G}})}$ denote a collection of positive edge weights. Thinking of $Y_e^{\scriptscriptstyle ({\mathcal {G}})}$ as the cost of crossing an edge *e*, we can define a metric on $V({\mathcal {G}})$ by setting

1.1 $$\begin{aligned} d_{{\mathcal {G}},Y^{({\mathcal {G}})}}(i,j)=\inf _{\pi :i\rightarrow j} \sum _{e\in \pi } Y_e^{\scriptscriptstyle ({\mathcal {G}})}, \end{aligned}$$

where the infimum is over all paths $\pi$ in ${\mathcal {G}}$ that join *i* to *j*, and $Y^{\scriptscriptstyle ({\mathcal {G}})}$ represents the edge weights $(Y_e^{\scriptscriptstyle ({\mathcal {G}})})_{e\in E({\mathcal {G}})}$. We will always assume that the infimum in (1.1) is attained uniquely, by some (finite) path $\pi _{i,j}$. We are interested in the situation where the edge weights $Y_e^{\scriptscriptstyle ({\mathcal {G}})}$ are *random*, so that $d_{{\mathcal {G}},Y^{({\mathcal {G}})}}$ is a random metric. In particular, when the graph ${\mathcal {G}}$ is very large, with $\left| V({\mathcal {G}})\right| =n$ say, we wish to understand the scaling behavior of the following quantities for fixed $i,j \in V({\mathcal {G}})$:

- The *distance* $W_n=d_{{\mathcal {G}},Y^{({\mathcal {G}})}}(i,j)$—the total edge cost of the optimal path $\pi _{i,j}$;
- The *hopcount* $H_n$—the number of edges in the optimal path $\pi _{i,j}$;
- The *topological structure*—the shape of the random neighborhood of a point.

In this paper, we consider FPP on the complete graph, which acts as a mean-field model for FPP on finite graphs. In [11], the question was raised what the *universality classes* are for this model. We bring the discussion substantially further by describing a way to distinguish several universality classes and by identifying the limiting behavior of first passage percolation in one of these classes. The cost regime introduced in (1.1) uses the information from all edges along the path and is known as the *weak disorder* regime. By contrast, in the *strong disorder* regime the cost of a path $\pi$ is given by $\max _{e \in \pi } Y_e^{\scriptscriptstyle ({\mathcal {G}})}$. We establish a firm connection between the weak and strong disorder regimes in first passage percolation. Interestingly, this connection also establishes a strong relation to invasion percolation (IP) on the Poisson-weighted infinite tree (PWIT), which is the local limit of IP on the complete graph, and also arises in the context of the minimal spanning tree on the complete graph (see e.g. [1]).

Our main interest is in the case ${\mathcal {G}}=K_n$, the complete graph on *n* vertices $V(K_n)=[n]:=\left\{ 1,\ldots ,n\right\}$, equipped with independent and identically distributed (i.i.d.) edge weights $(Y_e^{\scriptscriptstyle (K_n)})_{e \in E(K_n)}$. We write *Y* for a random variable with $Y\overset{d}{=}Y_e^{\scriptscriptstyle ({\mathcal {G}})}$, and assume that the distribution function $F_{\scriptscriptstyle Y}$ of *Y* is continuous. For definiteness, we study the optimal path $\pi _{1,2}$ between vertices 1 and 2. First, we introduce some general notation:

**Notation.** All limits in this paper are taken as *n* tends to infinity unless stated otherwise. A sequence of events $({\mathcal {A}}_n)_n$ happens *with high probability* (*whp*) if ${\mathbb {P}}({\mathcal {A}}_n) \rightarrow 1$. For random variables $(X_n)_n, X$, we write $X_n {\mathop {\longrightarrow }\limits ^{d}}X$, $X_n {\mathop {\longrightarrow }\limits ^{\scriptscriptstyle {{\mathbb {P}}}}}X$ and $X_n {\mathop {\longrightarrow }\limits ^{a.s.}}X$ to denote convergence in distribution, in probability and almost surely, respectively. For real-valued sequences $(a_n)_n$, $(b_n)_n$, we write $a_n=O(b_n)$ if the sequence $(a_n/b_n)_n$ is bounded; $a_n=o(b_n)$ if $a_n/b_n \rightarrow 0$; $a_n =\Theta (b_n)$ if the sequences $(a_n/b_n)_n$ and $(b_n/a_n)_n$ are both bounded; and $a_n \sim b_n$ if $a_n/b_n \rightarrow 1$. Similarly, for sequences $(X_n)_n$, $(Y_n)_n$ of random variables, we write $X_n=O_{\scriptscriptstyle {{\mathbb {P}}}}(Y_n)$ if the sequence $(X_n/Y_n)_n$ is tight; $X_n=o_{\scriptscriptstyle {{\mathbb {P}}}}(Y_n)$ if $X_n/ Y_n {\mathop {\longrightarrow }\limits ^{\scriptscriptstyle {{\mathbb {P}}}}}0$; and $X_n =\Theta _{{\mathbb {P}}}(Y_n)$ if the sequences $(X_n/Y_n)_n$ and $(Y_n/X_n)_n$ are both tight. We denote by $\lfloor x\rfloor$ the greatest integer not exceeding *x*. Moreover, *E* denotes an exponentially distributed random variable with mean 1. We often need to refer to results from [21], and we will write, e.g., [Part I, Lemma 2.18] for [21, Lemma 2.18].

For a brief overview of notation particular to this paper, see p. 81.

### First Passage Percolation with Regularly-Varying Edge Weights

In this paper, we will consider edge-weight distributions with a heavy tail near 0, in the sense that the distribution function $F_{\scriptscriptstyle Y}(y)$ decays slowly to 0 as $y\downarrow 0$. It will prove more convenient to express this notion in terms of inverse $F_{\scriptscriptstyle Y}^{-1}(u)$, since we can write

1.2 $$\begin{aligned} Y_e^{\scriptscriptstyle (K_n)} \overset{d}{=}F_{\scriptscriptstyle Y}^{-1}(U), \end{aligned}$$

where *U* is uniformly distributed on [0, 1]. Expressed in terms of $F_{\scriptscriptstyle Y}^{-1}$, saying that the edge-weight distribution is heavy-tailed near 0 means that $F_{\scriptscriptstyle Y}^{-1}(u)$ decays rapidly to 0 as $u\downarrow 0$. We will quantify this notion in terms of the logarithmic derivative of $F_{\scriptscriptstyle Y}^{-1}$, which will become large as $u\downarrow 0$.

In this section, we will assume that

1.3 $$\begin{aligned} u \frac{d}{du}\log F_{\scriptscriptstyle Y}^{-1}(u)=u^{-\alpha } L(1/u), \end{aligned}$$

where $\alpha \ge 0$ and $t\mapsto L(t)$ is slowly varying as $t\rightarrow \infty$. That is, for all $a>0$, $\lim _{t \rightarrow \infty }L(at)/L(t) = 1$. In other words, we assume that $u\mapsto u \frac{d}{du}\log F_{\scriptscriptstyle Y}^{-1}(u)= \frac{d}{d(\log u)}\log F_{\scriptscriptstyle Y}^{-1}(u)$ is regularly varying as $u\downarrow 0$. Recall that a function ${\tilde{L}}: (0,\infty ) \rightarrow (0,\infty )$ is called regularly varying as $u\downarrow 0$ if $\lim _{u\downarrow 0}{\tilde{L}}(au)/{\tilde{L}}(u)$ is finite but nonzero for all $a>0$.

Define a sequence $s_n$ by setting $u=1/n$:

1.4 $$\begin{aligned} s_n = \left. u \frac{d}{du}\log F_{\scriptscriptstyle Y}^{-1}(u) \right| _{u=1/n} = \frac{(F_{\scriptscriptstyle Y}^{-1})'(1/n)}{nF_{\scriptscriptstyle Y}^{-1}(1/n)}. \end{aligned}$$

The asymptotics of the sequence $(s_n)_n$ quantify how heavy-tailed the edge-weight distribution is. For instance, an identically constant sequence, say $s_n=s$, corresponds to a pure power law $F_{\scriptscriptstyle Y}(y)=y^{1/s}$, $F_{\scriptscriptstyle Y}^{-1}(u)=u^s$; larger values of *s* correspond to heavier-tailed distributions.

In this paper, we are interested in the regime where $s_n\rightarrow \infty$, which corresponds to a very heavy-tailed distribution function $F_{\scriptscriptstyle Y}(y)$ that decays to 0 slower than any power of *y*, as $y\downarrow 0$.

To describe our scaling results, define

1.5 $$\begin{aligned} u_n(x) = F_{\scriptscriptstyle Y}^{-1}(x/n). \end{aligned}$$

Then, for i.i.d. random variables $(Y_i)_{i \in {\mathbb {N}}}$ with distribution function $F_{\scriptscriptstyle Y}$,

1.6 $$\begin{aligned} {{\mathbb {P}}}\Bigl ( \min _{i\in [n]} Y_i\le u_n(x) \Bigr ) \rightarrow 1-{\mathrm e}^{-x}. \end{aligned}$$

In view of (1.6), the family $(u_n(x))_{x\in (0,\infty )}$ are the *characteristic values* for $\min _{i\in [n]} Y_i$. See [22] for a detailed discussion of extreme value theory.

#### Theorem 1.1

(Weight and hopcount—regularly-varying logarithmic derivatives) Suppose that the edge weights $$(Y_e^{\scriptscriptstyle (K_n)})_{e \in E(K_n)}$$ follow an *n*-independent distribution $$F_{\scriptscriptstyle Y}$$ that satisfies (1.3). If the sequence $$(s_n)_n$$ from (1.4) satisfies $$s_n/\log \log n\rightarrow \infty $$ and $$s_n=o(n^{1/3}),$$ then there exist sequences $$(\lambda _n)_n$$ and $$(\phi _n)_n$$ with $$\phi _n/s_n \rightarrow 1,$$$$\lambda _n u_n(1) \rightarrow {\mathrm e}^{-\gamma },$$ where $$\gamma $$ is Euler’s constant, such that

1.7 $$\begin{aligned} nF_{\scriptscriptstyle Y}\Big (W_n-\frac{1}{\lambda _n}\log {(n/s_n^3)}\Big )&{\mathop {\longrightarrow }\limits ^{d}}M^{\scriptscriptstyle (1)}\vee M^{\scriptscriptstyle (2)}, \end{aligned}$$

1.8 $$\begin{aligned} \frac{H_n-\phi _n\log {(n/s_n^3)}}{\sqrt{s_n^2\log {(n/s_n^3)}}}&{\mathop {\longrightarrow }\limits ^{d}}Z. \end{aligned}$$

Here *Z* is standard normal, and $M^{\scriptscriptstyle (1)}, M^{\scriptscriptstyle (2)}$ are i.i.d. random variables for which ${{\mathbb {P}}}(M^{\scriptscriptstyle (j)}\le x)$ is the survival probability of a Poisson Galton–Watson branching process with mean *x*.

Let us discuss the result in Theorem 1.1 in more detail. Under the hypotheses of Theorem 1.1, $u_n(x)$ varies heavily in *x* in the sense in that $u_n(x+\delta )/u_n(x)\rightarrow \infty$ for every $x,\delta >0$. Consequently, the extreme values are widely separated, which is characteristic of the *strong disorder regime*.

We see in (1.7) that $W_n-\frac{1}{\lambda _n}\log {(n/s_n^3)}\approx u_n(M^{\scriptscriptstyle (1)}\vee M^{\scriptscriptstyle (2)})$, which means that the weight of the smallest-weight path has a deterministic part $\frac{1}{\lambda _n}\log {(n/s_n^3)}$, while its random fluctuations are of the same order of magnitude as some of the typical values for the minimal edge weight adjacent to vertices 1 and 2. For $j \in \left\{ 1,2\right\}$, one can think of $M^{\scriptscriptstyle (j)}$ as the time needed to “escape” from the local neighborhood of vertex *j*. The sequences $(\lambda _n)_n$ and $(\phi _n)_n$ will be identified in (3.17)–(3.18), subject to slightly stronger assumptions.

The optimal paths in Theorem 1.1 are *long paths* because the asymptotic mean of the path length $H_n$ in (1.8) is of larger order than $\log n$, the path length that arises in many random graph contexts. See Sect. 2.2 for a comprehensive literature overview. The following example collects some edge-weight distributions that are covered by Theorem 1.1:

#### Example 1.2

(*Examples of weight distributions*)

- Let $a,\gamma >0$. Take $Y_e^{\scriptscriptstyle (K_n)} \overset{d}{=}\exp (-a E^\gamma )$, for which $\log F_{\scriptscriptstyle Y}^{-1}(u)=-a(\log (1/u))^\gamma$ and
  1.9 $$\begin{aligned} s_n = a\gamma (\log n)^{\gamma -1}. \end{aligned}$$
  The hypotheses of Theorem 1.1 are satisfied whenever $\gamma >1$.
- Let $a,\gamma >0$. Take $Y_e^{\scriptscriptstyle (K_n)} \overset{d}{=}U^{a(\log (1+\log (1/U)))^\gamma }$, for which $\log F_{\scriptscriptstyle Y}^{-1}(u)=a\log u (\log (1+\log (1/u)))^\gamma$ and
  1.10 $$\begin{aligned} s_n = a(\log (1+\log n))^\gamma + a\gamma \frac{\log n}{1+\log n}(\log (1+\log n))^{\gamma -1}. \end{aligned}$$
  We note that $s_n\sim a(\log \log n)^\gamma$ as $n\rightarrow \infty$. The hypotheses of Theorem 1.1 are satisfied whenever $\gamma >1$. We shall see, however, that the conclusions of Theorem 1.1 also hold when $0<\gamma \le 1$; see Sect. 2.1 and Lemma 4.8.
- Let $a,\beta >0$. Take $Y_e^{\scriptscriptstyle (K_n)} \overset{d}{=}\exp (-a U^{-\beta }/\beta )$, for which $\log F_{\scriptscriptstyle Y}^{-1}(u) = -au^{-\beta } /\beta$ and
  1.11 $$\begin{aligned} s_n = a n^\beta . \end{aligned}$$
  The hypotheses of Theorem 1.1 are satisfied when $0<\beta <1/3$. When $\beta \ge 1/3$, we conjecture that the hopcount scaling (1.8) fails; see the discussion in Sect. 2.2. An analogue of the weight convergence (1.7) holds in a modified form; see [Part I, Theorem 1.1 and Example 1.4 (c)].

Notice that every sequence $(s_n)_n$ of the form $s_n=n^{\alpha } L(n)$, for $\alpha \ge 0$ and *L* slowly varying at infinity, can be obtained from a distribution by taking $\log F_{\scriptscriptstyle Y}^{-1}(u)=\int u^{-1-\alpha }L(1/u)du$, i.e., the indefinite integral of the function $u\mapsto u^{-1-\alpha }L(1/u)$. In Sect. 2.1 we will weaken the requirement $s_n /\log \log n\rightarrow \infty$ to the requirement $s_n\rightarrow \infty$ subject to an additional regularity assumption.

### First Passage Percolation with *n*-Dependent Edge Weights

In Theorem 1.1, we started with a fixed edge-weight distribution and extracted a specific sequence $(s_n)_n$. For an essentially arbitrary distribution [subject to the relatively modest regular variation assumption in (1.3)], its FPP properties are fully encoded, at least for the purposes of the conclusions of Theorem 1.1, by the scaling properties of this sequence $(s_n)_n$. Thus, Theorem 1.1 shows the common behaviour of a *universality class* of edge-weight distributions, and shows that this universality class is described in terms of a sequence of real numbers $(s_n)_n$ and its scaling behaviour.

In this section, we reverse this setup. We take as input a sequence $(s_n)_n$ and consider the *n*-dependent edge-weight distribution

1.12 $$\begin{aligned} Y_e^{\scriptscriptstyle (K_n)} \overset{d}{=}E^{s_n}, \end{aligned}$$

where *E* is exponentially distributed with mean 1. (For legibility, our notation will not indicate the implicit dependence of $Y_e^{\scriptscriptstyle (K_n)}$ on *n*.) Then the conclusions of Theorem 1.1 hold verbatim:

#### Theorem 1.3

(Weight and hopcount—*n*-dependent edge weights) Let $$Y_e^{\scriptscriptstyle (K_n)}\overset{d}{=}E^{s_n},$$ where $$(s_n)_n$$ is a positive sequence with $$s_n \rightarrow \infty ,$$$$s_n=o(n^{1/3}).$$ Then

1.13 $$\begin{aligned} n\Big (W_n-\frac{1}{n^{s_n} \Gamma (1+1/s_n)^{s_n}}\log {(n/s_n^3)}\Big )^{1/s_n} {\mathop {\longrightarrow }\limits ^{d}}M^{\scriptscriptstyle (1)}\vee M^{\scriptscriptstyle (2)}, \end{aligned}$$

and

1.14 $$\begin{aligned} \frac{H_n-s_n\log {(n/s_n^3)}}{\sqrt{s_n^2\log {(n/s_n^3)}}}{\mathop {\longrightarrow }\limits ^{d}}Z, \end{aligned}$$

where *Z* is standard normal and $M^{\scriptscriptstyle (1)}, M^{\scriptscriptstyle (2)}$ are i.i.d. random variables for which ${{\mathbb {P}}}(M^{\scriptscriptstyle (j)}\le x)$ is the survival probability of a Poisson Galton–Watson branching process with mean *x*.

We note that Theorem 1.3 resolves a conjecture in [11]. This problem is closely related to the problem of *strong disorder* on the complete graph, and has attracted considerable attention in the physics literature [18, 23, 33]. The convergence in (1.13) was proved in [Part I, Theorem 1.5 (a)] without the subtraction of the term $\frac{1}{n^{s_n} \Gamma (1+1/s_n)^{s_n}}\log {(n/s_n^3)}$ in the argument, and under the stronger assumption that $s_n/\log \log {n}\rightarrow \infty .$^1^

The edge-weight distribution in Theorem 1.3 allows for a simpler intuitive explanation of (1.7), while the convergence (1.14) verifies the heuristics for the strong disorder regime in [11, Sect. 1.4]. See Remark 4.6 for a discussion of the relation between these two results. As mentioned in Sect. 1.1, strong disorder here refers to the fact that when $s_n\rightarrow \infty$ the values of the random weights $E_e^{s_n}$ depend strongly on the disorder $(E_e)_{e\in E({\mathcal {G}})}$, making small values increasingly more, and large values increasingly less, favorable. Mathematically, the elementary limit

1.15 $$\begin{aligned} \lim _{s\rightarrow \infty }(x_1^s+x_2^s)^{1/s}=x_1\vee x_2 \end{aligned}$$

expresses the convergence of the $\ell ^s$ norm towards the $\ell ^\infty$ norm and establishes a relationship between the weak disorder regime and the strong disorder regime of FPP.

Remarkably, a similar argument actually also applies to Theorem 1.1, exemplifying that these settings are in the same universality class. Indeed, Theorem 1.3 shows that *n*-dependent distributions can be understood in the same framework as the *n*-independent distributions in Example 1.2. We next explain this comparison and generalize our results further by explaining the universal picture behind them.

## The Universal Picture

In Sect. 2.1, we generalize the results in Theorems 1.1 and 1.3 to a larger class of edge weights and provide a common language that allows us to prove these results in one go. Having reached this higher level of abstraction, in Sect. 2.2, we will embed the results achieved here in the wider picture of universality classes of FPP, and provide conjectures or results how to describe all universality classes and what the scaling behaviour of each of them might be. Links to the relevant literature and existing results are provided. For a short guide to notation, see p. 81.

### Description of the Class of Edge Weights to Which Our Results Apply

In this section, we describe a general framework containing both Theorem 1.1 as well as Theorem 1.3. This framework, which is in terms of i.i.d. exponential random variables, determines the precise conditions that the edge weights need to satisfy for the results in Theorems 1.1–1.3 to apply. Interestingly, due to the parametrization in terms of exponential random variables, this general framework also provides a clear link between the near-critical Erdős–Rényi random graph and our first passage percolation problem where the lower extremes of the edge-weight distribution are highly separated. Finally and conveniently, this framework allows us to prove these theorems simultaneously. In particular, both the *n*-independent edge weights in Theorem 1.1, as well as the *n*-dependent ones in Theorem 1.3, are key examples of the class of edge weights that we will study in this paper.

For fixed *n*, the edge weights $(Y_e^{\scriptscriptstyle (K_n)})_{e\in E(K_n)}$ are independent for different *e*. However, there is no requirement that they are independent over *n*, and in fact in Sect. 5, we will produce $Y_e^{\scriptscriptstyle (K_n)}$ using a fixed source of randomness not depending on *n*. Therefore, it will be useful to describe the randomness on the edge weights $((Y_e^{\scriptscriptstyle (K_n)})_{e\in E(K_n)}:n \in {\mathbb {N}})$ uniformly across the sequence. It will be most useful to give this description in terms of exponential random variables. Fix independent exponential mean 1 variables $(X_e^{\scriptscriptstyle (K_n)})_{e\in E(K_n)}$, and define

2.1 $$\begin{aligned} Y_e^{\scriptscriptstyle (K_n)}=g(X_e^{\scriptscriptstyle (K_n)}), \end{aligned}$$

where $g:(0,\infty )\rightarrow (0,\infty )$ is a strictly increasing function. The relation between *g* and the distribution function $F_{\scriptscriptstyle Y}$ is given by

2.2 $$\begin{aligned} F_{\scriptscriptstyle Y}(y)=1-{\mathrm e}^{-g^{-1}(y)}\quad \text {and} \quad g(x)=F_{\scriptscriptstyle Y}^{-1} \left( 1-{\mathrm e}^{-x} \right) . \end{aligned}$$

We define

2.3 $$\begin{aligned} f_n(x)=g(x/n)=F_{\scriptscriptstyle Y}^{-1} \left( 1-{\mathrm e}^{-x/n} \right) . \end{aligned}$$

Let $Y_1,\dotsc ,Y_n$ be i.i.d. with $Y_i=g(E_i)$ as in (2.1). Since *g* is increasing,

2.4 $$\begin{aligned} \min _{i\in [n]}Y_i=g\bigl ( \min _{i\in [n]} E_i \bigr ) \overset{d}{=}g(E/n)=f_n(E). \end{aligned}$$

Because of this convenient relation between the edge weights $Y_e^{\scriptscriptstyle (K_n)}$ and exponential random variables, we will express our hypotheses about the distribution of the edge weights in terms of conditions on the functions $f_n(x)$ as $n\rightarrow \infty$.

Consider first the case $Y_e^{\scriptscriptstyle (K_n)} \overset{d}{=}E^{s_n}$ from Theorem 1.3. From (2.1), we have $g(x)=g_n(x)=x^{s_n}$, so that (2.3) yields

2.5 $$\begin{aligned} \text {for } Y_e^{\scriptscriptstyle (K_n)} \overset{d}{=}E^{s_n}, \quad f_n(x)=(x/n)^{s_n}=f_n(1)x^{s_n}. \end{aligned}$$

Thus, (2.4)–(2.5) show that the parameter $s_n$ measures the *relative sensitivity* of $\min _{i\in [n]}Y_i$ to fluctuations in the variable *E*. In general, we will have $f_n(x)\approx f_n(1)x^{s_n}$ if *x* is appropriately close to 1 and $s_n\approx f_n'(1)/f_n(1)$. These observations motivate the following conditions on the functions $(f_n)_n$, which we will use to relate the distributions of the edge weights $Y_e^{\scriptscriptstyle (K_n)}$, $n\in {\mathbb {N}}$, to a sequence $(s_n)_n$:

#### Condition 2.1

(Scaling of $f_n$) For every $x\ge 0,$

2.6 $$\begin{aligned} \frac{f_n(x^{1/s_n})}{f_n(1)} \rightarrow x. \end{aligned}$$

Even though we will rely on Condition 2.1 when $s_n\rightarrow \infty$ and $s_n=o(n^{1/3})$, we strongly believe that the scaling of the sequence $(s_n)_n$ actually *characterises* the universality classes, in the sense that the behaviour of $H_n$ and $W_n$ is similar for edge weights for $(s_n)_n$ with similar scaling behaviour, and different for sequences that have different scaling. We elaborate on this in Sect. 2.2.1, where we identify eight different universality classes and the expected and/or proved results in them.

#### Condition 2.2

(Density bound for small weights) There exist $$\varepsilon _0>0,$$$$\delta _0\in \left( 0,1\right] $$ and $$n_0 \in {\mathbb {N}}$$ such that

2.7 $$\begin{aligned} \varepsilon _0s_n \le \frac{xf_n'(x)}{f_n(x)}\le s_n/\varepsilon _0, \quad \text {whenever} \quad 1-\delta _0\le x\le 1\quad \text {and}\quad n\ge n_0. \end{aligned}$$

#### Condition 2.3

(Density bound for large weights)

- For all $R>1,$ there exist $\varepsilon >0$ and $n_0\in {\mathbb {N}}$ such that for every $1\le x\le R,$ and $n \ge n_0,$
  2.8 $$\begin{aligned} \frac{xf_n'(x)}{f_n(x)}\ge \varepsilon s_n. \end{aligned}$$
- For all $C>1,$ there exist $\varepsilon >0$ and $n_0\in {\mathbb {N}}$ such that (2.8) holds for every $n\ge n_0$ and every $x\ge 1$ satisfying $f_n(x)\le C f_n(1) \log n.$

Notice that Condition 2.1 implies that $f_n(1) \sim u_n(1)$ [recall the definition of $u_n(x)$ in (1.5)] whenever $s_n=o(n)$. Indeed, by (2.3) we can write $u_n(1)=f_n(x_n^{1/s_n})$ for $x_n = (-n \log (1-1/n))^{s_n}$. Since $s_n=o(n)$, we have $x_n=1-o(1)$ and the monotonicity of $f_n$ implies that $f_n(x_n^{1/s_n})/f_n(1)\rightarrow 1$. We remark also that (1.6) remains valid if $u_n(x)$ is replaced by $f_n(x)$.

We are now in a position to state our main theorem:

#### Theorem 2.4

(Weight and hopcount—general edge weights) Assume that Conditions 2.1–2.3 hold for a positive sequence $$(s_n)_n$$ with $$s_n\rightarrow \infty $$ and $$s_n=o(n^{1/3}).$$ Then there exist sequences $$(\lambda _n)_n$$ and $$(\phi _n)_n$$ such that $$\phi _n/s_n \rightarrow 1,$$$$\lambda _n f_n(1) \rightarrow {\mathrm e}^{-\gamma },$$ where $$\gamma $$ is Euler’s constant, and

2.9 $$\begin{aligned} f_n^{-1}\Big (W_n-\frac{1}{\lambda _n}\log {(n/s_n^3)}\Big )&{\mathop {\longrightarrow }\limits ^{d}}M^{\scriptscriptstyle (1)}\vee M^{\scriptscriptstyle (2)}, \end{aligned}$$

2.10 $$\begin{aligned} \frac{H_n-\phi _n\log {(n/s_n^3)}}{\sqrt{s_n^2\log {(n/s_n^3)}}}&{\mathop {\longrightarrow }\limits ^{d}}Z, \end{aligned}$$

where *Z* is standard normal, and $M^{\scriptscriptstyle (1)}, M^{\scriptscriptstyle (2)}$ are i.i.d. random variables for which ${{\mathbb {P}}}(M^{\scriptscriptstyle (j)}\le x)$ is the survival probability of a Poisson Galton–Watson branching process with mean *x*. The convergences in (2.9)–(2.10) hold jointly and the limiting random variables are independent.

The sequences $(\lambda _n)_n$ and $(\phi _n)_n$ are identified in (3.17)–(3.18), subject to the additional Condition 2.6. The proof of Theorem 2.4 is given in Sect. 3.7.

**Relation between** Theorem 2.4**and** **Theorems** 1.1**and** 1.3. Theorems 1.1 and 1.3 follow from Theorem 2.4: in the case $Y_e^{\scriptscriptstyle (K_n)}\overset{d}{=}E^{s_n}$ from Theorem 1.3, (2.6)–(2.8) hold identically with $\varepsilon _0=\varepsilon =1$ and we explicitly compute $\lambda _n=n^{s_n} \Gamma (1+1/s_n)^{s_n}$ and $\phi _n=s_n$ in Example 6.1. We will prove in Lemma 4.8 that the distributions in Theorem 1.1 satisfy the assumptions of Theorem 2.4. The convergence (1.7) in Theorem 1.1 is equivalent to (2.9) in Theorem 2.4 by the observation that, for any non-negative random variables $(T_n)_n$ and ${\mathcal {M}}$,

2.11 $$\begin{aligned} nF_{\scriptscriptstyle Y}(T_n)\rightarrow {\mathcal {M}}\iff f_n^{-1}(T_n)\rightarrow {\mathcal {M}}, \end{aligned}$$

where the convergence is in distribution, in probability or almost surely; see e.g. [Part I, Lemma 5.5] for an example.

The following example describes a generalization of Theorem 1.3:

#### Example 2.5

Let $(s_n)_n$ be a positive sequence with $s_n \rightarrow \infty$, $s_n=o(n^{1/3})$. Let *Z* be a positive-valued continuous random variable with distribution function *G* such that $G'(z)$ exists and is continuous at $z=0$ with $G'(0)>0$ [with $G'(0)$ interpreted as a right-hand derivative]. Take $Y_e^{\scriptscriptstyle (K_n)} \overset{d}{=}Z^{s_n}$, i.e., $F_{\scriptscriptstyle Y}(y)=G(y^{1/s_n})$. Then Conditions 2.1–2.3 hold and Theorem 2.4 applies.

For instance, we can take *Z* to be a uniform distribution on an interval (0, *b*), for any $b>0$. We give a proof of this assertion in Lemma 4.8.

Condition 2.3 can be strengthened to the following condition that will be equivalent for our purposes:

#### Condition 2.6

(Extended density bound) There exist $\varepsilon _0>0$ and $n_0 \in {\mathbb {N}}$ such that

2.12 $$\begin{aligned} \frac{xf_n'(x)}{f_n(x)}\ge \varepsilon _0s_n \quad \text {for every }x\ge 1, n \ge n_0. \end{aligned}$$

#### Lemma 2.7

It suffices to prove Theorem 2.4 assuming Conditions 2.1, 2.2 and 2.6.

Lemma 2.7, which is proved in Sect. 4.3, reflects the fact that the upper tail of the edge-weight distribution does not substantially influence the first passage percolation problem.

Henceforth, except where otherwise noted, we will assume Conditions 2.1, 2.2 and 2.6. We will reserve the notation $\varepsilon _0, \delta _0$ for some fixed choice of the constants in Conditions 2.2 and 2.6, with $\varepsilon _0$ chosen small enough to satisfy both conditions.

### Discussion of Our Results

In this section we discuss our results and state open problems.

#### The Universality Class in Terms of $s_n$

In Sect. 2.1, we have described an edge-weight universality class in terms of $s_n$. In this paper, we investigate the case where $s_n\rightarrow \infty$ with $s_n=o(n^{1/3})$. We conjecture that all universality classes can be described in terms of the scaling behaviour of the sequence $(s_n)_n$ and below identify the eight universality classes that describe the different scaling behaviours. These eight cases are defined by how fast $s_n\rightarrow 0$ (this gives rise to four cases), the case where $s_n$ converges to a positive and finite constant, and by how $s_n\rightarrow \infty$ (giving rise to three cases, including the one that is studied in this paper). We believe that this paper represents a major step forward in this direction in that it describes the scaling behaviour in a large regime of $(s_n)_n$ sequences. We next describe the eight scaling regimes of $s_n$ and the results proved and/or predicted for them. We conjecture that these eight cases describe *all* universality classes for FPP on the complete graph, and it would be of interest to make this universal picture complete. Let us now describe these eight cases.

**The regime** $s_n \rightarrow 0$. In view of (2.5), for $s_n \rightarrow 0$, the first passage percolation problem approximates the graph metric, where the approximation is stronger the faster $s_n$ tends to zero. We distinguish four different scaling regimes according to how fast $s_n \rightarrow 0$:

(i) Firstly, $s_n\log {n}\rightarrow \gamma \in \left[ 0,\infty \right)$: the case that $Y\overset{d}{=}E^{-\gamma }$ for $\gamma \in (0,\infty )$ falls into this class with $s_n=\gamma /\log n$ (see [20, Sect. 1.2]) and was investigated in [16], where it is proved that $H_n$ is concentrated on at most two values. For the case of *n*-dependent edge weights $Y_e^{\scriptscriptstyle (K_n)} \overset{d}{=}E^{s_n}$, it was observed in [20] that when $s_n \log n$ converges to $\gamma$ fast enough, the methods of [16] can be imitated and the concentration result for the hopcount continues to hold.
(ii) When $s_n\log {n}\rightarrow \infty$ but $s_n^2\log {n}\rightarrow 0$, the variance factor $s_n^2 \log (n/s_n^3)$ from the central limit theorem (CLT) in (2.10) tends to zero. Since $H_n$ is integer-valued, it follows that (2.10) must fail in this case. First order asymptotics are investigated in [20], and it is shown that $H_n/(s_n\log {n}){\mathop {\longrightarrow }\limits ^{\scriptscriptstyle {{\mathbb {P}}}}}1$, $W_n/(u_n(1) s_n\log {n}){\mathop {\longrightarrow }\limits ^{\scriptscriptstyle {{\mathbb {P}}}}}{\mathrm e}$. It is tempting to conjecture that there exists an integer $k=k_n\approx s_n\log {n}$ such that $H_n\in \left\{ k_n,k_n+1\right\}$ whp.
(iii) The regime where $s_n\log n \rightarrow \infty$ but $s_n^2\log n\rightarrow \gamma \in (0,\infty )$ corresponds to a *critical window* between the one- or two-point concentration conjectured in (ii) and the CLT scaling conjectured in (iv). It is natural to expect that $H_n-\lfloor \phi _n\log n\rfloor$ is tight for an appropriately chosen sequence $\phi _n\sim s_n$, although the distribution of $H_n-\lfloor \phi _n \log n\rfloor$ might only have subsequential limits because of integer effects. Moreover, we would expect these subsequential limits in distribution to match with (ii) and (iv) in the limits $\gamma \rightarrow 0$ or $\gamma \rightarrow \infty$, respectively.
(iv) When $s_n \rightarrow 0, s_n^2\log {n}\rightarrow \infty$, we conjecture that the CLT for the hopcount in Theorem 2.4 remains true, and that $u_n(1)^{-1}W_n-\frac{1}{\lambda _n}\log n$ converges to a Gumbel distribution for a suitable sequence $\lambda _n$ and $u_n(1)$. Unlike in the fixed *s* case, we expect no martingale limit terms to appear in the limiting distribution.

**The fixed** *s* **regime**. The fixed *s* regime was investigated in [11] in the case where $Y\overset{d}{=}E^s$, and describes the boundary case between $s_n\rightarrow 0$ and $s_n\rightarrow \infty$. We conjecture that for other sequences of random variables for which Condition 2.1 is valid for some $s\in (0,\infty )$ the CLT for the hopcount remains valid, while there exist *V* and $\lambda (s)$ depending on the distribution *Y* such that $u_n(1)^{-1}W_n-\frac{1}{\lambda (s)}\log {(n/s^3)}{\mathop {\longrightarrow }\limits ^{d}}V$. In general, *V* will not be equal to $(M^{\scriptscriptstyle (1)}\vee M^{\scriptscriptstyle (2)})^s$, see for example [11]. Instead, it is described by a sum of different terms involving Gumbel distributions and the martingale limit of a certain continuous-time branching process depending on the distribution. Our proof is inspired by the methods developed in [11]. The CLT for $H_n$ in the fixed *s* regime can be recovered from our proofs; in fact, the reasoning in that case simplifies considerably compared to our more general setup.

The results in [11] match up nicely with ours. Indeed, in [11], it was shown that

2.13 $$\begin{aligned} f_n^{-1} \big (W_n-\log {n}/\lambda (s)\big ) {\mathop {\longrightarrow }\limits ^{d}}\lambda (s)^{-1/s} \big (\Lambda _{1,2}-\log L_s^{\scriptscriptstyle (1)}-\log L_s^{\scriptscriptstyle (2)}-\log (1/s)\big )^{1/s}, \end{aligned}$$

where $\lambda (s)=\Gamma (1+1/s)^s$, $\Lambda _{1,2}$ is a Gumbel variable so that ${{\mathbb {P}}}(\Lambda _{1,2}\le x)={\mathrm e}^{-{\mathrm e}^{-x}}$ and $L_s^{\scriptscriptstyle (1)}, L_s^{\scriptscriptstyle (2)}$ are two independent copies of the random variable $L_s$ with ${{\mathbb {E}}}(L_s)=1$ solving the distributional equation

2.14 $$\begin{aligned} L_s{\mathop {=}\limits ^{d}}\sum _{i\ge 1} {\mathrm e}^{-\lambda (s) (E_1+\cdots +E_i)^{s}} L_{s,i}, \end{aligned}$$

where $(L_{s,i})_{i\ge 1}$ are i.i.d. copies of $L_s$ and $(E_i)_{i\ge 1}$ are i.i.d. exponentials with mean 1. We claim that the right-hand side of (2.13) converges to $M^{\scriptscriptstyle (1)}\vee M^{\scriptscriptstyle (2)}$ as $s\rightarrow \infty$, where $M^{\scriptscriptstyle (1)}, M^{\scriptscriptstyle (2)}$ are as in Theorem 2.4. This is equivalent to the statement that $(-\log L_s^{\scriptscriptstyle (j)})^{1/s} {\mathop {\longrightarrow }\limits ^{d}}M^{\scriptscriptstyle (j)}$ as $s\rightarrow \infty$. Assume that $(-\log L_s^{\scriptscriptstyle (j)})^{1/s}$ converges in distribution to a random variable ${\mathcal {M}}$. Then

2.15 $$\begin{aligned} \lim _{s \rightarrow \infty }\Big (-\log \Big (\sum _{i\ge 1} {\mathrm e}^{-\lambda (s) (E_1+\cdots +E_i)^{s}} L_{s,i}\Big )\Big )^{1/s}&= \min _{i\ge 1} \lim _{s\rightarrow \infty } \Big (\lambda (s) (E_1+\cdots +E_i)^{s} -\log L_{s,i}\Big )^{1/s}\nonumber \\&=\min _{i\ge 1}\Big ((E_1+\cdots +E_i)\vee \big (\lim _{s\rightarrow \infty } (-\log L_{s,i})^{1/s}\big )\Big ), \end{aligned}$$

and using (2.14) we deduce that ${\mathcal {M}}$ is the solution of the equation

2.16 $$\begin{aligned} {\mathcal {M}}\overset{d}{=}\min _{i\ge 1} (E_1+\cdots +E_i)\vee {\mathcal {M}}_i, \end{aligned}$$

where $({\mathcal {M}}_i)_{i\ge 1}$ are i.i.d. copies of ${\mathcal {M}}$ independent of $(E_i)_{i\ge 1}$. The unique solution to (2.16) is the random variable with ${{\mathbb {P}}}({\mathcal {M}}\le x)$ being the survival probability of a Poisson Galton–Watson process with mean *x*, so that ${\mathcal {M}}{\mathop {=}\limits ^{d}}M^{\scriptscriptstyle (1)}$.

**The regime** $s_n\rightarrow \infty$. The regime $s_n\rightarrow \infty$ can be further separated into three cases.

(i) Firstly, the case where $s_n\rightarrow \infty$ with $s_n/n^{1/3}\rightarrow 0$ is the main topic of this paper.
(ii) Secondly, the regime where $s_n/n^{1/3}\rightarrow \gamma \in (0,\infty )$ corresponds to the *critical window* between the minimal spanning tree case discussed below and the case (i) studied here. It is natural to expect (see also Theorems 1.1 and 1.3) that $H_n/n^{1/3}$ converges to a non-trivial limit that depends sensitively on $\gamma$, and that, when $\gamma \rightarrow 0$ and $\gamma \rightarrow \infty$ matches up with the cases (i) and (iii) discussed above and below, respectively.
(iii) Finally, the regime $s_n/n^{1/3} \rightarrow \infty$. Several of our methods do not extend to the case where $s_n/n^{1/3} \rightarrow \infty$; indeed, we conjecture that the CLT in Theorem 2.4 ceases to hold in this regime. In this case, our proof clearly suggests that first passage percolation (FPP) on the complete graph is closely approximated by invasion percolation (IP) on the Poisson-weighted infinite tree (PWIT), studied in [2], whenever $s_n\rightarrow \infty$, see also [21]. It it tempting to predict that $H_n/n^{1/3}$ converges to the same limit as the graph distance between two vertices for the minimal spanning tree on the complete graph as identified in [3].

#### First Passage Percolation on Random Graphs

FPP on random graphs has attracted considerable attention in the past years, and our research was strongly inspired by its studies. In [17], the authors show that for the configuration model with finite-variance degrees (and related graphs) and edge weights with a continuous distribution not depending on *n*, there exists only a *single* universality class. Indeed, if we define $W_n$ and $H_n$ to be the weight of and the number of edges in the smallest-weight path between two uniform vertices in the graph, then there exist positive, finite constants $\alpha , \beta , \lambda$ and sequences $(\alpha _n)_n,(\lambda _n)_n$, with $\alpha _n\rightarrow \alpha$, $\lambda _n\rightarrow \lambda$, such that $W_n-(1/\lambda _n)\log {n}$ converges in distribution, while $H_n$ satisfies a CLT with asymptotic mean $\alpha _n \log {n}$ and asymptotic variance $\beta \log {n}$.

Related results for exponential edge weights appear for the Erdős–Rényi random graph in [15], to certain inhomogeneous random graphs in [28] and to the small-world model in [30]. The diameter of the weighted graph is studied in [6], and relations to competition on *r*-regular graphs are examined in [7]. Finally, the smallest-weight paths with most edges from a single source or between any pair in the graph are investigated in [5].

We conjecture that our results are closely related to FPP on random graphs with *infinite-variance degrees*. Such graphs, sometimes called *scale-free* random graphs, have been suggested in the networking community as appropriate models for various real-world networks. See [8, 31] for extended surveys of real-world networks, and [19, 24, 32] for more details on random graph models of such real-world networks. FPP on infinite-variance random graphs with exponential weights was first studied in [13, 14], of which the case of finite-mean degrees studied in [14] is most relevant for our discussion here. There, it was shown that a linear transformation of $W_n$ converges in distribution, while $H_n$ satisfies a CLT with asymptotic mean and variance $\alpha \log {n}$, where $\alpha$ is a simple function of the power-law exponent of the degree distribution of the configuration model. Since the configuration model with infinite-variance degrees whp contains a complete graph of size a positive power of *n*, it can be expected that the universality classes on these random graphs are closely related to those on the complete graph $K_n$. In particular, the strong universality result for finite-variance random graphs is false, which can be easily seen by observing that for the weight distribution $1+E$, where *E* is an exponential random variable, the hopcount $H_n$ is of order $\log \log {n}$ (as for the graph distance [26]), rather than $\log {n}$ as it is for exponential weights. See [9] for two examples proving that strong universality indeed fails in the infinite-variance setting, and [4, 10] for further results. The area has attracted substantial attention through the work of Komjáthy and collaborators, see also [25, 29] for recent work in geometric contexts.

#### Extremal Functionals for FPP on the Complete Graph

Many more fine results are known for FPP on the complete graph with exponential edge weights. In [27], the weak limits of the rescaled path weight and flooding are determined, where the flooding is the maximal smallest weight between a source and all vertices in the graph. In [12] the same is performed for the diameter of the graph. It would be of interest to investigate the weak limits of the flooding and diameter in our setting.

## Detailed Results, Overview and Classes of Edge Weights

In this section, we provide an overview of the proof of our main results.

This section is organised as follows. In Sect. 3.1, we explain how FPP clusters can be described in terms of an appropriate *exploration process*, both from one as well as from two sources. In Sect. 3.2, we discuss how this exploration process can be coupled to first passage percolation on the Poisson-weighted infinite tree (PWIT). In Sect. 3.3, we interpret the FPP dynamics on the PWIT as a continuous-time branching process, and study one- and two-vertex characteristics associated with it. The two-vertex characteristics are needed since we explore from two sources. Due to the near-critical behavior of the involved branching processes, the FPP clusters may run at rather different speeds, and we need to make sure that their sizes are comparable. This is achieved by *freezing* the fastest growing one, which is explained in detail in Sect. 3.4, both for the time at which this happens as well as the sizes of the FPP cluster at the freezing times. There, we also investigate the collision times between the two exploration processes, which correspond to (near-) shortest paths between the two sources. In Sect. 3.5, we couple FPP on the complete graph from two sources to a continuous-time branching process from which we can retrieve the FPP clusters by a *thinning* procedure. In Sect. 3.6, we use the explicit distribution of the collision edge (whether thinned or not) to derive its scaling properties, both for the time at which it occurs, as well as the generations of the vertices it consist of. Finally, in Sect. 3.7, we show that the first point of the Cox process that describes the collision edge is with high probability *unthinned* and complete the proof of our main results.

### FPP Exploration Processes

To understand smallest-weight paths in the complete graph, we study the first passage *exploration process* from one or two sources. Recall from (1.1) that $d_{K_n,Y^{\scriptscriptstyle (K_n)}}(i,j)$ denotes the total cost of the optimal path $\pi _{i,j}$ between vertices *i* and *j*.

#### One-Source Exploration Process

For a vertex $j\in V(K_n)$, let the *one-source smallest-weight tree* $\mathsf{SWT}_t^{\scriptscriptstyle (j)}$ be the connected subgraph of $K_n$ defined by

3.1 $$\begin{aligned} \begin{aligned} V(\mathsf{SWT}_t^{\scriptscriptstyle (j)})&= \left\{ i\in V(K_n):d_{K_n,Y^{\scriptscriptstyle (K_n)}}(i,j)\le t\right\} \!, \\ E(\mathsf{SWT}_t^{\scriptscriptstyle (j)})&= \left\{ e\in E(K_n):e\in \pi _{j,i}\;\text { for some }i\in V(\mathsf{SWT}_t^{\scriptscriptstyle (j)})\right\} . \end{aligned} \end{aligned}$$

Note that $\mathsf{SWT}_t^{\scriptscriptstyle (j)}$ is indeed a tree: if two optimal paths $\pi _{j,k},\pi _{j,k'}$ pass through a common vertex *i*, both paths must contain $\pi _{j,i}$ since the minimizers of (1.1) are unique. Moreover, by construction, FPP distances from the source vertex *j* can be recovered from arrival times in the process $\mathsf{SWT}_t^{\scriptscriptstyle (j)}$:

3.2 $$\begin{aligned} d_{K_n,Y^{\scriptscriptstyle (K_n)}}(i,j)=\inf \left\{ t:i\in \mathsf{SWT}^{\scriptscriptstyle (j)}_t\right\} . \end{aligned}$$

To visualize the process $(\mathsf{SWT}_t^{\scriptscriptstyle (j)})_{t\ge 0}$, think of the edge weight $Y_e^{\scriptscriptstyle (K_n)}$ as the time required for fluid to flow across the edge *e*. Place a source of fluid at *j* and allow it to spread through the graph. Then $V(\mathsf{SWT}_t^{\scriptscriptstyle (j)})$ is precisely the set of vertices that have been wetted by time *t*, while $E(\mathsf{SWT}_t^{\scriptscriptstyle (j)})$ is the set of edges along which, at any time up to *t*, fluid has flowed from a wet vertex to a previously dry vertex. Equivalently, an edge is added to $\mathsf{SWT}_t^{\scriptscriptstyle (j)}$ whenever it becomes completely wet, with the additional rule that an edge is not added if it would create a cycle.

Because fluid begins to flow across an edge only after one of its endpoints has been wetted, the *age* of a vertex—the length of time that a vertex has been wet—determines how far fluid has traveled along the adjoining edges. Given $\mathsf{SWT}_t^{\scriptscriptstyle (j)}$, the future of the exploration process will therefore be influenced by the current ages of vertices in $\mathsf{SWT}_t^{\scriptscriptstyle (j)}$, and the nature of this effect depends on the probability law of the edge weights $(Y_e^{\scriptscriptstyle (K_n)})_e$. In the sequel, for a subgraph ${\mathcal {G}}=(V({\mathcal {G}}),E({\mathcal {G}}))$ of $K_n$, we write ${\mathcal {G}}$ instead of $V({\mathcal {G}})$ for the vertex set when there is no risk of ambiguity.

#### Two-Source Exploration Process

Consider now two vertices from $K_n$, which for simplicity we take to be vertices 1 and 2. The *two-source smallest-weight tree* $\mathsf{SWT}_t^{\scriptscriptstyle (1,2)}$ is the subgraph of $K_n$ defined by

3.3 $$\begin{aligned} \mathsf{SWT}_t^{\scriptscriptstyle (1,2)} = \mathsf{SWT}_t^{\scriptscriptstyle (1,2; 1)} \cup \mathsf{SWT}_t^{\scriptscriptstyle (1,2; 2)}, \end{aligned}$$

where

3.4 $$\begin{aligned} \begin{aligned} V(\mathsf{SWT}_t^{\scriptscriptstyle (1,2; 1)})&= \left\{ i\in [n] :d_{K_n,Y^{\scriptscriptstyle (K_n)}}(1,i)\le t\text { and }d_{K_n,Y^{\scriptscriptstyle (K_n)}}(1,i)< d_{K_n,Y^{\scriptscriptstyle (K_n)}}(2,i)\right\} , \\ V(\mathsf{SWT}_t^{\scriptscriptstyle (1,2; 2)})&= \left\{ i\in [n] :d_{K_n,Y^{\scriptscriptstyle (K_n)}}(2,i)\le t\text { and }d_{K_n,Y^{\scriptscriptstyle (K_n)}}(2,i) < d_{K_n,Y^{\scriptscriptstyle (K_n)}}(1,i)\right\} , \\ E(\mathsf{SWT}_t^{\scriptscriptstyle (1,2; j)})&= \left\{ e\in E(K_n) :e\in \pi _{j,i}\text { for some }i\in V(\mathsf{SWT}_t^{\scriptscriptstyle (1,2; j)})\right\} . \end{aligned} \end{aligned}$$

In other words, $\mathsf{SWT}_t^{\scriptscriptstyle (1,2)}$ is the union, over all vertices *i* within FPP distance *t* of vertex 1 or vertex 2, of an optimal path, either $\pi _{1,i}$ or $\pi _{2,i}$ whichever has smaller weight.

Because the edge weight distribution has no atoms, no two optimal paths have the same length. It follows that, a.s., $\mathsf{SWT}_t^{\scriptscriptstyle (1,2)}$ is the union of two *vertex-disjoint* trees for all *t*. (To see this, suppose vertex *i* is closer to vertex *j* than to vertex $j'$, where $\left\{ j,j'\right\} =\left\{ 1,2\right\}$. Then, given another vertex $i'$ and a path $\pi$ passing from $i'$ to *i* to $j'$, there must be a strictly shorter path passing from $i'$ to *i* to *j*.) We note that

3.5 $$\begin{aligned} V(\mathsf{SWT}_t^{\scriptscriptstyle (1,2)}) = V(\mathsf{SWT}_t^{\scriptscriptstyle (1)}) \cup V(\mathsf{SWT}_t^{\scriptscriptstyle (2)}), \quad E(\mathsf{SWT}_t^{\scriptscriptstyle (1,2)}) \subset E(\mathsf{SWT}_t^{\scriptscriptstyle (1)})\cup E(\mathsf{SWT}_t^{\scriptscriptstyle (2)}), \end{aligned}$$

with strict containment for sufficiently large *t*.

To visualize the process $(\mathsf{SWT}_t^{\scriptscriptstyle (1,2)})_{t\ge 0}$, place sources of fluid at vertices 1 and 2 and allow both fluids to spread through the graph. Then, as before, $V(\mathsf{SWT}_t^{\scriptscriptstyle (1,2)})$ is precisely the set of vertices that have been wetted by time *t*, while $E(\mathsf{SWT}_t^{\scriptscriptstyle (1,2)})$ is the set of edges along which, at any time up to *t*, fluid has flowed from a wet vertex to a previously dry vertex. Equivalently, an edge is added to $\mathsf{SWT}_t^{\scriptscriptstyle (1,2)}$ whenever it becomes completely wet, with the additional rules that an edge is not added if it would create a cycle *or* if it would connect the two connected components of $\mathsf{SWT}_t^{\scriptscriptstyle (1,2)}$.

From the process $\mathsf{SWT}_t^{\scriptscriptstyle (1,2)}$, we can partially recover FPP distances. Denote by $T^{\scriptscriptstyle \mathsf{SWT}^{\scriptscriptstyle (1,2)}}(i)=\inf \left\{ t\ge 0:i\in \mathsf{SWT}_t^{\scriptscriptstyle (1,2)}\right\}$ the arrival time of a vertex $i\in [n]$. Then, for $j\in \left\{ 1,2\right\}$,

3.6 $$\begin{aligned} d_{K_n,Y^{\scriptscriptstyle (K_n)}}(i,j)=T^{\scriptscriptstyle \mathsf{SWT}^{\scriptscriptstyle (1,2)}}(i) \quad \text {provided that }i\in \cup _{t\ge 0}\mathsf{SWT}_t^{\scriptscriptstyle (1,2; j)}. \end{aligned}$$

More generally, observing the process $(\mathsf{SWT}_t^{\scriptscriptstyle (1,2)})_{t\ge 0}$ allows us to recover the edge weights $Y_e^{\scriptscriptstyle (K_n)}$ for all $e\in \cup _{t\ge 0} E(\mathsf{SWT}_t^{\scriptscriptstyle (1,2)})$. However, in contrast to the one-source case, the FPP distance $W_n=d_{K_n,Y^{\scriptscriptstyle (K_n)}}(1,2)$ cannot be determined by observing the process $(\mathsf{SWT}_t^{\scriptscriptstyle (1,2)})_{t\ge 0}$. Indeed, if vertices $i_1,i_2$ satisfy $i_1\in \mathsf{SWT}_t^{\scriptscriptstyle (1,2;1)}$ and $i_2\in \mathsf{SWT}_t^{\scriptscriptstyle (1,2;2)}$ for some *t*, then by construction the edge $\left\{ i_1,i_2\right\}$ between them will never be added to $\mathsf{SWT}^{\scriptscriptstyle (1,2)}$ and there is no arrival time from which to determine the edge weight $Y_{\left\{ i_1,i_2\right\} }^{\scriptscriptstyle (K_n)}$.

The optimal weight $W_n$ is the minimum value

3.7 $$\begin{aligned} W_n = \min _{i_1\in \mathsf{SWT}_\infty ^{\scriptscriptstyle (1,2;1)},i_2\in \mathsf{SWT}_\infty ^{\scriptscriptstyle (1,2;2)}} \left( T^{\scriptscriptstyle \mathsf{SWT}^{\scriptscriptstyle (1,2)}}(i_1) + Y^{\scriptscriptstyle (K_n)}_{\left\{ i_1,i_2\right\} } + T^{\scriptscriptstyle \mathsf{SWT}^{\scriptscriptstyle (1,2)}}(i_2) \right) , \end{aligned}$$

which is uniquely attained a.s. by our assumptions on the edge weights.

##### Definition 3.1

(*Collision time and edge*) The *SWT collision time* is $T_\mathrm{coll}^{\scriptscriptstyle \mathsf{SWT}}=\tfrac{1}{2}W_n$. Let $I_1^{\scriptscriptstyle \mathsf{SWT}},I_2^{\scriptscriptstyle \mathsf{SWT}}$ denote the (a.s. unique) minimizers in (3.7). The edge $\left\{ I_1^{\scriptscriptstyle \mathsf{SWT}},I_2^{\scriptscriptstyle \mathsf{SWT}}\right\}$ is called the *SWT collision edge*.

In the fluid flow description above, $T_\mathrm{coll}^{\scriptscriptstyle \mathsf{SWT}}$ is the time when the fluid from vertex 1 and the fluid from vertex 2 first collide, and this collision takes place inside the collision edge. Note that since fluid flows at rate 1 from both sides simultaneously, the overall distance is given by $W_n=2T_\mathrm{coll}^{\scriptscriptstyle \mathsf{SWT}}$.

##### Proposition 3.2

(Smallest-weight path) The end points of the collision edge are explored before the collision time, $T^{\scriptscriptstyle \mathsf{SWT}^{(1,2)}}(I_j^{\scriptscriptstyle \mathsf{SWT}}) < T_\mathrm{coll}^{\scriptscriptstyle \mathsf{SWT}}$ for $j\in \left\{ 1,2\right\} .$ The optimal path $\pi _{1,2}$ from vertex 1 to vertex 2 is the union of the unique path in $\mathsf{SWT}^{\scriptscriptstyle (1,2;1)}_{T_\mathrm{coll}^{\scriptscriptstyle \mathsf{SWT}}}$ from 1 to $I_1^{\scriptscriptstyle \mathsf{SWT}};$ the collision edge $\left\{ I_1^{\scriptscriptstyle \mathsf{SWT}},I_2^{\scriptscriptstyle \mathsf{SWT}}\right\} ;$ and the unique path in $\mathsf{SWT}^{\scriptscriptstyle (1,2;2)}_{T_\mathrm{coll}^{\scriptscriptstyle \mathsf{SWT}}}$ from $I_2^{\scriptscriptstyle \mathsf{SWT}}$ to 2. Furthermore3.8

We will not use Proposition 3.2 and the formula (3.7), which are a special case of Lemma 3.20 and Theorem 3.22. These generalizations deal with a *freezing procedure* that we will explain below. Note that the conditioning in (3.8) reflects the information about $Y_{\{i_1,i_2\}}$ gained by knowing that $i_1$ and $i_2$ belong to different connected components of $\mathsf{SWT}^{\scriptscriptstyle (1,2)}_t$: during the period of time when one vertex was explored but not the other, the fluid must not have had time to flow from the earlier-explored vertex to the later-explored vertex.

### Coupling FPP on $K_n$ to FPP on the Poisson-Weighted Infinite Tree

In this section, we state results that *couple* FPP on $K_n$ to FPP on the Poisson-weighted infinite tree (PWIT). We start by explaining the key idea, coupling of order statistics of exponentials to Poisson processes, in Sect. 3.2.1. We continue to define the PWIT in Sect. 3.2.2. We then couple FPP on $K_n$ to FPP on the PWIT, for one source in Sect. 3.2.3 and finally for two sources in Sect. 3.2.4.

#### Order Statistics of Exponentials and Poisson Processes

To study the smallest-weight tree from a vertex, say vertex 1, let us consider the time until the first vertex is added. By construction, $\min _{i\in [n]\setminus \left\{ 1\right\} } Y_{\left\{ 1,i\right\} }^{\scriptscriptstyle (K_n)} \overset{d}{=}f_n\left( \frac{n}{n-1}E\right)$ [cf. (2.4)], where *E* is an exponential random variable of mean 1. We next extend this to describe the distribution of the order statistics $Y^{(K_n)}_{(1)}< Y^{(K_n)}_{(2)}< \cdots < Y^{(K_n)}_{(n-1)}$ of the weights of edges from vertex 1 to *all* other vertices.

By (2.1) and (2.3), we can write $Y_{\left\{ 1,i\right\} }^{\scriptscriptstyle (K_n)} = f_n(E'_i )$, where $E'_i=nX_{\left\{ 1,i\right\} }^{\scriptscriptstyle (K_n)}$ are independent, exponential random variables with rate 1/*n*. We can realize $E'_i$ as the first point of a Poisson point process ${\mathcal {P}}^{\scriptscriptstyle (i)}$ with rate 1/*n*, with points $X^{\scriptscriptstyle (i)}_1<X^{\scriptscriptstyle (i)}_2<\cdots$, chosen independently for different $i=2,\ldots ,n$. We can also form the Poisson point process ${\mathcal {P}}^{\scriptscriptstyle (1)}$ with rate 1/*n*, corresponding to $i=1$, although this Poisson point process is not needed to produce an edge weight. To each point of ${\mathcal {P}}^{\scriptscriptstyle (i)}$, associate the mark *i*.

Now amalgamate all *n* Poisson point processes to form a single Poisson point process of intensity 1, with points $X_1<X_2<\cdots$. Each point $X_k$ has an associated *mark* $M_k$. By properties of Poisson point processes, given the points $X_1<X_2<\cdots$, the marks $M_k$ are chosen uniformly at random from [*n*], different marks being independent.

To complete the construction of the edge weights $Y^{\scriptscriptstyle (K_n)}_{\left\{ 1,i\right\} }$, we need to recover the first points $X^{\scriptscriptstyle (i)}_1$, for all $i=2,\ldots ,n$, from the amalgamated points $X_1<X_2<\cdots$. Thus we will *thin* a point $X_k$ when $M_k=1$ (since $i=1$ is not used to form an edge weight) or when $M_k=M_{k'}$ for some $k'<k$ (since such a point is not the first point of its corresponding Poisson point process). Then

3.9 $$\begin{aligned} (Y_{(k)}^{\scriptscriptstyle (K_n)})_{k\in [n-1]}\overset{d}{=}(f_n(X_k))_{k\in {\mathbb {N}}, \, X_k\text { unthinned}}. \end{aligned}$$

In the next step, we extend this result to the smallest-weight tree $\mathsf{SWT}^{\scriptscriptstyle (1)}$ using a relation to FPP on the Poisson-weighted infinite tree.

#### The Poisson-Weighted Infinite Tree

The Poisson-weighted infinite tree is an infinite edge-weighted tree in which every vertex has infinitely many (ordered) children. Before giving the definitions, we recall the Ulam–Harris notation for describing trees.

Define the tree ${\mathcal {T}}^{\scriptscriptstyle (1)}$ as follows. The vertices of ${\mathcal {T}}^{\scriptscriptstyle (1)}$ are given by finite sequences of natural numbers headed by the symbol $\varnothing _1$, which we write as $\varnothing _1 j_1 j_2\cdots j_k$. The sequence $\varnothing _1$ denotes the root vertex of ${\mathcal {T}}^{\scriptscriptstyle (1)}$. We concatenate sequences $v=\varnothing _1 i_1\cdots i_k$ and $w=\varnothing _1 j_1\cdots j_m$ to form the sequence $vw=\varnothing _1 i_1\cdots i_k j_1\cdots j_m$ of length $\left| vw\right| =\left| v\right| +\left| w\right| =k+m$. Identifying a natural number *j* with the corresponding sequence of length 1, the $j^\text {th}$ child of a vertex *v* is *vj*, and we say that *v* is the parent of *vj*. Write $p\left( v\right)$ for the (unique) parent of $v\ne \varnothing _1$, and $p^{k}\!\left( v\right)$ for the ancestor *k* generations before, $k\le \left| v\right|$.

We can place an edge (which we could consider to be directed) between every $v\ne \varnothing _1$ and its parent; this turns ${\mathcal {T}}^{\scriptscriptstyle (1)}$ into a tree with root $\varnothing _1$. With a slight abuse of notation, we will use ${\mathcal {T}}^{\scriptscriptstyle (1)}$ to mean both the set of vertices and the associated graph, with the edges given implicitly according to the above discussion, and we will extend this convention to any subset $\tau \subset {\mathcal {T}}^{\scriptscriptstyle (1)}$. We also write $\partial \tau =\left\{ v\notin \tau :p\left( v\right) \in \tau \right\}$ for the set of children one generation away from $\tau$.

To describe the PWIT formally, we associate weights to the edges of ${\mathcal {T}}^{\scriptscriptstyle (1)}$. By construction, we can index these edge weights by non-root vertices, writing the weights as $X=(X_v)_{v\ne \varnothing _1}$, where the weight $X_v$ is associated to the edge between *v* and its parent *p*(*v*). We make the convention that $X_{v0}=0$.

##### Definition 3.3

(*Poisson-weighted infinite tree*) The *Poisson-weighted infinite tree* (PWIT) is the random tree $({\mathcal {T}}^{\scriptscriptstyle (1)},X)$ for which $X_{vk}-X_{v(k-1)}$ is exponentially distributed with mean 1, independently for each $v\in {\mathcal {T}}^{\scriptscriptstyle (1)}$ and each $k\in {\mathbb {N}}$. Equivalently, the weights $(X_{v1},X_{v2},\ldots )$ are the (ordered) points of a Poisson point process of intensity 1 on $(0,\infty )$, independently for each *v*.

Motivated by (3.9), we study FPP on ${\mathcal {T}}^{\scriptscriptstyle (1)}$ with edge weights $(f_n(X_v))_v$:

##### Definition 3.4

(*First passage percolation on the Poisson-weighted infinite tree*) For FPP on ${\mathcal {T}}^{\scriptscriptstyle (1)}$ with edge weights $(f_n(X_v))_v$, let the FPP edge weight between $v\in {\mathcal {T}}^{\scriptscriptstyle (1)}\setminus \left\{ \varnothing _1\right\}$ and $p\left( v\right)$ be $f_n(X_v)$. The FPP distance from $\varnothing _1$ to $v\in {\mathcal {T}}^{\scriptscriptstyle (1)}$ is

3.10 $$\begin{aligned} T_v = \sum _{k=0}^{\left| v\right| -1} f_n(X_{p^{k}\!\left( v\right) }) \end{aligned}$$

and the FPP exploration process $\mathsf{BP}^{\scriptscriptstyle (1)}=(\mathsf{BP}^{\scriptscriptstyle (1)}_t)_{t\ge 0}$ on ${\mathcal {T}}^{\scriptscriptstyle (1)}$ is defined by $\mathsf{BP}^{\scriptscriptstyle (1)}_t=\left\{ v\in {\mathcal {T}}^{\scriptscriptstyle (1)}:T_v\le t\right\}$.

Note that the FPP edge weights $(f_n(X_{vk}))_{k\in {\mathbb {N}}}$ are themselves the points of a Poisson point process on $(0,\infty )$, independently for each $v\in {\mathcal {T}}^{\scriptscriptstyle (1)}$. The intensity measure of this Poisson point process, which we denote by $\mu _n$, is the image of Lebesgue measure on $(0,\infty )$ under $f_n$. Since $f_n$ is strictly increasing by assumption, $\mu _n$ has no atoms and we may abbreviate $\mu _n(\left( a,b\right] )$ as $\mu _n(a,b)$ for simplicity. Thus $\mu _n$ is characterized by

3.11 $$\begin{aligned} \mu _n(a,b) = f_n^{-1}(b) - f_n^{-1}(a), \quad \int _0^\infty h(y) d\mu _n(y) = \int _0^\infty h(f_n(x)) dx, \end{aligned}$$

for any measurable function $h:\left[ 0,\infty \right) \rightarrow \left[ 0,\infty \right)$.

Clearly, and as suggested by the notation, the FPP exploration process $\mathsf{BP}$ is a continuous-time branching process:

##### Proposition 3.5

(FPP on PWIT is CTBP) The process $\mathsf{BP}^{\scriptscriptstyle (1)}$ is a continuous-time branching process (CTBP), started from a single individual $\varnothing _1,$ where the ages at childbearing of an individual form a Poisson point process with intensity $\mu _n,$ independently for each individual. The time $T_v$ is the birth time $T_v=\inf \left\{ t\ge 0:v\in \mathsf{BP}^{\scriptscriptstyle (1)}_t\right\}$ of the individual $v\in {\mathcal {T}}^{\scriptscriptstyle (1)}.$

#### Coupling One-Source Exploration to the PWIT

Similar to the analysis of the weights of the edges containing vertex 1, we now introduce a thinning procedure that allows us to couple $\mathsf{BP}^{\scriptscriptstyle (1)}$ and $\mathsf{SWT}^{\scriptscriptstyle (1)}$. Define $M_{\varnothing _1}=1$ and to each other $v\in {\mathcal {T}}^{\scriptscriptstyle (1)}\setminus \left\{ \varnothing _1\right\}$ associate a mark $M_v$ chosen independently and uniformly from [*n*].

##### Definition 3.6

(*Thinning—one CTBP*) The vertex $v\in {\mathcal {T}}^{\scriptscriptstyle (1)}\setminus \left\{ \varnothing _1\right\}$ is *thinned* if it has an ancestor $v_0=p^{k}\!\left( v\right)$ (possibly *v* itself) such that $M_{v_0}=M_w$ for some unthinned vertex $w\in {\mathcal {T}}^{\scriptscriptstyle (1)}$ with $T_w<T_{v_0}$.

This definition also appears as [Part I, Definition 2.8]. As explained there, this definition is not circular since whether or not a vertex *v* is thinned can be assessed recursively in terms of earlier-born vertices. Write ${\widetilde{\mathsf{BP}}}_t^{\scriptscriptstyle (1)}$ for the subgraph of $\mathsf{BP}_t^{\scriptscriptstyle (1)}$ consisting of unthinned vertices.

##### Definition 3.7

Given a subset $\tau \subset {\mathcal {T}}^{\scriptscriptstyle (1)}$ and marks $M=(M_v :v \in \tau )$ with $M_v\in [n]$, define $\pi _M(\tau )$ to be the subgraph of $K_n$ induced by the mapping $\tau \rightarrow [n]$, $v\mapsto M_v$. That is, $\pi _M(\tau )$ has vertex set $\left\{ M_v:v\in \tau \right\}$, with an edge between $M_v$ and $M_{p\left( v\right) }$ whenever $v,p\left( v\right) \in \tau$.

Note that if the marks $(M_v)_{v\in \tau }$ are distinct then $\pi _M(\tau )$ and $\tau$ are isomorphic graphs.

The following theorem, taken from [Part I, Theorem 2.10] and proved in [Part I, Sect. 3.3], establishes a close connection between FPP on $K_n$ and FPP on the PWIT with edge weights $(f_n(X_v))_{v\in \tau }$:

##### Theorem 3.8

(Coupling to FPP on PWIT—one source) The law of $(\mathsf{SWT}_t^{\scriptscriptstyle (1)})_{t\ge 0}$ is the same as the law of $\Bigl ( \pi _M\bigl ( {\widetilde{\mathsf{BP}}}_t^{\scriptscriptstyle (1)} \bigr ) \Bigr )_{t\ge 0}$.

Theorem 3.8 is based on an explicit coupling between the edge weights $(Y_e^{\scriptscriptstyle (K_n)})_e$ on $K_n$ and $(X_v)_v$ on ${\mathcal {T}}^{\scriptscriptstyle (1)}$. We will describe a related coupling in Sect. 3.5. A general form of those couplings is given in Sect. 5.

#### Coupling Two-Source Exploration to the PWIT

Let ${\mathcal {T}}^{\scriptscriptstyle (1,2)}$ be the disjoint union of two independent copies $({\mathcal {T}}^{\scriptscriptstyle (j)},X^{\scriptscriptstyle (j)})$, $j \in \left\{ 1,2\right\}$, of the PWIT. We shall assume that the copies ${\mathcal {T}}^{\scriptscriptstyle (j)}$ are vertex-disjoint, with roots $\varnothing _j$, so that we can unambiguously write $X_v$ instead of $X^{\scriptscriptstyle (j)}_v$ for $v\in {\mathcal {T}}^{\scriptscriptstyle (j)}$, $v\ne \varnothing _j$. We set $M_{\varnothing _j}=j$ for $j=1,2$, and otherwise the notation introduced for ${\mathcal {T}}^{\scriptscriptstyle (1)}$ is used on ${\mathcal {T}}^{\scriptscriptstyle (1,2)}$, verbatim. For example, for any subset $\tau \subseteq {\mathcal {T}}^{\scriptscriptstyle (1,2)}$, we write $\partial \tau =\left\{ v \not \in \tau :p\left( v\right) \in \tau \right\}$ for the boundary vertices of $\tau$, and we define the subgraph $\pi _M(\tau )$ for $\tau \subset {\mathcal {T}}^{\scriptscriptstyle (1,2)}$ just as in Definition 3.7.

As in Proposition 3.5, the two-source FPP exploration process on ${\mathcal {T}}^{\scriptscriptstyle (1,2)}$ with edge weights $(f_n(X_v))_v$ starting from $\varnothing _1$ and $\varnothing _2$ is equivalent to the union $\mathsf{BP}=\mathsf{BP}^{\scriptscriptstyle (1)} \cup \mathsf{BP}^{\scriptscriptstyle (2)}$ of two CTBPs. (In the fluid-flow formulation, the additional rule—an edge is not explored if it would join the connected components containing the two sources—does not apply.)

##### Definition 3.9

(*Thinning—two CTBPs*) The vertex $v\in {\mathcal {T}}^{\scriptscriptstyle (1,2)}\setminus \left\{ \varnothing _1,\varnothing _2\right\}$ is *thinned* if it has an ancestor $v_0=p^{k}\!\left( v\right)$ (possibly *v* itself) such that $M_{v_0}=M_w$ for some unthinned vertex $w\in {\mathcal {T}}^{\scriptscriptstyle (1,2)}$ with $T_w<T_{v_0}$.

Note that this two-CTBP thinning rule is applied simultaneously across both trees: for instance, a vertex $v\in {\mathcal {T}}^{\scriptscriptstyle (1)}$ can be thinned due to an unthinned vertex $w\in {\mathcal {T}}^{\scriptscriptstyle (2)}$. Henceforth we will be concerned with the two-CTBP version of thinning. Write ${\widetilde{\mathsf{BP}}}_t$ for the subgraph of $\mathsf{BP}_t=\mathsf{BP}_t^{\scriptscriptstyle (1)}\cup \mathsf{BP}_t^{\scriptscriptstyle (2)}$ consisting of unthinned vertices.

The following theorem is a special case of Theorem 3.26:

##### Theorem 3.10

(Coupling to FPP on PWIT—two sources) The law of $(\mathsf{SWT}_t^{\scriptscriptstyle (1,2)})_{t\ge 0}$ is the same as the law of $\Bigl ( \pi _M\bigl ( {\widetilde{\mathsf{BP}}}_t \bigr ) \Bigr )_{t\ge 0}$.

We will not use Theorem 3.10, but instead rely on its generalization Theorem 3.26, since, in our setting, $\mathsf{BP}^{\scriptscriptstyle (1)}$ and $\mathsf{BP}^{\scriptscriptstyle (2)}$ can grow at rather different speeds. We will counteract this unbalance by an appropriate *freezing* procedure, as explained in more detail later on. Theorem 3.26 generalizes Theorem 3.10 to include this freezing.

We next state an equality in law for the collision time and collision edge. As a preliminary step, note that (3.8) can be rewritten in terms of the measure $\mu _n$ as

3.12 $$\begin{aligned} \begin{aligned}&{\mathbb {P}}\left( T_\mathrm{coll}^{\scriptscriptstyle \mathsf{SWT}}>t\left| \,(\mathsf{SWT}_u)_{u\ge 0}\right. \right) \\&\quad = \exp \Bigg ( -\sum _{\begin{array}{c} i_1\in \mathsf{SWT}_t^{\scriptscriptstyle (1,2;1)}\\ i_2\in \mathsf{SWT}_t^{\scriptscriptstyle (1,2;2)} \end{array}} \!\!\!\!\!\mu _n\left( \big \vert T^{\scriptscriptstyle \mathsf{SWT}^{\scriptscriptstyle (1,2)}}(i_1) - T^{\scriptscriptstyle \mathsf{SWT}^{\scriptscriptstyle (1,2)}}(i_2)\big \vert , t-T^{\scriptscriptstyle \mathsf{SWT}^{\scriptscriptstyle (1,2)}}(i_1) + t-T^{\scriptscriptstyle \mathsf{SWT}^{\scriptscriptstyle (1,2)}}(i_2)\right) /n \Bigg ). \end{aligned} \end{aligned}$$

By a *Cox process* with random intensity measure *Z* (with respect to a $\sigma$-algebra ${\mathscr {F}}$) we mean a random point measure ${\mathcal {P}}$ such that *Z* is ${\mathscr {F}}$-measurable and, conditionally on ${\mathscr {F}}$, ${\mathcal {P}}$ has the distribution of a Poisson point process with intensity measure *Z*. For notational convenience, given a sequence of intensity measures $Z_n$ on ${\mathbb {R}}\times {\mathcal {X}}$, for some measurable space ${\mathcal {X}}$, we write $Z_{n,t}$ for the measures on ${\mathcal {X}}$ defined by $Z_{n,t}(\cdot )=Z_n(\left( -\infty , t\right] \times \cdot )$.

Thus (3.12) states that $T_\mathrm{coll}^{\scriptscriptstyle \mathsf{SWT}}$ has the law of the first point of a Cox process on ${\mathbb {R}}$, where the intensity measure is given by a sum over $\mathsf{SWT}^{\scriptscriptstyle (1,2;1)}\times \mathsf{SWT}^{\scriptscriptstyle (1,2;2)}$ (see also [11, Proposition 2.3]). Using Theorem 3.10, we can lift this equality in law to apply to the collision time and collision edge.

##### Theorem 3.11

(Cox process for collision edge) Let ${\mathcal {P}}_n^{\scriptscriptstyle \mathsf{SWT}}$ be a Cox process on $\left[ 0,\infty \right) \times {\mathcal {T}}^{\scriptscriptstyle (1)} \times {\mathcal {T}}^{\scriptscriptstyle (2)}$ (with respect to the $\sigma$-algebra generated by $\mathsf{BP}$ and $(M_v)_{v\in {\mathcal {T}}^{\scriptscriptstyle (1,2)}})$ with random intensity $Z_n^{\scriptscriptstyle \mathsf{SWT}}=(Z_{n,t}^{\scriptscriptstyle \mathsf{SWT}})_{t\ge 0}$ defined by

3.13 $$\begin{aligned} Z_{n,t}^{\scriptscriptstyle \mathsf{SWT}}(\left\{ v_1\right\} \times \left\{ v_2\right\} ) = {\mathbb {1}}_{\left\{ v_1\in \mathsf{BP}_t^{\scriptscriptstyle (1)},v_2\in \mathsf{BP}_t^{\scriptscriptstyle (2)}\right\} } \tfrac{1}{n} \mu _n\bigl (\left| T_{v_1}-T_{v_2}\right| , t-T_{v_1}+t-T_{v_2}\bigr ) \end{aligned}$$

for all $t\ge 0$. Let $(T_\mathrm{coll}^{\scriptscriptstyle {\mathcal {P}}_n,\mathsf{SWT}},V_\mathrm{coll}^{\scriptscriptstyle (1,\mathsf{SWT})},V_\mathrm{coll}^{\scriptscriptstyle (2,\mathsf{SWT})})$ denote the first point of ${\mathcal {P}}_n^{\scriptscriptstyle \mathsf{SWT}}$ for which $V_\mathrm{coll}^{\scriptscriptstyle (1,\mathsf{SWT})}$ and $V_\mathrm{coll}^{\scriptscriptstyle (2,\mathsf{SWT})}$ are unthinned. Then the law of $(T_\mathrm{coll}^{\scriptscriptstyle {\mathcal {P}}_n,\mathsf{SWT}},\pi _M({\widetilde{\mathsf{BP}}}_{T_\mathrm{coll}^{\scriptscriptstyle {\mathcal {P}}_n,\mathsf{SWT}}}){,}M_{V_\mathrm{coll}^{\scriptscriptstyle (1{,}\mathsf{SWT})}}{,}M_{V_\mathrm{coll}^{\scriptscriptstyle (2{,}\mathsf{SWT})}})$ is the same as the joint law of $T_\mathrm{coll}^{\scriptscriptstyle \mathsf{SWT}}=\tfrac{1}{2}W_n;$ the smallest-weight tree $\mathsf{SWT}_{T_\mathrm{coll}^{\scriptscriptstyle \mathsf{SWT}}}$ at the time $T_\mathrm{coll}^{\scriptscriptstyle \mathsf{SWT}};$ and the endpoints $I_1^{\scriptscriptstyle \mathsf{SWT}},I_2^{\scriptscriptstyle \mathsf{SWT}}$ of the SWT collision edge. In particular, the hopcount $H_n$ has the same distribution as $\left| V_\mathrm{coll}^{\scriptscriptstyle (1,\mathsf{SWT})}\right| +\left| V_\mathrm{coll}^{\scriptscriptstyle (2,\mathsf{SWT})}\right| +1$.

### FPP on the PWIT as a CTBP

In this section, we relate FPP on the PWIT to a continuous-time branching process (CTBP). In Sect. 3.3.1, we investigate the exploration from one vertex and describe this in terms of one-vertex characteristics. In Sect. 3.3.2, we extend this to the exploration from two vertices and relate this to two-vertex characteristics of CTBPs, which will be crucial to analyse shortest-weight paths in FPP on $K_n$ which we explore from two sources.

#### FPP on the PWIT as a CTBP: One-Vertex Characteristics

In this section, we analyze the CTBP $\mathsf{BP}^{\scriptscriptstyle (1)}$ introduced in Sect. 3.2. Notice that $(\mathsf{BP}_t^{\scriptscriptstyle (1)})_{t\ge 0}$ depends on *n* through its offspring distribution. We have to understand the coupled double asymptotics of *n* and *t* tending to infinity simultaneously.

Recall that we write $\left| v\right|$ for the generation of *v* (i.e., its graph distance from the root in the genealogical tree). To count particles in $\mathsf{BP}_t^{\scriptscriptstyle (1)}$, we use a non-random characteristic $\chi :\left[ 0,\infty \right) \rightarrow \left[ 0,\infty \right)$. Following [11], define the *generation-weighted vertex characteristic* by

3.14 $$\begin{aligned} z_t^\chi (a)=z_t^{\chi , \mathsf{BP}^{\scriptscriptstyle (1)}}(a) = \sum _{v\in \mathsf{BP}_t^{\scriptscriptstyle (1)}} a^{|v|} \chi (t-T_v) \quad \text {for all }a,t\ge 0. \end{aligned}$$

We make the convention that $\chi (t)=z_t^\chi (a)=0$ for $t<0$. For characteristics $\chi$, $\eta$ and for $a,b,t,u\ge 0$, write

3.15 $$\begin{aligned} m_t^\chi (a)={\mathbb {E}}(z_t^\chi (a)) \quad \text {and} \quad M_{t,u}^{\chi ,\eta }(a,b)={\mathbb {E}}(z_t^\chi (a) z_u^\eta (b)). \end{aligned}$$

Let ${\hat{\mu }}_n(\lambda )=\int {\mathrm e}^{-\lambda y}d\mu _n(y)$ denote the Laplace transform of $\mu _n$. For $a>0$, define $\lambda _n(a)>0$ by

3.16 $$\begin{aligned} a{\hat{\mu }}_n(\lambda _n(a))=1 \end{aligned}$$

whenever (3.16) has a unique solution. The parameters $\lambda _n$ and $\phi _n$ in Theorem 2.4 are given by

3.17 $$\begin{aligned} \lambda _n&=\lambda _n(1), \end{aligned}$$

3.18 $$\begin{aligned} \phi _n&= \lambda _n'(1)/\lambda _n(1). \end{aligned}$$

The asymptotics of $\lambda _n$ and $\phi _n$ stated in Theorem 2.4 is the content of the following lemma:

##### Lemma 3.12

(Asymptotics of BP-parameters) As $$n\rightarrow \infty ,$$$$\phi _n/s_n \rightarrow 1$$ and $$\lambda _n f_n(1) \rightarrow {\mathrm e}^{-\gamma },$$ where $$\gamma $$ is Euler’s constant.

Lemma 3.12 is proved in Sect. 6.3.

Typically, $z_t^\chi (a)$ grows exponentially in *t* at rate $\lambda _n(a)$. Therefore, we write

3.19 $$\begin{aligned}&{\bar{z}}_t^\chi (a)= {\mathrm e}^{-\lambda _n(a) t} z_t^\chi (a), \nonumber \\&{\bar{m}}_t^\chi (a)= {\mathbb {E}}({\bar{z}}_t^\chi (a)) = {\mathrm e}^{-\lambda _n(a) t} m_t^\chi (a), \nonumber \\&{\bar{M}}_{t,u}^{\chi ,\eta }(a,b)= {\mathbb {E}}({\bar{z}}_t^\chi (a) {\bar{z}}_u^\eta (b)) = {\mathrm e}^{-\lambda _n(a)t}{\mathrm e}^{-\lambda _n(b)u} M_{t,u}^{\chi ,\eta }(a,b). \end{aligned}$$

In the following theorem, we investigate the asymptotics of such generation-weighted one-vertex characteristics:

##### Theorem 3.13

(Asymptotics of one-vertex characteristics) Given $\varepsilon >0$ and a compact subset $A\subset (0,2),$ there is a constant $K<\infty$ such that for *n* sufficiently large, uniformly for $a,b\in A$ and for $\chi$ and $\eta$ bounded, non-negative, non-decreasing functions, $\lambda _n(1)[t\wedge u] \ge K,$

3.20 $$\begin{aligned}&\left| s_n^{-1} {\bar{m}}_t^\chi (a^{1/s_n}) - \textstyle {\int _0^\infty } {\mathrm e}^{-z}\chi \bigl (z/\lambda _n(a^{1/s_n})\bigr ) dz\right| \le \varepsilon \left\| \chi \right\| _\infty , \end{aligned}$$

3.21 $$\begin{aligned}&\left| s_n^{-3} {\bar{M}}_{t,u}^{\chi ,\eta }(a^{1/s_n},b^{1/s_n}) - \frac{\textstyle {\int _0^\infty } {\mathrm e}^{-z} \chi \bigl ( z/\lambda _n(a^{1/s_n}) \bigr )dz \, \textstyle {\int _0^\infty } {\mathrm e}^{-w}\eta \bigl ( w/\lambda _n(b^{1/s_n}) \bigr )dw}{\log (1/a+1/b)}\right| \nonumber \\&\quad \qquad \qquad \qquad \qquad \qquad \qquad \le \varepsilon \left\| \chi \right\| _\infty \left\| \eta \right\| _\infty . \end{aligned}$$

Moreover, there is a constant $K'<\infty$ independent of $\varepsilon$ such that ${\bar{m}}_t^\chi (a^{1/s_n}) \le K' \left\| \chi \right\| _\infty s_n$ and ${\bar{M}}_{t,u}^{\chi ,\eta }(a^{1/s_n},b^{1/s_n}) \le K' \left\| \chi \right\| _\infty \left\| \eta \right\| _\infty s_n^3$ for all *n* sufficiently large, uniformly over $u,t\ge 0$ and $a,b\in A$.

##### Corollary 3.14

(Asymptotics of means and variance of population size) The population size $$\left| \mathsf{BP}^{\scriptscriptstyle (1)}_t\right| $$ satisfies $${\mathbb {E}}(\left| \mathsf{BP}^{\scriptscriptstyle (1)}_t\right| )\sim s_n {\mathrm e}^{\lambda _n(1)t}$$ and $${{\,\mathrm{Var}\,}}(\left| \mathsf{BP}^{\scriptscriptstyle (1)}_t\right| )\sim s_n^3 {\mathrm e}^{2\lambda _n(1)t}/\log 2$$ in the limit as $$\lambda _n(1)t\rightarrow \infty ,$$$$n\rightarrow \infty $$.

Theorem 3.13 is proved in Sect. 6.4. Generally, we will be interested in characteristics $\chi =\chi _n$ for which $\chi _n\left( \lambda _n(1)^{-1}\cdot \right)$ converges as $n\rightarrow \infty$, so that the integral in (3.20) acts as a limiting value. In particular, Corollary 3.14 is the special case $\chi ={\mathbb {1}}_{\left[ 0,\infty \right) }$, $a=1$.

Since $s_n\rightarrow \infty$, Theorem 3.13 and Corollary 3.14 show that the variance of ${\bar{z}}^\chi _t(a^{1/s_n})$ is larger compared to the square of the mean, by a factor of order $s_n$. This suggests that $\mathsf{BP}^{\scriptscriptstyle (1)}_t$ is typically of order 1 when $\lambda _n(1)t$ is of order 1 [i.e., when *t* is of order $f_n(1)$, see Lemma 3.12] but has probability of order $1/s_n$ of being of size of order $s_n^2$. See also [Part I, Proposition 2.17] which confirms this behavior.

#### FPP on the PWIT as a CTBP: Two-Vertex Characteristics

Theorem 3.11 expresses the collision time $T_\mathrm{coll}^{\scriptscriptstyle \mathsf{SWT}}$ as the first point of a Cox process whose cumulative intensity is given by a double sum over two branching processes. To study such intensities, we introduce *generation-weighted two-vertex characteristics*. Let $\chi$ be a non-random, non-negative function on $\left[ 0,\infty \right) ^2$ and recall that $T_v=\inf \left\{ t \ge 0:v \in \mathsf{BP}_t\right\}$ denotes the birth time of vertex *v* and $\left| v\right|$ the generation of *v*. The generation-weighted two-vertex characteristic is given by

3.22 $$\begin{aligned} z_{\vec {t}}^\chi (\vec {a}) = \sum _{v_1\in \mathsf{BP}_{t_1}^{\scriptscriptstyle (1)}} \sum _{v_2\in \mathsf{BP}_{t_2}^{\scriptscriptstyle (2)}} a_1^{\left| v_1\right| } a_2^{\left| v_2\right| } \chi (t_1-T_{v_1},t_2-T_{v_2}), \end{aligned}$$

for all $t_1,t_2,a_1,a_2 \ge 0$, where we use vector notation $\vec {a}=(a_1,a_2)$, $\vec {t}=(t_1,t_2)$, and so on. We make the convention that $\chi (t_1,t_2)=z_{t_1,t_2}^\chi (\vec {a})=0$ for $t_1\wedge t_2<0$. As in (3.19), we rescale and write

3.23 $$\begin{aligned} \begin{aligned}&{\bar{z}}_{\vec {t}}^\chi (\vec {a}) = {\mathrm e}^{-\lambda _n(a_1) t_1} {\mathrm e}^{-\lambda _n(a_2) t_2} z_{t_1,t_2}^\chi (\vec {a}), \\&{\bar{m}}_{\vec {t}}^\chi (\vec {a}) = {\mathbb {E}}({\bar{z}}_{\vec {t}}^\chi (\vec {a})), \\&{\bar{M}}_{\vec {t},\vec {u}}^{\chi ,\eta }(\vec {a},\vec {b}) = {\mathbb {E}}({\bar{z}}_{\vec {t}}^\chi (\vec {a}) {\bar{z}}_{\vec {u}}^\eta (\vec {b})). \end{aligned} \end{aligned}$$

In (3.13), the cumulative intensity $Z_{n,t}^{\scriptscriptstyle \mathsf{SWT}}$ can be expressed in terms of a two-vertex characteristic. If we define

3.24 $$\begin{aligned} \chi _n(t_1,t_2)=\mu _n(\left| t_1-t_2\right| ,t_1+t_2) \end{aligned}$$

then the total cumulative intensity is given by

3.25 $$\begin{aligned} \left| Z_{n,t}^{\scriptscriptstyle \mathsf{SWT}}\right| =Z_{n,t}^{\scriptscriptstyle \mathsf{SWT}}({\mathcal {T}}^{\scriptscriptstyle (1)}\times {\mathcal {T}}^{\scriptscriptstyle (2)}) = \frac{1}{n} z_{t,t}^{\chi _n}(1,1). \end{aligned}$$

We will use the parameters $a_1,a_2$ to compute moment generating functions corresponding to $Z_{n,t}^{\scriptscriptstyle \mathsf{SWT}}$.

The characteristic $\chi _n$ will prove difficult to control directly, because its values fluctuate significantly in size: for instance, $\chi _n\left( \tfrac{1}{2}f_n(1),\tfrac{1}{2}f_n(1)\right) =1$ whereas $\chi _n\left( \tfrac{1}{2}f_n(1),f_n(1)\right) =O(1/s_n)$. Therefore, for $K\in (0,\infty )$, we define the truncated measure

3.26 $$\begin{aligned} \mu _n^{\scriptscriptstyle (K)}=\mu _n\big \vert _{\left( f_n(1-K/s_n),f_n(1+K/s_n)\right] }, \end{aligned}$$

and again write $\mu _n^{\scriptscriptstyle (K)}(\left( a,b\right] )=\mu _n^{\scriptscriptstyle (K)}(a,b)$ to shorten notation. For convenience, we will always assume that *n* is large enough that $s_n\ge K$. By analogy with (3.24), define

3.27 $$\begin{aligned} \chi _n^{\scriptscriptstyle (K)}(t_1,t_2)=\mu _n^{\scriptscriptstyle (K)}(\left| t_1-t_2\right| ,t_1+t_2). \end{aligned}$$

By construction, the total mass of $\mu _n^{\scriptscriptstyle (K)}$ is $2K/s_n$, so that $s_n \chi _n^{\scriptscriptstyle (K)}$ is uniformly bounded.

The following results identify the asymptotic behavior of ${\bar{z}}_{t_1,t_2}^{\chi ^{\scriptscriptstyle (K)}_n}(\vec {a})$ and show that, for $K\rightarrow \infty$, the contribution due to $\chi _n-\chi ^{\scriptscriptstyle (K)}_n$ becomes negligible. These results are formulated in Theorem 3.15, which investigates the truncated two-vertex characteristic, and Theorem 3.16, which studies the effect of truncation:

##### Theorem 3.15

(Convergence of truncated two-vertex characteristic) For every $$\varepsilon >0$$ and every compact subset $$A\subset (0,2),$$ there exists a constant $$K_0<\infty $$ such that for every $$K \ge K_0$$ there are constants $$K'<\infty $$ and $$n_0 \in {\mathbb {N}}$$ such that for all $$n \ge n_0,$$$$a_1,a_2,b_1,b_2\in A$$ and $$\lambda _n(1)[t_1\wedge t_2\wedge u_1\wedge u_2] \ge K',$$

3.28 $$\begin{aligned} \left| s_n^{-1} {\bar{m}}_{\vec {t}}^{\chi _n^{\scriptscriptstyle (K)}}(\vec {a}^{1/s_n}) - \zeta (a_2/a_1)\right| \le \varepsilon , \end{aligned}$$

and

3.29 $$\begin{aligned} \left| s_n^{-4} {\bar{M}}_{\vec {t},\vec {u}}^{\chi _n^{\scriptscriptstyle (K)},\chi _n^{\scriptscriptstyle (K)}} (\vec {a}^{1/s_n},\vec {b}^{1/s_n}) - \frac{\zeta (a_2/a_1)\zeta (b_2/b_1)}{\log (1/a_1+1/b_1)\log (1/a_2+1/b_2)}\right| \le \varepsilon , \end{aligned}$$

where $\zeta :(0,\infty )\rightarrow {\mathbb {R}}$ is the continuous function defined by

3.30 $$\begin{aligned} \zeta (a_1/a_2)= {\left\{ \begin{array}{ll} \frac{2a_1 a_2}{a_1+a_2}\frac{\log (a_2/a_1)}{a_2-a_1} , &{} \text {if } a_1\ne a_2,\\ 1, &{} \text {if }a_1=a_2. \end{array}\right. } \end{aligned}$$

Moreover, for every $$K<\infty $$ there are constants $$K''<\infty $$ and $$n_0'\in {\mathbb {N}}$$ such that for all $$n \ge n_0',$$$$t_1,t_2,u_1,u_2\ge 0$$ and $$a_1,a_2,b_1,b_2\in A,$$$${\bar{m}}_{\vec {t}}^{\chi _n^{\scriptscriptstyle (K)}}(\vec {a}^{1/s_n}) \le K'' s_n$$ and $${\bar{M}}_{\vec {t},\vec {u}}^{\chi _n^{\scriptscriptstyle (K)},\chi _n^{\scriptscriptstyle (K)}}(\vec {a}^{1/s_n},\vec {b}^{1/s_n})\le K'' s_n^4.$$

The exponents in Theorem 3.15 can be understood as follows. By Theorem 3.13, the first and second moments of a bounded one-vertex characteristic are of order $s_n$ and $s_n^3$, respectively. Therefore, for two-vertex characteristics, one can expect $s_n^2$ and $s_n^6$. Since $\chi _n^{\scriptscriptstyle (K)}=\frac{1}{s_n}s_n\chi _n^{\scriptscriptstyle (K)}$ appears once in the first and twice in the second moment, we arrive at $s_n$ and $s_n^4$, respectively.

##### Theorem 3.16

(The effect of truncation) For every $$K>0,$$$${\bar{m}}_{\vec {t}}^{\chi _n-\chi _n^{(K)}}(\vec {1})=O(s_n),$$ uniformly over $$t_1,t_2$$. Furthermore, given $$\varepsilon >0,$$ there exists $$K<\infty $$ such that, for all *n* sufficiently large, $${\bar{m}}_{\vec {t}}^{\chi _n-\chi _n^{\scriptscriptstyle (K)}}(\vec {1})\le \varepsilon s_n$$ whenever $$\lambda _n(1)[t_1\wedge t_2]\ge K.$$

Theorems 3.15 and 3.16 are proved in Sect. 7.

### CTBP Growth and the Need for Freezing: Medium Time Scales

Theorem 3.11 shows how to analyze the weight $W_n$ and hopcount $H_n$ in terms of a Cox process driven by two (*n*-dependent) branching processes. In this section, we describe how this analysis works when the branching processes grow normally (i.e., exponentially with a fixed prefactor). In the CTBP scaling results from Sect. 3.3, we have seen that the class of edge weights we consider gives rise to a more complicated scaling, with *n*-dependent prefactors that diverge to infinity. As we will explain, this causes a direct analysis to break down, and we define an appropriate *freezing* mechanism that we use to overcome this obstacle. In Sect. 3.4.1, we first explain what we mean with freezing and why we need it, and in Sect. 3.4.2 we explain how FPP from two sources can be frozen, and then later unfrozen, such that CTBP asymptotics can be used and collision times between the two FPP clusters can be analyzed.

#### Frozen FPP Exploration Process

Under reasonable hypotheses, a fixed CTBP grows exponentially under all measures of size. More precisely, there will be a single constant $\lambda$ such that $m^\eta _t(1)\sim A_\eta {\mathrm e}^{\lambda t}$ and $M^{\eta ,\eta }_{t,t}(1,1)\sim B_\eta {\mathrm e}^{2\lambda t}$ for constants $A_\eta ,B_\eta$, over a wide class of one-vertex characteristics $\eta :\left[ 0,\infty \right) \rightarrow {\mathbb {R}}$, including the choice $\eta =1$ that encodes the population size. Similarly, if $\chi$ is a two-vertex characteristic, we can expect that $m^\chi _{t,t}(\vec {1})\sim C_\chi {\mathrm e}^{2\lambda t}$ and $M^\chi _{(t,t),(t,t)}\sim D_\chi {\mathrm e}^{4\lambda t}$. Taking $\left| Z_{n,t}\right| =\tfrac{1}{n}z^\chi _{t,t}(1)$ as in (3.25), we would then expect the first point of the Cox process from Theorem 3.11 to appear at times *t* for which ${\mathrm e}^{2\lambda t}\approx n$. For such *t*, we have ${\mathrm e}^{\lambda t}\approx \sqrt{n}$, so that each branching process has of order $\sqrt{n}$ individuals and a typical individual is of order $\log n$ generations away from the root.

If these asymptotics hold, then a typical vertex *v* alive at such times *t* is unthinned whp. Indeed, for each of the $\approx \log n$ ancestors, there are at most $\approx \sqrt{n}$ other vertices that might have the same mark. Each pair of vertices has probability 1/*n* of having the same mark, leading to an upper bound of $\approx \frac{\sqrt{n}\log n}{n}$ on the probability that *v* is thinned.

In particular, the first point of ${\mathcal {P}}^{\scriptscriptstyle \mathsf{SWT}}_n$ coincides whp with $(T_\mathrm{coll}^{\scriptscriptstyle ({\mathcal {P}}_n,\mathsf{SWT})},V_\mathrm{coll}^{\scriptscriptstyle (1,\mathsf{SWT})},V_\mathrm{coll}^{\scriptscriptstyle (2,\mathsf{SWT})})$, the first unthinned point of ${\mathcal {P}}^{\scriptscriptstyle \mathsf{SWT}}_n$. Using Theorem 3.11, it is therefore possible to derive asymptotics of $W_n$ and $H_n$ by analysing the first point of ${\mathcal {P}}^{\scriptscriptstyle \mathsf{SWT}}_n$, which in turn can be done by a first- and second-moment analysis of the intensity measure $Z_{n,t}^{\scriptscriptstyle \mathsf{SWT}}$.

In the setting of this paper, however, this analysis breaks down. The branching processes now themselves depend on *n*, and their behaviour becomes irregular when *n* is large. One-vertex characteristics satisfy $m^\eta _t(1) \sim A'_\eta s_n {\mathrm e}^{\lambda _n(1) t}$ and $M^{\eta ,\eta }_{t,t}(1,1) \sim B'_\eta s_n^3 {\mathrm e}^{2\lambda _n(1) t}$. The mismatch of prefactors, $s_n$ versus $s_n^3$, suggests that the branching process has probability of order $1/s_n$ of growing to size $s_n^2$ in a time of order $1/\lambda _n(1)\approx f_n(1)$, and that this unlikely event is important to the long-run growth of the branching process.

We can balance the mismatched first and second moments by aggregating $s_n$ independent copies of the branching process. The sum of $s_n$ independent copies of $z^\eta _t(1)$ will have mean of order $A'_\eta s_n^2 {\mathrm e}^{\lambda _n(1)t}$ and second moment of order $B''_\eta s_n^4 {\mathrm e}^{2\lambda _n(1)t}$, where now the second moment is on the order of the square of the mean. [With proper attention to correlations, it is also possible to show that the two-vertex characteristics $z^{\chi _n}_{t,t}(1)$, summed over two groups of $s_n$ independent branching processes each, will have mean of order $C' s_n^3 {\mathrm e}^{2\lambda _n(1)t}$ and second moment $D' s_n^6 {\mathrm e}^{4\lambda _n(1)t}$.] This balancing makes a first- and second-moment analysis possible.

To achieve the same effect starting from two branching processes, wait until each branching process is large enough that it has of order $s_n$ new children in time of order 1. Then the collection of all individuals born after that time (and their descendants) will again have balanced first and second moments. However, as we will see, the time when each branching process becomes large enough is highly variable. In particular, by the time the slower-growing of the two branching processes is large enough, the faster-growing branching process will have become much too large. For this reason, we will need to *freeze* the faster-growing branching process to allow the other to catch up. In the following sections we explain how freezing affects the FPP exploration process, the coupling to the PWIT, the Cox process representation for the optimal path, and the effect of thinning. We now first explain precisely how we freeze our two branching processes.

The choice of the freezing times $T_\mathrm{fr}^{\scriptscriptstyle (j)}$ must attain two goals. First, we must ensure that, at the collision time $T_\mathrm{coll}$, the two branching processes with freezing are of comparable size (see Theorem 3.33 and the discussion following it). Second, we must ensure that, after the freezing times, the branching processes grow predictably, with the relatively steady exponential growth typical of supercritical branching processes (in spite of Theorem 3.13 and Corollary 3.14, where the mismatch between mean and variance shows that a branching process from a single initial individual has highly variable growth).

It has been argued in [Part I, Sect. 2.5] that the crossover to typical branching process behavior occurs when we begin to discover “lucky” vertices that have a large number of descendants—of order $s_n^2$—in a time of order $f_n(1)$. From [Part I, Theorem 2.15], we can see that this crossover coincides approximately with several other milestones: for instance, around the same time, the branching process also reaches height of order $s_n$ and total size of order $s_n^2$. For our purposes, it will be most important to control *moments* of the branching process after unfreezing, which will involve exponentially discounting future births at rate $\lambda _n(1)$.

These considerations lead to the following definition of the freezing times:

##### Definition 3.17

(*Freezing*) Define, for $j=1,2$, the *freezing times*

3.31 $$\begin{aligned} T_\mathrm{fr}^{\scriptscriptstyle (j)} = \inf \bigg \{ t\ge 0:\sum _{v\in \mathsf{BP}_t^{\scriptscriptstyle (j)}} \int _{t-T_v}^\infty {\mathrm e}^{-\lambda _n(1) \left( y-(t-T_v)\right) } d\mu _n(y) \ge s_n \bigg \}, \end{aligned}$$

and the *unfreezing time* $T_\mathrm{unfr}=T_\mathrm{fr}^{\scriptscriptstyle (1)} \vee T_\mathrm{fr}^{\scriptscriptstyle (2)}$. The *frozen cluster* is given by

3.32 $$\begin{aligned} {\mathcal {B}}_\mathrm{fr}=\mathsf{BP}_{T_\mathrm{unfr}}={\mathcal {B}}_\mathrm{fr}^{\scriptscriptstyle (1)}\cup {\mathcal {B}}_\mathrm{fr}^{\scriptscriptstyle (2)}, \quad \text {where} \quad {\mathcal {B}}_\mathrm{fr}^{\scriptscriptstyle (j)}=\mathsf{BP}_{T_\mathrm{fr}^{(j)}}^{\scriptscriptstyle (j)}. \end{aligned}$$

The variable $\int _{t-T_v}^\infty {\mathrm e}^{-\lambda _n(1) \left( y-(t-T_v)\right) } d\mu _n(y)$ represents the expected number of future offspring of vertex $v\in \mathsf{BP}_t^{\scriptscriptstyle (j)}$, exponentially time-discounted at rate $\lambda _n(1)$. In Definition 3.17, this expected discounted number (summed over all $v\in \mathsf{BP}_t^{\scriptscriptstyle (j)}$) is required to exceed $s_n$. This is the correct choice of scaling, because each newly born vertex has probability of order $1/s_n$ of being “lucky”—i.e., having of order $s_n^2$ descendants in time $f_n(1)$, see [Part I, Definition 2.14 and Proposition 2.17]—and the scaling $s_n$ in Definition 3.17 ensures that such a lucky vertex will be born in time $O_{\scriptscriptstyle {{\mathbb {P}}}}(f_n(1))$ after unfreezing.

Recall that $M^{\scriptscriptstyle (1)}, M^{\scriptscriptstyle (2)}$ are i.i.d. random variables for which ${{\mathbb {P}}}(M^{\scriptscriptstyle (j)}\le x)$ is the survival probability of a Poisson Galton–Watson branching process with mean *x*. The asymptotics of the freezing times $T_\mathrm{fr}^{\scriptscriptstyle (j)}$ and the frozen cluster ${\mathcal {B}}_\mathrm{fr}$ are as follows.

##### Theorem 3.18

(Properties of the freezing times and frozen cluster) 

- The freezing times satisfy $f_n^{-1}(T_\mathrm{fr}^{\scriptscriptstyle (j)}) {\mathop {\longrightarrow }\limits ^{\scriptscriptstyle {{\mathbb {P}}}}}M^{\scriptscriptstyle (j)}$ for $j=1,2.$
- The volume $\left| {\mathcal {B}}_\mathrm{fr}\right|$ of the frozen cluster is $O_{\mathbb {P}}(s_n^2)$.
- The maximum height $\max \left\{ \left| v\right| :v\in {\mathcal {B}}_\mathrm{fr}\right\}$ of the frozen cluster is $O_{\mathbb {P}}(s_n)$.

We expect, but do not prove, that the bounds in parts (b) and (c) are of the correct order, i.e., that the volume is $\Theta _{\scriptscriptstyle {{\mathbb {P}}}}(s_n^2)$ and the diameter $\Theta _{\scriptscriptstyle {{\mathbb {P}}}}(s_n)$. The proof of Theorem 3.18 is based on [Part I, Theorem 2.15] and is given in Sect. 9.5.

Since $M^{\scriptscriptstyle (1)}\ne M^{\scriptscriptstyle (2)}$ a.s., Theorem 3.18 (a) and the scaling properties of $f_n$ confirm that the two CTBPs $\mathsf{BP}^{\scriptscriptstyle (1)}$ and $\mathsf{BP}^{\scriptscriptstyle (2)}$ require substantially different times to grow large enough. Theorem 3.18 (b) and (c) will allow us to ignore the elements coming from the frozen cluster in the proof of Theorem 2.4. For instance, part (c) shows that heights within the frozen cluster are negligible in the central limit theorem scaling of (2.10).

From the proof of Theorem 2.4, we will see that

3.33 $$\begin{aligned} W_n - \frac{1}{\lambda _n}\log {(n/s_n^3)} = T_{\mathrm{fr}}^{\scriptscriptstyle (1)}+T_{\mathrm{fr}}^{\scriptscriptstyle (2)} + O_{\scriptscriptstyle {{\mathbb {P}}}}(f_n(1)). \end{aligned}$$

[The presence of a logarithm in (3.33) reflects the fact that, after $T_\mathrm{unfr}$, the branching processes grow exponentially.] The effects of the three terms in (3.33) can be combined using the following lemma:

##### Lemma 3.19

(Sums behave like maxima) Let $$(T_n^{\scriptscriptstyle (1)})_n,$$$$(T_n^{\scriptscriptstyle (2)})_n,$$$${\mathcal {M}}^{\scriptscriptstyle (1)},$$$${\mathcal {M}}^{\scriptscriptstyle (2)}$$ be random variables such that $${\mathcal {M}}^{\scriptscriptstyle (1)} \vee {\mathcal {M}}^{\scriptscriptstyle (2)} \ge 1$$ a.s. and $$(f_n^{-1}(T_n^{\scriptscriptstyle (1)}),f_n^{-1}(T_n^{\scriptscriptstyle (2)})) {\mathop {\longrightarrow }\limits ^{\scriptscriptstyle {{\mathbb {P}}}}}({\mathcal {M}}^{\scriptscriptstyle (1)},{\mathcal {M}}^{\scriptscriptstyle (2)}).$$ Then $$f_n^{-1}(T_n^{\scriptscriptstyle (1)}+T_n^{\scriptscriptstyle (2)}) {\mathop {\longrightarrow }\limits ^{\scriptscriptstyle {{\mathbb {P}}}}}{\mathcal {M}}^{\scriptscriptstyle (1)} \vee {\mathcal {M}}^{\scriptscriptstyle (2)}$$.

Lemma 3.19 is proved in Sect. 4. Theorem 3.18 (a) and Lemma 3.19 yield that $f_n^{-1}(T_{\mathrm{fr}}^{\scriptscriptstyle (1)}+T_{\mathrm{fr}}^{\scriptscriptstyle (2)}){\mathop {\longrightarrow }\limits ^{\scriptscriptstyle {{\mathbb {P}}}}}M^{\scriptscriptstyle (1)}\vee M^{\scriptscriptstyle (2)}$. Because $M^{\scriptscriptstyle (1)}, M^{\scriptscriptstyle (2)}>1$ a.s., we will be able to ignore the term $O_{\scriptscriptstyle {{\mathbb {P}}}}(f_n(1)),$ and the scaling of $W_n$ in Theorem 2.4 will follow.

#### FPP Exploration Process from Two Sources with Freezing and Collisions

Let $T_\mathrm{fr}^{\scriptscriptstyle (j)}$ be the freezing times defined in Definition 3.17, which are stopping times with respect to the filtration induced by $\mathsf{BP}^{\scriptscriptstyle (j)}_t$, $j \in \left\{ 1,2\right\}$. Define

3.34 $$\begin{aligned} T_\mathrm{unfr}&=T_\mathrm{fr}^{\scriptscriptstyle (1)}\vee T_\mathrm{fr}^{\scriptscriptstyle (2)}, \end{aligned}$$

3.35 $$\begin{aligned} R_j(t)&=(t\wedge T_\mathrm{fr}^{\scriptscriptstyle (j)}) + ((t-T_\mathrm{unfr})\vee 0), \end{aligned}$$

and

3.36 $$\begin{aligned} {\mathcal {B}}_t=\bigcup _{j=1}^2 {\mathcal {B}}_t^{\scriptscriptstyle (j)}, \quad {\mathcal {B}}_t^{\scriptscriptstyle (j)}=\left\{ v\in {\mathcal {T}}^{\scriptscriptstyle (j)} :T_v \le R_j(t)\right\} = \mathsf{BP}_{R_j(t)}^{\scriptscriptstyle (j)} \quad \text {for all }t\ge 0. \end{aligned}$$

In words, we run the two branching processes $\mathsf{BP}=\mathsf{BP}^{\scriptscriptstyle (1)}\cup \mathsf{BP}^{\scriptscriptstyle (2)}$ normally until the first freezing time, $T_\mathrm{fr}^{\scriptscriptstyle (1)}\wedge T_\mathrm{fr}^{\scriptscriptstyle (2)}$, when one of the two branching processes has become large enough. Then we freeze the larger CTBP and allow the smaller one to evolve normally until it is large enough, at time $T_\mathrm{unfr}=T_\mathrm{fr}^{\scriptscriptstyle (1)}\vee T_\mathrm{fr}^{\scriptscriptstyle (2)}$. At this time, which we call the *unfreezing* time $T_\mathrm{unfr}=T_\mathrm{fr}^{\scriptscriptstyle (1)}\vee T_\mathrm{fr}^{\scriptscriptstyle (2)}$, both CTBPs resume their usual evolution. The processes $R_j(t)$ are the on-off processes that encode this behaviour: $R_j(t)$ increases at constant rate 1, except for the interval between $T_\mathrm{fr}^{\scriptscriptstyle (1)}\wedge T_\mathrm{fr}^{\scriptscriptstyle (2)}$ and $T_\mathrm{unfr}$, where one of the two processes is constant. In the fluid flow picture, $R_j(t)$ represents the distance traveled by fluid from vertex $j\in \left\{ 1,2\right\}$. We call the process $({\mathcal {B}}_t)_{t\ge 0}$ the *two-source branching process with freezing*.

As with ${\mathcal {T}}^{\scriptscriptstyle (1,2)}$, we can consider ${\mathcal {B}}_t$ to be the union of two trees by placing an edge between each non-root vertex $v\notin \left\{ \varnothing _1,\varnothing _2\right\}$ and its parent. We denote by $T_v^{\mathcal {B}}=\inf \left\{ t\ge 0 :v\in {\mathcal {B}}_t\right\}$ the arrival time of the individual $v\in {\mathcal {T}}^{\scriptscriptstyle (1,2)}$ in ${\mathcal {B}}=({\mathcal {B}}_t)_{t\ge 0}$. Using the left-continuous inverse of $R_j(t)$, defined by

3.37 $$\begin{aligned} R_j^{-1}(y) = \inf \left\{ t\ge 0:R_j(t)\ge y\right\} = {\left\{ \begin{array}{ll} t &{} \text {if }t\le T_\mathrm{fr}^{\scriptscriptstyle (j)},\\ T_\mathrm{unfr}-T_\mathrm{fr}^{\scriptscriptstyle (j)}+t &{} \text {if }t>T_\mathrm{fr}^{\scriptscriptstyle (j)}, \end{array}\right. } \end{aligned}$$

we obtain

3.38 $$\begin{aligned} T_v^{\mathcal {B}}=R_j^{-1}(T_v)\quad \text {for }v\in {\mathcal {T}}^{\scriptscriptstyle (j)}. \end{aligned}$$

We next define the *two-source FPP exploration process with freezing* on $K_n$, which we will denote $({\mathcal {S}}_t)_{t\ge 0}$. Intuitively, ${\mathcal {S}}_t$ is the analogue of $\mathsf{SWT}^{\scriptscriptstyle (1,2)}_t$ under the assumption that fluid from vertex *j* has flowed a distance $R_j(t)$ by time *t*. As with $\mathsf{SWT}^{\scriptscriptstyle (1,2)}$, fluid from one vertex blocks fluid from the other vertex, so that ${\mathcal {S}}_t$ will consist of two vertex-disjoint trees for all *t*. However, because fluid from one vertex may be frozen while still blocking fluid from the other vertex, it will no longer be possible to directly specify the vertex set $V({\mathcal {S}}_t)$ as in (3.4). Instead we will define ${\mathcal {S}}=({\mathcal {S}}_t)_{t\ge 0}$ inductively using $R_j^{-1}$ such that at every time $t\ge 0$, ${\mathcal {S}}_t={\mathcal {S}}_t^{\scriptscriptstyle (1)}\cup {\mathcal {S}}_t^{\scriptscriptstyle (2)}$ is the disjoint union of two trees ${\mathcal {S}}_t^{\scriptscriptstyle (1)}$ and ${\mathcal {S}}_t^{\scriptscriptstyle (2)}$ with root 1 and 2, respectively.

At time $t=0$, let ${\mathcal {S}}_0$ be the subgraph of $K_n$ with vertex set $\left\{ 1,2\right\}$ and no edges. Suppose inductively that we have constructed $({\mathcal {S}}_t)_{0\le t\le \tau _{k-1}}$ up to the time $\tau _{k-1}$ where the $(k-1)$st vertex (not including the vertices 1 and 2) was added, for $1\le k\le n-2$, with the convention that $\tau _0=0$. Denote by $T^{\scriptscriptstyle {\mathcal {S}}}(i)=\inf \left\{ t\ge 0:i\in {\mathcal {S}}_t\right\}$ the arrival time of a vertex $i\in [n]$.

Consider the set $\partial {\mathcal {S}}_{\tau _{k-1}}$ of edges *e* joining a previously explored vertex ${\underline{e}}\in {\mathcal {S}}_{\tau _{k-1}}$ to a new vertex ${\overline{e}}\notin {\mathcal {S}}_{\tau _{k-1}}$. For such an edge, write $j(e)\in \left\{ 1,2\right\}$ for the index defined by $\underline{e}\in {\mathcal {S}}_{\tau _{k-1}}^{\scriptscriptstyle (j(e))}$. At time

3.39 $$\begin{aligned} \tau _k=\min _{e\in \partial {\mathcal {S}}_{\tau _{k-1}}} R_{j(e)}^{-1}\left( \Big . R_{j(e)}\left( \big . T^{\scriptscriptstyle {\mathcal {S}}}({\underline{e}}) \right) +Y_e^{\scriptscriptstyle (K_n)} \right) , \end{aligned}$$

we add the edge $e_k$ that attains the minimum in (3.39). Our assumptions on the edge weights $Y_e^{\scriptscriptstyle (K_n)}$ and the processes $R_1,R_2$ will imply that this minimum, and in addition the minimum

3.40 $$\begin{aligned} \min _{j\in \left\{ 1,2\right\} }\min _{e:e\in \partial {\mathcal {S}}^{(j)}_{\tau _{k-1}}} R_j^{-1}\left( R_j(T^{\scriptscriptstyle {\mathcal {S}}}({\underline{e}}))+Y_e^{\scriptscriptstyle (K_n)} \right) \end{aligned}$$

(with edges between ${\mathcal {S}}^{\scriptscriptstyle (1)}$ and ${\mathcal {S}}^{\scriptscriptstyle (2)}$ included), are uniquely attained a.s. We set ${\mathcal {S}}_t={\mathcal {S}}_{\tau _{k-1}}$ for $\tau _{k-1}\le t<\tau _k$, and we define ${\mathcal {S}}_{\tau _k}$ to be the graph obtained by adjoining $e_k$ to ${\mathcal {S}}_{\tau _{k-1}}$.

In the case $R_1(t)=R_2(t)=t$, ${\mathcal {S}}$ coincides with the two-source smallest-weight tree $\mathsf{SWT}^{\scriptscriptstyle (1,2)}$. In general, because the processes $R_1,R_2$ increase at variable speeds, the relationship between ${\mathcal {S}}$, $T^{\scriptscriptstyle {\mathcal {S}}}(i)$ and the FPP distances $d_{K_n,Y^{(K_n)}}(i,j)$ is subtle. For instance, it need not hold that $d_{K_n,Y^{(K_n)}}(1,i)=R_1(T^{\scriptscriptstyle {\mathcal {S}}}(i))$ for $i\in \cup _{t\ge 0}{\mathcal {S}}^{\scriptscriptstyle (1)}_t$. However, we have the following analogue of (3.7):

##### Lemma 3.20

(Minimal-weight representation) The weight of the optimal path $\pi _{1,2}$ from vertex 1 to vertex 2 is given by

3.41 $$\begin{aligned} W_n = \min _{i_1\in {\mathcal {S}}^{\scriptscriptstyle (1)},i_2\in {\mathcal {S}}^{\scriptscriptstyle (2)}} \left( R_1(T^{\scriptscriptstyle {\mathcal {S}}}(i_1)) + Y^{\scriptscriptstyle (K_n)}_{\left\{ i_1,i_2\right\} } + R_2(T^{\scriptscriptstyle {\mathcal {S}}}(i_2)) \right) , \end{aligned}$$

and the minimum is attained uniquely a.s.

The conclusion of Lemma 3.20 is easily seen when $R_1(t)=t, R_2(t)=0$ (in which case ${\mathcal {S}}^{\scriptscriptstyle (1)}$ is the same as $\mathsf{SWT}^{\scriptscriptstyle (1)}$ with vertex 2 removed) or when $R_1(t)=R_2(t)=t$ (in which case ${\mathcal {S}}$ reduces to $\mathsf{SWT}^{\scriptscriptstyle (1,2)}$). The proof of Lemma 3.20 in general requires some care, and is given in Sect. 5.3. The equality in (3.41) will be the basis of our analysis of $W_n$.

##### Definition 3.21

The *collision time* is

3.42 $$\begin{aligned} T_\mathrm{coll}= \inf \left\{ t\ge 0:R_1(t)+R_2(t)\ge W_n\right\} . \end{aligned}$$

The *collision edge* is the edge between the vertices $I_1\in {\mathcal {S}}^{\scriptscriptstyle (1)}$ and $I_2\in {\mathcal {S}}^{\scriptscriptstyle (2)}$ that attain the minimum in (3.41). We denote by $H(I_1)$, $H(I_2)$ the graph distance between 1 and $I_1$ in ${\mathcal {S}}^{\scriptscriptstyle (1)}$ and between 2 and $I_2$ in ${\mathcal {S}}^{\scriptscriptstyle (2)}$, respectively.

##### Theorem 3.22

(Exploration process at the collision time) The following statements hold almost surely: the endpoints $I_1,I_2$ of the collision edge are explored before time $T_\mathrm{coll}.$ The optimal path $\pi _{1,2}$ from vertex 1 to vertex 2 is the union of the unique path in ${\mathcal {S}}^{\scriptscriptstyle (1)}_{T_\mathrm{coll}}$ from 1 to $I_1;$ the collision edge $\{I_1,I_2\};$ and the unique path in ${\mathcal {S}}^{\scriptscriptstyle (2)}_{T_\mathrm{coll}}$ from $I_2$ to 2. The weight and hopcount satisfy

3.43 $$\begin{aligned} W_n = R_1(T_\mathrm{coll})+R_2(T_\mathrm{coll}), \quad H_n = H(I_1)+H(I_2)+1. \end{aligned}$$

Theorem 3.22 is proved in Sect. 5.3. The first equality in (3.43) is a simple consequence of continuity of $t\mapsto R_j(t)$ and the definition of $T_\mathrm{coll}$ in Definition 3.21.

##### Remark 3.23

The values of the process $({\mathcal {S}}_t)_{t\ge 0}$ depend implicitly on the choice of the processes $R_1,R_2$, which we have defined in terms of ${\mathcal {B}}$. Similarly, the value of $(T_{\mathrm{coll}},I_1,I_2)$ depends on the on-off processes $R_1(t),R_2(t)$, as well as on the edge weights $Y^{\scriptscriptstyle (K_n)}_{\left\{ i_1,i_2\right\} }$ for $i_1\in {\mathcal {S}}^{\scriptscriptstyle (1)},i_2\in {\mathcal {S}}^{\scriptscriptstyle (2)}$. In particular, the law of $(T_{\mathrm{coll}},I_1,I_2)$ will depend on the relationship between ${\mathcal {B}}$ and the edge weights $(Y_e^{\scriptscriptstyle (K_n)})$, which we will specify in Sect. 3.5.

However, regardless of the laws of ${\mathcal {S}}$ and of $(T_{\mathrm{coll}},I_1,I_2)$, the laws of $W_n$ and $H_n$ are the same.

### Coupling FPP on $K_n$ from Two Sources to a CTBP

In this section, we revisit the coupling of FPP on $K_n$ from two sources to a CTBP, which we initialized in Sect. 3.2, using the tools in Sect. 3.3 on vertex characteristics, as well as the freezing, unfreezing and collisions discussed in Sect. 3.4. In Sect. 3.5.1, we give the final conclusions of the coupling including freezing, and in Sect. 3.5.2, we relate the law of the weight of the smallest-weight path and its number of collision edges to a certain Cox process of collisions.

#### The Final Coupling Including Freezing

Similarly to Theorem 3.8, we next couple the FPP process ${\mathcal {S}}$ on $K_n$ and the FPP process ${\mathcal {B}}$ on ${\mathcal {T}}^{\scriptscriptstyle (1,2)}$. To this end, we introduce a thinning procedure for ${\mathcal {B}}=({\mathcal {B}}_t)_{t\ge 0}$: define $M_{\varnothing _j}=j$, for $j=1,2$. To each other $v\in {\mathcal {T}}^{\scriptscriptstyle (1,2)}\setminus \left\{ \varnothing _1,\varnothing _2\right\}$, we associate a mark $M_v$ chosen uniformly and independently from [*n*].

##### Definition 3.24

(*Thinning*) The vertex $v\in {\mathcal {T}}\setminus \left\{ \varnothing _1,\varnothing _2\right\}$ is *thinned* if it has an ancestor $v_0=p^{k}\!\left( v\right)$ (possibly *v* itself) such that $M_{v_0}=M_w$ for some unthinned vertex *w* with $T^{\mathcal {B}}_w<T^{\mathcal {B}}_{v_0}$.

The difference between Definition 3.24 and its closely related cousin Definition 3.9 is that Definition 3.24 includes freezing, which, as explained in Sect. 3.4, is crucial for our analysis. As with Definition 3.9, this definition is not circular, as vertices are investigated in their order of appearance. Write ${\widetilde{{\mathcal {B}}}}_t$ for the subgraph of ${\mathcal {B}}_t$ consisting of unthinned vertices.

From here onwards, we will work on a probability space that contains

- the two independent PWITs $({\mathcal {T}}^{\scriptscriptstyle (j)},X^{\scriptscriptstyle (j)})$, $j\in \left\{ 1,2\right\}$,
- the marks $$M_v$$, $$v \in {\mathcal {T}}^{\scriptscriptstyle (1,2)}$$,
- and a family of independent exponential random variables $E_e$, $e\in E(K_{\infty })$, with mean 1, independent of the PWITs and the marks.

Here $$E(K_\infty )=\left\{ \left\{ i,j\right\} :i,j \in {\mathbb {N}}, i< j\right\} $$.

On this probability space, we can construct the FPP edge weights on $K_n$ as follows. Let

3.44 $$\begin{aligned} T^{\scriptscriptstyle {\widetilde{{\mathcal {B}}}}}(i)=\inf \left\{ t \ge 0:M_v=i \text { for some } v\in {\widetilde{{\mathcal {B}}}}_t\right\} \end{aligned}$$

be the first time that a vertex with mark *i* appears in ${\widetilde{{\mathcal {B}}}}$ and denote the corresponding vertex by $V(i)\in {\mathcal {T}}^{\scriptscriptstyle (1,2)}$. Note that $T^{\scriptscriptstyle {\widetilde{{\mathcal {B}}}}}(i)$ is finite for all *i* almost surely since the FPP exploration process eventually explores every edge. For every edge $\left\{ i,i'\right\} \in E(K_n)$, we define

3.45 $$\begin{aligned} X(i,i')=\min \left\{ X_v :M_v=i', p\left( v\right) =V(i)\right\} , \end{aligned}$$

and

3.46 $$\begin{aligned} X_{\left\{ i,i'\right\} }^{\scriptscriptstyle (K_n)}= {\left\{ \begin{array}{ll} \tfrac{1}{n} X(i,i') &{} \text {if } T^{\scriptscriptstyle {\widetilde{{\mathcal {B}}}}}(i)<T^{\scriptscriptstyle {\widetilde{{\mathcal {B}}}}}(i'),\\ \tfrac{1}{n} X(i',i) &{} \text {if } T^{\scriptscriptstyle {\widetilde{{\mathcal {B}}}}}(i')<T^{\scriptscriptstyle {\widetilde{{\mathcal {B}}}}}(i),\\ E_{\left\{ i,i'\right\} } &{} \text {if } T^{\scriptscriptstyle {\widetilde{{\mathcal {B}}}}}(i)=T^{\scriptscriptstyle {\widetilde{{\mathcal {B}}}}}(i')=0. \end{array}\right. } \end{aligned}$$

The following proposition states that the random variables in (3.46) can be used to produce the correct edge weights on $K_n$ for our FPP problem:

##### Proposition 3.25

Let $(X_e^{\scriptscriptstyle (K_n)})_{e \in E(K_n)}$ be defined in (3.46), and write $Y_e^{\scriptscriptstyle (K_n)}=g(X_e^{\scriptscriptstyle (K_n)}),$ where *g* is the strictly increasing function from (2.1)–(2.2). Then the edge weights $(Y_e^{\scriptscriptstyle (K_n)})_{e\in E(K_n)}$ are i.i.d. with distribution function $F_{\scriptscriptstyle Y}$.

Proposition 3.25 will be generalized in Theorem 5.3 and proved in Sect. 5.4.

In Theorem 3.22, we have explained the (deterministic) relationship between ${\mathcal {S}}$ and the FPP problem with edge weights $Y_e^{\scriptscriptstyle (K_n)}$. Proposition 3.25 shows that, subject to (3.46), these edge weights have the desired distribution. We next explain the relationship between ${\mathcal {B}}$ and ${\mathcal {S}}$. Recall the subgraph $\pi _M(\tau )$ of $K_n$ introduced in Definition 3.7, which we extend to the case where $\tau \subset {\mathcal {T}}^{\scriptscriptstyle (1,2)}$.

##### Theorem 3.26

(The coupling with freezing) Under the edge-weight coupling (3.46), ${\mathcal {S}}_t=\pi _M({\widetilde{{\mathcal {B}}}}_t)$ for all $t\ge 0$ almost surely. Moreover, when ${\widetilde{{\mathcal {B}}}}$ and ${\mathcal {S}}$ are equipped with the FPP edge weights $Y_v=f_n(X_v)$ and $Y^{\scriptscriptstyle (K_n)}_e=g(X^{\scriptscriptstyle (K_n)}_e),$ respectively, the mapping $\pi _M:{\widetilde{{\mathcal {B}}}}\rightarrow {\mathcal {S}}$ is an isomorphism of edge-weighted graphs.

The proof is given in Sect. 5.4. Even though Theorem 3.26 is closely related to Theorem 3.10, it will be the version in Theorem 3.26 that we rely upon in our technical proofs. We now discuss its importance in more detail.

Theorem 3.26 achieves two goals. First, it relates the exploration process ${\mathcal {B}}$, defined in terms of two infinite underlying trees, to the smallest-weight tree process ${\mathcal {S}}$, defined in terms of a single finite graph. Because thinning gives an explicit coupling between these two objects, we will be able to control its effect, even when the total number of thinned vertices is relatively large. Consequently we will be able to study the FPP problem by analyzing a corresponding problem expressed in terms of ${\mathcal {B}}$ (see Theorems 3.27 and 3.28) and showing that whp thinning does not affect our conclusions (see Theorem 3.33).

Second, Theorem 3.26 allows us to relate FPP on the complete graph (*n*-independent dynamics run on an *n*-dependent weighted graph) with an exploration defined in terms of a pair of Poisson-weighted infinite trees (*n*-dependent dynamics run on an *n*-independent weighted graph). By analyzing the dynamics of ${\mathcal {B}}$ when *n* and $s_n$ are large, we obtain a fruitful *dual picture*: when the number of explored vertices is large, we find a *dynamic* rescaled branching process approximation that is essentially independent of *n*. When the number of explored vertices is small, we make use of a *static* approximation by invasion percolation found in [21]. In fact, under our scaling assumptions, FPP on the PWIT is closely related to *invasion percolation* (IP) on the PWIT which is defined as follows. Set $\mathrm{IP}^{\scriptscriptstyle (1)}(0)$ to be the subgraph consisting of $\varnothing _1$ only. For $k\in {\mathbb {N}}$, form $\mathrm{IP}^{\scriptscriptstyle (1)}(k)$ inductively by adjoining to $\mathrm{IP}^{\scriptscriptstyle (1)}(k-1)$ the boundary vertex $v\in \partial \mathrm{IP}^{\scriptscriptstyle (1)}(k-1)$ of minimal weight. We note that, since we consider only the relative ordering of the various edge weights, we can use either the PWIT edge weights $(X_v)_v$ or the FPP edge weights $(f_n(X_v))_v$.

Write $\mathrm{IP}^{\scriptscriptstyle (1)}(\infty )=\bigcup _{k=1}^\infty \mathrm{IP}^{\scriptscriptstyle (1)}(k)$ for the limiting subgraph. We remark that $\mathrm{IP}^{\scriptscriptstyle (1)}(\infty )$ is a strict subgraph of ${\mathcal {T}}^{\scriptscriptstyle (1)}$ a.s. (in contrast to FPP, which eventually explores every edge). Indeed, define

3.47 $$\begin{aligned} M^{\scriptscriptstyle (1)}=\sup \left\{ X_{v}:v\in \mathrm{IP}^{\scriptscriptstyle (1)}(\infty )\setminus \left\{ \varnothing _1\right\} \right\} , \end{aligned}$$

the largest weight of an invaded edge. Then ${\mathbb {P}}(M^{\scriptscriptstyle (1)}<x)$ is the survival probability of a Poisson Galton–Watson branching process with mean *x*, as in Theorems 1.1, 1.3 and 2.4.

Consequently, (2.9) in Theorem 2.4 can be read as a decomposition of the weight $W_n$ of the smallest-weight path into a deterministic part $\frac{1}{\lambda _n} \log (n/s_n^3)$ coming from the branching process dynamics and the weight of the largest edge explored by invasion percolation starting from two sources $f_n(M^{\scriptscriptstyle (1)}\vee M^{\scriptscriptstyle (2)})$.

#### A Cox Process for the Collisions

Similarly to Theorem 3.11, we can relate the collision time and collision edge to a Cox process driven by ${\mathcal {B}}$. To state this result, we will use a slightly different coupling between ${\mathcal {B}}$ and $K_n$ than the one just described. More precisely, we alter the definition (3.36) of ${\mathcal {B}}$ and work with a copy having the same law. This alteration only affects the thinned part ${\mathcal {B}}\setminus {\widetilde{{\mathcal {B}}}}$, and the pointwise relationships in (3.46) and Theorem 3.26 continue to apply. As discussed in Remark 3.23, this change affects the law of $({\mathcal {S}}, T_\mathrm{coll},I_1,I_2)$ but not of $W_n$ and $H_n$. The full details can be found in Sect. 5.5.

##### Theorem 3.27

(A Cox process for the collision edges with freezing) Let ${\mathcal {P}}_n$ be a Cox process on $\left[ 0,\infty \right) \times {\mathcal {T}}^{\scriptscriptstyle (1)} \times {\mathcal {T}}^{\scriptscriptstyle (2)}$ [with respect to the $\sigma$-algebra generated by ${\mathcal {B}},R_1,R_2$ and $(M_v)_{v\in {\mathcal {B}}}]$ with random intensity measure $Z_n=(Z_{n,t})_{t\ge 0}$ defined by

3.48 $$\begin{aligned}&Z_{n,t}(\left\{ v_1\right\} \times \left\{ v_2\right\} ) = {\mathbb {1}}_{\left\{ v_1\in {\mathcal {B}}_t^{\scriptscriptstyle (1)},v_2\in {\mathcal {B}}_t^{\scriptscriptstyle (2)}\right\} } \tfrac{1}{n} \mu _n\bigl (\Delta R_{v_1,v_2}, R_1(t)-R_1(T_{v_1}^{\mathcal {B}})+R_2(t)-R_2(T_{v_2}^{\mathcal {B}})\bigr ) \end{aligned}$$

for all $t\ge 0,$ where

3.49 $$\begin{aligned} \Delta R_{v_1,v_2}= {\left\{ \begin{array}{ll} R_1(T_{v_2}^{\mathcal {B}})-R_1(T_{v_1}^{\mathcal {B}}), &{} \text {if }T_{v_1}^{\mathcal {B}}\le T_{v_2}^{\mathcal {B}},\\ R_2(T_{v_1}^{\mathcal {B}})-R_2(T_{v_2}^{\mathcal {B}}), &{} \text {if }T_{v_2}^{\mathcal {B}}\le T_{v_1}^{\mathcal {B}}. \end{array}\right. } \end{aligned}$$

Let $(T_\mathrm{coll}^{\scriptscriptstyle ({\mathcal {P}}_n)},V_\mathrm{coll}^{\scriptscriptstyle (1)},V_\mathrm{coll}^{\scriptscriptstyle (2)})$ denote the first point of ${\mathcal {P}}_n$ for which $V_\mathrm{coll}^{\scriptscriptstyle (1)}$ and $V_\mathrm{coll}^{\scriptscriptstyle (2)}$ are unthinned. Then, for a suitable coupling, the law of $(T_\mathrm{coll}^{\scriptscriptstyle ({\mathcal {P}}_n)},R_1(T_\mathrm{coll}^{\scriptscriptstyle ({\mathcal {P}}_n)})+R_2(T_\mathrm{coll}^{\scriptscriptstyle ({\mathcal {P}}_n)}),\pi _M({\widetilde{{\mathcal {B}}}}_{T_\mathrm{coll}^{\scriptscriptstyle ({\mathcal {P}}_n)}}),M_{V_\mathrm{coll}^{\scriptscriptstyle (1)}},M_{V_\mathrm{coll}^{\scriptscriptstyle (2)}})$ is the same as the joint law of the collision time $T_\mathrm{coll};$ the optimal weight $W_n;$ the smallest-weight tree ${\mathcal {S}}_{T_\mathrm{coll}}$ at time $T_\mathrm{coll};$ and the endpoints $I_1,I_2$ of the collision edge. In particular, the hopcount $H_n$ has the same distribution as $\left| V_\mathrm{coll}^{\scriptscriptstyle (1)}\right| +\left| V_\mathrm{coll}^{\scriptscriptstyle (2)}\right| +1$.

Theorem 3.27 is the version of Theorem 3.11 that includes freezing.

*Sketch of the proof* By Lemma 3.20, Theorem 3.22 and the fact that $R_1+R_2$ is strictly increasing, $T_\mathrm{coll}>t$ is equivalent to $W_n>R_1(t)+R_2(t)$, which is in turn equivalent to

3.50 $$\begin{aligned} Y_{\left\{ i_1,i_2\right\} }^{\scriptscriptstyle (K_n)} > R_1(t)+R_2(t)-R_1(T^{\scriptscriptstyle {\mathcal {S}}}(i_1))-R_2(T^{\scriptscriptstyle {\mathcal {S}}}(i_2)) \quad \text {for all }i_1\in {\mathcal {S}}^{\scriptscriptstyle (1)}_t,\quad i_2\in {\mathcal {S}}^{\scriptscriptstyle (2)}_t . \end{aligned}$$

On the other hand, the fact that $i_1\in {\mathcal {S}}^{\scriptscriptstyle (1)}_t$ and $i_2\in {\mathcal {S}}^{\scriptscriptstyle (2)}_t$ implies that between the times $T^{\scriptscriptstyle {\mathcal {S}}}(i_1)\wedge T^{\scriptscriptstyle {\mathcal {S}}}(i_2)$ and $T^{\scriptscriptstyle {\mathcal {S}}}(i_1)\vee T^{\scriptscriptstyle {\mathcal {S}}}(i_2)$ when the first vertex and the second vertex of $\left\{ i_1,i_2\right\}$ were explored, respectively, the flow from the first explored vertex did not reach the other vertex. This translates to precisely the information that $Y_{\left\{ i_1,i_2\right\} }^{\scriptscriptstyle (K_n)}>\Delta R_{i_1,i_2}$.

Because of the relation ${\mathcal {S}}_t=\pi _M({\widetilde{{\mathcal {B}}}}_t)$, the set of pairs $(i_1,i_2)\in {\mathcal {S}}^{\scriptscriptstyle (1)}_t \times {\mathcal {S}}^{\scriptscriptstyle (2)}_t$ can be identified with the set of pairs of unthinned vertices $(v_1, v_2)\in {\widetilde{\mathsf{BP}}}^{\scriptscriptstyle (1)}_t \times {\widetilde{\mathsf{BP}}}^{\scriptscriptstyle (2)}_t$ via $i_j=M_{v_j}$. Moreover, under the edge-weight coupling (3.46), the connecting edge weight $Y^{\scriptscriptstyle (K_n)}_{\left\{ i_1,i_2\right\} }$ is determined based on the birth times of children of $v_1$ with mark $M_{v_2}$ (if $T^{\mathcal {B}}_{v_1}<T^{\mathcal {B}}_{v_2}$) or children of $v_2$ with mark $M_{v_2}$ (if $T^{\mathcal {B}}_{v_1}<T^{\mathcal {B}}_{v_2}$). In either case, given ${\mathcal {B}}_t$ and $(M_v)_{v\in {\mathcal {B}}_t}$ for $t\le T^{\mathcal {B}}_{v_1}\vee T^{\mathcal {B}}_{v_2}$, the conditional law of such birth times is Poisson with intensity given by (3.48).

To complete the proof, it remains to ensure that knowledge of ${\mathcal {B}}_t$ and $(M_v)_{v\in {\mathcal {B}}_t}$ for $t>T^{\mathcal {B}}_{{\tilde{v}}_1}\vee T^{\mathcal {B}}_{{\tilde{v}}_2}$ does not reveal any other information about the birth times used to determine the connecting edge weights $Y^{\scriptscriptstyle (K_n)}_{\left\{ i_1,i_2\right\} }$. We will accomplish this by redefining ${\mathcal {B}}$ and $R_1,R_2$ so that they use a conditionally independent copy of those birth times. See Sect. 5.5 for more details and the full proof of Theorem 3.27. $\square$

Theorem 3.27 means that we can study first passage percolation on $K_n$ by studying a CTBP problem and then controlling the effect of thinning. In fact, with Theorem 3.27 in hand, we will no longer need to refer to $K_n$ at all.

The remainder of the proof of Theorem 2.4 will be to use Theorems 3.26 and 3.27. In Sect. 3.6, we will first study the properties of the collision edges ignoring the thinning, and in Sect. 3.7, we will show that whp the first collision edge is not thinned to conclude the proof.

### The Collision Edge and Its Properties: Long Time Scales

Theorem 3.27 expresses the collision edge in terms of the first unthinned point of ${\mathcal {P}}_n$. We begin by stating the asymptotic behavior of the first point (whether thinned or not) of ${\mathcal {P}}_n$:

#### Theorem 3.28

(The first point of the Cox process) Let ${\mathcal {P}}_n$ be the Cox process in Theorem 3.27, and let $(T_\mathrm{first},V_\mathrm{first}^{\scriptscriptstyle (1)},V_\mathrm{first}^{\scriptscriptstyle (2)})$ denote its first point. Then

3.51 $$\begin{aligned} T_\mathrm{first}=T_\mathrm{unfr}+\frac{\log (n/s_n^3)}{2\lambda _n}+O_{\scriptscriptstyle {{\mathbb {P}}}}(1/\lambda _n). \end{aligned}$$

Furthermore, recalling the sequence $(\phi _n)_n$ from (3.18), the pair

3.52 $$\begin{aligned} \left( \frac{\left| V_\mathrm{first}^{\scriptscriptstyle (1)}\right| -\tfrac{1}{2}\phi _n\log (n/s_n^3)}{\sqrt{s_n^2\log (n/s_n^3)}} , \frac{\left| V_\mathrm{first}^{\scriptscriptstyle (2)}\right| -\tfrac{1}{2}\phi _n\log (n/s_n^3)}{\sqrt{s_n^2\log (n/s_n^3)}} \right) \end{aligned}$$

converges in distribution to a pair of independent normal random variables of mean 0 and variance $\tfrac{1}{2},$ and is asymptotically independent of $T_\mathrm{first}$ and of ${\mathcal {B}}$.

The proof of Theorem 3.28, presented at the end of the current section, is based on a general convergence result for Cox processes, which we now describe. Consider a sequence of Cox processes $({\mathcal {P}}_n^*)_n$ on ${\mathbb {R}}\times {\mathbb {R}}^2$ with random intensity measures $(Z_n^*)_n$, with respect to $\sigma$-fields $({\mathscr {F}}_n)_n$. We will write ${\mathcal {P}}_{n,t}^*$ for the measure defined by ${\mathcal {P}}_{n,t}^*(\cdot )={\mathcal {P}}_n^*(\left[ -\infty ,t\right) \times \cdot )$. Define

3.53 $$\begin{aligned} T_{n,k}^*=\inf \left\{ t:\left| {\mathcal {P}}_{n,t}^*\right| \ge k\right\} \end{aligned}$$

and let $A_{n,k}$ be the event that $T_{n,j}^*\notin \left\{ \pm \infty \right\}$ and $\vert {\mathcal {P}}_{n,T_{n,j}}^*\vert =j$, for $j=1,\ldots ,k$. That is, $A_{n,k}$ is the event that the points of ${\mathcal {P}}_n^*$ with the *k* smallest *t*-values are uniquely defined. On $A_{n,k}$, let $X_{n,k}$ denote the unique point for which ${\mathcal {P}}_n^*(\left\{ T_{n,k}^*\right\} \times \left\{ X_{n,k}\right\} )=1$, and otherwise set $X_{n,k}=\dagger$, an isolated cemetery point.

The following theorem gives a sufficient condition for the first points of such a Cox process to converge towards independent realizations of a probability measure *Q*. To state it, we write

3.54 $$\begin{aligned} {\hat{R}}({\vec {\xi }})=\int _{{\mathbb {R}}^d} \! {\mathrm e}^{\vec {\xi }\cdot \vec {x}} dR(\vec {x}) \end{aligned}$$

for the moment generating function of a measure *R* on ${\mathbb {R}}^d$ and $\vec {\xi } \in {\mathbb {R}}^d$.

#### Theorem 3.29

Fix a probability measure *Q* on ${\mathbb {R}}^2$ with ${\hat{Q}}(\vec {\xi })<\infty$ for all $\vec {\xi } \in {\mathbb {R}}^2,$ a non-decreasing continuous function $q:{\mathbb {R}}\rightarrow (0,\infty )$ satisfying $\lim _{t\rightarrow -\infty }q(t)=0.$ Suppose that we can find a decomposition $Z_n^*=Z^{\prime \scriptscriptstyle (K)}_n+Z^{\prime \prime \scriptscriptstyle (K)}_n$ for each $K>0,$ and sub-$\sigma$-fields ${\mathscr {F}}'_n\subset {\mathscr {F}}_n,$ such that

- for each fixed $\varepsilon >0, t,u\in {\mathbb {R}}, \vec {\xi }\in {\mathbb {R}}^2,$ there exists $K_0<\infty$ such that, for all $K\ge K_0,$
  3.55 $$\begin{aligned} (1-\varepsilon )q(t){\hat{Q}}(\vec {\xi })\le {\mathbb {E}}\left( \left. {\hat{Z}}^{\prime \scriptscriptstyle (K)}_{n,t}(\vec {\xi })\,\right| {\mathscr {F}}'_n\right) \le (1+\varepsilon )q(t){\hat{Q}}(\vec {\xi }), \end{aligned}$$
  3.56 $$\begin{aligned}&{\mathbb {E}}\left( \left. \Big ( \frac{{\hat{Z}}^{\prime \scriptscriptstyle (K)}_{n,t}(\vec {\xi })}{q(t){\hat{Q}}(\vec {\xi })}-\frac{\left| Z^{\prime \scriptscriptstyle (K)}_{n,u}\right| }{q(u)} \Big )^2\,\right| {\mathscr {F}}'_n\right) \le \varepsilon ,&\text {and} \end{aligned}$$
  3.57 $$\begin{aligned} {\mathbb {E}}\left( \left. \vert Z^{\prime \prime \scriptscriptstyle (K)}_{n,t}\vert \,\right| {\mathscr {F}}'_n\right) < \varepsilon q(t), \end{aligned}$$
  all this with probability at least $1-\varepsilon$ for *n* sufficiently large; and
- for each $\varepsilon >0,$ there exists ${\overline{t}}$ such that
  3.58 $$\begin{aligned} \liminf _{n\rightarrow \infty }{\mathbb {P}}\left( \big \vert Z_{n,{\overline{t}}}^*\big \vert >1/\varepsilon \right) \ge 1-\varepsilon . \end{aligned}$$

Then the random sequence $(X_{n,j})_{j=1}^\infty$ converges in distribution to an i.i.d. random sequence $(X_j)_{j=1}^\infty$ where $X_j$ has law *Q*. Moreover $\left\{ (T_{n,j})_{j=1}^k:n\in {\mathbb {N}}\right\}$ is tight, $(X_{n,j})_{j=1}^\infty$ is asymptotically independent of ${\mathscr {F}}_n$ and, if $(T_j,X_j)_{j=1}^\infty$ is any subsequential limit of $(T_{n,j},X_{n,j})_{j=1}^\infty ,$ then $(T_j)_{j=1}^\infty$ and $(X_j)_{j=1}^\infty$ are independent.

Theorem 3.29 is proved in Sect. 8.

To apply Theorem 3.29, we will rescale and recentre both time and the heights of vertices. Furthermore, we will remove the effect of the frozen cluster ${\mathcal {B}}_\mathrm{fr}$.

#### Definition 3.30

(*Rescaling and recentering*) For $v\in {\mathcal {T}}^{\scriptscriptstyle (1,2)}\setminus {\mathcal {B}}_\mathrm{fr}$, define $p^\mathrm{unfr}(v)$ to be the unique ancestor $v'$ of *v* for which $v'\in \partial {\mathcal {B}}_\mathrm{fr}$ (with $p^\mathrm{unfr}(v)=v$ if $p\left( v\right) \in {\mathcal {B}}_\mathrm{fr}$). Write

3.59 $$\begin{aligned} \left| v\right| ^*&= \frac{\left| v\right| -\left| p^\mathrm{unfr}(v)\right| - \tfrac{1}{2}\phi _n \log (n/s_n^3)}{ \sqrt{s_n^2\log (n/s_n^3)}} , \end{aligned}$$

3.60 $$\begin{aligned} t^*&= \lambda _n(1)(t -T_\mathrm{unfr}) - \tfrac{1}{2}\log (n/s_n^3) . \end{aligned}$$

Define ${\mathcal {P}}_n^*$ to be the image under the mapping $(t,v_1,v_2)\mapsto (t^*,\left| v_1\right| ^*,\left| v_2\right| ^*)$ of the restriction of ${\mathcal {P}}_n$ to $\left[ 0,\infty \right) \times ({\mathcal {T}}^{\scriptscriptstyle (1)}\setminus {\mathcal {B}}_\mathrm{fr}^{\scriptscriptstyle (1)})\times ({\mathcal {T}}^{\scriptscriptstyle (2)}\setminus {\mathcal {B}}_\mathrm{fr}^{\scriptscriptstyle (2)})$.

#### Theorem 3.31

(Our collision Cox process is nice) The point measures $({\mathcal {P}}_n^*)_n$ are Cox processes and satisfy the hypotheses of Theorem 3.29 when *Q* is the law of a pair of independent $N\left( 0,\tfrac{1}{2}\right)$ random variables, $q(t^*)={\mathrm e}^{2t^*},$ and ${\mathscr {F}}'_n$ is the $\sigma$-field generated by the frozen cluster ${\mathcal {B}}_\mathrm{fr}$.

We prove Theorem 3.31 in Sect. 9.3. All the vertices relevant to ${\mathcal {P}}_n^*$ are born after the unfreezing time $T_\mathrm{unfr}$, and therefore appear according to certain CTBPs. Theorem 3.31 will therefore be proved by a first and second moment analysis of the two-vertex characteristics from Sect. 3.3.2.

To use Theorem 3.31 in the proof of Theorem 3.28, we will show that the first point $(T_\mathrm{first}^*,H_1^*,H_2^*)$ of ${\mathcal {P}}_n^*$ and the first point $(T_\mathrm{first},V_\mathrm{first}^{\scriptscriptstyle (1)},V_\mathrm{first}^{\scriptscriptstyle (2)})$ of ${\mathcal {P}}_n$ are whp related as in (3.59)–(3.60). This will follow from part (b) of the following lemma, which we will prove in Sects. 9.2 and 9.4:

#### Lemma 3.32

Let $K<\infty$ and ${\overline{t}}=T_\mathrm{unfr}+\lambda _n(1)^{-1}\left( \frac{1}{2} \log (n/s_n^3) +K\right)$. Then

- $$\left| {\mathcal {B}}_{{\overline{t}}}\right| =O_{{\mathbb {P}}}(\sqrt{ns_n});$$ and
- $${\mathcal {P}}_n\left( [0,{\overline{t}}]\times {\mathcal {B}}_\mathrm{fr}^{\scriptscriptstyle (1)}\times {\mathcal {T}}^{\scriptscriptstyle (2)} \right) = {\mathcal {P}}_n\left( [0,{\overline{t}}]\times {\mathcal {T}}^{\scriptscriptstyle (1)}\times {\mathcal {B}}_\mathrm{fr}^{\scriptscriptstyle (2)} \right) = 0\,whp.$$

Assuming Lemma 3.32 (b) and Theorem 3.31, we can now prove Theorem 3.28:

#### Proof of Theorem 3.28

By construction, the first point $(T_\mathrm{first}^*,H_1^*,H_2^*)$ of ${\mathcal {P}}_n^*$ is the image of some point $(T,V_1,V_2)$ of ${\mathcal {P}}_n$ under the mapping $(t,v_1,v_2)\mapsto (t^*,\left| v_1\right| ^*,\left| v_2\right| ^*)$. Theorems 3.29 and 3.31 imply that $T_\mathrm{first}^*=O_{\mathbb {P}}(1)$, so that $T=T_\mathrm{unfr}+\lambda _n(1)^{-1}\left( \tfrac{1}{2}\log (n/s_n^3)+\right. \left. O_{\mathbb {P}}(1)\right)$ by (3.60). We may therefore apply Lemma 3.32 (b) to conclude that ${\mathcal {P}}_n\left( [0,T]\times {\mathcal {B}}_\mathrm{fr}^{\scriptscriptstyle (1)}\right. \left. \times {\mathcal {T}}^{\scriptscriptstyle (2)} \right) = {\mathcal {P}}_n\left( [0,T]\times {\mathcal {T}}^{\scriptscriptstyle (1)}\times {\mathcal {B}}_\mathrm{fr}^{\scriptscriptstyle (2)} \right) = 0\,whp.$

In particular, whp, $$(T,V_1,V_2)$$ equals the first point $$(T_\mathrm{first},V_\mathrm{first}^{\scriptscriptstyle (1)},V_\mathrm{first}^{\scriptscriptstyle (2)})$$ of $${\mathcal {P}}_n$$, and therefore $$H_j^*=\left| V_\mathrm{first}^{\scriptscriptstyle (j)}\right| ^*$$. In Theorem 3.28, the heights are to be rescaled as in (3.52) rather than (3.59). However, these differ only by the term $$\left| p^\mathrm{unfr}(V_\mathrm{first}^{\scriptscriptstyle (j)})\right| /s_n\sqrt{\log (n/s_n^3)}$$. By Theorem 3.18 (c), we have $$\left| p^\mathrm{unfr}(V_\mathrm{first}^{\scriptscriptstyle (j)})\right| =1+O_{\mathbb {P}}(s_n)$$, since $$p(p^\mathrm{unfr}(V_\mathrm{first}^{\scriptscriptstyle (j)}))\in {\mathcal {B}}^{\scriptscriptstyle (j)}_\mathrm{fr}$$ by construction. Hence the term $$\left| p^\mathrm{unfr}(V_\mathrm{first}^{\scriptscriptstyle (j)})\right| /s_n\sqrt{\log (n/s_n^3)}$$ is $$o_{\mathbb {P}}(1)$$. Finally, the asymptotic independence statements follow from those in Theorem 5.3 and (3.51) follows from the tightness of $$T_\mathrm{first}^*.$$$$\square $$

### Thinning and Completion of the Proof

In this section, we explain that the first point of the Cox process is whp unthinned and conclude our main results:

#### Theorem 3.33

(First point of Cox process is whp unthinned) Let ${\mathcal {P}}_n$ be the Cox process in Theorem 3.27, and let $(T_\mathrm{first},V_\mathrm{first}^{\scriptscriptstyle (1)},V_\mathrm{first}^{\scriptscriptstyle (2)})$ denote its first point. Then $V_\mathrm{first}^{\scriptscriptstyle (1)}$ and $V_\mathrm{first}^{\scriptscriptstyle (2)}$ are whp unthinned. Consequently, whp $T_\mathrm{coll}^{\scriptscriptstyle ({\mathcal {P}}_n)}=T_\mathrm{first},V_\mathrm{coll}^{\scriptscriptstyle (1)}=V_\mathrm{first}^{\scriptscriptstyle (1)}, V_\mathrm{coll}^{\scriptscriptstyle (2)}=V_\mathrm{first}^{\scriptscriptstyle (2)}.$

#### Proof

According to Definition 3.24, the vertex $V_\mathrm{first}^{\scriptscriptstyle (j)}$, $j\in \left\{ 1,2\right\}$, will be thinned if and only if some non-root ancestor $v_0$ of $V_\mathrm{first}^{\scriptscriptstyle (j)}$ has $M_{v_0}=M_w$, where $w\in {\mathcal {B}}_{T_\mathrm{first}}$ is unthinned and $T_w^{\mathcal {B}}<T_{v_0}^{\mathcal {B}}$. We obtain an upper bound by dropping the requirement that *w* should be unthinned and relaxing the condition $T_w^{\mathcal {B}}<T_{v_0}^{\mathcal {B}}$ to $T_w^{\mathcal {B}}\le T_\mathrm{first}$ and $w\ne v_0$. Each such pair of vertices $(v_0,w)$ has conditional probability 1/*n* of having the same mark, so, by a union bound,

3.61 $$\begin{aligned} {\mathbb {P}}\left( \left. \big .V_\mathrm{first}^{\scriptscriptstyle (1)}\text { or }V_\mathrm{first}^{\scriptscriptstyle (2)}\text { is thinned}\,\right| V_\mathrm{first}^{\scriptscriptstyle (1)},V_\mathrm{first}^{\scriptscriptstyle (2)},\left| {\mathcal {B}}_{T_{\mathrm{first}}}\right| \right) \le \tfrac{1}{n}(\left| V_\mathrm{first}^{\scriptscriptstyle (1)}\right| +\left| V_\mathrm{first}^{\scriptscriptstyle (2)}\right| )\left| {\mathcal {B}}_{T_{\mathrm{first}}}\right| . \end{aligned}$$

By Theorem 3.28, $\left| V_\mathrm{first}^{\scriptscriptstyle (j)}\right| =O_{\mathbb {P}}(s_n\log (n/s_n^3))$. Moreover $T_\mathrm{first}=T_\mathrm{unfr}+\lambda _n(1)^{-1}\left( \tfrac{1}{2}\log (n/s_n^3)+O_{\mathbb {P}}(1)\right)$, so that $\left| {\mathcal {B}}_{T_\mathrm{first}}\right| =O_{\mathbb {P}}(\sqrt{ns_n})$ by Lemma 3.32 (a). Hence

3.62 $$\begin{aligned} {\mathbb {P}}\left( \left. \big .V_\mathrm{first}^{\scriptscriptstyle (1)}\text { or }V_\mathrm{first}^{\scriptscriptstyle (2)}\text { is thinned}\,\right| V_\mathrm{first}^{\scriptscriptstyle (1)},V_\mathrm{first}^{\scriptscriptstyle (2)},\left| {\mathcal {B}}_{T_{\mathrm{first}}}\right| \right) \le O_{\mathbb {P}}\left( \frac{\log (n/s_n^3)}{\sqrt{n/s_n^3}} \right) , \end{aligned}$$

and this upper bound is $o_{\mathbb {P}}(1)$ since $n/s_n^3\rightarrow \infty$. $\square$

Note that other choices of $R_1(t),R_2(t)$ would make Theorem 3.33 false. For the first point of ${\mathcal {P}}_n$ to appear, the intensity measure $Z_{n,t}$, which is given by 1/*n* times a sum over ${\mathcal {B}}_t^{\scriptscriptstyle (1)}\times {\mathcal {B}}_t^{\scriptscriptstyle (2)}$, must be of order 1. If $R_1(t)=t$, $R_2(t)=0$, for instance, then ${\mathcal {B}}_t^{\scriptscriptstyle (2)}$ is small and it follows that ${\mathcal {B}}_t^{\scriptscriptstyle (1)}$ must be large (of size at least of order *n*) at time $t=T_\mathrm{first}$. In this case thinning would have a very strong effect. We note that this argument applies even to relatively well-behaved edge distributions such as the $E^s$ edge weights considered in [11], where the exploration must proceed simultaneously from both endpoints with $R_1(t)=R_2(t)=t$.

In the heavy-tailed case that we consider, even the symmetric choice $R_1(t)=R_2(t)=t$ is in effect unbalanced. Indeed, at the earlier of the two freezing times, $t=\min \left\{ T_\mathrm{fr}^{\scriptscriptstyle (1)},T_\mathrm{fr}^{\scriptscriptstyle (2)}\right\}$, the faster-growing cluster has reached size $O_{\scriptscriptstyle {{\mathbb {P}}}}(s_n^2)$, whereas

3.63 $$\begin{aligned} \min \left\{ T_\mathrm{fr}^{\scriptscriptstyle (1)},T_\mathrm{fr}^{\scriptscriptstyle (2)}\right\} \approx f_n(\min \left\{ M^{\scriptscriptstyle (1)},M^{\scriptscriptstyle (2)}\right\} ) < f_n(\max \left\{ M^{\scriptscriptstyle (1)},M^{\scriptscriptstyle (2)}\right\} ) \end{aligned}$$

(see Theorem 3.18) implies that the slower-growing cluster has not yet explored the unique edge of weight $\max \left\{ M^{\scriptscriptstyle (1)},M^{\scriptscriptstyle (2)}\right\}$ and therefore has size $O_{\scriptscriptstyle {{\mathbb {P}}}}(1)$. This is a crucial reason for introducing the freezing procedure of Sect. 3.4.

We are now ready to complete the proof of Theorem 2.4:

#### Proof of Theorem 2.4

According to Lemma 2.7, we can assume Conditions 2.1, 2.2 and 2.6. We begin with the hopcount result (2.10). By Theorem 3.27, $H_n \overset{d}{=}\left| V_\mathrm{coll}^{\scriptscriptstyle (1)}\right| +\left| V_\mathrm{coll}^{\scriptscriptstyle (2)}\right| +1$, where $(T_\mathrm{coll}^{\scriptscriptstyle ({\mathcal {P}}_n)},V_\mathrm{coll}^{\scriptscriptstyle (1)},V_\mathrm{coll}^{\scriptscriptstyle (2)})$ is the first point of the Cox process ${\mathcal {P}}_n$ for which $V_\mathrm{coll}^{\scriptscriptstyle (1)}$ and $V_\mathrm{coll}^{\scriptscriptstyle (1)}$ are unthinned. By Theorem 3.33, $T_\mathrm{coll}^{\scriptscriptstyle ({\mathcal {P}}_n)}=T_\mathrm{first}$ whp, so that the pairs $(V_\mathrm{coll}^{\scriptscriptstyle (1)},V_\mathrm{coll}^{\scriptscriptstyle (2)})$ and $(V_\mathrm{first}^{\scriptscriptstyle (1)},V_\mathrm{first}^{\scriptscriptstyle (2)})$ in Theorems 3.27 and 3.28 are the same whp. Hence, whp,

3.64 $$\begin{aligned} \frac{H_n-\phi _n\log (n/s_n^3)}{\sqrt{s_n^2 \log (n/s_n^3)}}&\overset{d}{=}\frac{\left| V_\mathrm{first}^{\scriptscriptstyle (1)}\right| -\tfrac{1}{2}\phi _n\log (n/s_n^3)}{\sqrt{s_n^2 \log (n/s_n^3)}} + \frac{\left| V_\mathrm{first}^{\scriptscriptstyle (2)}\right| -\tfrac{1}{2}\phi _n\log (n/s_n^3)}{\sqrt{s_n^2 \log (n/s_n^3)}} + o_{\scriptscriptstyle {{\mathbb {P}}}}(1) , \end{aligned}$$

so that Theorem 3.28 implies the CLT for $H_n$ in (2.10).

For the weight result (2.9), Theorem 3.27 states that $W_n \overset{d}{=}R_1(T_\mathrm{coll}^{\scriptscriptstyle ({\mathcal {P}}_n)})+R_2(T_\mathrm{coll}^{\scriptscriptstyle ({\mathcal {P}}_n)})$. On the event $\left\{ T_\mathrm{first}\ge T_\mathrm{unfr}\right\} \cap \left\{ T_\mathrm{first}=T_\mathrm{coll}^{\scriptscriptstyle ({\mathcal {P}}_n)}\right\}$ (which, by Theorem 3.28 and the argument above, occurs whp), the definition (3.35) of $R_1(t),R_2(t)$ leads to $R_1(T_\mathrm{coll}^{\scriptscriptstyle ({\mathcal {P}}_n)})+R_2(T_\mathrm{coll}^{\scriptscriptstyle ({\mathcal {P}}_n)})=T_\mathrm{fr}^{\scriptscriptstyle (1)}+T_\mathrm{fr}^{\scriptscriptstyle (2)}+2(T_\mathrm{first}-T_\mathrm{unfr})$. Using again Theorem 3.28, we obtain

3.65 $$\begin{aligned} W_n \overset{d}{=}T_\mathrm{fr}^{\scriptscriptstyle (1)}+T_\mathrm{fr}^{\scriptscriptstyle (2)}+\frac{\log (n/s_n^3)}{\lambda _n}+O_{\scriptscriptstyle {{\mathbb {P}}}}(1/\lambda _n). \end{aligned}$$

Therefore, (2.9) follows from Theorem 3.18 (a) and Lemmas 3.12 and 3.19. Finally, the independence of the limiting variables follows from the asymptotic independence in Theorem 3.28.

$$\square $$

**Organisation of this paper.** In the remainder of the paper, we use the outline given in Sects. 2.1–3.7 and give the details and proofs omitted there. We have already mentioned how these results complete the proof of our main results, as well as where these results are proved, we thus now restrict to the relation to the local behavior as described in the companion paper [21]. While [21] focusses on the local behaviour of FPP on $K_n$, in this paper we extend the analysis to the *global* behavior. We will rely on some results from [21], but in a highly localized way. Indeed, in Sect. 5 we rely on some coupling results from [21], in particular [Part I, Theorem 3.4] and some of its extensions. In Sect. 9, we mainly rely on [Part I, Theorem 2.15] and some related results. The other sections do not rely on [21].

## Scaling Properties of $f_n$ and $\mu _n$

In this section, we derive several useful consequences of Conditions 2.1, 2.2 and 2.6, including Lemma 3.19. We also verify that the edge-weight distributions in Examples 1.2 and 2.5 satisfy the hypotheses of Theorem 2.4, and we prove Lemma 2.7, showing that we may without loss of generality assume the stronger condition Condition 2.6 in place of Condition 2.3.

### Growth and Density Bounds for $f_n$ and $\mu _n$

In this section, we explore the key implications of Conditions 2.1–2.2 and 2.6 on $f_n$ and on the intensity measure $\mu _n$.

#### Lemma 4.1

Assume Conditions 2.2 and 2.6. Then there exists $n_0 \in {\mathbb {N}}$ such that

4.1 $$\begin{aligned} f_n(x)\le \left( \frac{x}{x'} \right) ^{\varepsilon _0s_n} f_n(x') \quad \text {whenever }1-\delta _0\le x\le x',\quad n \ge n_0 . \end{aligned}$$

#### Proof

Divide (2.7) or (2.12) by *x* and integrate between *x* and $x'$ to obtain $\log {f_n(x')}-\log {f_n(x)}\ge \varepsilon _0s_n \left( \log x' - \log x \right)$ whenever $1-\delta _0\le x\le x'$, $n \ge n_0$, as claimed. $\square$

We call Condition 2.6 a density bound because it implies the following lemma, which will also be useful in the study of two-vertex characteristics in Sect. 7:

#### Lemma 4.2

Assume Conditions 2.2 and 2.6. Then, for *n* sufficiently large, on the interval $(f_n(1-\delta _0),\infty ),$ the measure $\mu _n$ is absolutely continuous with respect to Lebesgue measure and

4.2 $$\begin{aligned} {\mathbb {1}}_{\left\{ y>f_n(1-\delta _0)\right\} } d\mu _n(y) \le \frac{1}{\varepsilon _0s_n} \frac{f_n^{-1}(y)}{y} dy. \end{aligned}$$

#### Proof

By Conditions 2.2 and 2.6, $f_n$ is strictly increasing on $(1-\delta _0,\infty )$, so $y=f_n(\mu _n(0,y))$ for $y>f_n(1-\delta _0)$. Differentiating and again applying Conditions 2.2 and 2.6, we get

$$\begin{aligned} 1=f'_n(\mu _n(0,y)) \frac{d}{dy}\mu _n(0,y) \ge \varepsilon _0s_n \frac{f_n(\mu _n(0,y))}{\mu _n(0,y)} \frac{d}{dy}\mu _n(0,y) , \quad y>f_n(1-\delta _0). \end{aligned}$$

$$\square $$

#### Lemma 4.3

Assume Conditions 2.2 and 2.6. Then, for *n* sufficiently large, the density of $\mu _n$ with respect to Lebesgue measure is at most $1/(\varepsilon _0s_n f_n(1))$ on the interval $(f_n(1),\infty )$.

#### Proof

From Lemma 4.1 it follows immediately that $f_n^{-1}(y)\le (y/f_n(1))^{1/\varepsilon _0s_n}\le y/f_n(1)$ for all $y> f_n(1)$ and sufficiently large *n*. The result now follows from Lemma 4.2. $\square$

#### Lemma 4.4

Assume Conditions 2.2 and 2.6. Then, given $\varepsilon , {\bar{\varepsilon }}>0,$ there exist $n_0 \in {\mathbb {N}}$ and $K<\infty$ such that, for all $n \ge n_0$ and $t\ge 0,$

4.3 $$\begin{aligned} \int {\mathrm e}^{-\varepsilon y/f_n(1)}{\mathbb {1}}_{\left\{ y\ge Kf_n(1)\right\} } \mu _n(t+dy)\le {\bar{\varepsilon }}/s_n. \end{aligned}$$

#### Proof

By Lemma 4.3, for large *n*, the density of $\mu _n$ with respect to Lebesgue measure is bounded from above by $1/(\varepsilon _0s_n f_n(1))$ on $(f_n(1),\infty )$. Hence, for $K>1$,

$$\begin{aligned} \int {\mathrm e}^{-\varepsilon y/f_n(1)}{\mathbb {1}}_{\left\{ y\ge Kf_n(1)\right\} } \mu _n(t+dy)\le \int _t^{\infty } {\mathrm e}^{-\varepsilon (y-t) /f_n(1)}{\mathbb {1}}_{\left\{ y-t\ge Kf_n(1)\right\} } \frac{dy}{\varepsilon _0s_n f_n(1)} = \frac{{\mathrm e}^{-\varepsilon K}}{\varepsilon _0s_n \varepsilon }. \end{aligned}$$

Taking *K* sufficiently large proves the claim. $\square$

#### Lemma 4.5

Assume Conditions 2.1, 2.2 and 2.6. Then, given $K<\infty ,$ there exist $\varepsilon _K>0$ and $n_0\in {\mathbb {N}}$ such that, for $0\le t\le K f_n(1)$ and $n\ge n_0,$

4.4 $$\begin{aligned} \int {\mathrm e}^{-\lambda _n(1) y} \mu _n(t+dy)\ge \varepsilon _K/s_n. \end{aligned}$$

#### Proof

For any $$0\le t\le Kf_n(1)$$,

4.5 $$\begin{aligned} \int {\mathrm e}^{-\lambda _n(1)y} \mu _n(t+dy) = \int {\mathrm e}^{-\lambda _n(1)(y-t)} {\mathbb {1}}_{\left\{ y\ge t\right\} } d\mu _n(y) \ge {\mathrm e}^{-2\lambda _n(1)Kf_n(1)} \mu _n(Kf_n(1), 2Kf_n(1)). \end{aligned}$$

By Lemma 3.12, $\lambda _n f_n(1)$ converges to a finite constant. Since $f_n$ is strictly increasing on $(1-\delta _0,\infty )$, $y=f_n(f_n^{-1}(y))$ for all $y >f_n(1-\delta _0)$. Writing $x_n = f_n^{-1}(y)$ and using Conditions 2.1 and 2.6, we get

$$\begin{aligned} \frac{y (f_n^{-1})'(y)}{f_n^{-1}(y)} = \Big [\frac{x_n f_n'(x_n)}{f_n(x_n)}\Big ]^{-1} \le (\varepsilon _0 s_n)^{-1} \quad \forall y >f_n(1-\delta _0). \end{aligned}$$

By Condition 2.1, $f_n(1+x/s_n)/f_n(1)\rightarrow {\mathrm e}^x$ for every given *x*, and it follows with Taylor’s theorem that $f_n^{-1}(f_n(1)K) \sim f_n^{-1}\left( f_n\left( 1+\frac{\log K}{s_n}\right) \right)$ and, therefore, $\mu _n(Kf_n(1),2Kf_n(1))=f_n^{-1}(2Kf_n(1))-f_n^{-1}(Kf_n(1)) \sim (\log 2)/s_n$. $\square$

We are now in the position to prove Lemma 3.19:

#### Proof of Lemma 3.19

By monotonicity, $f_n^{-1}(T_n^{\scriptscriptstyle (1)}+T_n^{\scriptscriptstyle (2)}) \ge \max \left\{ f_n^{-1}(T_n^{\scriptscriptstyle (1)}),f_n^{-1}(T_n^{\scriptscriptstyle (2)})\right\} {\mathop {\longrightarrow }\limits ^{\scriptscriptstyle {{\mathbb {P}}}}}{\mathcal {M}}^{\scriptscriptstyle (1)} \vee {\mathcal {M}}^{\scriptscriptstyle (2)}$. For the matching upper bound, let $T_n=T_n^{\scriptscriptstyle (1)} \vee T_n^{\scriptscriptstyle (2)}$ and ${\mathcal {M}}={\mathcal {M}}^{\scriptscriptstyle (1)} \vee {\mathcal {M}}^{\scriptscriptstyle (2)}$, so that $f_n^{-1}(T_n){\mathop {\longrightarrow }\limits ^{\scriptscriptstyle {{\mathbb {P}}}}}{\mathcal {M}}$. Noting that $f_n^{-1}(T_n^{\scriptscriptstyle (1)}+T_n^{\scriptscriptstyle (2)}) \le f_n^{-1}(2 T_n)$, it suffices to show that $f_n^{-1}(2T_n){\mathop {\longrightarrow }\limits ^{\scriptscriptstyle {{\mathbb {P}}}}}{\mathcal {M}}$. But for $\delta >0$, Lemma 4.1 implies that $f_n(x+\delta )/f_n(x)$ tends to infinity, and is in particular larger than 2 for *n* sufficiently large, uniformly over $x\in [1,R]$ for any $R<\infty$. It follows that $f_n^{-1}(2T_n)\le (1\vee f_n^{-1}(T_n))+\delta$ with high probability, for any $\delta >0$. Since ${\mathcal {M}}\ge 1$ a.s. and $\delta >0$ was arbitrary, this completes the proof. $\square$

We conclude the section with a remark on the connection between Theorem 2.4 above and [Part I, Theorem 2.1] which states that, if $s_n/\log \log n \rightarrow \infty$, then

4.6 $$\begin{aligned} f_n^{-1}(W_n) {\mathop {\longrightarrow }\limits ^{d}}M^{\scriptscriptstyle (1)}\vee M^{\scriptscriptstyle (2)}. \end{aligned}$$

#### Remark 4.6

The statements for $W_n$ in Theorem 2.4 and (4.6) are consistent. Indeed, if $s_n/\log \log n\rightarrow \infty$, then Lemmas 4.1 and 3.12 imply

4.7 $$\begin{aligned} f_n^{-1}\Big (\frac{1}{\lambda _n} \log \big (n/s_n^3\big )\Big ) \le \Big (\frac{\log (n/s_n^3)}{\lambda _n f_n(1)}\Big )^{1/\varepsilon _0 s_n}=(1+o(1)) \exp \Big (\frac{1}{\varepsilon _0 s_n} \log \log (n/s_n^3)\Big )=1+o(1). \end{aligned}$$

Hence, Lemma 3.19 gives that (2.9) and (4.6) agree if $s_n/\log \log n\rightarrow \infty$.

### Analysis of Specific Edge-Weight Distributions

#### Lemma 4.7

With the notation of (2.2)–(2.3), the relation (1.3) [with $t\mapsto L(t)$ slowly varying as $t\rightarrow \infty ]$ holds if and only if

4.8 $$\begin{aligned} x\frac{d}{dx}\log g(x) = x^{-\alpha } {\tilde{L}}(1/x) \end{aligned}$$

with $$t\mapsto {\tilde{L}}(t)$$ slowly varying as $$t\rightarrow \infty $$. If either of these two equivalent conditions hold, then $$L(t)\sim {\tilde{L}}(t)$$ as $$t\rightarrow \infty $$ and the sequences $$(s_n)_n,$$$$({\tilde{s}}_n)_n$$ defined by (1.4) and

4.9 $$\begin{aligned} {\tilde{s}}_n = \frac{f_n'(1)}{f_n(1)} \end{aligned}$$

satisfy $s_n\sim {\tilde{s}}_n$ as $n\rightarrow \infty$. Moreover if in addition $s_n\rightarrow \infty$ (or equivalently ${\tilde{s}}_n\rightarrow \infty )$ then Conditions 2.1, 2.2 and 2.3 (a) hold for the sequences $(f_n(x),s_n)$ or $(f_n(x),{\tilde{s}}_n)$. If the stronger statement $s_n/\log \log n\rightarrow \infty$ (or equivalently ${\tilde{s}}_n/\log \log n\rightarrow \infty )$ holds, then Condition 2.3 (b) holds.

#### Proof

The proof is virtually identical to the proof of [Part I, Lemma 5.4], and we indicate only those parts of the proof that differ.

We prove Condition 2.1 for $f_n$ and the sequence ${\tilde{s}}_n$ defined in (4.9). We compute

4.10 $$\begin{aligned} \frac{1}{{\tilde{s}}_n}\frac{xf_n'(x)}{f_n(x)}=\frac{g(1/n)}{(1/n)g'(1/n)}\frac{\frac{x}{n}g'\left( \frac{x}{n}\right) }{g\left( \frac{x}{n}\right) }=x^{-\alpha } \frac{{\widetilde{L}}(n/x)}{{\widetilde{L}}(n)}. \end{aligned}$$

Noting that $xf_n'(x)/f_n(x)$ is the derivative of $\log f_n(x)$ with respect to $q=\log x$, we find

4.11 $$\begin{aligned} \frac{f_n(x^{1/s_n})}{f_n(1)} = \exp \left( \int _0^{\log (x^{1/s_n})} s_n {\mathrm e}^{-\alpha q} \frac{{\widetilde{L}}(n{\mathrm e}^{-q})}{{\widetilde{L}}(n)} dq \right) = \exp \left( \int _0^{\log x} {\mathrm e}^{-\alpha \theta /s_n} \frac{{\widetilde{L}}(n{\mathrm e}^{-\theta /s_n})}{{\widetilde{L}}(n)} d\theta \right) , \end{aligned}$$

after the substitution $\theta =s_n q$. As $n\rightarrow \infty$, we have ${\tilde{s}}_n\rightarrow \infty$ by assumption, so that the last integrand converges to 1 pointwise. For each fixed *x*, the convergence is uniform over $\theta$ by properties of slowly varying functions, so that $f_n(x^{1/s_n})/f_n(1)\rightarrow {\mathrm e}^{\log x}=x$ as required. (This argument remains valid, interchanging the limits of integration as necessary, when $x<1$.)

For Condition 2.3 (b), we note that if $s_n/\log \log n\rightarrow \infty$ then Condition 2.3 (a) implies Condition 2.3 (b) because, for any $\varepsilon>0, R>1$,

$$\begin{aligned} \frac{f_n(R)}{f_n(1)\log n} \ge \frac{R^{\varepsilon s_n}}{\log n} = \exp \left( \varepsilon \log R s_n - \log \log n \right) \rightarrow \infty . \end{aligned}$$

$$\square $$

#### Lemma 4.8

The edge-weight distributions and the associated sequences $(s_n)_n$ from Example 1.2 (for all values of the parameters $a,\gamma$ and $\beta )$ and Example 2.5 satisfy Conditions 2.1–2.3.

#### Proof

For Example 1.2, it is readily verified that the regular-variation condition (1.3) holds. By Lemma 4.7 it remains to show that Condition 2.3 (b) holds when $s_n/\log \log n\nrightarrow \infty$, i.e., for Example 1.2 (b) with $0<\gamma \le 1$. It suffices to find a sequence $x_n$ with $g(x_n/n)/g(1/n)\log n\rightarrow \infty$ such that $s_n^{-1} xg'(x)/g(x)\rightarrow 1$ uniformly over $1/n\le x\le x_n/n$; in fact we will take $x_n=\log n$.

Since $u\mapsto u^{a(\log (1+\log (1/u)))^\gamma }$ is increasing, it follows that, with $q=\log (1/u)$,

4.12 $$\begin{aligned} F_{\scriptscriptstyle Y}^{-1}(u) = u^{a(\log (1+\log (1/u)))^\gamma } = \exp \left( -a q(\log (1+q))^\gamma \right) . \end{aligned}$$

Setting $u=1-{\mathrm e}^{-x}$ and $q=\log (1/u)=-\log (1-{\mathrm e}^{-x})$, (2.2) and the chain rule lead to

4.13 $$\begin{aligned} \frac{xg'(x)}{g(x)} = \frac{x{\mathrm e}^{-x}}{1-{\mathrm e}^{-x}} \left( a(\log (1+q))^\gamma + a\gamma \frac{q}{1+q}(\log (1+q))^{\gamma -1} \right) . \end{aligned}$$

In particular, we see that $xg'(x)/g(x)$ is a slowly-varying function of $q=-\log (1-{\mathrm e}^{-x})$, say $xg'(x)/g(x)=h(q)$ where $q\mapsto h(q)$ is slowly varying as $x\downarrow 0$, $q\rightarrow \infty$. Since $q=\log (1/x)+O(x)$ as $x\downarrow 0$, we find that $q=\log n + O(\log \log n)+O((\log n)/n)=(\log n)(1+o(1))$ uniformly on $1/n\le x\le (\log n)/n$, and consequently

4.14 $$\begin{aligned} \frac{1}{s_n} \frac{xg'(x)}{g(x)} = \frac{h(q)}{h(-\log (1-{\mathrm e}^{-1/n}))} = 1+o(1) \end{aligned}$$

uniformly on $1/n\le x\le (\log n)/n$ by properties of slowly varying functions, as required. On the other hand $xg'(x)/g(x)\rightarrow \infty$ as $x\downarrow 0$ implies in particular that $xg'(x)/g(x)\ge 2$ for *x* sufficiently small, so that

4.15 $$\begin{aligned} \frac{g((\log n)/n)}{g(1/n)} \ge (\log n)^2 \end{aligned}$$

for *n* sufficiently large and we have shown that $g(x_n/n)/g(1/n)\log n\rightarrow \infty$, as required.

For Example 2.5, we compute $f_n(x)=\left( \big . G^{-1}(1-{\mathrm e}^{-x/n}) \right) ^{s_n}$ and

4.16 $$\begin{aligned} \frac{1}{s_n} \frac{xf_n'(x)}{f_n(x)} = \frac{(x/n)(G^{-1})'(1-{\mathrm e}^{-x/n})}{G^{-1}(1-{\mathrm e}^{-x/n})}. \end{aligned}$$

Write $a=G'(0)>0$. Since *Z* is positive-valued, we have $G(0)=0$ and therefore $u/G^{-1}(u)\rightarrow a$ as $u\downarrow 0$, whereas $(G^{-1})'(u)\rightarrow 1/a$ as $u\rightarrow 0$. This implies that the quantity in (4.16) tends to 1 whenever $x/n\rightarrow 0$, which allows us to conclude Conditions 2.2 and 2.3 (a). Similarly, from $s_n\rightarrow \infty$ we can infer that $xf_n'(x)/f_n(x)\ge 2$, uniformly over $x\le \log n$, for *n* sufficiently large, whence $f_n(\log n)/f_n(1)\log n\rightarrow \infty$ and Condition 2.3 (b) holds. Finally, as in (4.11), we find

4.17 $$\begin{aligned} \frac{f_n(x^{1/s_n})}{f_n(1)} = \exp \left( \int _0^{\log x} \left. \frac{1}{s_n}\frac{{\tilde{x}} f_n'({\tilde{x}})}{f_n({\tilde{x}})} \right| _{{\tilde{x}}={\mathrm e}^{\theta /s_n}} d\theta \right) \end{aligned}$$

and comparing with (4.16), the integrand converges to 1 as $n\rightarrow \infty$, uniformly over $\theta$ for any fixed *x*, and Condition 2.1 follows. $\square$

### Equivalence of Conditions: Proof of Lemma 2.7

The proof of Lemma 2.7 is based on the observation that if the functions $f_n,{\tilde{f}}_n$ agree on the interval $[0,{\overline{x}}]$ then, for the FPP problems with edge weights $f_n(nX_e^{\scriptscriptstyle (K_n)})$ and ${\tilde{f}}_n(nX_e^{\scriptscriptstyle (K_n)})$, respectively, the optimal paths and their corresponding edge weights are identical whenever either optimal path has weight less than $f_n({\overline{x}})={\tilde{f}}_n({\overline{x}})$.

#### Proof of Lemma 2.7

Let $\delta _0$ be the constant from Condition 2.2, let $R>1$, and define

4.18 $$\begin{aligned} x_{n,R}= & {} R \vee \big (\inf \left\{ x\ge 1:f_n(x)\ge 4{\mathrm e}^\gamma f_n(1)\log n\right\} \big ),\nonumber \\ \varepsilon _0^{\scriptscriptstyle (R)}= & {} \frac{1}{2} \liminf \limits _{n\rightarrow \infty } \inf \limits _{1-\delta _0\le x \le x_{n,R}} \frac{x }{s_n} \frac{d}{dx} \log f_n(x). \end{aligned}$$

Conditions 2.2 and 2.3 imply that $\varepsilon _0^{\scriptscriptstyle (R)}>0$ for any $R>1$, and there exists $n_0^{\scriptscriptstyle (R)}\in {\mathbb {N}}$ such that $x \frac{d}{dx} \log f_n(x)\ge \varepsilon _0^{\scriptscriptstyle (R)}s_n$ for $x\in [1-\delta _0, x_{n,R}]$ whenever $n\ge n_0^{\scriptscriptstyle (R)}$. For definiteness, we take $n_0^{\scriptscriptstyle (R)}$ minimal with this property. We may uniquely define $f_{n,R}:\left[ 0,\infty \right) \rightarrow \left[ 0,\infty \right)$ by requiring that $f_{n,R}=f_n$ if $n<n_0^{\scriptscriptstyle (R)}$ and if $n\ge n_0^{\scriptscriptstyle (R)}$ then (a) $f_{n,R}(x)=f_n(x)$ for all $x\le x_{n,R}$, and (b) $\frac{x}{s_n} \frac{d}{dx} \log f_{n,R}(x)$ is constant on $\left[ x_{n,R},\infty \right)$. By construction, the sequence $(f_{n,R})_{n}$ satisfies Condition 2.6 for any fixed $R>1$. Furthermore, given any $x>0$, $R>1$ implies that $x^{1/s_n}\le R\le x_{n,R}$ for *n* sufficiently large, and it follows that $f_{n,R}$ satisfies Condition 2.1. Since $x_{n,R}\ge R>1$ it follows that Condition 2.2 holds for $(f_{n,R})_{n}$, too.

Let $\mu _{n,R}$ and $\lambda _{n,R}$ denote the analogues of $\mu _n$ and $\lambda _n$ when $f_n$ is replaced by $f_{n,R}$, and let $\lambda _{n,R}=\lambda _{n,R}(1)$ and $\phi _{n,R}=\lambda '_{n,R}(1)/\lambda _{n,R}(1)$ denote the corresponding parameters [see (3.11) and (3.16)–(3.18)]. Let $W_{n,R}, H_{n,R}$ denote the weight and hopcount, respectively, associated to the FPP problem on $K_n$ with edge weights $f_{n,R}(n X_e^{\scriptscriptstyle (K_n)})$. Abbreviate $w_{n,R}=W_{n,R}-\log (n/s_n^3)/\lambda _{n,R}$, $h_{n,R}=(H_{n,R}-\phi _{n,R}\log (n/s_n^3))/\sqrt{s_n^2\log (n/s_n^3)}$.

By assumption, Theorem 2.4 holds assuming Conditions 2.1, 2.2 and 2.6. Therefore, it applies to $f_{n,R}$. Using Theorem 2.4, we conclude that for any $k\in {\mathbb {N}}$, we may find $n_0^{\scriptscriptstyle (R,k)}\in {\mathbb {N}}$ such that $n_0^{\scriptscriptstyle (R,k)}\ge n_0^{\scriptscriptstyle (R)}$ and

4.19a $$\begin{aligned}&\sup _{x,y\in {\mathbb {R}}} \left| {\mathbb {P}}\left( f_{n,R}^{-1}\left( w_{n,R} \right) \le x, h_{n,R} \le y \right) - {\mathbb {P}}(M^{\scriptscriptstyle (1)}\vee M^{\scriptscriptstyle (2)}\le x){\mathbb {P}}(Z\le y) \right| \le \frac{1}{k}, \end{aligned}$$

4.19b $$\begin{aligned}&\left| \lambda _{n,R}f_{n,R}(1)-{\mathrm e}^{-\gamma }\right| \le \frac{1}{k}, \quad \left| \frac{\phi _{n,R}}{s_n}-1\right| \le \frac{1}{k}, \end{aligned}$$

4.19c $$\begin{aligned}&f_{n,R}(R-1) + \frac{\log (n/s_n^3)}{\lambda _{n,R}} \le f_{n,R}(R) \vee 4{\mathrm e}^\gamma f_n(1)\log n \end{aligned}$$

whenever $n\ge n_0^{\scriptscriptstyle (R,k)}$, and for definiteness we take $n_0^{\scriptscriptstyle (R,k)}$ minimal with these properties. Indeed, using the continuity of $M^{\scriptscriptstyle (1)}\vee M^{\scriptscriptstyle (2)}$ and *Z*, the uniform convergence in (4.19a) follows from the pointwise convergence at a finite grid $((x_i,y_i))_i$ depending on *k* and monotonicity of the distribution functions. For (4.19c), use the inequality $a+b\le 2(a\vee b)$, Lemma 3.12, and note that $2f_{n,R}(R-1)\le f_{n,R}(R)$ for *n* sufficiently large by Lemma 4.1. Set

4.20 $$\begin{aligned} R_n=\big (2 \vee \max \left\{ k\in {\mathbb {N}}:n\ge n_0^{\scriptscriptstyle (k,k)}\right\} \big ) \wedge n, \quad \text {and}\quad \lambda _n=\lambda _{n,R_n}, \quad \phi _n=\phi _{n,R_n}. \end{aligned}$$

Since $n_0^{\scriptscriptstyle (k,k)}$ is finite for each $k\in {\mathbb {N}}$, it follows that $R_n\rightarrow \infty$. Moreover, as soon as $n\ge n_0^{\scriptscriptstyle (2,2)}$, we have $n\ge n_0^{\scriptscriptstyle (R_n,R_n)}$, so that (4.19a)–(4.19c) hold with $(R,k)=(R_n,R_n)$. By construction, $f_{n,R}(1)=f_n(1)$, and we conclude in particular that $\phi _n/s_n\rightarrow 1$ and $\lambda _n f_n(1)\rightarrow {\mathrm e}^{-\gamma }$.

Given two functions $f_n$ and ${\tilde{f}}_n$, we can couple the corresponding FPP problems by choosing edge weights $f_n(nX_e^{\scriptscriptstyle (K_n)})$ and ${\tilde{f}}_n(nX_e^{\scriptscriptstyle (K_n)})$, respectively. Let ${\bar{x}}>0$. On the event $\left\{ W_n \le f_n({\bar{x}})\right\}$, the optimal path $\pi _{1,2}$ uses only edges of weight at most $f_n({\bar{x}})$. If $f_n$ and ${\tilde{f}}_n$ agree on the interval $[0,{\bar{x}}]$, then the edges along that path have the same weights in the two FPP problems and we deduce that $W_n={\tilde{W}}_n$ and $H_n={\tilde{H}}_n$, where ${\tilde{W}}_n$ and ${\tilde{H}}_n$ are the weight and the hopcount of the optimal path in the problem corresponding to ${\tilde{f}}_n$.

Consequently, on the event $\left\{ W_{n,R_n}\le f_{n,R_n}(x_{n,R_n})\right\}$, $W_n=W_{n,R_n}$ and $H_n=H_{n,R_n}$. By (4.19a), it remains to show that this event occurs whp. Since $R_n\rightarrow \infty$, we conclude from (4.19a) that $W_{n,R_n}\le f_{n,R_n}(R_n-1)+\log (n/s_n^3)/\lambda _{n,R_n}$ whp. But from the definition of $x_{n,R_n}$ and $f_{n,R_n}$ it follows that $f_{n,R_n}(x_{n,R_n})\ge f_{n,R_n}(R_n)\vee 4{\mathrm e}^\gamma f_{n,R_n}(1)\log n$, so (4.19c) completes the proof. $\square$

## Coupling $K_n$ and the PWIT

In Theorem 3.26, we indicated that two random processes, the first passage exploration processes ${\mathcal {S}}$ and ${\mathcal {B}}$ on $K_n$ and ${\mathcal {T}}^{\scriptscriptstyle (1,2)}$, respectively, could be coupled. In this section we explain how this coupling arises as a special case of a general family of couplings between $K_n$, understood as a random edge-weighted graph with i.i.d. exponential edge weights, and the PWIT. We rely on some results from the companion paper [21], in particular [Part I, Theorem 3.4, Lemma 3.6 and Proposition 3.7].

### Exploration Processes and the Definition of the Coupling

As in Sect. 3.5, we define $M_{\varnothing _j}=j$, for $j=1,2$, and to each $v\in {\mathcal {T}}^{\scriptscriptstyle (1,2)}\setminus \left\{ \varnothing _1,\varnothing _2\right\}$, we associate a mark $M_v$ chosen uniformly and independently from [*n*]. We next define what an exploration process is:

#### Definition 5.1

(*Exploration process on two PWITs*) Let ${\mathscr {F}}_0$ be a $\sigma$-field containing all null sets, and let $({\mathcal {T}}^{\scriptscriptstyle (1,2)},X)$ be independent of ${\mathscr {F}}_0$. We call a sequence ${\mathcal {E}}=({\mathcal {E}}_k)_{k\in {\mathbb {N}}_0}$ of subsets of ${\mathcal {T}}^{\scriptscriptstyle (1,2)}$ an *exploration process* if, with probability 1, ${\mathcal {E}}_0=\left\{ \varnothing _1,\varnothing _2\right\}$ and, for every $k\in {\mathbb {N}}$, either ${\mathcal {E}}_k={\mathcal {E}}_{k-1}$ or else ${\mathcal {E}}_k$ is formed by adjoining to ${\mathcal {E}}_{k-1}$ a previously unexplored child $v_k\in \partial {\mathcal {E}}_{k-1}$, where the choice of $v_k$ depends only on the weights $X_w$ and marks $M_w$ for vertices $w \in {\mathcal {E}}_{k-1}\cup \partial {\mathcal {E}}_{k-1}$ and on events in ${\mathscr {F}}_0$.

Examples for exploration processes are given by FPP and IP on ${\mathcal {T}}^{\scriptscriptstyle (1,2)}$. For FPP, as defined in Definition 3.4, it is necessary to convert to discrete time by observing the branching process at those moments when a new vertex is added. The standard IP on ${\mathcal {T}}^{\scriptscriptstyle (1,2)}$ is defined as follows. Set $\mathrm{IP}(0)=\left\{ \varnothing _1,\varnothing _2\right\}$. For $k \in {\mathbb {N}}$, form $\mathrm{IP}(k)$ inductively by adjoining to $\mathrm{IP}(k-1)$ the boundary vertex $v\in \partial \mathrm{IP}(k-1)$ of minimal weight. However, an exploration process is also obtained when we specify at each step (in any suitably measurable way) whether to perform an invasion step in ${\mathcal {T}}^{\scriptscriptstyle (1)}$ or ${\mathcal {T}}^{\scriptscriptstyle (2)}$.

For $k \in {\mathbb {N}}$, let ${\mathscr {F}}_k$ be the $\sigma$-field generated by ${\mathscr {F}}_0$ together with the weights $X_w$ and marks $M_w$ for vertices $w \in {\mathcal {E}}_{k-1}\cup \partial {\mathcal {E}}_{k-1}$. Note that the requirement on the choice of $v_k$ in Definition 5.1 can be expressed as the requirement that ${\mathcal {E}}$ is $({\mathscr {F}}_k)_k$-adapted.

For $v\in {\mathcal {T}}^{\scriptscriptstyle (1,2)}$, define the exploration time of *v* by

5.1 $$\begin{aligned} N_v=\inf \left\{ k \in {\mathbb {N}}_0:v\in {\mathcal {E}}_k\right\} . \end{aligned}$$

#### Definition 5.2

(*Thinning of two PWITs*) The vertex $v\in {\mathcal {T}}^{\scriptscriptstyle (1,2)}\setminus \left\{ \varnothing _1,\varnothing _2\right\}$ is *thinned* if it has an ancestor $v_0=p^{k}\!\left( v\right)$ (possibly *v* itself) such that $M_{v_0}=M_w$ for some unthinned vertex *w* with $N_w<N_{v_0}$. Write ${\widetilde{{\mathcal {E}}}}_k$ for the subgraph of ${\mathcal {E}}_k$ consisting of unthinned vertices.

We define the stopping times

5.2 $$\begin{aligned} N(i)=\inf \left\{ k \in {\mathbb {N}}_0:M_v=i\text { for some }v\in {\widetilde{{\mathcal {E}}}}_k\right\} \end{aligned}$$

at which $i\in [n]$ first appears as a mark in the unthinned exploration process. Note that, on the event $\left\{ N(i)<\infty \right\}$, ${\widetilde{{\mathcal {E}}}}_k$ contains a *unique* vertex in ${\mathcal {T}}^{\scriptscriptstyle (1,2)}$ whose mark is *i*, for any $k\ge N(i)$; call that vertex *V*(*i*). On this event, we define

5.3 $$\begin{aligned} X(i,i')=\min \left\{ X_w :M_w=i', p\left( w\right) =V(i)\right\} . \end{aligned}$$

We define, for an edge $\left\{ i,i'\right\} \in E(K_n)$,

5.4 $$\begin{aligned} X_{\left\{ i,i'\right\} }^{\scriptscriptstyle (K_n)}= {\left\{ \begin{array}{ll} \tfrac{1}{n} X(i,i') &{} \text {if } N(i)<N(i'),\\ \tfrac{1}{n} X(i',i) &{} \text {if } N(i')<N(i),\\ E_{\left\{ i,i'\right\} } &{} \text {if } N(i)=N(i')=\infty \text { or } N(i)=N(i')=0, \end{array}\right. } \end{aligned}$$

where $(E_e)_{e\in E(K_n)}$ are exponential variables with mean 1, independent of each other and of $(X_v)_{v}$.

#### Theorem 5.3

If ${\mathcal {E}}$ is an exploration process on the union ${\mathcal {T}}^{\scriptscriptstyle (1,2)}$ of two PWITs, then the edge weights $X_e^{\scriptscriptstyle (K_n)}$ defined in (5.4) are exponential with mean 1,  independently for each $e \in E(K_n)$.

The idea underlying Theorem 5.3 is that each variable $\tfrac{1}{n}X(i,i')$ is exponentially distributed conditionally on the past up to the moment *N*(*i*) when it may be used to set the value of $X_{\left\{ i,i'\right\} }^{\scriptscriptstyle (K_n)}$. Theorem 5.3 restates [Part I, Theorem 3.4] and is proved in that paper.

### Minimal-Rule Exploration Processes

An important class of exploration processes, which includes both FPP and IP, are those exploration processes determined by a minimal rule in the following sense:

#### Definition 5.4

A *minimal rule* for an exploration process ${\mathcal {E}}$ on ${\mathcal {T}}^{\scriptscriptstyle (1,2)}$ is an $({\mathscr {F}}_k)_k$-adapted sequence $(S_k,\prec _k)_{k=1}^\infty$, where $S_k\subset \partial {\mathcal {E}}_{k-1}$ is a (possibly empty) subset of the boundary vertices of ${\mathcal {E}}_{k-1}$ and $\prec _k$ is a strict total ordering of the elements of $S_k$ (if any) such that the implication

5.5 $$\begin{aligned} w\in S_k,\quad p\left( v\right) =p\left( w\right) , \quad M_v=M_w,\quad X_v<X_w \;\implies \; v\in S_k, v \prec _k w \end{aligned}$$

holds. An exploration process is *determined by the minimal rule* $(S_k,\prec _k)_{k=1}^\infty$ if ${\mathcal {E}}_k={\mathcal {E}}_{k-1}$ whenever $S_k=\varnothing$ and otherwise ${\mathcal {E}}_k$ is formed by adjoining to ${\mathcal {E}}_{k-1}$ the unique vertex $v_k\in S_k$ that is minimal with respect to $\prec _k$.

In words, in every step *k* there is a set of boundary vertices $S_k$ from which we can select for the next exploration step. The content of (5.5) is that, whenever a vertex $w\in S_k$ is available for selection, then all siblings of *w* with the same mark but smaller weight are also available for selection and are preferred over *w*.

For FPP without freezing on ${\mathcal {T}}^{\scriptscriptstyle (1,2)}$ with edge weights $f_n(X_v)$, we take $v \prec _k w$ if and only if $T_v < T_w$ [recall (3.10)] and take $S_k=\partial {\mathcal {E}}_{k-1}$. For IP on ${\mathcal {T}}^{\scriptscriptstyle (1,2)}$, we have $v \prec _k w$ if and only if $X_v<X_w$; the choice of subset $S_k$ can be used to enforce, for instance, whether the *k*th step is taken in ${\mathcal {T}}^{\scriptscriptstyle (1)}$ or ${\mathcal {T}}^{\scriptscriptstyle (2)}$.

Recall the subtree ${\widetilde{{\mathcal {E}}}}_k$ of unthinned vertices from Definition 5.2 and the subgraph $\pi _M({\widetilde{{\mathcal {E}}}}_k)$ from Definition 3.7. That is, $\pi _M({\widetilde{{\mathcal {E}}}}_k)$ is the union of two trees with roots 1 and 2, respectively, and for $v\in {\widetilde{{\mathcal {E}}}}_k \setminus \left\{ \varnothing _1,\varnothing _2\right\}$, $\pi _M({\widetilde{{\mathcal {E}}}}_k)$ contains vertices $M_v$ and $M_{p\left( v\right) }$ and the edge $\left\{ M_v, M_{p\left( v\right) }\right\}$.

For any $i\in [n]$ for which $N(i)<\infty$, recall that *V*(*i*) is the unique vertex of ${\widetilde{{\mathcal {E}}}}_k$ ($k\ge N(i)$) for which $M_{V(i)}=i$. Define $V(i,i')$ to be the first child of *V*(*i*) with mark $i'$.

Recalling (5.3), an equivalent characterization of $V(i,i')$ is

5.6 $$\begin{aligned} X(i,i')=X_{V(i,i')}. \end{aligned}$$

The following lemma shows that, for an exploration process determined by a minimal rule, unthinned vertices must have the form $V(i,i')$:

#### Lemma 5.5

Suppose ${\mathcal {E}}$ is an exploration process determined by a minimal rule $(S_k,\prec _k)_{k=1}^\infty$ and $k\in {\mathbb {N}}$ is such that ${\widetilde{{\mathcal {E}}}}_k\ne {\widetilde{{\mathcal {E}}}}_{k-1}$. Let $i_k=M_{p\left( v_k\right) }$ and $i_k'=M_{v_k}$. Then $v_k=V(i_k,i_k')$.

See the proof of [Part I, Lemma 3.6].

If ${\mathcal {E}}$ is an exploration process determined by a minimal rule, then we define

5.7 $$\begin{aligned} S_k^{\scriptscriptstyle (K_n)}=\left\{ \left\{ i,i'\right\} \in E(K_n) :i \in \pi _M({\widetilde{{\mathcal {E}}}}_{k-1}), i' \notin \pi _M({\widetilde{{\mathcal {E}}}}_{k-1}), V(i,i') \in S_k\right\} \end{aligned}$$

and

5.8 $$\begin{aligned} e_1 \;{\widetilde{\prec }}_k\; e_2 \;\iff \; V(i_1,i_1') \prec _k V(i_2,i_2'), \quad e_1,e_2\in S_k^{\scriptscriptstyle (K_n)}, \end{aligned}$$

where $e_j=\left\{ i_j,i'_j\right\}$ and $i_j \in \pi _M({\widetilde{{\mathcal {E}}}}_{k-1}), i'_j \notin \pi _M({\widetilde{{\mathcal {E}}}}_{k-1})$ as in (5.7).

#### Proposition 5.6

(Thinned minimal rule) Suppose ${\mathcal {E}}$ is an exploration process determined by a minimal rule $(S_k,\prec _k)_{k=1}^\infty$. Then, under the edge-weight coupling (5.4), the edge weights of $\pi _M({\widetilde{{\mathcal {E}}}}_k)$ are determined by

5.9 $$\begin{aligned} X_{\{M_v,M_{p\left( v\right) }\}}^{\scriptscriptstyle (K_n)} = \tfrac{1}{n} X_v \quad \text {for any }v\in \cup _{k=1}^\infty {\widetilde{{\mathcal {E}}}}_k\setminus \left\{ \varnothing _1,\varnothing _2\right\} \end{aligned}$$

and generally

5.10 $$\begin{aligned} X_{\left\{ i,i'\right\} }^{\scriptscriptstyle (K_n)} = \tfrac{1}{n} X_{V(i,i')} \quad \text {whenever} \quad i\in \pi _M({\widetilde{{\mathcal {E}}}}_{k-1}),\quad i'\notin \pi _M({\widetilde{{\mathcal {E}}}}_{k-1})\quad \text {for some }k\in {\mathbb {N}}. \end{aligned}$$

Moreover, for any $$k\in {\mathbb {N}}$$ for which $${\widetilde{{\mathcal {E}}}}_k\ne {\widetilde{{\mathcal {E}}}}_{k-1},$$$$\pi _M({\widetilde{{\mathcal {E}}}}_k)$$ is formed by adjoining to $$\pi _M({\widetilde{{\mathcal {E}}}}_{k-1})$$ the unique edge $$e_k\in S_k^{\scriptscriptstyle (K_n)}$$ that is minimal with respect to $${\widetilde{\prec }}_k$$.

Proposition 5.6 asserts that the subgraph $\pi _M({\widetilde{{\mathcal {E}}}}_k)$ of $K_n$, equipped with the edge weights $(X_e^{\scriptscriptstyle (K_n)})_{e\in E(\pi _M({\widetilde{{\mathcal {E}}}}_k))}$, is isomorphic as an edge-weighted graph to the subgraph ${\widetilde{{\mathcal {E}}}}_k$ of ${\mathcal {T}}^{\scriptscriptstyle (1,2)}$, equipped with the edge weights $\left( \tfrac{1}{n} X_v\right) _{v\in {\widetilde{{\mathcal {E}}}}_k\setminus \left\{ \varnothing _1,\varnothing _2\right\} }$. Furthermore, the subgraphs $\pi _M({\widetilde{{\mathcal {E}}}}_k)$ can be grown by an inductive rule. Thus the induced subgraphs $(\pi _M({\widetilde{{\mathcal {E}}}}_k))_{k=0}^\infty$ themselves form a minimal-rule exploration process on $K_n$, with a minimal rule derived from that of ${\mathcal {E}}$, with the caveat that ${\widetilde{\prec }}_k$ may depend on edge weights from ${\mathcal {E}}_{k-1}\setminus {\widetilde{{\mathcal {E}}}}_{k-1}$ as well as from $\pi _M({\widetilde{{\mathcal {E}}}}_{k-1})$.

See the proof of [Part I, Proposition 3.7] for the proof of Proposition 5.6.

### FPP and the Two Smallest-Weight Trees: Proof of Theorem 3.22

In this section, we discuss the relationship between ${\mathcal {S}}$ and FPP distances, and we prove Lemma 3.20 and Theorem 3.22.

#### Proof of Lemma 3.20

Define

5.11 $$\begin{aligned} {\bar{{\mathcal {S}}}}_u={\bar{{\mathcal {S}}}}_u^{\scriptscriptstyle (1)}\cup {\bar{{\mathcal {S}}}}_u^{\scriptscriptstyle (2)}, \quad {\bar{{\mathcal {S}}}}_u^{\scriptscriptstyle (j)}=\mathsf{SWT}^{\scriptscriptstyle (j)}_{R_j(u)}. \end{aligned}$$

To describe the discrete-time evolution of ${\bar{{\mathcal {S}}}}=({\bar{{\mathcal {S}}}}_u)_{u \ge 0}$, denote by ${\bar{\tau }}_{k-1}$ the time where the $(k-1)$th vertex (not including vertices 1 and 2) was added to ${\bar{{\mathcal {S}}}}$. At time $u=0$, ${\bar{{\mathcal {S}}}}_0$ is equal to ${\mathcal {S}}_0$, and contains vertex 1 and 2 and no edges. Having constructed the process until time ${\bar{\tau }}_{k-1}$, at time

5.12 $$\begin{aligned} {\bar{\tau }}_k&= \min _{j\in \left\{ 1,2\right\} }\min _{e\in \partial {\bar{{\mathcal {S}}}}_{{\bar{\tau }}_{k-1}}^{\scriptscriptstyle (j)}} R_j^{-1}\left( d_{K_n,Y^{(K_n)}}(j,{\underline{e}})+Y_e^{\scriptscriptstyle (K_n)} \right) \end{aligned}$$

adjoin the boundary edge $e_k$ to ${\bar{{\mathcal {S}}}}_{{\bar{\tau }}_{k-1}}^{\scriptscriptstyle (j_k)}$, where $(j_k,e_k)$ is the minimizer in (5.12). [As in (3.39), *e* is an edge from ${\underline{e}}\in {\bar{{\mathcal {S}}}}_{{\bar{\tau }}_{k-1}}^{\scriptscriptstyle (j)}$ to ${\overline{e}}\notin {\bar{{\mathcal {S}}}}_{{\bar{\tau }}_{k-1}}^{\scriptscriptstyle (j)}]$. Note that the arrival times in ${\bar{{\mathcal {S}}}}^{\scriptscriptstyle (j)}$ are given by

5.13 $$\begin{aligned} T^{\scriptscriptstyle {\bar{{\mathcal {S}}}}^{(j)}}({\underline{e}})=R_j^{-1}(d_{K_n,Y^{(K_n)}}(j,{\underline{e}})) \end{aligned}$$

by construction, so that we may rewrite (5.12) as

5.14 $$\begin{aligned} {\bar{\tau }}_k = \min _{j\in \left\{ 1,2\right\} }\min _{e\in \partial {\bar{{\mathcal {S}}}}_{{\bar{\tau }}_{k-1}}^{\scriptscriptstyle (j)}} R_j^{-1}\left( R_j\bigl ( T^{\scriptscriptstyle {\bar{{\mathcal {S}}}}^{(j)}}({\underline{e}}) \bigr )+Y_e^{\scriptscriptstyle (K_n)} \right) . \end{aligned}$$

Comparing (5.14) with (3.39), ${\mathcal {S}}$ and ${\bar{{\mathcal {S}}}}$ will evolve in the same way until the time

5.15 $$\begin{aligned} {\bar{\tau }}=\min \left\{ t:{\bar{{\mathcal {S}}}}^{\scriptscriptstyle (1)}_t\cap {\bar{{\mathcal {S}}}}^{\scriptscriptstyle (2)}_t\ne \varnothing \right\} \end{aligned}$$

when ${\bar{{\mathcal {S}}}}$ first accepts an edge between ${\bar{{\mathcal {S}}}}^{\scriptscriptstyle (1)}$ and ${\bar{{\mathcal {S}}}}^{\scriptscriptstyle (2)}$. In particular, the minimization problem in (5.14) will be the same as in (3.40), and the minimizer will be a.s. unique, as long as ${\bar{\tau }}_k\le {\bar{\tau }}$. Therefore we can choose $J,J'$ with $\left\{ J,J'\right\} =\left\{ 1,2\right\}$ and $I\in {\bar{{\mathcal {S}}}}^{\scriptscriptstyle (J)}_{{\bar{\tau }}-}, I'\in {\bar{{\mathcal {S}}}}^{\scriptscriptstyle (J')}_{{\bar{\tau }}-}$ such that, at time ${\bar{\tau }}$, the edge between *I* and $I'$ is adjoined to ${\bar{{\mathcal {S}}}}^{\scriptscriptstyle (J)}$. [In other words, $j=J$ and $e=\left\{ I,I'\right\}$ is the minimizer in (5.14)].

Because the minimizer in (5.14) is unique, no vertex is added to ${\mathcal {S}}$ at time ${\bar{\tau }}$. In particular, $T^{\scriptscriptstyle {\mathcal {S}}}(i)<{\bar{\tau }}$ for every $i\in {\mathcal {S}}_{{\bar{\tau }}}$. Since ${\mathcal {S}}^{\scriptscriptstyle (j)}_t$ and ${\bar{{\mathcal {S}}}}^{\scriptscriptstyle (j)}_t$ agree for $t<{\bar{\tau }}$, the arrival times before ${\bar{\tau }}$ must coincide. Recalling (5.13),

5.16 $$\begin{aligned} \begin{aligned}&T^{\scriptscriptstyle {\mathcal {S}}}(i)=T^{\scriptscriptstyle {\bar{{\mathcal {S}}}}^{(j)}}(i) = R_j^{-1}(d_{K_n,Y^{(K_n)}}(j,i)) \\&d_{K_n,Y^{(K_n)}}(j,i) = R_j(T^{\scriptscriptstyle {\mathcal {S}}}(i)) \end{aligned} \quad \text {if }i\in {\mathcal {S}}^{\scriptscriptstyle (j)}_{{\bar{\tau }}}. \end{aligned}$$

In addition, ${\mathcal {S}}^{\scriptscriptstyle (J')}_{{\bar{\tau }}}$ and ${\bar{{\mathcal {S}}}}^{\scriptscriptstyle (J')}_{{\bar{\tau }}}$ have the same vertex set while ${\mathcal {S}}^{\scriptscriptstyle (J)}_{{\bar{\tau }}}$ and ${\bar{{\mathcal {S}}}}^{\scriptscriptstyle (J)}_{{\bar{\tau }}}$ differ only by the vertex $I'$. It follows that

5.17 $$\begin{aligned} d_{K_n,Y^{(K_n)}}(j,i) \ge R_j(\tau ) \quad \text {if }i\notin {\mathcal {S}}^{\scriptscriptstyle (j)}_{{\bar{\tau }}}. \end{aligned}$$

Consider the optimal path from vertex *J* to vertex $I'$. Since $I'$ is adjoined to ${\bar{{\mathcal {S}}}}^{\scriptscriptstyle (J)}$ at time ${\bar{\tau }}$, it follows from (5.13) that $d_{K_n,Y^{(K_n)}}(J,I')=R_J({\bar{\tau }})$. Moreover, since *I* is the parent of $I'$ in ${\bar{{\mathcal {S}}}}^{\scriptscriptstyle (J)}_{{\bar{\tau }}}=\mathsf{SWT}^{\scriptscriptstyle (J)}_{R_J({\bar{\tau }})}$, we have $d_{K_n,Y^{(K_n)}}(J,I')=d_{K_n,Y^{(K_n)}}(J,I)+Y^{\scriptscriptstyle (K_n)}_{\left\{ I,I'\right\} }$. Applying (5.16) to the path from *J* to *I* to $I'$ to $J'$,

5.18 $$\begin{aligned} W_n&\le d_{K_n,Y^{(K_n)}}(J,I)+Y^{\scriptscriptstyle (K_n)}_{\left\{ I,I'\right\} } + d_{K_n,Y^{(K_n)}}(J',I') \nonumber \\&= R_J(T^{\scriptscriptstyle {\mathcal {S}}}(I))+Y^{\scriptscriptstyle (K_n)}_{\left\{ I,I'\right\} }+R_{J'}(T^{\scriptscriptstyle {\mathcal {S}}}(I'))\nonumber \\&=R_J(\tau )+R_{J'}(T^{\scriptscriptstyle {\mathcal {S}}}(I')) \le R_J({\bar{\tau }})+R_{J'}({\bar{\tau }}). \end{aligned}$$

In particular, both sides of (3.41) are bounded above by $R_1({\bar{\tau }})+R_2({\bar{\tau }})$.

The bound (5.18) will allow us to exclude vertices that arrive after time ${\bar{\tau }}$. To this end, we will show that (a) if a path $\pi$ from vertex 1 to vertex 2 contains a vertex not belonging to ${\mathcal {S}}_{{\bar{\tau }}}$, then the weight of $\pi$ is greater than $R_1({\bar{\tau }})+R_2({\bar{\tau }})$; and (b) if $i_1\in {\mathcal {S}}^{\scriptscriptstyle (1)}\setminus {\mathcal {S}}^{\scriptscriptstyle (1)}_{{\bar{\tau }}}$ or $i_2\in {\mathcal {S}}^{\scriptscriptstyle (2)}\setminus {\mathcal {S}}^{\scriptscriptstyle (2)}_{{\bar{\tau }}}$, then the term $R_1(T^{\scriptscriptstyle {\mathcal {S}}}(i_1)) + Y^{\scriptscriptstyle (K_n)}_{\left\{ i_1,i_2\right\} } + R_2(T^{\scriptscriptstyle {\mathcal {S}}}(i_2))$ in the minimum (3.41) is greater than $R_1({\bar{\tau }})+R_2({\bar{\tau }})$.

For (a), suppose that $\pi$ contains a vertex $i\notin {\mathcal {S}}_{{\bar{\tau }}}$. Since the vertex sets of ${\mathcal {S}}_{{\bar{\tau }}}$ and ${\bar{{\mathcal {S}}}}_{{\bar{\tau }}}$ coincide, it follows that $i\notin {\bar{{\mathcal {S}}}}_{{\bar{\tau }}}$, and by right continuity $i\notin {\bar{{\mathcal {S}}}}_t$ for some $t>{\bar{\tau }}$. Since $R_1+R_2$ is strictly increasing, (5.13) shows that the weight of $\pi$ is at least

5.19 $$\begin{aligned} d_{K_n,Y^{(K_n)}}(1,i)+d_{K_n,Y^{(K_n)}}(2,i) \ge R_1(t)+R_2(t)>R_1({\bar{\tau }})+R_2({\bar{\tau }}). \end{aligned}$$

For (b), suppose for specificity that $i_1\in {\mathcal {S}}^{\scriptscriptstyle (1)}\setminus {\mathcal {S}}^{\scriptscriptstyle (1)}_{{\bar{\tau }}}$. If in addition $i_2\in {\mathcal {S}}^{\scriptscriptstyle (2)}\setminus {\mathcal {S}}^{\scriptscriptstyle (2)}_{{\bar{\tau }}}$ then $T^{\scriptscriptstyle {\mathcal {S}}}(i_1),T^{\scriptscriptstyle {\mathcal {S}}}(i_2)>{\bar{\tau }}$ and the strict monotonicity of $R_1+R_2$ gives the desired result. We may therefore suppose $i_2\in {\mathcal {S}}^{\scriptscriptstyle (2)}_{{\bar{\tau }}}$. Since ${\mathcal {S}}^{\scriptscriptstyle (1)}$ and ${\mathcal {S}}^{\scriptscriptstyle (2)}$ are disjoint, we must have $i_1\notin {\mathcal {S}}^{\scriptscriptstyle (2)}_{{\bar{\tau }}}$, so that $d_{K_n,Y^{(K_n)}}(2,i_1)\ge R_2({\bar{\tau }})$. In particular, by considering the optimal path from 2 to $i_2$ together with the edge from $i_2$ to $i_1$, we conclude that $d_{K_n,Y^{(K_n)}}(2,i_2)+Y^{\scriptscriptstyle (K_n)}_{\left\{ i_1,i_2\right\} }\ge R_2({\bar{\tau }})$. By the assumption $i_2\in {\mathcal {S}}^{\scriptscriptstyle (2)}_{{\bar{\tau }}}$, we may rewrite this as $R_2(T^{\scriptscriptstyle {\mathcal {S}}}(i_2))+Y^{\scriptscriptstyle (K_n)}_{\left\{ i_1,i_2\right\} }\ge R_2({\bar{\tau }})$. Together with $R_1(T^{\scriptscriptstyle {\mathcal {S}}}(i_1))>R_1({\bar{\tau }})$, this proves (b).

To complete the proof, consider a path $\pi$ from vertex 1 to vertex 2. By statement (a) and (5.18), $\pi$ must contain only vertices from ${\mathcal {S}}_{{\bar{\tau }}}$ if it is to be optimal. Since $1\in {\mathcal {S}}^{\scriptscriptstyle (1)}_{{\bar{\tau }}}$ but $2\in {\mathcal {S}}^{\scriptscriptstyle (2)}_{{\bar{\tau }}}$, it follows that $\pi$ must contain an edge between some pair of vertices $i_1\in {\mathcal {S}}^{\scriptscriptstyle (1)}_{{\bar{\tau }}}$ and $i_2\in {\mathcal {S}}^{\scriptscriptstyle (2)}_{{\bar{\tau }}}$. The minimum possible weight of such a path is $d_{K_n,Y^{(K_n)}}(1,i_1)+Y^{\scriptscriptstyle (K_n)}_{\left\{ i_1,i_2\right\} }+d_{K_n,Y^{(K_n)}}(2,i_2)$, which agrees with the corresponding term in (3.41) by (5.16). Therefore (3.41) is verified if the minimum is taken only over $i_1\in {\mathcal {S}}^{\scriptscriptstyle (1)}_{{\bar{\tau }}}, i_2\in {\mathcal {S}}^{\scriptscriptstyle (2)}_{{\bar{\tau }}}$. But statement (b) and (5.18) shows that the remaining terms must be strictly greater. $\square$

#### Proof of Theorem 3.22

Since $R_1+R_2$ is strictly increasing, the relation $W_n=R_1(T_\mathrm{coll})+R_2(T_\mathrm{coll})$ is a reformulation of Definition 3.21.

Recall the time ${\bar{\tau }}$ from the proof of Lemma 3.20. We showed there that the minimizer of (3.41) must come from vertices $i_1\in {\mathcal {S}}^{\scriptscriptstyle (1)}_{{\bar{\tau }}}$, $i_2\in {\mathcal {S}}^{\scriptscriptstyle (2)}_{{\bar{\tau }}}$. In particular, by (5.16),

5.20 $$\begin{aligned} W_n=R_1\left( T^{\scriptscriptstyle {\mathcal {S}}}(I_1)\right) +Y^{\scriptscriptstyle (K_n)}_{\left\{ I_1,I_2\right\} }+R_2\left( T^{\scriptscriptstyle {\mathcal {S}}}(I_2)\right) \end{aligned}$$

expresses $W_n$ as the weight of the path formed as the union of the optimal path from 1 to $I_1$; the edge from $I_1$ to $I_2$; and the optimal path from $I_2$ to 2. In particular, $\pi _{1,2}$ is the same as this path. Since $T^{\scriptscriptstyle {\mathcal {S}}}(I_j)<\tau$ and ${\mathcal {S}}^{\scriptscriptstyle (j)}_t=\mathsf{SWT}^{\scriptscriptstyle (j)}_{R_j(t)}$ for $t<{\bar{\tau }}$, it follows that the optimal paths from *j* to $I_j$ coincide with the unique paths in ${\mathcal {S}}^{\scriptscriptstyle (j)}_{{\bar{\tau }}}$ between these vertices. The relation $H_n=H(I_1)+H(I_2)+1$ follows by counting the edges in these subpaths.

It remains to show that $T^{\scriptscriptstyle {\mathcal {S}}}(I_j)<T_\mathrm{coll}$. Define $t_1=R_1^{-1}(R_1(T^{\scriptscriptstyle {\mathcal {S}}}(I_1))+Y^{\scriptscriptstyle (K_n)}_{\left\{ I_1,I_2\right\} })$. Recalling (3.39), we see that $t_1$ is the time at which the edge from $I_1$ to $I_2$ is adjoined to ${\mathcal {S}}^{\scriptscriptstyle (1)}$, provided that $I_2$ has not already been added to ${\mathcal {S}}$ at some point strictly before time $t_1$. By construction, $I_2$ is added to ${\mathcal {S}}^{\scriptscriptstyle (2)}$, not ${\mathcal {S}}^{\scriptscriptstyle (1)}$, so it must be that $T^{\scriptscriptstyle {\mathcal {S}}}(I_2)<t_1$. [Equality is not possible because of our assumption that the minimizers of (3.39) are unique.] Aiming for a contradiction, suppose that $T^{\scriptscriptstyle {\mathcal {S}}}(I_2)\ge T_\mathrm{coll}$. Comparing the relation $W_n=R_1(T_\mathrm{coll})+R_2(T_\mathrm{coll})$ to (5.20) gives $R_1(T^{\scriptscriptstyle {\mathcal {S}}}(I_2))+Y^{\scriptscriptstyle (K_n)}_{\left\{ I_1,I_2\right\} }\le R_1(T_\mathrm{coll})$, so that $t_1\le T_\mathrm{coll}$. This is a contradiction since $t_1>T^{\scriptscriptstyle {\mathcal {S}}}(I_2)\ge T_\mathrm{coll}$. Similarly we must have $T^{\scriptscriptstyle {\mathcal {S}}}(I_1)< T_\mathrm{coll}$. This shows that the unique paths in ${\mathcal {S}}^{\scriptscriptstyle (j)}_\tau$ from *j* to $I_j$ are actually paths in ${\mathcal {S}}^{\scriptscriptstyle (j)}_{T_\mathrm{coll}}$, as claimed. $\square$

### ${\mathcal {B}}$ and ${\mathcal {S}}$ as Exploration Processes: Proof of Theorem 3.26

Before proving Theorem 3.26, we show that the discrete-time analogue of ${\mathcal {B}}$ is an exploration process determined by a minimal rule:

#### Lemma 5.7

Let $$v_k$$ denote the *k*th vertex added to $${\mathcal {B}},$$ excluding the root vertices $$\varnothing _1,\varnothing _2,$$ and set $${\mathcal {E}}_k={\mathcal {B}}_{T^{\mathcal {B}}_{v_k}}$$ for $$k\ge 1,$$$${\mathcal {E}}_0={\mathcal {B}}_0=\left\{ \varnothing _1,\varnothing _2\right\} .$$ Then $${\mathcal {E}}$$ is an exploration process determined by a minimal rule.

#### Proof

Consider the *k*th step and define

5.21 $$\begin{aligned} \tau _\mathrm{next}^{\scriptscriptstyle (j)}(k)=\min _{v\in \partial {\mathcal {E}}_{k-1}\cap {\mathcal {T}}^{\scriptscriptstyle (j)}} T_v, \end{aligned}$$

i.e., the next birth time of a vertex in ${\mathcal {T}}^{\scriptscriptstyle (j)}$ in the absence of freezing, and let $v_k^{\scriptscriptstyle (j)}$ denote the a.s. unique vertex attaining the minimum in (5.21). Recalling the definition of the filtration ${\mathscr {F}}_k$, we see that $\tau _\mathrm{next}^{\scriptscriptstyle (j)}(k)$ and $v_k^{\scriptscriptstyle (j)}$ are ${\mathscr {F}}_k$-measurable.

The variable $T_\mathrm{fr}^{\scriptscriptstyle (j)}$ is not ${\mathscr {F}}_k$-measurable. However, the event $\left\{ T_\mathrm{fr}^{\scriptscriptstyle (j)}<\tau _\mathrm{next}^{\scriptscriptstyle (j)}(k)\right\}$*is* ${\mathscr {F}}_k$-measurable. To see this, define

5.22 $$\begin{aligned} \tau _\mathrm{fr}^{\scriptscriptstyle (j)}(k) = \inf \bigg \{t\ge 0:\sum _{v\in {\mathcal {E}}_{k-1}\cap {\mathcal {T}}^{\scriptscriptstyle (j)}} {\mathbb {1}}_{\left\{ T_v\le t\right\} } \int _{t-T_v}^\infty {\mathrm e}^{-\lambda _n(1)(y-(t-T_v))} d\mu _n(y) \ge s_n\bigg \}, \end{aligned}$$

so that $\tau _\mathrm{fr}^{\scriptscriptstyle (j)}(k)$ is ${\mathscr {F}}_k$-measurable, and abbreviate $\tau _\mathrm{unfr}(k)=\tau _\mathrm{fr}^{\scriptscriptstyle (1)}(k)\vee \tau _\mathrm{fr}^{\scriptscriptstyle (2)}(k)$. We will use $\tau _\mathrm{fr}^{\scriptscriptstyle (j)}(k)$ as an approximation to $T_\mathrm{fr}^{\scriptscriptstyle (j)}$ based on the information available in ${\mathscr {F}}_k$. By analogy with (3.35) and (3.37), we also define

5.23 $$\begin{aligned} \begin{aligned} R_{j,k}(t)&= \left( t\wedge \tau _\mathrm{fr}^{\scriptscriptstyle (j)}(k) \right) + \left( (t-\tau _\mathrm{unfr}(k))\vee 0 \right) , \\ R_{j,k}^{-1}(t)&= {\left\{ \begin{array}{ll} t&{}\text {if }t\le \tau _\mathrm{fr}^{\scriptscriptstyle (j)}(k),\\ \tau _\mathrm{unfr}(k) -\tau _\mathrm{fr}^{\scriptscriptstyle (j)}(k)+t &{}\text {if }t>\tau _\mathrm{fr}^{\scriptscriptstyle (j)}(k). \end{array}\right. } \end{aligned} \end{aligned}$$

We note the following:

(i) $${\mathcal {E}}_{k-1}\cap {\mathcal {T}}^{\scriptscriptstyle (j)}=\mathsf{BP}^{\scriptscriptstyle (j)}_{t'}$$ and $$\tau _\mathrm{next}^{\scriptscriptstyle (j)}(k)=\min \left\{ T_v:v\in \partial \mathsf{BP}^{\scriptscriptstyle (j)}_{t'}\right\} $$, where $$t'=T_{v_{k-1}}$$.
(ii) The sum in (5.22) agrees with the sum in the definition (3.31) of $T_\mathrm{fr}^{\scriptscriptstyle (j)}$ whenever $t<\tau _\mathrm{next}^{\scriptscriptstyle (j)}(k)$.
(iii) $T_\mathrm{fr}^{\scriptscriptstyle (j)}<\tau _\mathrm{next}^{\scriptscriptstyle (j)}(k)$ if and only if $\tau _\mathrm{fr}^{\scriptscriptstyle (j)}(k)<\tau _\mathrm{next}^{\scriptscriptstyle (j)}(k)$, and in this case $T_\mathrm{fr}^{\scriptscriptstyle (j)}=\tau _\mathrm{fr}^{\scriptscriptstyle (j)}(k)$.
(iv) For $j\in \left\{ 1,2\right\}$, let $I_k^{\scriptscriptstyle (j)} \subseteq [0,\tau _\mathrm{next}^{\scriptscriptstyle (j)}(k)]$ be nonempty. The minimizers of $R_{j,k}^{-1}(t)$ and $R_j^{-1}(t)$ over all pairs (*t*, *j*) with $t \in I_k^{\scriptscriptstyle (j)}$ and $j \in \left\{ 1,2\right\}$ agree and return the same value.

Statement (i) follows by induction and (3.36). For (ii), note that the sums in (3.31) and (5.22) agree whenever $\mathsf{BP}_t^{\scriptscriptstyle (j)}\subset {\mathcal {E}}_{k-1}\cap {\mathcal {T}}^{\scriptscriptstyle (j)}$, so using (i) it suffices to show that $\mathsf{BP}_t^{\scriptscriptstyle (j)}\subset \mathsf{BP}^{\scriptscriptstyle (j)}_{t'}$ for $t<\tau _\mathrm{next}^{\scriptscriptstyle (j)}(k)$. But for any $t'$ and any $t<\min \left\{ T_v:v\in \partial \mathsf{BP}^{\scriptscriptstyle (j)}_{t'}\right\}$ we have $\mathsf{BP}^{\scriptscriptstyle (j)}_t\subset \mathsf{BP}^{\scriptscriptstyle (j)}_{t'}$ by definition, so the second part of (i) completes the proof.

Statement (iii) follows from (ii): if one of the sums in (3.31) or (5.22) exceeds $s_n$ before time $\tau _\mathrm{next}^{\scriptscriptstyle (j)}(k)$, then so does the other, and the first times where they do so are the same. In particular, $\left\{ T_\mathrm{fr}^{\scriptscriptstyle (j)} <\tau _\mathrm{next}^{\scriptscriptstyle (j)}(k)\right\}$ is ${\mathscr {F}}_k$-measurable because $\tau _\mathrm{fr}^{\scriptscriptstyle (j)}(k)$ and $\tau _\mathrm{next}^{\scriptscriptstyle (j)}(k)$ are.

To prove (iv), we distinguish three cases. If $\tau _\mathrm{fr}^{\scriptscriptstyle (j)}(k) \ge \tau _\mathrm{next}^{\scriptscriptstyle (j)}(k)$, then $R_{j,k}^{-1}$ and $R_j^{-1}$ reduce to the identity on $[0,\tau _\mathrm{next}^{\scriptscriptstyle (j)}(k)]$ by (iii). Hence, (iv) holds if $\tau _\mathrm{fr}^{\scriptscriptstyle (j)}(k) \ge \tau _\mathrm{next}^{\scriptscriptstyle (j)}(k)$ for both $j \in \left\{ 1,2\right\}$. If $\tau _\mathrm{fr}^{\scriptscriptstyle (j)}(k) < \tau _\mathrm{next}^{\scriptscriptstyle (j)}(k)$ for both $j \in \left\{ 1,2\right\}$, then $R_{j,k}^{-1}$ and $R_j^{-1}$ agree everywhere according to (iii). Finally, consider the case that $\tau _\mathrm{fr}^{\scriptscriptstyle (j)}(k) \ge \tau _\mathrm{next}^{\scriptscriptstyle (j)}(k)$ and $\tau _\mathrm{fr}^{\scriptscriptstyle (j')}(k) < \tau _\mathrm{next}^{\scriptscriptstyle (j')}(k)$ for $\left\{ j,j'\right\} =\left\{ 1,2\right\}$. Then $T_\mathrm{fr}^{\scriptscriptstyle (j)} \ge \tau _\mathrm{next}^{\scriptscriptstyle (j)}(k)$ and, therefore, $R_{j,k}^{-1}(t)=R_{j}^{-1}(t)=t$ on $I_k^{\scriptscriptstyle (j)}$. Moreover, $T_\mathrm{fr}^{\scriptscriptstyle (j')}=\tau _\mathrm{fr}^{\scriptscriptstyle (j')}(k)$ implying that $R_{j',k}^{-1}(t)=R_{j'}^{-1}(t)=t$ on $[0,\tau _\mathrm{fr}^{\scriptscriptstyle (j')}]$ and for $t \in \left( \tau _\mathrm{fr}^{\scriptscriptstyle (j')}(k),\tau _{\mathrm{next}}^{\scriptscriptstyle (j')}(k)\right]$, $R_{j',k}^{-1}(t)\ge \tau _\mathrm{unfr}(k) \ge \tau _\mathrm{next}^{\scriptscriptstyle (j)}(k)$ and $R_{j'}^{-1}(t)\ge T_\mathrm{unfr}\ge \tau _\mathrm{next}^{\scriptscriptstyle (j)}(k)$. Hence, in all three cases, the functions agree on the relevant domain and we have proved (iv).

Set $(v_k^{{\mathcal {E}}},j_k^{{\mathcal {E}}})$ to be the pair that minimizes $R_{j,k}^{-1}(T_v)$ among all pairs (*v*, *j*) with $v\in \partial {\mathcal {E}}_{k-1}\cap {\mathcal {T}}^{\scriptscriptstyle (j)}$, $j\in \left\{ 1,2\right\}$. Note that $R_{j,k}^{-1}(t)$ can be infinite when $\tau _\mathrm{fr}^{\scriptscriptstyle (j)}(k)<\infty$ and $\tau _\mathrm{fr}^{\scriptscriptstyle (j')}(k)=\infty$, where $\left\{ j,j'\right\} =\left\{ 1,2\right\}$, but in this case $R_{j',k}^{-1}(t)$ must be finite for all *t*. Furthermore $R_{j,k}^{-1}$ is strictly increasing whenever it is finite. Recalling that the times $T_v$ are formed from variables with continuous distributions, it follows that the minimizing pair $(v_k^{\mathcal {E}},j_k^{\mathcal {E}})$ is well defined a.s.

Since $R_{j,k}^{-1}$ is ${\mathscr {F}}_k$-measurable, the choice of $v_k^{\mathcal {E}}$ is determined by a minimal rule with $S_k=\partial {\mathcal {E}}_{k-1}$. To complete the proof, we must show that $v_k^{\mathcal {E}}=v_k$.

The vertex $v_k$ is the first coordinate of the pair $(v_k,j_k)$ that minimizes $T^{\mathcal {B}}_v=R_j^{-1}(T_v)$ over all pairs (*v*, *j*) with $v\in \partial {\mathcal {B}}^{\scriptscriptstyle (j)}_{T^{\mathcal {B}}_{v_{k-1}}}$, $j\in \left\{ 1,2\right\}$. (Once again, this minimizing pair is well defined a.s., reflecting the fact that, a.s., ${\mathcal {B}}$ never adds more than one vertex at a time.) Since ${\mathcal {E}}_{k-1}={\mathcal {B}}_{T^{\mathcal {B}}_{v_{k-1}}}$, the pairs $(v_k^{\mathcal {E}},j_k^{\mathcal {E}})$ and $(v_k,j_k)$ are both minimizers over the set $\left\{ (v,j):v\in \partial {\mathcal {E}}_{k-1}\cap {\mathcal {T}}^{\scriptscriptstyle (j)}\right\}$. Moreover, since $R_j^{-1}$ and $R_{j,k}^{-1}$ are both strictly increasing (when finite), both minimizations can be restricted to the set $\left\{ (v_k^{\scriptscriptstyle (j)},j):j=1,2\right\}$, where $v_k^{\scriptscriptstyle (j)}$ is the minimizer in (5.21). Since $T_{v_k^{(j)}}=\tau _\mathrm{next}^{\scriptscriptstyle (j)}(k)$, statement (iv) with $I_{j,k}=\left\{ \tau _\mathrm{next}^{\scriptscriptstyle (j)}(k)\right\}$ implies $v_k^{\mathcal {E}}=v_k$, as claimed. $\square$

#### Proof of Proposition 3.25

By Theorem 5.3, the edge weights $X^{\scriptscriptstyle (K_n)}_e$ associated via (5.4) to the exploration process in Lemma 5.7 are independent exponential random variables with mean 1. Recalling (2.1)–(2.2), the corresponding edge weights $Y^{\scriptscriptstyle (K_n)}_e=g(X^{\scriptscriptstyle (K_n)}_e)$ are independent with distribution function $F_{\scriptscriptstyle Y}$. To complete the proof it suffices to observe that $N(i)<N(i')$ if and only if $T^{{\widetilde{{\mathcal {B}}}}}(i)<T^{{\widetilde{{\mathcal {B}}}}}(i')$ and that $T^{{\widetilde{{\mathcal {B}}}}}(i)$ is finite for all $i\in [n]$ since the FPP process explores every edge in ${\mathcal {T}}^{\scriptscriptstyle (1,2)}$ eventually. Hence, definitions (3.46) and (5.4) agree. $\square$

#### Proof of Theorem 3.26

We resume the notation from the proof of Lemma 5.7. Create the edge weights on $K_n$ according to (5.4). Denote by $\tau _{k'-1}$ the time where the $(k'-1)$th vertex (not including the vertices 1 and 2) was added to ${\mathcal {S}}$ [see (3.39)]. As in the proof of Theorem 3.8, both ${\widetilde{{\mathcal {B}}}}$ and ${\mathcal {S}}$ are increasing jump processes and $\pi _M({\widetilde{{\mathcal {B}}}}_0)={\mathcal {S}}_0$. By an inductive argument we can suppose that $k, k'\in {\mathbb {N}}$ are such that ${\widetilde{{\mathcal {E}}}}_k\ne {\widetilde{{\mathcal {E}}}}_{k-1}$ and $\pi _M({\widetilde{{\mathcal {E}}}}_{k-1})={\mathcal {S}}_{\tau _{k'-1}}$. The proof will be complete if we can prove that (a) the edge $e_{k'}'$ adjoined to ${\mathcal {S}}_{\tau _{k'-1}}$ to form ${\mathcal {S}}_{\tau _{k'}}$ is the same as the edge $e_k=\left\{ i_k,i_k'\right\}$ adjoined to $\pi _M({\widetilde{{\mathcal {E}}}}_{k-1})$ to form $\pi _M({\widetilde{{\mathcal {E}}}}_k)$; and (b) $\tau _{k'}=T^{\mathcal {B}}_{v_k}$.

Let $i\in {\mathcal {S}}_{\tau _{k'-1}}$, and let $j\in \left\{ 1,2\right\}$ be such that $i\in {\mathcal {S}}^{\scriptscriptstyle (j)}_{\tau _{k'-1}}$. By the inductive hypothesis, $V(i)\in {\widetilde{{\mathcal {E}}}}_{k-1}\cap {\mathcal {T}}^{\scriptscriptstyle (j)}$ and the unique path in ${\mathcal {S}}_{\tau _{k'-1}}$ from *i* to *j* is the image of the unique path in ${\widetilde{{\mathcal {E}}}}_{k-1}$ from *V*(*i*) to $\varnothing _j$ under the mapping $v\mapsto M_v$ (recall Definition 3.7). According to (5.9), (2.1) and (2.3), the edge weights along this path are $Y^{\scriptscriptstyle (K_n)}_{\{M_{p^{m-1}\!\left( V(i)\right) },M_{p^{m}\!\left( V(i)\right) }\}}=g(X^{\scriptscriptstyle (K_n)}_{\{M_{p^{m-1}\!\left( V(i)\right) },M_{p^{m}\!\left( V(i)\right) }\}})=g\left( \tfrac{1}{n}X_{p^{m-1}\!\left( V(i)\right) }\right)$ and $f_n(X_{p^{m-1}\!\left( V(i)\right) })$, for $m=1,\dotsc ,\left| V(i)\right|$. Summing gives $d_{{\mathcal {S}}_{\tau _{k'-1}},Y^{\scriptscriptstyle (K_n)}}(j,i)=T_{V(i)}$.

In addition, let $i'\notin {\mathcal {S}}_{\tau _{k'-1}}$ and write $e=\left\{ i,i'\right\}$. By (5.10), $X_e^{\scriptscriptstyle (K_n)}=\tfrac{1}{n}X_{V(i,i')}$, so that $Y_e^{\scriptscriptstyle (K_n)}=f_n(X_{V(i,i')})$. Thus the expression in the right-hand side of (3.39) reduces to $R_j^{-1}\left( T_{V(i)}+f_n(X_{V(i,i')}) \right)$, i.e., $R_j^{-1}\left( T_{V(i,i')} \right)$. The edge $e_{k'}'$ minimizes this expression over all $i\in {\mathcal {S}}_{\tau _{k'-1}}$, $i'\notin {\mathcal {S}}_{\tau _{k'-1}}$. By Proposition 5.6, the edge $e_k=\left\{ i_k,i_k'\right\}$ minimizes $R_{j,k}^{-1}(T_{V(i,i')})$ over all $i\in \pi _M({\widetilde{{\mathcal {E}}}}_{k-1})$, $i'\notin \pi _M({\widetilde{{\mathcal {E}}}}_{k-1})$ (with *j* such that $V(i)\in {\mathcal {E}}^{\scriptscriptstyle (j)}_{k-1}$). By the induction hypothesis, statement (iv) in the proof of Lemma 5.7 and monotonicity, these two minimization problems have the same minimizers, proving (a), and return the same value, which completes the proof of (b). $\square$

### Coupling and Cox Processes: Proof of Theorem 3.27

In this section we explain the modified coupling in, and give the proof of Theorem 3.27.

#### Proof of Theorem 3.27

The edge-weight coupling (3.46) selects the edge weights $Y^{\scriptscriptstyle (K_n)}_{\left\{ i_1,i_2\right\} }$ for $i_1\in {\mathcal {S}}^{\scriptscriptstyle (1)},i_2\in {\mathcal {S}}^{\scriptscriptstyle (2)}$ based on values $f_n(X_w)$ for which $p\left( w\right) \in {\widetilde{\mathsf{BP}}}$ and $\left\{ M_{p\left( w\right) },M_w\right\} =\left\{ i_1,i_2\right\}$. Under the present definition of ${\mathcal {B}}$ [see (3.36)], such vertices *w* are eventually explored, and consequently the values $f_n(X_w)$ can be recovered by observing $({\mathcal {B}}_t)_{t\ge 0}$. On the other hand, in the context of Theorem 3.27, we want the values $f_n(X_w)$ to behave as a Cox process [with respect to ${\mathcal {B}},R_1,R_2,(M_v)_{v\in {\mathcal {B}}}$]. For this reason, we will modify the definition of ${\mathcal {B}}$ so that it does not explore vertices *w* of this kind, and we will replace the contribution of those vertices using an additional source of randomness.^2^

As always, we have two independent PWITs $({\mathcal {T}}^{\scriptscriptstyle (j)},X^{\scriptscriptstyle (j)})$, $j\in \left\{ 1,2\right\}$, the marks $M_v$, $v \in {\mathcal {T}}^{\scriptscriptstyle (1,2)}$, and a family of independent exponential random variables $E_e$, $e\in E(K_{\infty })$, with mean 1, independent of the PWITs and the marks. In addition, from each vertex *v* we initialise an independent PWIT with vertices $(v,w')$, edge weights $X'_{(v,w')}$ [such that $(X'_{(v,w'k)})_{k=1}^\infty$ forms a Poisson point process with rate 1 on $(0,\infty )$] and marks $M'_{(v,w')}$ uniform on [*n*], all independent of each other and of the original variables $X_v,M_v$.

First consider $({\mathcal {B}},{\widetilde{{\mathcal {B}}}},R_1,R_2)$ as normally constructed, without using the auxiliary PWITs with vertices $(v,w')$. Fix $i,i'\in [n]$, $i\ne i'$, and suppose for definiteness that $T^{\scriptscriptstyle {\widetilde{{\mathcal {B}}}}}(i)< T^{\scriptscriptstyle {\widetilde{{\mathcal {B}}}}}(i')$. [If instead $T^{\scriptscriptstyle {\widetilde{{\mathcal {B}}}}}(i')< T^{\scriptscriptstyle {\widetilde{{\mathcal {B}}}}}(i)$, interchange the roles of *i* and $i'$ in the following discussion.] According to (3.46) or (5.4), the edge weight $X^{\scriptscriptstyle (K_n)}_{\left\{ i,i'\right\} }$ is set to be $\tfrac{1}{n}X(i,i')$, where $X(i,i')$ is the first point in the Poisson point process

5.24 $$\begin{aligned} {\mathcal {P}}^{\scriptscriptstyle (i,i')}=\sum _{w:p\left( w\right) =V(i), M_w=i'} \delta _{X_w} \end{aligned}$$

of intensity 1/*n*.

Now condition on *V*(*i*) and $V(i')$ belonging to different trees, say $V(i)\in {\mathcal {T}}^{\scriptscriptstyle (J)}, V(i')\in {\mathcal {T}}^{\scriptscriptstyle (J')}$ where $\left\{ J,J'\right\} =\left\{ 1,2\right\}$. For this to happen, the children of *V*(*i*) having mark $i'$ must *not* have been explored by time $T^{\scriptscriptstyle {\mathcal {B}}}_{V(i')}$. A child *w* of *V*(*i*) is explored at time $R_J^{-1}(T_w)=R_J^{-1}(T_{V(i)}+f_n(X_w))$, so we can reformulate this by saying that the Poisson point process ${\mathcal {P}}^{\scriptscriptstyle (i,i')}$ must contain no point *x* with $R_J^{-1}(T_{V(i)}+f_n(x)) \le T_{V(i')}^{\scriptscriptstyle {\mathcal {B}}}$. Using the relation $T_v=R_j(T^{\scriptscriptstyle {\mathcal {B}}}_v)$ for $v\in {\mathcal {T}}^{\scriptscriptstyle (j)}$, we can rewrite this as the condition that ${\mathcal {P}}^{\scriptscriptstyle (i,i')}$ contains no point *x* with $f_n(x)\le R_J(T_{V(i')}^{\scriptscriptstyle {\mathcal {B}}})-R_J(T_{V(i)}^{\scriptscriptstyle {\mathcal {B}}})=\Delta R_{V(i),V(i')}$. However, the condition gives no information about points of larger value. It follows that, conditionally on $({\mathcal {B}}_t,{\widetilde{{\mathcal {B}}}}_t,R_1(t),R_2(t))_{t\le T^{\scriptscriptstyle {\widetilde{{\mathcal {B}}}}}(i')}$, ${\mathcal {P}}^{\scriptscriptstyle (i,i')}$ is a Poisson point process of intensity 1/*n* on $(f_n^{-1}(\Delta R_{V(i),V(i')}),\infty )$.

To preserve this property when conditioning on $({\mathcal {B}}_t,R_1(t),R_2(t),(M_v)_{v\in {\mathcal {B}}_t})$ for $t> T^{\scriptscriptstyle {\widetilde{{\mathcal {B}}}}}(i')$, replace the edge weights and marks of all vertices *w* (and their descendants) for which $p\left( w\right) =V(i)$, $M_w=i'$ and $f_n(X_w)>\Delta R_{V(i),V(i')}$, by the edge weights and marks of the auxiliary vertices $(V(i),w')$ (and their descendants) for which $p\left( w'\right) =V(i)$, $M'_{(V(i),w')}=i'$ and $f_n(X'_{(V(i),w')})>\Delta R_{V(i),V(i')}$. Modify the evolution of ${\mathcal {B}},R_1,R_2$ for $t>T^{\scriptscriptstyle {\widetilde{{\mathcal {B}}}}}(i')$ so as to use the replacement edge weights $X'_{v,w'}$, but continue to use the original edge weights $X_w$ in the edge-weight coupling (5.4). [Formally, modify the minimal rule from Lemma 5.7 so that vertices are ineligible for exploring once they have been replaced, but add to the sum in (5.22) the contribution from any replacement vertices from the auxiliary PWITs that would have been explored at time *t*.]

These replacements do not change the law of ${\mathcal {B}},R_1,R_2,(M_v)_{v\in {\mathcal {B}}}$, or the edge weights $X^{\scriptscriptstyle (K_n)}_e$. The pointwise equality between $\pi _M({\widetilde{{\mathcal {B}}}})$ and ${\mathcal {S}}$ is unaffected: the replaced vertices are thinned and therefore do not affect ${\widetilde{{\mathcal {B}}}}$. Finally, the evolution of ${\widetilde{{\mathcal {B}}}}$ for $t>T^{\scriptscriptstyle {\widetilde{{\mathcal {B}}}}}(i_2)$ now gives no additional information about the edge weights $\left\{ X_w:p\left( w\right) =V(i_1), M_w=i_2, f_n(X_w)>\Delta R_{V(i),V(i')}\right\}$. In particular, conditionally on ${\mathcal {B}},R_1,R_2,(M_v)_{v\in {\mathcal {B}}}$ and the event that $V(i)\in {\mathcal {T}}^{\scriptscriptstyle (J)}, V(i')\in {\mathcal {T}}^{\scriptscriptstyle (J')}$ for some choice of $\left\{ J,J'\right\} =\left\{ 1,2\right\}$, the law of ${\mathcal {P}}^{\scriptscriptstyle (i,i')}$ is that of a Poisson point process with intensity measure 1/*n* on $(f_n^{-1}(\Delta R_{V(i),V(i')}),\infty )$. Furthermore, the Poisson point processes corresponding to different $i,i'$ will be conditionally independent.

We can now give an explicit construction of ${\mathcal {P}}_n$. We begin by defining ${\mathcal {P}}_n$ on the subspace given by unthinned pairs of vertices:

5.25 $$\begin{aligned}&\text {for }i_1,i_2\in [n]\quad \text {such that }V(i_1)\in {\mathcal {T}}^{\scriptscriptstyle (1)},\quad V(i_2)\in {\mathcal {T}}^{\scriptscriptstyle (2)}, \nonumber \\ {}&\quad \text {and for } \left\{ j,j'\right\} =\left\{ 1,2\right\} \quad \text {with } T^{\scriptscriptstyle {\mathcal {B}}}_{V(i_j)}<T^{\scriptscriptstyle {\mathcal {B}}}_{V(i_{j'})}, \nonumber \\&\quad \left. {\mathcal {P}}_n \big .\right| _{\left[ 0,\infty \right) \times \left\{ V(i_1)\right\} \times \left\{ V(i_2)\right\} } = \sum _{\begin{array}{c} w:p\left( w\right) =V(i_j),\\ M_w=i_{j'} \end{array}} \delta _{\left( (R_1+R_2)^{-1}\left( T_{V(i_1)}+f_n(X_w)+T_{V(i_2)}\right) , V(i_1), V(i_2)\right) }. \end{aligned}$$

In the notation above, $\left. {\mathcal {P}}_n \big .\right| _{\left[ 0,\infty \right) \times \left\{ V(i_1)\right\} \times \left\{ V(i_2)\right\} }$ is the image of ${\mathcal {P}}^{\scriptscriptstyle (i_j,i_{j'})}$ under the mapping

5.26 $$\begin{aligned} x\mapsto (R_1+R_2)^{-1}(T_{V(i_1)}+f_n(x)+T_{V(i_2)}). \end{aligned}$$

In particular, by the remarks above, $\left. {\mathcal {P}}_n\big .\right| _{\left[ 0,\infty \right) \times {\widetilde{{\mathcal {B}}}}^{\scriptscriptstyle (1)}\times {\widetilde{{\mathcal {B}}}}^{\scriptscriptstyle (2)}}$ has the conditional law of a Poisson point process conditionally on ${\mathcal {B}},R_1,R_2,(M_v)_{v\in {\mathcal {B}}}$. To compute its intensity measure, note that the mapping $x\mapsto y=f_n(x)$ sends 1/*n* times Lebesgue measure on $(f_n^{-1}(\Delta R_{V(i),V(i')}),\infty )$ to the measure $\tfrac{1}{n}\left. \mu _n\big .\right| _{(\Delta R_{V(i),V(i')}, \infty )}$. It follows that the further mapping $y\mapsto (R_1+R_2)^{-1}(T_{V(i_1)}+y+T_{V(i_2)})$ leads to the intensity measure specified by (3.48), where we have again used the relation $T_v=R_j(T^{\scriptscriptstyle {\mathcal {B}}}_v)$ for $v\in {\mathcal {T}}^{\scriptscriptstyle (j)}$. Thus $\left. {\mathcal {P}}_n\big .\right| _{\left[ 0,\infty \right) \times {\widetilde{{\mathcal {B}}}}^{\scriptscriptstyle (1)}\times {\widetilde{{\mathcal {B}}}}^{\scriptscriptstyle (2)}}$ is a Cox process of the correct intensity.

Finally, we may extend ${\mathcal {P}}_n$ to be a Cox process on $\left[ 0,\infty \right) \times {\mathcal {T}}^{\scriptscriptstyle (1)}\times {\mathcal {T}}^{\scriptscriptstyle (2)}$ with the specified intensity, by defining $\left. {\mathcal {P}}_n\big .\right| _{\left[ 0,\infty \right) \times \left\{ v_1\right\} \times \left\{ v_2\right\} }$ using an independent source of randomness for any pair of vertices $v_1,v_2$ for which $v_1\in {\mathcal {B}}^{\scriptscriptstyle (1)}\setminus {\widetilde{{\mathcal {B}}}}^{\scriptscriptstyle (1)}$ or $v_2\in {\mathcal {B}}^{\scriptscriptstyle (2)}\setminus {\widetilde{{\mathcal {B}}}}^{\scriptscriptstyle (2)}$. Note that the details of this extension are unimportant since such pairs $(v_1,v_2)$ are not considered in the definition of $(T_\mathrm{coll}^{\scriptscriptstyle ({\mathcal {P}}_n)},V_\mathrm{coll}^{\scriptscriptstyle (1)},V_\mathrm{coll}^{\scriptscriptstyle (2)})$.

Observe that under this construction of ${\mathcal {P}}_n$ and under the edge-weight coupling (3.46),

5.27 $$\begin{aligned} (T_\mathrm{coll}^{\scriptscriptstyle ({\mathcal {P}}_n)},V_\mathrm{coll}^{\scriptscriptstyle (1)},V_\mathrm{coll}^{\scriptscriptstyle (2)}) = (T_\mathrm{coll}, V(I_1), V(I_2)). \end{aligned}$$

Indeed, consider any $i_1,i_2\in [n]$ with $V(i_1)\in {\mathcal {T}}^{\scriptscriptstyle (1)}$ and $V(i_2)\in {\mathcal {T}}^{\scriptscriptstyle (2)}$. We note that this assumption is equivalent to $i_1\in {\mathcal {S}}^{\scriptscriptstyle (1)}$ and $i_2\in {\mathcal {S}}^{\scriptscriptstyle (2)}$. Taking $\left\{ J,J'\right\} =\left\{ 1,2\right\}$ with $T^{\mathcal {B}}_{V(i_J)}<T^{\mathcal {B}}_{V(i_{J'})}$, (3.46) gives

5.28 $$\begin{aligned} Y^{\scriptscriptstyle (K_n)}_{\left\{ i_1,i_2\right\} } = g\left( X^{\scriptscriptstyle (K_n)}_{\left\{ i_1,i_2\right\} }\right) = g\left( \tfrac{1}{n}X(i_J,i_{J'})\right) = f_n(X(i_J,i_{J'})). \end{aligned}$$

The value $X(i_J,i_{J'})$ coincides with the first point of ${\mathcal {P}}^{\scriptscriptstyle (i_J,i_{J'})}$, and applying the increasing mapping (5.26) it follows that the first point of $\left. {\mathcal {P}}_n \big .\right| _{\left[ 0,\infty \right) \times \left\{ V(i_1)\right\} \times \left\{ V(i_2)\right\} }$ has time coordinate

5.29 $$\begin{aligned} (R_1+R_2)^{-1}\left( T_{V(i_1)}+Y^{\scriptscriptstyle (K_n)}_{\left\{ i_1,i_2\right\} }+T_{V(i_2)} \right) . \end{aligned}$$

Using Lemma 3.20, the strict monotonicity of $R_1+R_2$, and the relation $T_{V(i)}=R_j(T^{\mathcal {B}}_{V(i)})=R_j(T^{\scriptscriptstyle {\mathcal {S}}}(i))$ for $V(i)\in {\mathcal {T}}^{\scriptscriptstyle (j)}$, $i\in {\mathcal {S}}^{\scriptscriptstyle (j)}$, we see that

5.30 $$\begin{aligned} T_\mathrm{coll}&= (R_1+R_2)^{-1}(W_n) = (R_1+R_2)^{-1}\left( \min _{i_1\in {\mathcal {S}}^{\scriptscriptstyle (1)},i_2\in {\mathcal {S}}^{\scriptscriptstyle (2)}} T_{V(i_1)}+Y^{\scriptscriptstyle (K_n)}_{\left\{ i_1,i_2\right\} }+T_{V(i_2)} \right) \nonumber \\&= \min _{i_1\in {\mathcal {S}}^{\scriptscriptstyle (1)},i_2\in {\mathcal {S}}^{\scriptscriptstyle (2)}} (R_1+R_2)^{-1}\left( T_{V(i_1)}+Y^{\scriptscriptstyle (K_n)}_{\left\{ i_1,i_2\right\} }+T_{V(i_2)} \right) \end{aligned}$$

is the result of minimizing (5.29) over all choices of $i_1,i_2$, and $I_1,I_2$ are the corresponding minimizers. On the other hand, $T_\mathrm{coll}^{\scriptscriptstyle ({\mathcal {P}}_n)}$ is the result of minimizing the first point of $\left. {\mathcal {P}}_n \big .\right| _{\left[ 0,\infty \right) \times \left\{ v_1\right\} \times \left\{ v_2\right\} }$ over all choices of unthinned vertices $v_1\in {\mathcal {T}}^{\scriptscriptstyle (1)},v_2\in {\mathcal {T}}^{\scriptscriptstyle (2)}$, and $V_\mathrm{coll}^{\scriptscriptstyle (1)},V_\mathrm{coll}^{\scriptscriptstyle (2)}$ are the corresponding minimizers. Every such pair $(v_1,v_2)$ can be written as $v_j=V(i_j)$ for some $i_j\in {\mathcal {S}}^{\scriptscriptstyle (j)}$, $j=1,2$, and in fact $i_j=M_{v_j}$ in this correspondence. Hence these two minimizations problems are equivalent and their unique minimizers coincide, and we have proved (5.27).

The remaining statements in Theorem 3.27 follow from (5.27) and the relations $W_n=R_1(T_\mathrm{coll})+R_2(T_\mathrm{coll})$, ${\mathcal {S}}_t=\pi _M({\widetilde{{\mathcal {B}}}}_t)$ for all *t*, and $M_{V(i)}=i, V(M_v)=v$ for $i\in {\mathcal {S}},v\in {\widetilde{{\mathcal {B}}}}$. $\square$

In the remainder of the paper, we will be concerned only with the equality in law from Theorem 3.27. We can therefore continue to define ${\mathcal {B}}$ as in (3.36), ignoring the modified construction given in the proof of Theorem 3.27. The edge-weight coupling (3.46) between ${\mathcal {T}}^{\scriptscriptstyle (1,2)}$ and $K_n$, and indeed the edge weights on $K_n$ generally, will play no further direct role in the analysis.

## Branching Processes and Random Walks

In this section, we prove Theorem 3.13 by continuing the analysis of the branching process $\mathsf{BP}^{\scriptscriptstyle (1)}$ introduced in Sect. 3. In Sect. 6.1 we identify a random walk which facilitates moment computations of the one-vertex characteristics. Section 6.2 contains precise results about the scaling behavior of the random walk and the parameter $\lambda _n(a)$. The results are proved in Sect. 6.3. Section 6.4 identifies the asymptotics of the first two moments of one-vertex characteristics. Having understood these, we investigate two-vertex characteristics in Sect. 7 and prove Theorem 3.15.

### Continuous-Time Branching Processes and Random Walks

Recall that $\mathsf{BP}^{\scriptscriptstyle (1)}=(\mathsf{BP}_t^{\scriptscriptstyle (1)})_{t\ge 0}$ denotes a CTBP with original ancestor $\varnothing _1$. Using Ulam–Harris notation, the children of the root are the vertices *v* with $p\left( v\right) =\varnothing _1$, and their birth times $(T_v)_{p\left( v\right) =\varnothing _1}$ form a Poisson point process with intensity $\mu _n$. For $v \in {\mathcal {T}}^{\scriptscriptstyle (1)}$, write $\mathsf{BP}^{\scriptscriptstyle (v)}$ for the branching process of descendants of such a *v*, re-rooted and time-shifted to start at $t=0$. Formally,

6.1 $$\begin{aligned} \mathsf{BP}^{\scriptscriptstyle (v)}_t=\left\{ w\in {\mathcal {T}}^{\scriptscriptstyle (1)}:vw\in \mathsf{BP}^{\scriptscriptstyle (1)}_{T_v+t}\right\} . \end{aligned}$$

In particular, $\mathsf{BP}^{\scriptscriptstyle (1)}=\mathsf{BP}^{\scriptscriptstyle (\varnothing _1)}$, and the processes $(\mathsf{BP}^{\scriptscriptstyle (v)})_{p\left( v\right) =\varnothing _1}$ are independent of each other and of $(T_v)_{p\left( v\right) =\varnothing _1}$. We may express this compactly by saying that the sum of point masses ${\mathcal {Q}}=\sum _{p\left( v\right) =\varnothing _1} \delta _{(T_v,\mathsf{BP}^{\scriptscriptstyle (v)})}$ forms a Poisson point process with intensity $d\mu _n \otimes d{\mathbb {P}}(\mathsf{BP}^{\scriptscriptstyle (1)}\in \cdot )$, where ${\mathbb {P}}(\mathsf{BP}^{\scriptscriptstyle (1)}\in \cdot )$ is the law of the entire branching process. Recalling the definition of the one-vertex characteristic from (3.14), we deduce that

6.2 $$\begin{aligned} \begin{aligned} z_t^\chi (a)&= \chi (t)+a\sum _{v:p\left( v\right) =\varnothing } {\mathbb {1}}_{\left\{ T_v\le t\right\} } z_{t-T_v}^{\chi , \mathsf{BP}^{\scriptscriptstyle (v)}}(a) \\&= \chi (t)+a\int d{\mathcal {Q}}(y,bp) {\mathbb {1}}_{\left\{ y\le t\right\} } z_{t-y}^{\chi , bp}(a). \end{aligned} \end{aligned}$$

Note that (6.2) holds jointly for all $a,t,\chi$. To draw conclusions for $m_t^\chi (a)$ and $M_{t,u}^{\chi ,\eta }(a,b)$, the expectation of $z_t^\chi (a)$ and $z_t^\chi (a)z_u^{\eta }(b)$, respectively, defined in (3.15), we will use the formulas

6.3 $$\begin{aligned} \begin{aligned} {\mathbb {E}}\Big ( \sum _{p\in \tilde{{\mathcal {Q}}}} f(p) \Big )&= \int f(p) \, d{\tilde{\mu }}(p), \\ {{\,\mathrm{Cov}\,}}\Big ( \sum _{p\in \tilde{{\mathcal {Q}}}} f_1(p),\sum _{p\in \tilde{{\mathcal {Q}}}} f_2(p) \Big )&= \int f_1(p) f_2(p) \, d{\tilde{\mu }}(p), \end{aligned} \end{aligned}$$

where $\tilde{{\mathcal {Q}}}$ is a Poisson point process with some intensity ${\tilde{\mu }}$ (and assuming the integrals exist). Apply (6.3) to (6.2) with $f(y,bp)={\mathbb {1}}_{\left\{ y\le t\right\} } z_{t-y}^{\chi , bp}(a)$ to get

6.4 $$\begin{aligned} m_t^\chi (a)&= \chi (t)+a\int d\mu _n(y){\mathbb {1}}_{\left\{ y\le t\right\} } \int d{\mathbb {P}}(\mathsf{BP}^{\scriptscriptstyle (1)}=bp) z_{t-y}^{\chi ,bp}(a) \nonumber \\&= \chi (t)+a\int _0^t d\mu _n(y) m_{t-y}^\chi (a). \end{aligned}$$

Similarly

6.5 $$\begin{aligned} M_{t,u}^{\chi ,\eta }(a,b)-m_t^\chi (a) m_u^\eta (b)&= ab\int d\mu _n(y){\mathbb {1}}_{\left\{ y\le t\right\} }{\mathbb {1}}_{\left\{ y\le u\right\} } \int d{\mathbb {P}}(\mathsf{BP}^{\scriptscriptstyle (1)}=bp) z_{t-y}^{\chi ,bp}(a) z_{u-y}^{\eta ,bp}(b) \nonumber \\&= ab\int _0^{t\wedge u} d\mu _n(y) M_{t-y,u-y}^{\chi ,\eta }(a,b) . \end{aligned}$$

Recall from (3.16) that ${\hat{\mu }}_n(\lambda )=\int {\mathrm e}^{-\lambda t}d\mu _n(t)$ denotes the Laplace transform of $\mu _n$ and that for $a>0$ the parameter $\lambda _n(a)>0$ is the unique solution to $a{\hat{\mu }}_n(\lambda _n(a))=1$. [In general, if ${\hat{\mu }}_n(\lambda _0)$ is finite for some $\lambda _0\ge 0$, then the equation has a solution whenever $1/{\hat{\mu }}_n(\lambda _0)\le a<1/\mu _n(\left\{ 0\right\} )$, and this solution is unique if $\mu _n$ assigns positive mass to $(0,\infty )$. Our assumptions imply that, for *n* sufficiently large, $\lambda _n(a)$ exists uniquely for any $a>0$.] Since $z_t^\chi (a)$ typically grows exponentially at rate $\lambda _n(a)$, we study the rescaled versions ${\bar{m}}_t^\chi (a)$, ${\bar{M}}_{t,u}^{\chi ,\eta }(a,b)$ defined in (3.19) and let

6.6 $$\begin{aligned} d\nu _a(y)= a {\mathrm e}^{-\lambda _n(a) y} d\mu _n(y). \end{aligned}$$

Then (6.4) becomes ${\bar{m}}_t^\chi (a)={\mathrm e}^{-\lambda (a)t}\chi (t)+\int _0^t d\nu _a(y) {\bar{m}}_{t-y}^\chi (a)$. Since $\nu _a$ is a probability measure by construction, this recursion can be solved in terms of a random walk:

6.7 $$\begin{aligned} {\bar{m}}_t^\chi (a) = {\mathbb {E}}_a\Big ( \sum _{j=0}^\infty {\mathrm e}^{-\lambda _n(a) (t-S_j)} \chi (t-S_j) \Big ) \! , \quad \text {where} \quad S_j=\sum _{i=1}^j D_i, \quad {\mathbb {P}}_a(D_i\in \cdot ) = \nu _a(\cdot ). \end{aligned}$$

From (6.5), we obtain similarly

6.8 $$\begin{aligned} {\bar{M}}_{t,u}^{\chi ,\eta }(a,b) ={\mathbb {E}}_{ab}\Big ( \sum _{j=0}^\infty {\mathrm e}^{-S_j[\lambda _n(a)+\lambda _n(b)-\lambda _n(ab)]} {\bar{m}}_{t-S_j}^\chi (a) {\bar{m}}_{u-S_j}^\eta (b) \Big ) \! , \end{aligned}$$

where $S_j=\sum _{i=1}^j D_i$ now has distribution ${\mathbb {P}}_{ab}(D_i\in \cdot ) = \nu _{ab}(\cdot )$.

Note that for a random variable $D$ with law $\nu _a$, for every $h\ge 0$ measurable,

6.9 $$\begin{aligned} {\mathbb {E}}_a(h(D))=\int h(y) a {\mathrm e}^{-\lambda _n(a) y} \, d\mu _n(y). \end{aligned}$$

Moreover, let $\nu ^*_a$ denote the size-biasing of $\nu _a$, i.e.,

6.10 $$\begin{aligned} d\nu ^*_a(y)=\frac{y \, d\nu _a(y)}{\int y \, d\nu _a(y)} \quad \text {so that}\quad {\mathbb {E}}_a(h(D^*))=\frac{{\mathbb {E}}_a(Dh(D))}{{\mathbb {E}}_a(D)} \end{aligned}$$

for $h\ge 0$ measurable. Here and in all of the following we assume that $D$ and $D^*$ have laws $\nu _a$ and $\nu ^*_a$ respectively under ${\mathbb {E}}_a$. Let *U* be uniform on [0, 1], and let $(D_i)_{i \ge 1}$ be independent with law $\nu _a$, and independent of *U* and $D^*$. Besides the random walk $(S_j)_j$ from (6.7)–(6.8), it is useful to study the random walk $(S_j^*)_j$ with

6.11 $$\begin{aligned} S_0^*=UD^* \quad \text {and} \quad S_j^*=S_0^*+\sum _{i=1}^j D_i \quad \text {for all }j\ge 1. \end{aligned}$$

### Random Walk Convergence

In this section, we investigate asymptotics of the random walks in (6.7)–(6.8) and (6.11). Recall that the branching process $\mathsf{BP}^{\scriptscriptstyle (1)}$ is derived from the intensity measure $\mu _n$, where for $h :\left[ 0,\infty \right) \rightarrow \left[ 0,\infty \right)$ measurable,

6.12 $$\begin{aligned} \int h(y) d\mu _n(y) = \int _0^\infty h(f_n(x)) dx. \end{aligned}$$

In particular, all the quantities $z,m,M,\nu _a,D_i,{\mathbb {P}}_a$ and ${\mathbb {E}}_a$ as well as the random walks $(S_j)_j$ and $(S_j^*)_j$ depend implicitly on *n*. We will give sufficient conditions for the sequence of walks $(S_j)_j$, $(S^*_j)_j$ to have scaling limits; in all cases the scaling limit is the Gamma process. This is the content of Theorem 6.3 below.

As a motivation, we first look at the key example $Y_e^{\scriptscriptstyle (K_n)}\overset{d}{=}E^{s_n}$:

#### Example 6.1

Let $$Y_e^{\scriptscriptstyle (K_n)}\overset{d}{=}E^{s_n}$$. Then

6.13 $$\begin{aligned} \begin{aligned}&F_{\scriptscriptstyle Y}(x)=1-{\mathrm e}^{-x^{1/s_n}}\quad \text {and} \quad F_{\scriptscriptstyle Y}^{-1}(x)=(-\log (1-x))^{s_n},\\&f_n(x)= (x/n)^{s_n}=f_n(1) x^{s_n} \quad \text {and} \quad f_n^{-1}(x)=n x^{1/s_n}. \end{aligned} \end{aligned}$$

One easily checks that for all $a>0, \beta >0$,

6.14 $$\begin{aligned} \begin{aligned}&f_n(1) \lambda _n(a^{1/s_n})=a \Gamma (1+1/s_n)^{s_n}, \quad s_n \lambda _n(a^{1/s_n}) {\mathbb {E}}_{a^{1/s_n}}(D)=1,\\&s_n \lambda _n(a^{1/s_n})^2 {\mathbb {E}}_{a^{1/s_n}}(D^2)=1+1/s_n, \quad a \left( {\hat{\mu }}_n \bigl ( \lambda _n(a^{1/s_n})\beta \bigr ) \right) ^{s_n} = 1/\beta . \end{aligned} \end{aligned}$$

Notice that $\Gamma (1+1/s_n)^{s_n} \rightarrow {\mathrm e}^{-\gamma }$ for $n \rightarrow \infty$.

Theorem 6.3 will show that in general the same identities hold asymptotically under our conditions on $f_n$. In fact, we will prove Theorem 6.3 under weaker assumptions on $f_n$:

#### Condition 6.2

There exist $$\varepsilon _0>0$$ and a sequence $$(\delta _n)_{n \in {\mathbb {N}}} \in \left( 0,1\right] ^{{\mathbb {N}}}$$ such that $$s_n f_n(1-\delta _n)/f_n(1)=o(1),$$$$f_n(x^{1/s_n})\ge f_n(1)x^{\varepsilon _0}$$ for $$x\ge 1$$ and $$f_n(x^{1/s_n})\le f_n(1)x^{\varepsilon _0}$$ for $$(1-\delta _n)^{s_n}\le x\le 1$$.

Conditions 2.2 and 2.6 together imply Condition 6.2: we may set $\delta _n=\delta _0$, with $\varepsilon _0$ chosen as for Conditions 2.2 and 2.6, and replacing $(x,x')$ in Lemma 4.1 by $(1,x^{1/s_n})$ or $(x^{1/s_n},1)$ verifies the inequalities in Condition 6.2.

#### Theorem 6.3

(Random walk convergence) Suppose that Conditions 2.1 and 6.2 hold. Then, for any $a\in (0,\infty )$,

- the parameter $\lambda _n(a)$ exists for *n* sufficiently large, and, for all $\beta >0,$
  6.15 $$\begin{aligned}&\lim _{n\rightarrow \infty } f_n(1) \lambda _n(a^{1/s_n}) = a {\mathrm e}^{-\gamma } , \end{aligned}$$
  6.16 $$\begin{aligned}&\lim _{n\rightarrow \infty } s_n\lambda _n(a^{1/s_n}){\mathbb {E}}_{a^{1/s_n}}(D) = 1, \end{aligned}$$
  6.17 $$\begin{aligned}&\lim _{n\rightarrow \infty } s_n\lambda _n(a^{1/s_n})^2{\mathbb {E}}_{a^{1/s_n}}(D^2) = 1, \end{aligned}$$
  6.18 $$\begin{aligned}&\lim _{n\rightarrow \infty } a \left( {\hat{\mu }}_n \bigl ( \lambda _n(a^{1/s_n})\beta \bigr ) \right) ^{s_n} = 1/\beta , \end{aligned}$$
  where $\gamma$ is Euler’s constant;
- under ${\mathbb {E}}_{a^{1/s_n}},$ the process $(\lambda _n(a^{1/s_n}) S_{\lfloor s_n t\rfloor })_{t\ge 0}$ converges in distribution (in the Skorohod topology on compact subsets) to a Gamma process $(\Gamma _t)_{t\ge 0},$ i.e., the Lévy process such that $\Gamma _t$ has the Gamma(*t*, 1) distribution;
- under ${\mathbb {E}}_{a^{1/s_n}},$ the variable $\lambda _n(a^{1/s_n}) D^*$ converges in distribution to an exponential random variable *E* with mean 1,  and the process $(\lambda _n(a^{1/s_n}) S^*_{\lfloor s_n t\rfloor })_{t\ge 0}$ converges in distribution to the sum $(U E+ \Gamma _t)_{t\ge 0}$ where *U* is Uniform on [0, 1] and $U,E,(\Gamma _t)_{t\ge 0}$ are independent.

Moreover, given a compact subset $A\subset (0,\infty ),$ all of the convergences occur uniformly for $a\in A$ and, for (6.18), for $\beta \in A$.

Theorem 6.3 will be proved in Sect. 6.3. We stress that the proof or Theorem 6.3 uses only Conditions 2.1 and 6.2 and the relevant definitions but no other results stated so far.

### Convergence of Random Walks: Proof of Theorem 6.3

For notational convenience, we make the abbreviations

6.19 $$\begin{aligned} {\tilde{x}}=x^{1/s_n}, \quad {\tilde{a}}=a^{1/s_n}, \quad {\tilde{b}}=b^{1/s_n},\text { etc.,} \end{aligned}$$

which we will use extensively in this section and in Sects. 7 and 9.

#### Proof of Theorem 6.3

We begin by proving

6.20 $$\begin{aligned} {\hat{\mu }}_n\left( \frac{a{\mathrm e}^{-\gamma }}{f_n(1)}\right) = 1 - \frac{\log a}{s_n} + o(1/s_n). \end{aligned}$$

Recalling (6.12), we have

6.21 $$\begin{aligned} {\hat{\mu }}_n(\lambda )=\int _0^\infty {\mathrm e}^{-\lambda f_n({\tilde{x}})}d{\tilde{x}}. \end{aligned}$$

Write $${\tilde{f}}_n({\tilde{x}})=f_n({\tilde{x}}){\mathbb {1}}_{\left\{ {\tilde{x}}\ge 1-\delta _n\right\} }$$. Then

6.22 $$\begin{aligned} {\hat{\mu }}_n(\lambda )=\int _0^{\infty } {\mathrm e}^{-\lambda {\tilde{f}}_n({\tilde{x}})} \, d{\tilde{x}} - \int _0^{1-\delta _n} \big (1-{\mathrm e}^{-\lambda f_n({\tilde{x}})}\big ) \, d{\tilde{x}}, \end{aligned}$$

and take $\lambda =a{\mathrm e}^{-\gamma }/f_n(1)$ to estimate $\int _0^{1-\delta _n} (1-{\mathrm e}^{-a{\mathrm e}^{-\gamma }f_n({\tilde{x}})/f_n(1)})\,d{\tilde{x}} = O(f_n(1-\delta _n)/f_n(1))=o(1/s_n)$ by Condition 6.2. Hence, for the purposes of proving (6.20), it is no loss of generality to assume that $f_n(x^{1/s_n})\le f_n(1)x^{\varepsilon _0}$ for all $x\le 1$.

Inspired by (2.5), where $f_n({\tilde{x}})=f_n(1){\tilde{x}}^{s_n}$, we compute

6.23 $$\begin{aligned} \int _0^\infty {\mathrm e}^{-\lambda f_n(1){\tilde{x}}^{s_n}} d{\tilde{x}} = \int _0^\infty \frac{1}{s_n} x^{1/s_n - 1} {\mathrm e}^{-\lambda f_n(1) x} dx = \left( \frac{\Gamma (1+1/s_n)^{s_n}}{\lambda f_n(1)} \right) ^{1/s_n}. \end{aligned}$$

In particular, setting $\lambda =a\Gamma (1+1/s_n)^{s_n}/f_n(1)$ gives $\int _0^\infty \exp \left( -a\Gamma (1+1/s_n)^{s_n}{\tilde{x}}^{s_n} \right) d{\tilde{x}}=a^{-1/s_n}$, which is $1-(\log a)/s_n+o(1/s_n)$. Subtracting this from (6.21), we can therefore prove (6.20) if we show that

6.24 $$\begin{aligned} s_n\int _0^\infty \left( {\mathrm e}^{-a {\mathrm e}^{-\gamma } f_n({\tilde{x}})/f_n(1)} - {\mathrm e}^{-a \Gamma (1+1/s_n)^{s_n} {\tilde{x}}^{s_n}} \right) d{\tilde{x}} \rightarrow 0, \end{aligned}$$

or equivalently, by the substitution ${\tilde{x}}=x^{1/s_n}$, if we show that

6.25 $$\begin{aligned} \int _0^\infty x^{1/s_n - 1} \left( {\mathrm e}^{-a {\mathrm e}^{-\gamma } f_n(x^{1/s_n})/f_n(1)} - {\mathrm e}^{-a \Gamma (1+1/s_n)^{s_n} x} \right) dx \rightarrow 0. \end{aligned}$$

Note that $\Gamma (1+1/s)^s\rightarrow {\mathrm e}^{-\gamma }$ as $s\rightarrow \infty$. Together with Condition 2.1, this implies that the integrand in (6.25) converges pointwise to 0. For $x\le 1$, $f_n(x^{1/s_n}) \le f_n(1)x^{\varepsilon _0}$ means that the integrand is bounded by $O(x^{\varepsilon _0-1}+1)$. For $x\ge 1$, Condition 6.2 implies that the integrand is bounded by ${\mathrm e}^{-\delta x^{\varepsilon _0}}$ for some $\delta >0$. Dominated convergence therefore completes the proof of (6.20). It is easy to see that the proof of (6.25), and hence (6.20), holds uniformly in $a\in A$, where $A \subseteq (0,\infty )$ is a fixed but arbitrary compact set.

To conclude (6.15) from (6.20), we use the monotonicity of $\mu _n$ and $\lambda _n$: given $\delta >0$, we have ${\hat{\mu }}_n\bigl (a{\mathrm e}^{-\gamma }/f_n(1)\bigr )^{s_n}\le a^{-1}+\delta$ for all sufficiently large *n*, uniformly in $a\in A$. Replacing *a* by $a'=(1/a - \delta )^{-1}$ shows that $\lambda _n({\tilde{a}})\le a' {\mathrm e}^{-\gamma }/f_n(1)$ for all *n* large enough, uniformly in *a*. A lower bound holds similarly; take $\delta \rightarrow 0$ to conclude (6.15).

The proof of (6.16) is similar to that of (6.15). Using (6.9), we compute

6.26 $$\begin{aligned} s_n \lambda _n({\tilde{a}}){\mathbb {E}}_{{\tilde{a}}}(D)&= s_n \lambda _n({\tilde{a}})\int y{\tilde{a}} {\mathrm e}^{-\lambda _n({\tilde{a}}) y} d\mu _n(y) \nonumber \\&= \lambda _n({\tilde{a}})f_n(1){\tilde{a}} \int _0^\infty x^{1/s_n - 1} \frac{f_n({\tilde{x}})}{f_n(1)} {\mathrm e}^{-\lambda _n({\tilde{a}}) f_n({\tilde{x}})} dx. \end{aligned}$$

By (2.6) and (6.15), the integrand in (6.26) converges pointwise to ${\mathrm e}^{-a{\mathrm e}^{-\gamma }x}$, and satisfies a similar dominated convergence estimates as (6.25) by Condition 6.2. Hence (6.26) converges to $a{\mathrm e}^{-\gamma }\int _0^\infty {\mathrm e}^{-a {\mathrm e}^{-\gamma } x} dx = 1$ as claimed. The proof of (6.17) is similar.

To prove (6.18), let $b_n$ be defined by $\lambda _n({\tilde{b}}_n)=\beta \lambda _n({\tilde{a}})$. By (6.15) and monotonicity, it follows that $b_n\rightarrow \beta a$ [for if $\limsup _{n \rightarrow \infty } b_n\ge (1+\varepsilon )\beta a$ then $\limsup _{n \rightarrow \infty } f_n(1)\lambda _n({\tilde{b}}_n)\ge (1+\varepsilon )\beta a{\mathrm e}^{-\gamma }=(1+\varepsilon )\lim _{n \rightarrow \infty } f_n(1)\beta \lambda _n({\tilde{a}})$, a contradiction, and similarly if $\liminf _{n \rightarrow \infty } b_n\le (1-\varepsilon )\beta a$]. But ${\hat{\mu }}_n(\lambda _n({\tilde{b}}_n))^{s_n}=b_n^{-1}$, giving the result.

Since $\lambda _n({\tilde{a}}) S_{\lfloor s_n t\rfloor }$ is non-decreasing and right-continuous, and has i.i.d. increments if $s_n t$ is restricted to integer values, it suffices to show that its limiting distribution is $\Gamma (t,1)$ for a fixed *t*, where $\Gamma (t,1)$ denotes a standard Gamma variable with parameter *t*. For this, we note that its Laplace transform is

6.27 $$\begin{aligned} {\mathbb {E}}_{{\tilde{a}}}\left( {\mathrm e}^{-\tau \lambda _n({\tilde{a}})S_{\lfloor s_n t\rfloor }} \right) = \bigl ({\tilde{a}}{\hat{\mu }}_n\bigl ( \lambda _n({\tilde{a}})(1+\tau )\bigr )\bigr )^{\lfloor s_n t\rfloor } . \end{aligned}$$

Since $s_n\rightarrow \infty$, (6.18) yields that the right-hand side tends to $(1+\tau )^{-t}$. This is the Laplace transform of a $\Gamma (t,1)$ variable, and thus completes the proof of (b).

For the proof of part (c) define $b_n$ by $\lambda _n({\tilde{b}}_n)=(1+\tau )\lambda _n({\tilde{a}})$ for a given $\tau \ge 0$. Then (6.10) and (6.9) yield

6.28 $$\begin{aligned} {\mathbb {E}}_{{\tilde{a}}}({\mathrm e}^{-\tau \lambda _n({\tilde{a}}) D^*}) = \frac{{\mathbb {E}}_{{\tilde{a}}}(D{\mathrm e}^{-\tau \lambda _n({\tilde{a}})D})}{{\mathbb {E}}_{{\tilde{a}}}(D)} = \frac{{\tilde{a}} \int y {\mathrm e}^{-\lambda _n({\tilde{a}})y(1+\tau )}d\mu _n(y)}{{\mathbb {E}}_{{\tilde{a}}}(D)} = \frac{{\tilde{a}}{\mathbb {E}}_{{\tilde{b}}_n}(D)}{{\tilde{b}}_n {\mathbb {E}}_{{\tilde{a}}}(D)} . \end{aligned}$$

By the same argument as in the proof of (6.18), $b_n \rightarrow (1+\tau )a$. Combining with (6.16), we conclude that ${\mathbb {E}}_{{\tilde{a}}}({\mathrm e}^{-\tau \lambda _n({\tilde{a}}) D^*})\rightarrow (1+\tau )^{-1}$ and $\lambda _n({\tilde{a}})D^*$ converges to an exponential variable with mean 1. So the rest of part (c) follows from part (b).

The remaining uniformity claims follow from the uniformity in (6.15). The uniformity statements in parts (b) and (c) follow from the observation that the Radon–Nikodym derivatives $d{\mathbb {P}}_{{\tilde{a}}}\left( (\lambda _n({\tilde{a}})S_{\lfloor s_n t\rfloor })_{0\le t\le K}\in \cdot \right) /d{\mathbb {P}}_{{\tilde{a}}'}\left( (\lambda _n({\tilde{a}}')S_{\lfloor s_n t\rfloor })_{0\le t\le K}\in \cdot \right)$ (and similarly for $D^*$ and $S^*$) are tight for $K<\infty$, uniformly over $a,a'\in A$. $\square$

#### Lemma 6.4

For all $$a \in (0,\infty ),$$$$\lambda _n'(a)= 1/(a{\mathbb {E}}_a(D))$$.

#### Proof

Denote ${\hat{\mu }}_n'(\lambda )=\frac{d}{d\lambda } {\hat{\mu }}_n(\lambda )$ and ${\hat{\mu }}_n''(\lambda )=\frac{d^2}{d\lambda ^2} {\hat{\mu }}_n(\lambda )$. Using the definitions of ${\hat{\mu }}_n$ and $\nu _a$, an elementary computation shows that $-a{\hat{\mu }}'_n(\lambda _n(a))={\mathbb {E}}_a(D)$. Moreover, by (3.16), $a{\hat{\mu }}_n(\lambda _n(a))=1$ and the claim follows.

#### Proof of Lemma 3.12

By (6.15), $\lambda _n f_n(1) \rightarrow {\mathrm e}^{-\gamma }$ and Lemma 6.4 gives

6.29 $$\begin{aligned} \phi _n=\frac{\lambda _n'(1)}{\lambda _n(1)}= \frac{1}{\lambda _n(1) {\mathbb {E}}_1(D)}. \end{aligned}$$

Now (6.16) implies that $\phi _n/s_n \rightarrow 1$, as required. $\square$

#### Corollary 6.5

Uniformly for *a* in a compact subset of $(0,\infty ),$

6.30 $$\begin{aligned} \lambda _n(a^{1/s_n}) = \lambda _n(1)\left( 1 +\frac{\phi _n}{s_n} \left( a-1\right) + o\big ((a-1)^2\big ) \right) . \end{aligned}$$

#### Proof

By the same arguments as in the proof of Lemma 6.4, the function $F(a)=\lambda _n(a^{1/s_n})$ satisfies $-{\tilde{a}}{\hat{\mu }}'_n(F(a))={\mathbb {E}}_{{\tilde{a}}}(D)$ and ${\tilde{a}}{\hat{\mu }}''_n(F(a))={\mathbb {E}}_{{\tilde{a}}}(D^2)$. By (3.16), $a{\hat{\mu }}_n(F(a))^{s_n}=1$ and we deduce that $s_n {\mathbb {E}}_{{\tilde{a}}}(D) F'(a)=1/a$ and

6.31 $$\begin{aligned} s_n {\mathbb {E}}_{{\tilde{a}}}(D) F''(a) = \frac{s_n {\mathbb {E}}_{{\tilde{a}}}(D^2)}{\bigl (a s_n {\mathbb {E}}_{{\tilde{a}}}(D)\bigr )^2} - \frac{1+\frac{1}{s_n}}{a^2}. \end{aligned}$$

The right-hand side of (6.31) converges uniformly to 0 by (6.16)–(6.17). Applying (6.16) again and noting from (6.15) that $\lambda _n({\tilde{a}})/\lambda _n(1)$ is bounded, it follows that $F''(a)/\lambda _n(1)$ converges uniformly to 0. A Taylor expansion of *F* around the point 1 and (6.29) complete the proof. $\square$

The following lemma is a consequence of Theorem 6.3 and will be used in the proof of the second moment statements of Theorem 3.13:

#### Lemma 6.6

Assume the hypotheses of Theorem 6.3, and let $A\subset (0,2)$ be compact. For any measurable, bounded function $h \ge 0,$ set

6.32 $$\begin{aligned} \Xi (h)={\mathbb {E}}_{a^{1/s_n}b^{1/s_n}}\left( \sum _{j=0}^\infty {\mathrm e}^{-\left[ \lambda _n(a^{1/s_n})+\lambda _n(b^{1/s_n})-\lambda _n(a^{1/s_n}b^{1/s_n})\right] S_j} h(S_j) \right) . \end{aligned}$$

There are constants $$K<\infty $$ and $$n_0 \in {\mathbb {N}}$$ independent of *h* such that $$\Xi (h)\le Ks_n \left\| h\right\| _\infty $$ for all $$n\ge n_0$$ and $$a,b\in A.$$ Moreover, for any $$\varepsilon >0$$ there are constants $$K'<\infty ,$$$$n_0' \in {\mathbb {N}}$$ independent of *h* such that for all $$a,b\in A,$$$$n\ge n_0',$$

6.33 $$\begin{aligned} -\varepsilon \left\| h\right\| _\infty +\frac{\inf \left\{ h(y):\lambda _n(1)y\le K'\right\} }{\log (1/a+1/b)} \le \frac{\Xi (h)}{s_n} \le \varepsilon \left\| h\right\| _\infty +\frac{\sup \left\{ h(y):\lambda _n(1)y\le K'\right\} }{\log (1/a+1/b)}. \end{aligned}$$

Note that $\log (1/a+1/b)$ is positive and bounded away from 0 by our assumption on *A*.

#### Proof of Lemma 6.6

We rewrite $\Xi (h)$ in integral form, bound *h* by its maximum, and use (6.9) and the definition of ${\hat{\mu }}_n$, to obtain

6.34 $$\begin{aligned} \Xi (h)&=s_n \int _0^\infty {\mathbb {E}}_{{\tilde{a}}{\tilde{b}}}\left( {\mathrm e}^{-[\lambda _n({\tilde{a}})+\lambda _n({\tilde{b}})-\lambda _n({\tilde{a}}{\tilde{b}})]S_{\lfloor s_nt\rfloor }} h(S_{\lfloor s_n t\rfloor }) \right) dt \end{aligned}$$

6.35 $$\begin{aligned}&\le s_n \left\| h\right\| _\infty \int _0^\infty \left[ {\tilde{a}}{\tilde{b}}{\hat{\mu }}_n \! \left( \lambda _n({\tilde{a}})+\lambda _n({\tilde{b}}) \right) \right] ^{\lfloor s_n t\rfloor } dt . \end{aligned}$$

By (6.15) and (6.18), we deduce $(\lambda _n({\tilde{a}})+\lambda _n({\tilde{b}}))/\lambda _n(1) \rightarrow (a+b)$ and

6.36 $$\begin{aligned} \big [{\tilde{a}}{\tilde{b}}{\hat{\mu }}_n(\lambda _n({\tilde{a}})+\lambda _n({\tilde{b}}))\big ]^{s_n}\rightarrow ab/(a+b). \end{aligned}$$

Since $\log ((a+b)/ab)=\log (1/a+1/b)$ is positive and uniformly bounded away from 0, the integral in (6.35) is uniformly bounded for sufficiently large *n* and we conclude that there is a constant $K<\infty$ with $\Xi (h) \le K s_n \left\| h\right\| _\infty$.

For (6.33), let $\varepsilon >0$ be given. Since $A\subset (0,2)$ is compact, (6.15) implies that there exists $\delta >0$ and $n_0' \in {\mathbb {N}}$ such that $\lambda _n({\tilde{a}})+\lambda _n({\tilde{b}})-\lambda _n({\tilde{a}}{\tilde{b}}) \ge \delta f_n(1)$ for all $n\ge n_0'$ and $a,b\in A$. Using again (6.36), we may take $t_0$ and $n_0'$ sufficiently large that $\int _{t_0}^\infty [{\tilde{a}}{\tilde{b}} {\hat{\mu }}_n(\lambda _n({\tilde{a}})+\lambda _n({\tilde{b}}))]^{\lfloor s_n t\rfloor } dt \le \tfrac{1}{3}\varepsilon$ and $\left| \int _0^{t_0} [{\tilde{a}}{\tilde{b}}{\hat{\mu }}_n(\lambda _n({\tilde{a}})+\lambda _n({\tilde{b}}))]^{\lfloor s_n t\rfloor } dt - 1/\log (1/a+1/b)\right| \le \tfrac{1}{3}\varepsilon$ for all $n\ge n_0'$, $a,b\in A$. Furthermore, Theorem 6.3(b) implies that the family of laws ${\mathbb {P}}_{{\tilde{a}}{\tilde{b}}}(\lambda _n(1)S_{\lfloor s_n t\rfloor }\in \cdot )$, $t\le t_0$, $a,b\in A$, is tight. Hence we may take $K'$ large enough that $t_0{\mathrm e}^{-\delta K'}\le 1$ and ${\mathbb {P}}_{{\tilde{a}}{\tilde{b}}}(\lambda _n(1)S_{\lfloor s_n t\rfloor } > K')\le \tfrac{1}{3}\varepsilon$, uniformly for $t\le t_0$. We conclude from (6.34) that

6.37 $$\begin{aligned}&\int _0^{t_0} \left[ \inf \left\{ h(y):\lambda _n(1)y\le K'\right\} {\mathbb {E}}_{{\tilde{a}}{\tilde{b}}}\left( {\mathrm e}^{-[\lambda _n({\tilde{a}})+\lambda _n({\tilde{b}})-\lambda _n({\tilde{a}}{\tilde{b}})]S_{\lfloor s_n t\rfloor }} \right) - \tfrac{1}{3}\varepsilon {\mathrm e}^{-\delta K'} \left\| h\right\| _\infty \right] dt \nonumber \\&\quad \le \frac{\Xi (h)}{s_n} \le \int _0^{t_0} \left[ \sup \left\{ h(y):\lambda _n(1)y\le K'\right\} {\mathbb {E}}_{{\tilde{a}}{\tilde{b}}}\left( {\mathrm e}^{-[\lambda _n({\tilde{a}})+\lambda _n({\tilde{b}})-\lambda _n({\tilde{a}}{\tilde{b}})]S_{\lfloor s_n t\rfloor }} \right) + \tfrac{1}{3}\varepsilon {\mathrm e}^{-\delta K'} \left\| h\right\| _\infty \right] dt \nonumber \\&\qquad + \int _{t_0}^\infty \left\| h\right\| _\infty {\mathbb {E}}_{{\tilde{a}}{\tilde{b}}}\left( {\mathrm e}^{-(\lambda _n({\tilde{a}})+\lambda _n({\tilde{b}})-\lambda _n({\tilde{a}}{\tilde{b}}))S_{\lfloor s_n t\rfloor }} \right) dt \end{aligned}$$

for $n\ge n_0'$. Using again ${\mathbb {E}}_{{\tilde{a}}{\tilde{b}}}({\mathrm e}^{-S_{\lfloor s_n t\rfloor }(\lambda _n({\tilde{a}})+\lambda _n({\tilde{b}})-\lambda _n({\tilde{a}}{\tilde{b}}))})=[{\tilde{a}}{\tilde{b}}{\hat{\mu }}_n(\lambda _n({\tilde{a}})+\lambda _n({\tilde{b}}))]^{\lfloor s_n t\rfloor }$, we see that the hypotheses on $t_0$ and $n_0'$ imply (6.33). $\square$

### Means of One-Vertex Characteristics: Proof of Theorem 3.13

In this section, we prove Theorem 3.13. Further, we set the stage for the proofs of Theorems 3.15 and 3.16 in Sect. 7.

Recall from (6.7) that

$$\begin{aligned} {\bar{m}}_t^\chi (a) = {\mathbb {E}}_a\left( \sum _{j=0}^\infty {\mathrm e}^{-\lambda _n(a)(t-S_j)}\chi (t-S_j) \right) . \end{aligned}$$

Thus, ${\bar{m}}_t^\chi (a)$ can be understood as the expected integral of ${\mathrm e}^{-\lambda _n(a)t}\chi (t)$ against the counting measure on the random set $\left\{ t-S_j:j\in {\mathbb {N}}_0\right\}$. When $t\rightarrow \infty$, this measure approaches its stationary equivalent, which is the counting measure on the point-stationary set $\left\{ t-S^*_j:j\in {\mathbb {N}}_0\right\}$ (see [34]). Since the expected value of this stationary measure is a multiple of the Lebesgue measure, ${\bar{m}}_t^\chi (a)$ will approach (as $t\rightarrow \infty$) the same multiple of $\int _0^\infty {\mathrm e}^{-\lambda _n(a)t} \chi (t) dt$. In the following we make this more precise. We begin with general estimates that apply to any CTBP with any intensity measure $\mu$, and we will write simply $\lambda (a)$ for the parameter defined by the analogue of (3.16). Similarly, all other notation introduced for $\mathsf{BP}^{\scriptscriptstyle (1)}$ will be used for a general CTBP.

#### Proposition 6.7

Let $(S_j^*)_j$ be the random walk defined in (6.11). Let $\chi$ be a non-negative characteristic. Then, for all $a,t> 0,$

6.38 $$\begin{aligned} {\mathbb {E}}_a({\bar{m}}^\chi _{t-UD^*}(a))={\mathbb {E}}_a\left( \sum _{j=0}^\infty {\mathrm e}^{-\lambda (a)(t-S^*_j)}\chi (t-S^*_j) \right) =\frac{\int _0^t {\mathrm e}^{-\lambda (a)u} \chi (u)\, du}{{\mathbb {E}}_a(D)}. \end{aligned}$$

#### Proof

The first equality is (6.7); the second follows because the set $\left\{ t-S^*_j:j \in {\mathbb {N}}_0\right\}$ is point-stationary in the sense of [34]. Alternatively, the equality may be verified by taking Laplace transforms with respect to *t*. $\square$

In (6.7) and (6.38), we may collapse the tail of the sum into a single value of ${\bar{m}}_u^\chi (a)$. Namely, if *J* is a stopping time for $(S_j)_j$ or $(S^*_j)_j$, respectively, then by the strong Markov property

6.39 $$\begin{aligned} \begin{aligned} {\bar{m}}_t^\chi (a)&={\mathbb {E}}_a\left( {\bar{m}}_{t-S_J}^\chi (a) +\sum _{j=0}^{J-1} {\mathrm e}^{-\lambda (a)(t-S_j)} \chi (t-S_j) \right) , \\ \frac{\int _0^t {\mathrm e}^{-\lambda (a)u} \chi (u)\, du}{{\mathbb {E}}_a(D)}&={\mathbb {E}}_a\left( {\bar{m}}_{t-S^*_J}^\chi (a) +\sum _{j=0}^{J-1} {\mathrm e}^{-\lambda (a)(t-S^*_j)} \chi (t-S^*_j) \right) . \end{aligned} \end{aligned}$$

The following lemmas provide bounds on ${\bar{m}}_t^\chi (a)$ when $m_t^\chi (a)$ is non-decreasing in *t*:

#### Lemma 6.8

Suppose $\chi$ is a non-negative characteristic such that $m_t^\chi (a)$ is non-decreasing in *t* (in particular, this holds if $\chi$ is non-decreasing). Let $(S_j)_j$ be as in (6.7) and $(S^*_j)_j$ as in (6.38), and suppose that $(S_j)_j$ and $(S_j^*)_j$ are independent. Let $\varepsilon >0$ and set $J=\inf \left\{ j:\left| S_j-S^*_j\right| \le \varepsilon \right\}$. Then, for $a,t>0,$

6.40 $$\begin{aligned}&{\mathrm e}^{-2\lambda (a)\varepsilon } \frac{\int _0^{t-\varepsilon } {\mathrm e}^{-\lambda (a)u} \chi (u)\, du}{{\mathbb {E}}_a(D)} -{\mathbb {E}}_a\left( \sum _{j=0}^{J-1} {\mathrm e}^{-\lambda (a)(t-\varepsilon -S^*_j)} \chi (t-\varepsilon -S^*_j) \right) \nonumber \\&\quad \le {\bar{m}}_t^\chi (a) \le {\mathrm e}^{2\lambda (a)\varepsilon } \frac{\int _0^{t+\varepsilon } {\mathrm e}^{-\lambda (a)u} \chi (u)\, du}{{\mathbb {E}}_a(D)} +{\mathbb {E}}_a\left( \sum _{j=0}^{J-1} {\mathrm e}^{-\lambda (a)(t-S_j)} \chi (t-S_j) \right) . \end{aligned}$$

#### Proof

The hypotheses imply $t-\varepsilon -S^*_J \le t-S_J \le t+\varepsilon -S^*_J$ and therefore ${\mathrm e}^{-2\lambda (a)\varepsilon } {\bar{m}}_{t-\varepsilon -S^*_J}^\chi (a)\le {\bar{m}}_{t-S_J}^\chi (a)\le {\mathrm e}^{2\lambda (a)\varepsilon } {\bar{m}}_{t+\varepsilon -S^*_J}^\chi (a)$. Combining with (6.39) gives the result. $\square$

#### Lemma 6.9

Suppose $\chi$ is a non-negative characteristic such that $m_t^\chi (a)$ is non-decreasing in *t*. Then, for all $a,t> 0$ and $K>0,$

6.41 $$\begin{aligned} {\bar{m}}_t^\chi (a) \le \frac{{\mathrm e}^K}{{\mathbb {E}}_a\left( {\mathrm e}^{\lambda (a)S^*_0} {\mathbb {1}}_{\left\{ \lambda (a) S^*_0 \le K\right\} } \right) } \frac{\int _0^\infty {\mathrm e}^{-\lambda (a)u} \chi (u) \, du}{{\mathbb {E}}_a(D)}. \end{aligned}$$

#### Proof

On $\left\{ \lambda (a) S^*_0 \le K\right\}$ we have ${\bar{m}}_{t+K/\lambda (a)-S^*_0}^\chi (a)\ge {\mathrm e}^{-K} {\mathrm e}^{\lambda (a) S^*_0} {\bar{m}}_t^\chi (a)$. Apply (6.38) and replace the limit of integration by $\infty$ to obtain the result. $\square$

#### Lemma 6.10

Let $$\chi $$ be a non-negative, non-decreasing characteristic such that $$\int _0^\infty {\mathrm e}^{-\lambda (a)u} \chi (u) \, du <\infty ,$$ and fix $$a,K>0$$. Then, for all $$t>0,$$$$\sum _{j=0}^\infty {\mathrm e}^{-\lambda (a)(t-S_j)} \chi (t-S_j)$$ is square-integrable under $${\mathbb {E}}_a$$ and, abbreviating $$C_{a,K}={\mathrm e}^K/{\mathbb {E}}_a({\mathrm e}^{\lambda (a)S^*_0}{\mathbb {1}}_{\left\{ \lambda (a) S^*_0\le K\right\} })$$,

6.42 $$\begin{aligned} {\mathbb {E}}_a \left( \left( \sum _{j=0}^\infty {\mathrm e}^{-\lambda (a)(t-S_j)} \chi (t-S_j) \right) ^2 \right) \le C_{a,K} \frac{\int _0^\infty {\mathrm e}^{-2\lambda (a)u} \chi (u)^2 du}{{\mathbb {E}}_a(D)}+2C_{a,K}^2\frac{(\int _0^\infty {\mathrm e}^{-\lambda (a)u}\chi (u) du)^2}{{\mathbb {E}}_a(D)^2}. \end{aligned}$$

The same bound holds with $(S_j)_j$ replaced by $(S^*_j)_j$.

#### Proof

Since $\chi$ is non-decreasing, $\int _0^\infty {\mathrm e}^{-\lambda (a)u} \chi (u) du <\infty$ implies that ${\mathrm e}^{-\lambda (a)u} \chi (u)$ must be bounded. Hence $\int _0^\infty {\mathrm e}^{-2\lambda (a)u} \chi (u)^2 du <\infty$ also. Applying Lemma 6.9 to $\chi$ and $\chi ^2$, we deduce

6.43 $$\begin{aligned}&{\mathbb {E}}_a \left( \left( \sum _{j=0}^\infty {\mathrm e}^{-\lambda (a)(t-S_j)} \chi (t-S_j)\right) ^2 \right) \nonumber \\&\quad = {\mathbb {E}}_a\left( \sum _{j=0}^\infty {\mathrm e}^{-2\lambda (a)(t-S_j)} \chi (t-S_j)^2\right) \nonumber \\&\qquad + 2{\mathbb {E}}_a\left( \sum _{j=0}^\infty {\mathrm e}^{-\lambda (a)(t-S_j)} \chi (t-S_j) \sum _{k=j+1}^\infty {\mathrm e}^{-\lambda (a)(t-S_k)} \chi (t-S_k)\right) \nonumber \\&\quad = {\bar{m}}_t^{\chi ^2}(a) + 2{\mathbb {E}}_a\left( \sum _{j=0}^\infty {\mathrm e}^{-\lambda (a)(t-S_j)} \chi (t-S_j) {\bar{m}}_{t-S_{j+1}}^\chi (a)\right) \nonumber \\&\quad \le C_{a,K} \frac{\int _0^\infty {\mathrm e}^{-\lambda (a)u} \chi (u)^2 du}{{\mathbb {E}}_a(D)}+2C_{a,K}\frac{\int _0^\infty {\mathrm e}^{-\lambda (a)u} \chi (u)du}{{\mathbb {E}}_a(D)}{\mathbb {E}}_a\left( \sum _{j=0}^\infty {\mathrm e}^{-\lambda (a)(t-S_j)}\chi (t-S_j)\right) . \end{aligned}$$

Another application of Lemma 6.9 gives (6.42). Finally replacing $(S_j)_j$ by $(S^*_j)_j$ is equivalent to replacing *t* by $t-UD^*$. Since the upper bound in (6.42) does not depend on *t*, the result follows. $\square$

We now specialise to the offspring distribution $\mu _n$ and apply the convergence results of Theorem 6.3 to prove Theorem 3.13:

#### Proof of Theorem 3.13

By Lemma 6.9, for all $a,t>0$,

6.44 $$\begin{aligned} {\bar{m}}^\chi _t({\tilde{a}})\le s_n \frac{{\mathrm e}^1}{{\mathbb {P}}_{{\tilde{a}}}(\lambda _n({\tilde{a}})S_0^*\le 1)}\frac{\int _0^\infty \lambda _n({\tilde{a}}) {\mathrm e}^{-\lambda _n({\tilde{a}}) u} \left\| \chi \right\| _\infty du}{s_n \lambda _n({\tilde{a}}){\mathbb {E}}_{{\tilde{a}}}(D)}, \end{aligned}$$

and, by Theorem 6.3, ${\mathbb {P}}_{{\tilde{a}}}(\lambda _n({\tilde{a}})S_0^*\le 1) \rightarrow {\mathbb {P}}(UE \le 1)$ and $s_n \lambda _n({\tilde{a}}) {\mathbb {E}}_{{\tilde{a}}}(D) \rightarrow 1$ uniformly in $a \in A$. Hence, the uniform bound for ${\bar{m}}_t^\chi ({\tilde{a}})$ follows. By the same reasoning, Lemma 6.10 yields a constant $C<\infty$ such that

6.45 $$\begin{aligned} {\mathbb {E}}_{{\tilde{a}}}\left( \left( \sum _{j=0}^\infty {\mathrm e}^{-\lambda _n({\tilde{a}})(t-S_j)} \chi (t-S_j)\right) ^2\right) \le C s_n^2 \left\| \chi \right\| _\infty ^2, \end{aligned}$$

an estimate that will be needed shortly.

For (3.20), fix $\varepsilon >0$. Apply Lemma 6.8 with $\varepsilon$ and *a* replaced by ${\tilde{\varepsilon }}=\lambda _n({\tilde{a}})^{-1}\varepsilon$ and ${\tilde{a}}$, with the stopping time $J_n=\inf \left\{ j:\left| S_j - S^*_j\right| \le {\tilde{\varepsilon }}\right\} =\inf \left\{ j:\lambda _n({\tilde{a}})\left| S_j-S^*_j\right| \le \varepsilon \right\}$. By (6.15), we may choose *K* large enough that $\int _{t-{\tilde{\varepsilon }}}^\infty \lambda _n({\tilde{a}}){\mathrm e}^{-\lambda _n({\tilde{a}})z} \chi (z) dz \le \left\| \chi \right\| _\infty \varepsilon$ whenever $\lambda _n(1)t\ge K$. By (6.16), it follows that the first terms in the upper and lower bounds of Lemma 6.8 are $s_n\int _0^\infty \lambda _n({\tilde{a}}){\mathrm e}^{-\lambda _n({\tilde{a}})z} \chi (z)dz + O(\varepsilon )s_n \left\| \chi \right\| _\infty$. Therefore it is enough to show that the error term ${\mathbb {E}}_{{\tilde{a}}}\left( \sum _{j=0}^{J_n-1} {\mathrm e}^{-\lambda _n({\tilde{a}})(t-{\tilde{\varepsilon }}-S^*_j)} \chi (t-{\tilde{\varepsilon }}-S^*_j)\right)$ is also $O(\varepsilon )s_n \left\| \chi \right\| _\infty$ for $\lambda _n(1)t$ sufficiently large, uniformly in $a\in A$ (the same proof will work for the term with $S_j$).

To prove this, observe that the variables $(J_n/s_n, \lambda _n({\tilde{a}}) S^*_{J_n})_{n\in {\mathbb {N}},a\in A}$ are tight. Indeed, the rescaled processes $(\lambda _n({\tilde{a}})S_{\lfloor s_n t\rfloor })_{t\ge 0},( \lambda _n({\tilde{a}})S^*_{\lfloor s_n t\rfloor })_{t\ge 0}$ converge by Theorem 6.3 to independent Gamma processes (with different initial conditions). These limiting processes approach to within $\varepsilon /2$ at some random but finite time, and tightness follows.

Thus we may choose $C'$ large enough that the event ${\mathcal {A}}=\left\{ J_n\le C' s_n\right\} \cup \left\{ \lambda _n({\tilde{a}})S^*_{J_n}\le C'\right\}$ satisfies ${\mathbb {P}}_{{\tilde{a}}}({\mathcal {A}}^c)\le \varepsilon ^2$. Using the Cauchy–Schwarz inequality and (6.45),

6.46 $$\begin{aligned}&{\mathbb {E}}_{{\tilde{a}}}\left( {\mathbb {1}}_{{\mathcal {A}}^c} \sum _{j=0}^{J_n-1} {\mathrm e}^{-\lambda _n({\tilde{a}})(t-{\tilde{\varepsilon }}-S^*_j)} \chi (t-{\tilde{\varepsilon }}-S^*_j) \right) \nonumber \\&\quad \le \left[ {\mathbb {P}}_{{\tilde{a}}}({\mathcal {A}}^c) {\mathbb {E}}_{{\tilde{a}}} \! \left( \left( \sum _{j=0}^\infty {\mathrm e}^{-\lambda _n({\tilde{a}})(t-{\tilde{\varepsilon }}-S^*_j)} \chi (t-{\tilde{\varepsilon }}-S^*_j) \right) ^2 \right) \right] ^{1/2} \le \sqrt{C} \varepsilon s_n \left\| \chi \right\| _\infty , \end{aligned}$$

whereas

6.47 $$\begin{aligned} {\mathbb {E}}_{{\tilde{a}}}\left( {\mathbb {1}}_{{\mathcal {A}}} \sum _{j=0}^{J_n-1} {\mathrm e}^{-\lambda _n({\tilde{a}})(t-{\tilde{\varepsilon }}-S^*_j)} \chi (t-{\tilde{\varepsilon }}-S^*_j) \right)&\le C' s_n \left\| \chi \right\| _\infty {\mathrm e}^{-\lambda _n({\tilde{a}})(t-{\tilde{\varepsilon }})+C'}. \end{aligned}$$

By (6.15), the right-hand side is at most $\varepsilon s_n\left\| \chi \right\| _\infty$, uniformly over $a\in A$, if $\lambda _n(1) t \ge K$ with *K* sufficiently large. This completes the proof of (3.20).

We turn to the estimates for ${\bar{M}}_{t,u}^{\chi ,\eta }({\tilde{a}},{\tilde{b}})$. In view of (6.8), apply Lemma 6.6 to $h(y)={\bar{m}}_{t-y}^\chi ({\tilde{a}}){\bar{m}}_{u-y}^\eta ({\tilde{b}})$. By the first part of the current proof, $\left\| h\right\| _{\infty }=O(s_n^2)\left\| \chi \right\| _\infty \left\| \eta \right\| _\infty$ and for any $\varepsilon >0$ we can make the infimum and supremum in (6.33) differ from $s_n^2 \textstyle {\int _0^\infty } {\mathrm e}^{-z}\chi \bigl ( z/\lambda _n({\tilde{a}}) \bigr ) dz \cdot \textstyle {\int _0^\infty } {\mathrm e}^{-w}\eta \bigl ( w/\lambda _n({\tilde{b}}) \bigr ) dw$ by at most $\varepsilon s_n^2$, by taking $\lambda _n(1)[t\wedge u]$ large enough. $\square$

## Continuous-Time Branching Processes: Two-Vertex Characteristics

In view of Theorems 3.11 and 3.27, we wish to consider generation-weighted two-vertex characteristics of the form

7.1 $$\begin{aligned} z_{\vec {t}}^\chi (\vec {a}) = \sum _{v_1\in \mathsf{BP}_{t_1}^{(1)}} \sum _{v_2\in \mathsf{BP}_{t_2}^{(2)}} a_1^{|v_1|} a_2^{|v_2|} \chi (t_1-T_{v_1},t_2-T_{v_2}) \end{aligned}$$

for $\chi (\vec {t})=\chi _n(\vec {t})=\mu _n(\left| t_1-t_2\right| ,t_1+t_2)$ defined in (3.24). As discussed in Sect. 3.3.2, we split $\chi _n$ into $\chi _n^{\scriptscriptstyle (K)}$ and $\chi _n-\chi _n^{\scriptscriptstyle (K)}$ for some $K\in (0,\infty )$.

Regarding $t_1$ as fixed,

7.2 $$\begin{aligned} s_n \chi _n^{\scriptscriptstyle (K)}(t_1,t_2)= s_n \mu _n^{\scriptscriptstyle (K)}(t_1-t_2,t_1+t_2) - {\mathbb {1}}_{\left\{ t_2\ge t_1\right\} } s_n \mu _n^{\scriptscriptstyle (K)}(t_1-t_2,t_2-t_1) \end{aligned}$$

expresses the one-vertex characteristic $s_n\chi _n^{\scriptscriptstyle (K)}(t_1,\cdot )$ as the difference of two uniformly bounded, non-negative, non-decreasing functions.

We extend our abbreviations from (6.19) to vectors and write

7.3 $$\begin{aligned} {\tilde{\vec {a}}}=\vec {a}^{1/s_n} =({\tilde{a}}_1,{\tilde{a}}_2) =(a_1^{1/s_n}, a_2^{1/s_n}) \; \text {etc.} \end{aligned}$$

### Truncated Two-Vertex Characteristic: Proof of Theorem 3.15

In this section, we prove Theorem 3.15. For any two-vertex characteristic $\chi$, note that $z_{\vec {t}}^\chi (\vec {a})$ can be written in terms of two one-vertex characteristics as follows:

7.4 $$\begin{aligned} z_{\vec {t}}^\chi (\vec {a})=z_{t_1}^{\rho ', \mathsf{BP}^{(1)}}(a_1), \quad \text {where}\quad \rho '(t'_1)=\sum _{v_2\in \mathsf{BP}_{t_2}^{(2)}} a_2^{|v_2|} \chi (t'_1,t_2-T_{v_2})=z_{t_2}^{\chi (t'_1,\cdot ), \mathsf{BP}^{(2)}}(a_2). \end{aligned}$$

Similarly, we may evaluate the two-vertex mean ${\bar{m}}_{\vec {t}}^{\chi _n^{\scriptscriptstyle (K)}}(\vec {a})$ via two one-vertex means:

7.5 $$\begin{aligned} {\bar{m}}_{\vec {t}}^{\chi _n^{\scriptscriptstyle (K)}}({\tilde{\vec {a}}}) = {\bar{m}}_{t_1}^{\rho _{t_2,{\tilde{a}}_2}}({\tilde{a}}_1), \quad \text {where}\quad \rho _{t_2,{\tilde{a}}_2}(t'_1) = {\bar{m}}_{t_2}^{\chi _n^{\scriptscriptstyle (K)}(t'_1,\cdot )}({\tilde{a}}_2). \end{aligned}$$

For this reason we first give estimates for the one-vertex characteristic $\chi _n^{\scriptscriptstyle (K)}(t_1,\cdot )$ uniformly in $t_1$. In their statements, we rely on the function $\zeta$ given in (3.30) and on the following definition:

7.6 $$\begin{aligned} I(z)=\int _0^\infty \left( {\mathrm e}^{- \left| y-z\right| } - {\mathrm e}^{-(y+z)} \right) \frac{dy}{y}. \end{aligned}$$

#### Proposition 7.1

Assume the hypotheses of Theorem 6.3. For every $$\varepsilon >0$$ and for every compact subset $$A\subset (0,2),$$ there is a constant $$K_0<\infty $$ such that for every $$K\ge K_0$$ there exist constants $$K'<\infty $$ and $$n_0 \in {\mathbb {N}}$$ such that for $$n \ge n_0,$$$$a_1,a_2,b_1,b_2\in A$$ and $$t'_1\ge 0,$$

7.7 $$\begin{aligned} \begin{aligned} \left| {\bar{m}}_{t_2}^{\chi _n^{\scriptscriptstyle (K)}(t'_1,\cdot )}({\tilde{a}}_2)-I\bigl ( \lambda _n({\tilde{a}}_2)t'_1 \bigr )\right|&\le \varepsilon , \\ \left| s_n^{-3}{\bar{M}}_{t_1,u_1}^{\rho _{t_2,{\tilde{a}}_2},\rho _{u_2,{\tilde{b}}_2}}({\tilde{a}}_1,{\tilde{b}}_1) - \frac{\zeta (a_2/a_1)\zeta (b_2/b_1)}{\log (1/a_1+1/b_1)}\right|&\le \varepsilon , \end{aligned} \quad \text {if} \; \lambda _n(1)[t_1\wedge t_2 \wedge u_1\wedge u_2] \ge K'. \end{aligned}$$

Moreover, for every $K<\infty ,$ there are constants $K''<\infty$ and $n_0' \in {\mathbb {N}}$ such that

7.8 $$\begin{aligned} {\bar{m}}_{t_2}^{\chi _n^{\scriptscriptstyle (K)}(t_1,\cdot )}({\tilde{a}}_2)\le K'', \quad {\bar{M}}_{t_1,u_1}^{\rho _{t_2,{\tilde{a}}_2},\rho _{u_2,{\tilde{b}}_2}}({\tilde{a}}_1,{\tilde{b}}_1) \le K'' s_n^3 \end{aligned}$$

for all $n\ge n_0'$, $t_1,t_2,u_1,u_2\ge 0$ and $a_1,a_2,b_1,b_2\in A$.

Note that ${\bar{m}}_{t_2}^{\chi _n^{\scriptscriptstyle (K)}(t_1,\cdot )}({\tilde{a}}_2)$ is asymptotically constant, instead of growing like $s_n$ as in Theorem 3.13. This reflects the fact that $\chi _n^{\scriptscriptstyle (K)}$ itself is typically of scale $1/s_n$.

#### Proof of Proposition 7.1

The integrand in (7.6) can be bounded by $2{\mathrm e}^{-y+1}$ if $z\le 1$ and by ${\mathbb {1}}_{\left\{ y\le 1\right\} }2{\mathrm e}^{-z+1}+{\mathbb {1}}_{\left\{ y>1\right\} }{\mathrm e}^{-\left| y-z\right| }/y$ if $z\ge 1$. It follows that we may choose $K_0<\infty$ sufficiently large that $\left| \int _{a{\mathrm e}^{-K-\gamma }}^{a{\mathrm e}^{K-\gamma }}({\mathrm e}^{- \left| y-z\right| } - {\mathrm e}^{-(y+z)}) dy/y - I(z)\right| <\tfrac{1}{3}\varepsilon$, for all $z\ge 0$, $a\in A$, $K \ge K_0$. Here, $\gamma$ again denotes Euler’s constant.

Applying Theorem 3.13 to each of the uniformly bounded, non-negative, non-decreasing functions in (7.2), we conclude that for every $K<\infty$, ${\bar{m}}_{t_2}^{\chi _n^{\scriptscriptstyle (K)}(t'_1,\cdot )}({\tilde{a}}_2)$ is uniformly bounded and is within $\tfrac{1}{3}\varepsilon$ of $\int _0^\infty s_n\lambda _n({\tilde{a}}_2){\mathrm e}^{-\lambda _n({\tilde{a}}_2)t}\chi _n^{\scriptscriptstyle (K)}(t'_1,t) \, dt$ if $\lambda _n(1)t_2$ is sufficiently large, uniformly over $a_2\in A$. Use Fubini’s Theorem and (6.12), write $z=\lambda _n({\tilde{a}}_2)t'_1$ and substitute ${\tilde{x}}=x^{1/s_n}$ to compute

7.9 $$\begin{aligned}&\int _0^\infty s_n\lambda _n({\tilde{a}}_2){\mathrm e}^{-\lambda _n({\tilde{a}}_2)t}\chi _n^{\scriptscriptstyle (K)}(t'_1,t) \, dt \nonumber \\&\quad =s_n \int d\mu _n^{\scriptscriptstyle (K)}(y) \int _0^\infty {\mathbb {1}}_{\left\{ \left| y-t'_1\right| \le t\le y+t'_1\right\} } \lambda _n({\tilde{a}}_2){\mathrm e}^{-\lambda _n({\tilde{a}}_2)t} \, dt \nonumber \\&\quad = s_n \int _{1-K/s_n}^{1+K/s_n} \left( {\mathrm e}^{-\lambda _n({\tilde{a}}_2)\left| f_n({\tilde{x}})-t_1'\right| }-{\mathrm e}^{-\lambda _n({\tilde{a}}_2)(f_n({\tilde{x}})+t_1')} \right) d{\tilde{x}} \nonumber \\&\quad = \int _{(1-K/s_n)^{s_n}}^{(1+K/s_n)^{s_n}} \left( {\mathrm e}^{-\vert \lambda _n({\tilde{a}}_2)f_n({\tilde{x}})-z\vert }-{\mathrm e}^{-(\lambda _n({\tilde{a}}_2)f_n({\tilde{x}})+z)} \right) x^{1/s_n-1} dx. \end{aligned}$$

Monotonicity, Condition 2.1 and (6.15) imply that $\lambda _n({\tilde{a}}_2)f_n({\tilde{x}})\rightarrow a_2 {\mathrm e}^{-\gamma }x$ as $n\rightarrow \infty$, uniformly over $x\in [(1-K/s_n)^{s_n},(1+K/s_n)^{s_n}]$ and $a_2\in A$. Hence the integral in (7.9) is within $\tfrac{1}{3}\varepsilon$ of $\int _{{\mathrm e}^{-K}}^{{\mathrm e}^K} ({\mathrm e}^{-\vert a_2{\mathrm e}^{-\gamma }x-z\vert }-{\mathrm e}^{-(a_2{\mathrm e}^{-\gamma }x+z)})dx/x$ for *n* sufficiently large, uniformly in $z\ge 0$ and $a_2\in A$. Substituting $y=a_2{\mathrm e}^{-\gamma }x$ and using $K\ge K_0$, we obtain the desired statements for ${\bar{m}}_{t_2}^{\chi _n^{\scriptscriptstyle (K)}(t'_1,\cdot )}({\tilde{a}}_2)$.

For ${\bar{M}}_{t_1,u_1}^{\rho _{t_2,{\tilde{a}}_2},\rho _{u_2,{\tilde{b}}_2}}({\tilde{a}}_1,{\tilde{b}}_1)$, the statements for ${\bar{m}}_{t_2}^{\chi _n^{\scriptscriptstyle (K)}(t'_1,\cdot )}({\tilde{a}}_2)$ can be interpreted to say that the characteristics $\rho _{t_2,{\tilde{a}}_2}(\cdot ),\rho _{u_2,{\tilde{b}}_2}(\cdot )$ are uniformly bounded and lie within $\tfrac{1}{2}\varepsilon$ of the characteristics $I\!\left( \lambda _n({\tilde{a}}_2) \, \cdot \right)$, $I\!\bigl ( \lambda _n({\tilde{b}}_2) \, \cdot \bigr )$ if $\lambda _n(1)t_2,\lambda _n(1)u_2$ are sufficiently large. It is readily verified that *I*(*z*) can be written as the difference of two bounded, non-negative, non-decreasing functions. We may therefore apply Theorem 3.13 to these characteristics. A calculation shows that

7.10 $$\begin{aligned} \int _0^\infty {\mathrm e}^{-z}I(rz)dz=\zeta (r), \quad r=\lambda _n({\tilde{a}}_2)/\lambda _n({\tilde{a}}_1), \end{aligned}$$

where $\zeta$ is defined in (3.30). By (6.15), we have $r\rightarrow a_2/a_1$ uniformly over $a_1,a_2 \in A$; since $\zeta$ is continuous, this completes the proof. $\square$

#### Proof of Theorem 3.15

Interchanging the roles of $t_1$ and $t_2$ in (7.2) and using Proposition 7.1, we can write $\rho _{t_2, {\tilde{a}}_2}$ in (7.5) as the difference of two bounded, non-negative, non-decreasing functions and Theorem 3.13 yields that $\frac{1}{s_n}{\bar{m}}_{\vec {t}}^{\chi _n^{\scriptscriptstyle (K)}}({\tilde{\vec {a}}})$ is bounded. To show (3.28), Proposition 7.1 allows us to replace $\rho _{t_2, {\tilde{a}}_2}$ in (7.5) by $I(\lambda _n({\tilde{a}}_2\cdot ))$, making an error of at most $\varepsilon s_n$. Since *I* can be written as the difference of two bounded, non-negative, non-decreasing functions, Theorem 3.13, (7.10) and the fact that $\zeta (r)\rightarrow \zeta (a_2/a_1)$ uniformly, yield the claim.

For ${\bar{M}}_{\vec {t},\vec {u}}^{\chi _n^{\scriptscriptstyle (K)},\chi _n^{\scriptscriptstyle (K)}}({\tilde{\vec {a}}},{\tilde{\vec {b}}})$, use (7.4) to obtain [similarly to (6.8)]

7.11 $$\begin{aligned} {\bar{M}}_{\vec {t},\vec {u}}^{\chi ,\eta }(\vec {a},\vec {b})&= {\mathbb {E}}_{a_1 b_1, a_2 b_2} \left( \sum _{j_1=0}^\infty \sum _{j_2=0}^\infty {\mathrm e}^{-[\lambda _n(a_1)+\lambda _n(b_1)-\lambda _n(a_1 b_1)] S_{j_1}^{\scriptscriptstyle (1)}} {\mathrm e}^{-[\lambda _n(a_2)+\lambda _n(b_2)-\lambda _n(a_2 b_2)] S_{j_2}^{\scriptscriptstyle (2)}} \right. \nonumber \\&\qquad \qquad \qquad \qquad \left. \cdot \, {\bar{m}}_{t_1-S_{j_1}^{\scriptscriptstyle (1)} \! , \, t_2-S_{j_2}^{\scriptscriptstyle (2)}}^\chi (\vec {a}) {\bar{m}}_{u_1-S_{j_1}^{\scriptscriptstyle (1)} \! , \, u_2-S_{j_2}^{\scriptscriptstyle (2)}}^\eta (\vec {b}) \!\right) \! , \end{aligned}$$

where now $(S_{j}^{\scriptscriptstyle (1)})_j$ and $(S_{j}^{\scriptscriptstyle (2)})_j$ are independent random walks and $(S_j^{\scriptscriptstyle (i)})_j$ has step distribution $\nu _{a_i b_i}$, $i=1,2$. Applying Lemma 6.6 twice and using the results from the first part of the proof, we obtain the desired conclusions. $\square$

### The Effect of Truncation: Proof of Theorem 3.16

In this section, we control the effect of truncation and prove Theorem 3.16, by showing that the remainder $\chi _n-\chi _n^{\scriptscriptstyle (K)}$ has a negligible first moment.

We will write $\chi _n=\int d\mu _n(y) \Psi _y$, where

7.12 $$\begin{aligned} \Psi _y(t_1,t_2)={\mathbb {1}}_{\left\{ \left| t_1-t_2\right| \le y, t_1+t_2\ge y\right\} }. \end{aligned}$$

The same is true for $\chi _n^{\scriptscriptstyle (K)}$ and $\mu _n^{\scriptscriptstyle (K)}$, so that, by (6.12) and the substitution ${\tilde{x}}=x^{1/s_n}$,

7.13 $$\begin{aligned} s_n^{-1}{\bar{m}}^{\chi _n-\chi _n^{(K)}}_{\vec {t}}(\vec {1})&= \int _0^{1-\delta _0} s_n^{-1} {\bar{m}}^{\Psi _{f_n({\tilde{x}})}}_{\vec {t}}(\vec {1}) d{\tilde{x}}\nonumber \\&\quad + \left( \int _{(1-\delta _0)^{s_n}}^{(1-K/s_n)^{s_n}} +\int _{(1+K/s_n)^{s_n}}^\infty \right) s_n^{-2}{\bar{m}}^{\Psi _{f_n({\tilde{x}})}}_{\vec {t}}(\vec {1}) x^{1/s_n - 1} dx \nonumber \\&= I_0+I_1+I_2 . \end{aligned}$$

We must therefore show that $I_0,I_1,I_2$ are uniformly bounded and can be made small by making *K* and $\lambda _n(1)[t_1\wedge t_2]$ large. To this end, we will bound the two-vertex mean ${\bar{m}}^{\Psi _y}_{\vec {t}}(\vec {1})$ in terms of one-vertex means. Abbreviate

7.14 $$\begin{aligned} \eta ^{\scriptscriptstyle (q)}(t)={\mathbb {1}}_{\left\{ 0\le t\le q\right\} }. \end{aligned}$$

#### Lemma 7.2

For any $$y\in (0,\infty ),$$

7.15 $$\begin{aligned}&{\bar{m}}^{\Psi _y}_{\vec {t}}(\vec {1}) \le \frac{1}{y} \bigg [ \int _0^\infty {\mathrm e}^{-2\lambda _n(1)r}{\bar{m}}_{t_1-r}^{\eta ^{\scriptscriptstyle (2y)}}(1){\bar{m}}_{t_2-r}^{\eta ^{\scriptscriptstyle (2y)}}(1) dr \nonumber \\&\quad + \int _0^y {\mathrm e}^{-\lambda _n(1)(y-r)} \left( {\bar{m}}_{t_1-y+r}^{\eta ^{\scriptscriptstyle (2r)}}(1){\bar{m}}_{t_2}^{\eta ^{\scriptscriptstyle (r)}}(1) + {\bar{m}}_{t_2-y+r}^{\eta ^{\scriptscriptstyle (2r)}}(1){\bar{m}}_{t_1}^{\eta ^{\scriptscriptstyle (r)}}(1) \right) \! dr \bigg ]. \end{aligned}$$

#### Proof

Note that

7.16 $$\begin{aligned} \Psi _y\le \frac{1}{y}\Big [\int _0^\infty {\mathbb {1}}_{[r,r+2y]^2}dr+\int _0^y({\mathbb {1}}_{[y-r,y+r]\times [0,r]}+{\mathbb {1}}_{[0,r]\times [y-r,y+r]}) dr\Big ] \end{aligned}$$

since, for any $\vec {t}$ for which $\Psi _y(\vec {t})>0$, the measure of the sets of parameter values *r* for which $\vec {t}$ belongs to the relevant rectangles is at least *y* in total. Then the identities ${\bar{m}}^{{\mathbb {1}}_{[a,b]\times [c,d]}}_{t_1,t_2}(\vec {a})={\bar{m}}_{t_1}^{{\mathbb {1}}_{[a,b]}}(a_1){\bar{m}}_{t_2}^{{\mathbb {1}}_{[c,d]}}(a_2)$ and

7.17 $$\begin{aligned} {\bar{m}}^{{\mathbb {1}}_{[c,d]}}_t(a) = {\mathrm e}^{-\lambda _n(a)c} {\bar{m}}_{t-c}^{\eta ^{\scriptscriptstyle (d-c)}}(a) \end{aligned}$$

complete the proof. $\square$

Using Lemma 7.2, it will suffice to bound the one-vertex means ${\bar{m}}^{\eta ^{\scriptscriptstyle (q)}}_t(1)$. We will use different bounds depending on the relative sizes of *q*, *t* and $f_n(1)$, as in the following lemma:

#### Lemma 7.3

There is a constant $C<\infty$ such that, for *n* sufficiently large,

7.18a $$\begin{aligned}&{\bar{m}}_t^{\eta ^{\scriptscriptstyle (q)}}(1) \le Cs_n \quad \text {for all }t,q\ge 0, \end{aligned}$$

7.18b $$\begin{aligned}&{\bar{m}}_t^{\eta ^{\scriptscriptstyle (q)}}(1) \le Cs_n\frac{q+s_n f_n(1-\delta _0)}{f_n(1)} \quad \text {if }t\ge f_n(1-1/s_n),\quad q\le \tfrac{1}{2}t, \end{aligned}$$

7.18c $$\begin{aligned}&{\bar{m}}_t^{\eta ^{\scriptscriptstyle (q)}}(1) \le C\frac{q+s_n f_n(1-\delta _0)}{s_n t(1-f_n^{-1}(t))^2} \quad \text {if } t<f_n(1),\quad q\le \tfrac{1}{2}t, \end{aligned}$$

7.18d $$\begin{aligned}&{\bar{m}}_t^{\eta ^{\scriptscriptstyle (2q)}}(1) \le \frac{2}{1-f_n^{-1}(q)}, \quad \text {if }q<f_n(1),\quad t \ge 0. \end{aligned}$$

#### Proof

Theorem 3.13 and $\left\| \eta ^{\scriptscriptstyle (q)}\right\| _\infty =1$ imply (7.18a). For (7.18d), use the representation (6.7) and note that, starting from the first index *J* for which $\eta ^{\scriptscriptstyle (2q)}(t-S_J)\ne 0$ (if one exists), the total number of indices *j* for which $\eta ^{\scriptscriptstyle (2q)}(t-S_j)\ne 0$ is stochastically bounded by the waiting time (starting from *J*) until the second step where $Y_j>q$. Then ${\mathbb {P}}_1(D_j\le q)\le f_n^{-1}(q)$ proves (7.18d).

For (7.18b)–(7.18c), we employ a size-biasing argument on the jump sizes $D_i$. For $i\le j$, write $S'_{j,i}=\sum _{1\le k\le j, k\ne i} D_k$. We can therefore rewrite (6.7) (noting that the term $j=0$ vanishes) as

7.19 $$\begin{aligned} {\bar{m}}_t^{\eta ^{\scriptscriptstyle (q)}}(1) = \sum _{j=1}^\infty \sum _{i=1}^j {\mathbb {E}}_1\left( {\mathrm e}^{-\lambda _n(1)(t-S'_{j,i})} {\mathbb {E}}_1\left( \left. {\mathrm e}^{\lambda _n(1)D_i} \frac{D_i}{S'_{j,i}+D_i} {\mathbb {1}}_{\left\{ t-q-S'_{j,i}\le D_i \le t-S'_{j,i}\right\} }\,\right| S'_{j,i}\right) \right) . \end{aligned}$$

We split according to whether $D_i>f_n(1-\delta _0)$ or $D_i\le f_n(1-\delta _0)$. For any measurable function $h\ge 0$, (6.9) and Lemma 4.2 imply

7.20 $$\begin{aligned} {\mathbb {E}}_1\left( {\mathrm e}^{\lambda _n(1)D_i} D_i h(D_i){\mathbb {1}}_{\left\{ D_i> f_n(1-\delta _0)\right\} } \right) \le \frac{1}{\varepsilon _0s_n} \int h(y) f_n^{-1}(y) dy. \end{aligned}$$

On the other hand, ${\mathbb {E}}_1\left( {\mathrm e}^{\lambda _n(1)D_i}D_i h(D_i){\mathbb {1}}_{\left\{ D_i\le f_n(1-\delta _0)\right\} } \right) \le \max \left\{ yh(y):y\le f_n(1-\delta _0)\right\}$ by (6.9). Consequently, writing $x^+:=x\vee 0$,

7.21 $$\begin{aligned} {\bar{m}}_t^{\eta ^{\scriptscriptstyle (q)}}(1)&\le \sum _{j=1}^\infty \sum _{i=1}^j {\mathbb {E}}_1\left( {\mathrm e}^{-\lambda _n(1)(t-S'_{j,i})} {\mathbb {1}}_{\left\{ t-S'_{j,i}\ge 0\right\} } \left[ \frac{1}{\varepsilon _0 s_n} \int _{(t-q-S'_{j,i})^+}^{t-S'_{j,i}} \frac{f_n^{-1}(y)}{S'_{j,i}+y}dy + \frac{f_n(1-\delta _0)}{t-q} \right] \right) \nonumber \\&\le \sum _{j=1}^\infty \sum _{i=1}^j {\mathbb {E}}_1\left( {\mathrm e}^{-\lambda _n(1)(t-S'_{j,i})} {\mathbb {1}}_{\left\{ t-S'_{j,i}\ge 0\right\} } \left[ \frac{f_n^{-1}(t-S'_{j,i})}{\varepsilon _0s_n} \log \left( \frac{t}{t-q} \right) + \frac{f_n(1-\delta _0)}{t-q} \right] \right) \nonumber \\&\le \frac{2}{t}\sum _{j=1}^\infty \sum _{i=1}^j {\mathbb {E}}_1\left( {\mathrm e}^{-\lambda _n(1)(t-S'_{j,i})} {\mathbb {1}}_{\left\{ t-S'_{j,i}\ge 0\right\} } \left[ \frac{qf_n^{-1}(t-S'_{j,i})}{\varepsilon _0s_n} + f_n(1-\delta _0) \right] \right) \end{aligned}$$

since $-\log (1-x) \le 2x$ for $x \in [0,1/2]$ and $q\le \tfrac{1}{2}t$. To obtain (7.18c), we note that ${\mathbb {P}}_1(S'_{j,i}\le t)\le f_n^{-1}(t)^{j-1}$, as in the proof of (7.18d), and therefore

7.22 $$\begin{aligned} {\bar{m}}_t^{\eta ^{\scriptscriptstyle (q)}}(1) \le C'\frac{qf_n^{-1}(t)+s_nf_n(1-\delta _0)}{s_n t} \sum _{j=1}^\infty jf_n^{-1}(t)^{j-1}, \end{aligned}$$

and $f_n^{-1}(t) < f_n^{-1}(f_n(1))=1$ completes the proof of (7.18c).

Finally, to prove (7.18b) we now reverse the argument that led to (7.21) by reintroducing a term $D_i$. By (6.12),

7.23 $$\begin{aligned} \int y_i{\mathbb {1}}_{\left\{ y_i\le f_n(1)\right\} } d\mu _n(y_i)=\int _0^1 f_n({\tilde{x}})d{\tilde{x}}\ge \int _{1-1/s_n}^1 f_n(1-1/s_n) d{\tilde{x}}=\frac{f_n(1-1/s_n)}{s_n}, \end{aligned}$$

so that ${\mathbb {E}}_1({\mathrm e}^{\lambda _n(1)D_i}D_i {\mathbb {1}}_{\left\{ D_i\le f_n(1)\right\} } /f_n(1-1/s_n)) \ge 1/s_n$. Abbreviate $\rho (u)={\mathbb {1}}_{\left\{ u\ge 0\right\} }[q f_n^{-1}(u)/\varepsilon _0+s_n f_n(1-\delta _0)]$. Note that $\rho$ is increasing and that on $\left\{ D_i\le f_n(1)\right\}$ we have $t-S'_{j,i}\le t-S_j+f_n(1)$. Continuing from (7.21), we estimate

7.24 $$\begin{aligned} {\bar{m}}_t^{\eta ^{\scriptscriptstyle (q)}}(1)&\le \frac{2}{t}\sum _{j=1}^\infty \sum _{i=1}^j {\mathbb {E}}_1\left( {\mathrm e}^{-\lambda _n(1)(t-S'_{j,i})} \rho (t-S'_{j,i}) {\mathbb {E}}_1\left( {\mathrm e}^{\lambda _n(1)D_i} \frac{D_i}{f_n(1-1/s_n)} {\mathbb {1}}_{\left\{ D_i\le f_n(1)\right\} } \right) \right) \nonumber \\&\le \frac{2}{t}\sum _{j=1}^\infty \sum _{i=1}^j {\mathbb {E}}_1\left( {\mathrm e}^{-\lambda _n(1)(t-S_j)} \rho (t+f_n(1)-S_j) \frac{D_i}{f_n(1-1/s_n)} \right) \nonumber \\&= \frac{2{\mathrm e}^{\lambda _n(1)f_n(1)}}{f_n(1-1/s_n)} \sum _{j=0}^\infty {\mathbb {E}}_1\left( {\mathrm e}^{-\lambda _n(1)(t+f_n(1)-S_j)} \rho (t+f_n(1)-S_j)\frac{S_j}{t} \right) \nonumber \\&\le \frac{2 {\mathrm e}^{\lambda _n(1)f_n(1)}}{f_n(1-1/s_n)} \frac{t+f_n(1)}{t} {\bar{m}}_{t+f_n(1)}^\rho (1) , \end{aligned}$$

where in the last inequality we have used that $\rho (t+f_n(1)-S_j)=0$ if $S_j > t+f_n(1)$. As in the proof of Theorem 3.13, we use Lemma 6.9, Theorem 6.3 and the definition of $\rho$ to obtain

7.25 $$\begin{aligned} {\bar{m}}_{t+f_n(1)}^\rho (1)&\le O(1) \int _0^{\infty } s_n \lambda _n(1) {\mathrm e}^{-\lambda _n(1)u} \rho (u) \, du \nonumber \\&= O(s_n) \Big [ \frac{q}{\varepsilon _0} \int _0^{\infty } \lambda _n(1){\mathrm e}^{-\lambda _n(1) u} f_n^{-1}(u) \, du +s_n f_n(1-\delta _0)\Big ]. \end{aligned}$$

By Condition 6.2, we have $f_n^{-1}(u)\le (u/f_n(1))^{1/\varepsilon _0s_n}$ for $u\ge f_n(1)$. Changing variables and using that $\varepsilon _0s_n\ge 1$ for large *n*, we obtain

7.26 $$\begin{aligned}&\int _0^{\infty } \lambda _n(1){\mathrm e}^{-\lambda _n(1) u} f_n^{-1}(u) \, du \le 1+ \int _0^{\infty } \lambda _n(1)f_n(1) {\mathrm e}^{-\lambda _n(1) f_n(1) u} u \,\nonumber \\&du= 1+\frac{1}{\lambda _n(1) f_n(1)}=O(1) \end{aligned}$$

according to (6.15). Hence ${\bar{m}}_{t+f_n(1)}^\rho (1)=O(s_n)(q+s_n f_n(1-\delta _0))$. The other factors in (7.24) are $O(1/f_n(1))$ because of (6.15), Condition 2.1, and the assumption $t\ge f_n(1-1/s_n)$. This completes the proof of (7.18b). $\square$

To make use of the bounds (7.18c)–(7.18d), we note the following consequence of Condition 2.2:

#### Lemma 7.4

Suppose without loss of generality that the constant $\delta _0$ from Condition 2.2 satisfies $\delta _0<1$. Then there exists $C<\infty$ such that, uniformly over $f_n(1-\delta _0)\le u\le f_n(1),$

7.27 $$\begin{aligned} f_n^{-1}(u)\le 1-\frac{1}{Cs_n}\log (f_n(1)/u). \end{aligned}$$

#### Proof

It suffices to show that $x\le 1-(Cs_n)^{-1} \log (f_n(1)/f_n(x))$ for $1-\delta _0\le x\le 1$, i.e., that $\log (f_n(1)/f_n(x))\le Cs_n(1-x)$. But Condition 2.2 implies that $\log (f_n(1)/f_n(x))\le \varepsilon _0^{-1}s_n \log (1/x)$, as in the proof of Lemma 4.1, so a Taylor expansion gives the result.

#### Proof of Theorem 3.16

We will show that each of the terms $I_0,I_1,I_2$ in (7.13) is uniformly bounded, and furthermore can be made arbitrarily small by taking *K* large enough (for $I_1$ and $I_2$) and $\lambda _n(1)[t_1\wedge t_2]$ large enough (for $I_0$). We begin with the term $I_2$ [i.e., $x\ge (1+K/s_n)^{s_n}$]. Lemma 7.2, (7.18a) and (6.15) give

7.28 $$\begin{aligned}&{\bar{m}}^{\Psi _{f_n({\tilde{x}})}}_{\vec {t}}(\vec {1}) \le \frac{O(s_n^2)}{f_n({\tilde{x}})} \left[ \int _0^\infty {\mathrm e}^{-2\lambda _n(1)r} dr +2\int _0^{f_n({\tilde{x}})} {\mathrm e}^{-\lambda _n(1)(f_n({\tilde{x}})-r)}dr \right] =\frac{O(s_n^2)f_n(1)}{f_n({\tilde{x}})}. \end{aligned}$$

By Lemma 4.1, $f_n({\tilde{x}})\ge f_n(1)x^{\varepsilon _0}$ for $x\ge 1$, so that $I_2\le O(1)\int _{(1+K/s_n)^{s_n}}^{\infty } x^{1/s_n-\varepsilon _0-1}\, dx$. Since $\int _1^\infty x^{-\varepsilon _0-1}\,dx<\infty$, it follows that $I_2$ is uniformly bounded and can be made arbitrarily small by taking *K*, and hence $(1+K/s_n)^{s_n}$, large enough, uniformly over $t_1,t_2$.

For $I_1$, we again start by estimating ${\bar{m}}^{\Psi _{y}}_{\vec {t}}(\vec {1})$ where $y=f_n({\tilde{x}})$ with ${\tilde{x}} \in [1-\delta _0,1-K/s_n]$. Suppose for definiteness, and without loss of generality by symmetry, that $t_1\le t_2$. Split the first integral from Lemma 7.2 into the intervals $[0,t_1-f_n(1-1/s_n)]$, $[t_1-f_n(1-1/s_n),t_1-4y]$ and $[t_1-4y,t_1]$ (noting that the integrand vanishes for $r>t_1$) and denote the summand by $\theta _n^{\scriptscriptstyle 11}(y)$, $\theta _n^{\scriptscriptstyle 12}(y)$ and $\theta _n^{\scriptscriptstyle 13}(y)$. The second summand in Lemma 7.2 is called $\theta _n^{\scriptscriptstyle 14}(y)$. The corresponding parts of $I_1$ are denoted by $I_{11}, \ldots , I_{14}$.

We first estimate $\theta _n^{\scriptscriptstyle 13}(y)$ and $\theta _n^{\scriptscriptstyle 14}(y)$. Since $f_n(1-\delta _0)\le y \le f_n(1-K/s_n) < f_n(1)$, (7.18d) and Lemma 7.4 give

7.29 $$\begin{aligned} \theta _n^{\scriptscriptstyle 13}(y)+\theta _n^{\scriptscriptstyle 14}(y)&\le \frac{1}{y} \Big [\int _{t_1-4y}^{t_1} \Big (\frac{2}{1-f_n^{-1}(y)}\Big )^2 \, dr + \int _0^y 2 \frac{2}{1-f_n^{-1}(r)} \frac{2}{1-f_n^{-1}(r/2)}\, dr \Big ]\nonumber \\&\le \frac{O(1)}{(1-f_n^{-1}(y))^2} \le \frac{O(s_n^2)}{\big (\log (f_n(1)/y)\big )^2}. \end{aligned}$$

According to Lemma 4.1, $y=f_n({\tilde{x}})\le f_n(1)x^{\varepsilon _0}$ for all $1-\delta _0\le {\tilde{x}} \le 1$. Substitute $x={\mathrm e}^{-u}$ to obtain

7.30 $$\begin{aligned} I_{13}+I_{14}&\le O(1) \int _{(1-\delta _0)^{s_n}}^{(1-K/s_n)^{s_n}} \frac{1}{(\log (1/x^{\varepsilon _0}))^2} x^{1/s_n-1} \, dx\le O(1) \int _{K}^{\infty } \frac{1}{u^2} \, du. \end{aligned}$$

Hence $I_{13}+I_{14}$ is uniformly bounded and can be made arbitrarily small by taking *K* large.

For $\theta _n^{\scriptscriptstyle 11}(y)$, $r \in [0,t_1-f_n(1-1/s_n)]$ implies $t_2-r \ge t_1-r \ge f_n(1-1/s_n)$ and since $2y \le 2f_n(1-K/s_n) \le \frac{1}{2} f_n(1-1/s_n)$ for large *n* by Condition 2.1, we can apply first (7.18b) and then (6.15) to obtain

7.31 $$\begin{aligned} \theta _n^{\scriptscriptstyle 11}(y)\le & {} \frac{1}{y} \int _0^{t_1-f_n(1-1/s_n)} {\mathrm e}^{-2\lambda _n(1) r} O(s_n^2)\Big ( \frac{2y +s_nf_n(1-\delta _0)}{f_n(1)}\Big )^2 dr \,\nonumber \\= & {} O(s_n^2) \frac{(y +s_nf_n(1-\delta _0))^2}{yf_n(1)}. \end{aligned}$$

Using that $(a+b)^2 \le 2(a^2 + b^2)$ for all $a,b \in {\mathbb {R}}$ and that $f_n({\tilde{x}})\le f_n(1)x^{\varepsilon _0}$ and, for $x\in (1-\delta _0,1-K/s_n)$, $f_n({\tilde{x}})\ge f_n(1-\delta _0)$, we obtain

7.32 $$\begin{aligned} I_{11}&\le \int _{(1-\delta _0)^{s_n}}^{(1-K/s_n)^{s_n}} O(1) \Big (\frac{f_n({\tilde{x}})}{f_n(1)} + \frac{s_n^2 f_n(1-\delta _0)^2}{f_n({\tilde{x}})f_n(1)} \Big ) x^{1/s_n-1} \, dx \nonumber \\&\le O(1) \bigg [\int _{(1-\delta _0)^{s_n}}^{(1-K/s_n)^{s_n}} x^{\varepsilon _0+1/s_n-1} \, dx + s_n^2 \frac{f_n(1-\delta _0)}{f_n(1)} \int _{(1-\delta _0)^{s_n}}^{(1-K/s_n)^{s_n}} x^{1/s_n-1} \, dx\bigg ]. \end{aligned}$$

The first summand is bounded and can be made arbitrarily small by choosing *K* large. The second summand is arbitrarily small for large *n* uniformly in *K* since $f_n(1-\delta _0)/f_n(1) \le (1-\delta _0)^{\varepsilon _0s_n} = o(s_n^{-3})$ according to Lemma 4.1.

For $\theta _n^{\scriptscriptstyle 12}(y)$ we substitute $u=t_1-r$ to obtain

7.33 $$\begin{aligned} \theta _n^{\scriptscriptstyle 12}(y) = \frac{1}{y} \int _{4y}^{f_n(1-1/s_n)} {\mathrm e}^{-2\lambda _n(1) (t_1-u)} {\bar{m}}_u^{\eta ^{\scriptscriptstyle (2y)}}(1) {\bar{m}}_{t_2-t_1+u}^{\eta ^{\scriptscriptstyle (2y)}}(1) \, du. \end{aligned}$$

We consider the two cases $t_2-t_1 \ge f_n(1)/2$ and $0 \le t_2-t_1 <f_n(1)/2$ separately. First $t_2 -t_1 \ge f_n(1)/2$. Then $t_2-t_1+u \ge f_n(1)/2+4 f_n(1-\delta _0) \ge f_n(1-1/s_n)$ for sufficiently large *n*. Hence (7.18b), (7.18c) and Lemma 7.4 yield

7.34 $$\begin{aligned} \theta _n^{\scriptscriptstyle 12}(y)&\le \frac{1}{y} \int _{4y}^{f_n(1-1/s_n)}O(1) \frac{2y +s_nf_n(1-\delta _0)}{s_n u (1-f_n^{-1}(u))^2} s_n \frac{2y +s_nf_n(1-\delta _0)}{f_n(1)} \, du \nonumber \\&\le O(s_n^2) \frac{(y +s_nf_n(1-\delta _0))^2}{yf_n(1)} \int _{4y}^{f_n(1-1/s_n)}\frac{1}{u \big (\log (f_n(1)/u)\big )^2} \, du \nonumber \\&\le O(s_n^2) \frac{(y +s_nf_n(1-\delta _0))^2}{yf_n(1)} \int _{\log (f_n(1)/f_n(1-1/s_n))}^\infty \frac{1}{\xi ^2} \, d\xi , \end{aligned}$$

where the last integral [in which $\xi =\log (f_n(1)/u)$] is *O*(1) since $\log (f_n(1)/f_n(1-1/s_n))\rightarrow 1$ as $n\rightarrow \infty$. Hence, in the case $t_2-t_1 \ge f_n(1)/2$, we have the same bound for $\theta _n^{\scriptscriptstyle 12}(y)$ as for $\theta _n^{\scriptscriptstyle 11}(y)$.

Now let $t_2-t_1 <f_n(1)/2$, and abbreviate $u'=t_2-t_1+u$. Recalling that $f_n(1-1/s_n)/f_n(1)\rightarrow {\mathrm e}^{-1}$, we have $u' \le f_n(1)/2 +f_n(1-1/s_n) < \tfrac{9}{10}f_n(1)$ for large *n*, uniformly over $u\le f_n(1-1/s_n)$. We first apply (7.18c) to both factors in (7.33) and then use Lemma 7.4 to obtain

7.35 $$\begin{aligned} \theta _n^{\scriptscriptstyle 12}(y)&\le \frac{1}{y} \int _{4y}^{f_n(1-1/s_n)}O(1) \frac{2y +s_nf_n(1-\delta _0)}{s_n u (1-f_n^{-1}(u))^2} \frac{2y +s_nf_n(1-\delta _0)}{s_n u' (1-f_n^{-1}(u'))^2} \, du\nonumber \\&\le O(1) \frac{(y+s_nf_n(1-\delta _0))^2}{ys_n^2} \int _{4y}^{f_n(1-1/s_n)} \frac{s_n^2}{u \big (\log (f_n(1)/u)\big )^2} \frac{s_n^2}{u' \big (\log (f_n(1)/u')\big )^2} \, du. \end{aligned}$$

In (7.35), we have $u'\ge u$, and we are particularly concerned with the case $u'=u$. The function $u'\mapsto (u'/f_n(1)) (\log (f_n(1)/u'))^2$ is not monotone over $u' \in \left[ u,\tfrac{9}{10}f_n(1)\right]$, but it is increasing over $\left( 0,\tfrac{1}{10}f_n(1)\right)$ and bounded from zero and infinity over $\left[ \tfrac{1}{10}f_n(1),\tfrac{9}{10}f_n(1)\right]$. We may therefore find a constant $c>0$ such that $u'(\log (f_n(1)/u'))^2 \ge c u(\log (f_n(1)/u))^2$ whenever $u\le u'\le \tfrac{9}{10}f_n(1)$. The bound (7.35) may therefore be simplified to

7.36 $$\begin{aligned} \theta _n^{\scriptscriptstyle 12}(y) \le O(s_n^2) \frac{(y+s_nf_n(1-\delta _0))^2}{y} \int _{4y}^{f_n(1-1/s_n)} \frac{1}{u^2 \log (f_n(1)/u)^4} \, du, \end{aligned}$$

and an integration by parts shows that

7.37 $$\begin{aligned} \theta _n^{\scriptscriptstyle 12}(y) \le O(s_n^2) \frac{(y+s_nf_n(1-\delta _0))^2}{y^2 (\log (f_n(1)/y))^4}. \end{aligned}$$

Inserting the bound (7.37) into $I_{12}$ and using again $(a+b)^2 \le 2(a^2+b^2)$, we conclude that

7.38 $$\begin{aligned} I_{12}&\le \int _{(1-\delta _0)^{s_n}}^{(1-K/s_n)^{s_n}} O(1) \frac{f_n({\tilde{x}})^2+s_n^2 f_n(1-\delta _0)^2}{f_n({\tilde{x}})^2 (\log (f_n(1)/f_n({\tilde{x}})))^4 } x^{1/s_n-1} \, dx. \end{aligned}$$

Recall from Lemma 4.1 the bounds $f_n({\tilde{x}})\le f_n(1)x^{\varepsilon _0}$ and $f_n(1-\delta _0)\le f_n({\tilde{x}})((1-\delta _0)/{\tilde{x}})^{\varepsilon _0s_n}$. We split the integral for the second summand in (7.38) at $(1-\delta _0/2)^{s_n}$. For $x \ge (1-\delta _0/2)^{s_n}$, $f_n(1-\delta _0)\le f_n({\tilde{x}})(1-\delta ')^{\varepsilon _0s_n}$ for some $\delta ' \in (0,1)$. For $x \le (1-\delta _0/2)^{s_n}$, $\log (f_n(1)/f_n({\tilde{x}})) \ge \varepsilon _0\log (1/x) \ge c s_n$ for some $c>0$. Hence

7.39 $$\begin{aligned} I_{12}&\le O(1) \Big (1+ s_n^2(1-\delta ')^{\varepsilon _0s_n}\Big ) \int _{(1-\delta _0)^{s_n}}^{(1-K/s_n)^{s_n}} \frac{1}{x(\log (1/x))^4}\, dx \nonumber \\&\quad +O(1) \int _{(1-\delta _0)^{s_n}}^{(1-\delta _0/2)^{s_n}} \frac{s_n^2 f_n(1-\delta _0)^2}{f_n({\tilde{x}})^2 s_n^4} x^{1/s_n-1} \, dx \nonumber \\&\le O(1) \int _0^{{\mathrm e}^{-K}} \frac{1}{x(\log (1/x))^4}\, dx + O\left( s_n^{-2}\right) \int _{(1-\delta _0)^{s_n}}^{(1-\delta _0/2)^{s_n}}(1-\delta _0)^{2 \varepsilon _0s_n} x^{-1-2 \varepsilon _0} \, dx. \end{aligned}$$

The first summand in (7.39) is bounded and can be made arbitrarily small by choosing *K* large, while the second summand is arbitrarily small for large *n* uniformly in *K*. We have now handled all four contributions to $I_1$.

Finally, for $I_0$, let $y\le f_n(1-\delta _0)$ and note that $\Psi _y(t'_1,\cdot )={\mathbb {1}}_{[|t'_1-y|,t'_1+y]}\le {\mathbb {1}}_{[(t'_1-y)^+,t'_1+y]}$, where $(t_1'-y)^+:=(t_1'-y) \vee 0$. From (7.17) and (7.18d) it follows that

7.40 $$\begin{aligned} {\bar{m}}^{\Psi _y(t'_1,\cdot )}_{t_2}(1)&\le {\mathbb {1}}_{\left\{ t_2\ge t'_1-f_n(1-\delta _0)\right\} } {\mathrm e}^{-\lambda _n(1) (t_1'-y)^+} {\bar{m}}_{t_2-(t_1'-y)^+}^{\eta ^{\scriptscriptstyle (t_1'+y-(t_1'-y)^{+})}}(1) \nonumber \\&\le {\mathbb {1}}_{\left\{ t_2\ge t'_1-f_n(1-\delta _0)\right\} } \frac{2}{1-f_n^{-1}(2f_n(1-\delta _0))} =O(1) {\mathbb {1}}_{[0,t_2+f_n(1-\delta _0)]}(t_1'), \end{aligned}$$

and therefore ${\bar{m}}^{\Psi _y}_{\vec {t}}(\vec {1})\le O(s_n)$ by (7.4)–(7.5) and (7.18a). We conclude that $I_0$ is uniformly bounded. To show the smallness, we sharpen the bound (7.40) when $t'_1$ is far from $t_2$ by using (7.18b) instead of (7.18d), to obtain for $t_2\ge t'_1+f_n(1-\delta _0)+f_n(1)$,

7.41 $$\begin{aligned} {\bar{m}}^{\Psi _y(t'_1,\cdot )}_{t_2}(1)\le {\bar{m}}_{t_2-(t_1'-y)^+}^{\eta ^{\scriptscriptstyle (t_1'+y-(t_1'-y)^{+})}}(1)\le O(s_n) \frac{2y+s_n f_n(1-\delta _0)}{f_n(1)}= O(s_n^2)\frac{f_n(1-\delta _0)}{f_n(1)}. \end{aligned}$$

Combining (7.40)–(7.41), we have ${\bar{m}}^{\Psi _y(t'_1,\cdot )}_{t_2}(1) \le O(s_n^2)\frac{f_n(1-\delta _0)}{f_n(1)}+O(1){\mathbb {1}}_{\left\{ t_2-2f_n(1)\le t'_1\le t_2+f_n(1)\right\} }$. Applying (7.4)–(7.5), (7.17), (7.18a) and (6.15) we conclude that

7.42 $$\begin{aligned} {\bar{m}}^{\Psi _y}_{\vec {t}}(\vec {1})\le O(s_n^3)\frac{f_n(1-\delta _0)}{f_n(1)} + O(s_n) {\mathrm e}^{-\lambda _n(1)t_2} \end{aligned}$$

and consequently $I_0$ may be made small by taking $t_2$ large. $\square$

## First Points of Cox Processes: Proof of Theorem 3.29

Let ${\mathcal {X}}$ denote a topological space equipped with its Borel $\sigma$-field and let $({\mathcal {P}}_n)_{n\ge 1}$ be a sequence of Cox processes on ${\mathbb {R}}\times {\mathcal {X}}$ with random intensity measures $(Z_n)_{n\ge 1}$. That is, there exist $\sigma$-fields ${\mathscr {F}}_n$ such that $Z_n$ is ${\mathscr {F}}_n$-measurable and, conditionally on ${\mathscr {F}}_n$, ${\mathcal {P}}_n$ is a Poisson point process with (random) intensity $Z_n$. For instance, Theorem 3.27 expresses the first passage distance and hopcount in terms of the first point of a Cox process. In this section, we determine sufficient conditions to identify the limiting distribution of the first points of ${\mathcal {P}}_n$ based on the intensity measure at fixed times *t*.

This section is organised as follows. We start in Sect. 8.1 with preparations of convergence of Cox processes. In Sect. 8.2, we use these results to prove Theorem 3.29.

### Preparations: Weak Convergence of Cox Processes

We will write ${\mathcal {P}}_{n,t}$ for the measure defined by ${\mathcal {P}}_{n,t}(\cdot )={\mathcal {P}}_n(\left( -\infty ,t\right] \times \cdot )$, and given a partition $t_0<\cdots <t_N$ we abbreviate $\Delta {\mathcal {P}}_{n,i}={\mathcal {P}}_{n,t_i}-{\mathcal {P}}_{n,t_{i-1}}$; similarly for $Z_{n,t}, \Delta Z_{n,i}$. Write $\left| \mu \right|$ for the total mass of a measure $\mu$.

Define

8.1 $$\begin{aligned} T_{n,k}=\inf \left\{ t:\left| {\mathcal {P}}_{n,t}\right| \ge k\right\} , \end{aligned}$$

and let $A_{n,k}$ be the event that $T_{n,j}\notin \left\{ \pm \infty \right\}$ and $\left| {\mathcal {P}}_{n,T_{n,j}}\right| =j$, for $j=1,\dotsc ,k$. That is, $A_{n,k}$ is the event that the points of ${{\,\mathrm{supp}\,}}{\mathcal {P}}_n$ with the *k* smallest *t*-values are uniquely defined. On $A_{n,k}$, let $X_{n,k}$ denote the unique point for which ${\mathcal {P}}_n(\left\{ T_{n,k}\right\} \times \left\{ X_{n,k}\right\} )=1$, and otherwise set $X_{n,k}=\dagger$, an isolated cemetery point.

We will impose the following conditions on the intensity measures $(Z_n)_n$, expressed in terms of a probability measure *Q* on ${\mathcal {X}}$ and a family ${\mathcal {H}}$ of measurable functions $h:{\mathcal {X}}\rightarrow {\mathbb {R}}$.

#### Condition 8.1

(Regularity of Cox process intensities)

- For any $t\in {\mathbb {R}}$ and for any $h\in {\mathcal {H}},$
  8.2 $$\begin{aligned} \int _{{\mathcal {X}}} h \, dZ_{n,t} - \left| Z_{n,t}\right| \int _{{\mathcal {X}}} h \, dQ {\mathop {\longrightarrow }\limits ^{\scriptscriptstyle {{\mathbb {P}}}}}0. \end{aligned}$$
- For each $\varepsilon >0,$ there exists ${\underline{t}}\in {\mathbb {R}}$ such that
  8.3 $$\begin{aligned} \liminf _{n\rightarrow \infty }{\mathbb {P}}\left( \left| Z_{n,{\underline{t}}}\right| <\varepsilon \right) \ge 1-\varepsilon . \end{aligned}$$
- For each $\varepsilon >0,$ there exists ${\overline{t}}\in {\mathbb {R}}$ such that
  8.4 $$\begin{aligned} \liminf _{n\rightarrow \infty }{\mathbb {P}}\left( \left| Z_{n,{\overline{t}}}\right| >1/\varepsilon \right) \ge 1-\varepsilon . \end{aligned}$$
- For each $\varepsilon >0$ and each ${\underline{t}}<{\overline{t}},$ there exists a partition $t_0={\underline{t}}<t_1<\cdots <t_N={\overline{t}}$ of $[{\underline{t}},{\overline{t}}]$ such that
  8.5 $$\begin{aligned} \liminf _{n\rightarrow \infty } {\mathbb {P}}\left( \sum _{i=1}^N \left| \Delta Z_{n,i}\right| ^2 \le \varepsilon \right) \ge 1-\varepsilon . \end{aligned}$$

We make the convention that any function *h* on ${\mathcal {X}}$ is extended to ${\mathcal {X}}\cup \left\{ \dagger \right\}$ by $h(\dagger )=0$.

#### Proposition 8.2

Suppose that Condition 8.1 holds for a probability measure *Q* on $${\mathcal {X}}$$ and a family $${\mathcal {H}}$$ of bounded measurable functions $$h:{\mathcal {X}}\rightarrow {\mathbb {R}}$$. Then, for each fixed $$k\in {\mathbb {N}},$$$${\mathbb {P}}(A_{n,k})\rightarrow 1,$$ the collection $$\left\{ (T_{n,j})_{j=1}^k :n\in {\mathbb {N}}\right\} $$ of random vectors is tight, and

8.6 $$\begin{aligned} {\mathbb {E}}\left( \left. \prod _{j=1}^k g_j(T_{n,j}) h_j(X_{n,j})\,\right| {\mathscr {F}}_n\right) - {\mathbb {E}}\left( \left. \prod _{j=1}^k g_j(T_{n,j})\,\right| {\mathscr {F}}_n\right) \prod _{j=1}^k \int _{{\mathcal {X}}} h_j \, dQ {\mathop {\longrightarrow }\limits ^{\scriptscriptstyle {{\mathbb {P}}}}}0 \end{aligned}$$

for all bounded continuous functions $g_1,\dotsc ,g_k:{\mathbb {R}}\rightarrow {\mathbb {R}}$ and all $h_1,\dotsc ,h_k\in {\mathcal {H}}$.

#### Theorem 8.3

Suppose Condition 8.1 holds when either

$${\mathcal {H}}$$
is the family of all bounded continuous functions on ${\mathcal {X}};$
$${\mathcal {X}}={\mathbb {R}}^d$$ and $${\mathcal {H}}$$
is the family of functions $h(\vec {x})={\mathrm e}^{i \vec {\xi }\cdot \vec {x}}$ for $\vec {\xi }\in {\mathbb {R}}^d;$ or
$${\mathcal {X}}=\left[ 0,\infty \right) ^d$$ and $${\mathcal {H}}$$
is the family of functions $h(\vec {x})={\mathrm e}^{-\vec {\xi }\cdot \vec {x}}$ for

Then

- the random sequence $(X_{n,j})_{j=1}^\infty$ converges in distribution [with respect to the product topology on $({\mathcal {X}}\cup \left\{ \dagger \right\} )^{\mathbb {N}}]$ to a random sequence $(X_j)_{j=1}^\infty ,$ where the $X_j$ are independent with law *Q*; 
- the sequence $(X_{n,j})_{j=1}^\infty$ is asymptotically independent of ${\mathscr {F}}_n;$
- the collection $\left\{ (T_{n,j})_{j=1}^k :n\in {\mathbb {N}}\right\}$ of random vectors is tight; and
- if $(T_j,X_j)_{j=1}^\infty$ is any subsequential limit in distribution of $(T_{n,j},X_{n,j})_{j=1}^\infty ,$ then $(T_j)_{j=1}^\infty$ and $(X_j)_{j=1}^\infty$ are independent.

#### Proof of Theorem 8.3 assuming Proposition 8.2

Because of the product topology, it suffices to consider finite sequences $(T_{n,j},X_{n,j})_{j=1}^k$ for a fixed $k\in {\mathbb {N}}$. Applying (8.6) with $g_j(t)=1$ gives the convergence of $(X_{n,j})_{j=1}^k$. The independence of $(T_j)_{j=1}^k$ and $(X_j)_{j=1}^k$ follows from the product form of (8.6), and the asymptotic independence of $X_j$ from ${\mathscr {F}}_n$ follows because of the conditional expectations in (8.6). $\square$

We first prove the following lemma. Given $t_0<\cdots <t_N$, write $B_{n,k}$ for the event that there exist (random) integers $1\le I_1<\cdots <I_k\le N$ with $\left| \Delta {\mathcal {P}}_{n,I_j}\right| =1$ for $j=1,\dotsc ,k$ and $\left| \big .\smash {{\mathcal {P}}_{n,t_{I_k}}}\right| =k$. (That is, $B_{n,k}$ is the event that each of the first *k* points of ${\mathcal {P}}_n$ is the unique point in some interval $\left( t_{i-1},t_i\right]$. In particular $B_{n,k}\subset A_{n,k}$.)

#### Lemma 8.4

Assume Conditions 8.1 (b)–(c). Then, given $\varepsilon >0$ and $k\in {\mathbb {N}},$ there exists $[{\underline{t}},{\overline{t}}]$ and a partition ${\underline{t}}=t_0<\cdots <t_N={\overline{t}}$ of $[{\underline{t}},{\overline{t}}]$ such that $\liminf _{n\rightarrow \infty } {\mathbb {P}}(B_{n,k})\ge 1-\varepsilon$. In particular, ${\mathbb {P}}(A_{n,k})\rightarrow 1$.

#### Proof

Given a partition ${\underline{t}}=t_0<\cdots <t_N={\overline{t}}$, the complement $B_{n,k}^c$ is the event that ${\mathcal {P}}_n$ contains a point in $\left( -\infty ,{\underline{t}}\right]$, fewer than *k* points in $\left( -\infty ,{\overline{t}}\right]$, or more than one point in some interval $\left( t_{i-1},t_i\right]$. By Conditions 8.1 (b)–(c), we may choose ${\underline{t}},{\overline{t}}$ such that the first two events each have probability at most $\varepsilon /3$ for *n* large. Since ${\mathbb {P}}\left( \left. \left| \Delta {\mathcal {P}}_{n,i}\right| \ge 2 \,\,\right| Z_n\right) = 1-{\mathrm e}^{-\left| \Delta Z_{n,i}\right| }(1+\left| \Delta Z_{n,i}\right| ) \le \left| \Delta Z_{n,i}\right| ^2$, Condition 8.1 (c) gives a partition of $[{\underline{t}},{\overline{t}}]$ such that the third event also has probability at most $\varepsilon /3$ for *n* large. $\square$

#### Proof of Proposition 8.2

Fix any $\varepsilon >0$ and bounded, continuous functions $g_1,\ldots ,g_k$. Choose $t_0<\cdots <t_N$ as in Lemma 8.4. By taking a refinement, we may assume that $\left| g_j(t)-g_j(t_i)\right| \le \varepsilon$ for each $t\in \left( t_{i-1},t_i\right]$ and each *i*, *j*. Define $\psi (t)=t_i$ if $t_{i-1}< t \le t_i$ and $\psi (t)=t_N$ otherwise, and set ${\tilde{g}}_j=g_j\circ \psi$. Partitioning according to the integers $I_j$,

8.7 $$\begin{aligned}&{\mathbb {1}}_{B_{n,k}}\prod _{j=1}^k {\tilde{g}}_j(T_{n,j})h_j(X_{n,j})= \sum _{\vec {i}} {\mathbb {1}}_{B_{n,k}}{\mathbb {1}}_{\left\{ \vec {I}=\vec {i}\right\} } \prod _{j=1}^k g_j(t_{i_j}) \int _{{\mathcal {X}}} h_j \, \Delta {\mathcal {P}}_{n,i_j} , \end{aligned}$$

where the sum is over $\vec {i} \in {\mathbb {N}}^k$ with $1\le i_1<\cdots <i_k\le N$, and we write $\vec {I}=(I_1, \ldots , I_k)$. Observe that a Poisson point process ${\mathcal {P}}$ with intensity $\mu$ satisfies ${\mathbb {E}}({\mathbb {1}}_{\left\{ \left| {\mathcal {P}}\right| =1\right\} } \int h \, d{\mathcal {P}})={\mathrm e}^{-\left| \mu \right| }\int h \, d\mu$. Consequently,

8.8 $$\begin{aligned}&{\mathbb {E}}\left( \left. {\mathbb {1}}_{B_{n,k}}\prod _{j=1}^k {\tilde{g}}_j(T_{n,j})h_j(X_{n,j})\,\right| {\mathscr {F}}_n\right) = \sum _{\vec {i}} {\mathrm e}^{-\left| Z_{n,t_{i_k}}\right| } \prod _{j=1}^k g_j(t_{i_j}) \int _{\mathcal {X}}h_j \, \Delta Z_{n,i_j}. \end{aligned}$$

Apply (8.8) twice, with the original $h_j$’s and with the constant functions ${\tilde{h}}_j(x)=\int h_j\, dQ$, to get

8.9 $$\begin{aligned}&{\mathbb {E}}\left( \left. {\mathbb {1}}_{B_{n,k}} \left[ \prod _{j=1}^k {\tilde{g}}_j(T_{n,j}) h_j(X_{n,j}) - \prod _{j=1}^k {\tilde{g}}_j(T_{n,j}) \int _{\mathcal {X}}h_j \, dQ \right] \,\right| {\mathscr {F}}_n\right) \nonumber \\&\quad = \sum _{\vec {i}} {\mathrm e}^{-\left| Z_{n,t_{i_k}}\right| } \left[ \prod _{j=1}^k g_j(t_{i_j}) \int _{\mathcal {X}}h_j \, \Delta Z_{n,i_j} - \prod _{j=1}^k g_j(t_{i_j}) \left| \Delta Z_{n,i_j}\right| \int _{\mathcal {X}}h_j \, dQ \right] . \end{aligned}$$

The right-hand side of (8.9) is bounded (since $g_j$, $h_j$, and $\vert \Delta Z_{n,i_j}\vert {\mathrm e}^{-\vert \Delta Z_{n,i_j}\vert }$ are bounded) and, by Condition 8.1 (a), converges to 0 in probability, and hence also in expectation. By the choice of the partition, $\left| {\tilde{g}}_j(T_{n,j})-g_j(T_{n,j})\right| \le \varepsilon$ on $B_{n,k}$ and $\limsup _{n\rightarrow \infty }{\mathbb {P}}(B_{n,k}^c)\le \varepsilon$. Now let $(F_n)_n$ be a uniformly bounded sequence of ${\mathbb {R}}$-valued random variables such that $F_n$ is ${\mathscr {F}}_n$-measurable. Since all the functions involved are bounded, there exists $C<\infty$ such that

8.10 $$\begin{aligned}&\limsup _{n\rightarrow \infty } \left| {\mathbb {E}}\left( F_n\prod _{j=1}^k g_j(T_{n,j}) h_j(X_{n,j}) \right) - {\mathbb {E}}\left( F_n\prod _{j=1}^k g_j(T_{n,j}) \right) \prod _{j=1}^k \int _{\mathcal {X}}h_j \, dQ\right| \le C\varepsilon , \end{aligned}$$

which completes the proof. $\square$

When ${\mathcal {X}}={\mathbb {R}}^d$, another natural family is ${\mathcal {H}}=\left\{ h(\vec {x})={\mathrm e}^{\vec {\xi } \cdot \vec {x}}:\vec {\xi }\in {\mathbb {R}}^d\right\}$. However, these functions are *not* bounded, so it is necessary to modify the argument of Proposition 8.2 and Theorem 8.3. Recall from (3.54) that we write ${\hat{R}}$ for the moment generating function of a measure *R* on ${\mathbb {R}}^d$.

#### Proposition 8.5

Let $${\mathcal {X}}={\mathbb {R}}^d$$. Suppose Condition 8.1 holds when $${\mathcal {H}}$$ is the family of functions $$h(x)={\mathrm e}^{\vec {\xi } \cdot \vec {x}}$$ for $$\vec {\xi }\in {\mathbb {R}}^d,$$$$\vert \vec {\xi }\vert \le \delta ,$$ where $$\delta >0$$ and $${\hat{Q}}(\vec {\xi })<\infty $$ for all $$\vert \vec {\xi }\vert \le \delta $$. Then the conclusions (a)–(d) of Theorem 8.3 hold.

#### Proof

Fix any $\varepsilon >0$, $k\in {\mathbb {N}}$, $g_1,\ldots ,g_k$ bounded, continuous functions, and choose $t_0<\cdots <t_N$ as in Lemma 8.4. By taking a refinement, we may assume that $t_i-t_{i-1}\le \varepsilon$. Let $C_n$ be the event that ${\hat{Z}}_{n,t_i}(\vec {\xi }_0) \le \left| Z_{n,t_i}\right| {\hat{Q}}(\vec {\xi }_0) + \varepsilon$ for each $i=1,\dotsc ,N$ and for each $\vec {\xi }_0\in \{\delta /\sqrt{d}, -\delta /\sqrt{d}\}^d$. By Condition 8.1 (a), ${\mathbb {P}}(C_n)\rightarrow 1$. Let $X_j$ be independent random variables with law *Q*, and define ${\tilde{X}}_{n,j}=X_{n,j}$ on $B_{n,k}\cap C_n$ and ${\tilde{X}}_{n,j}=X_j$ otherwise. Recall the notations $\psi (t),{\tilde{g}}_j(t)$ from the proof of Proposition 8.2 and set ${\tilde{T}}_{n,j}=\psi (T_{n,j})$. Set $h_j(\vec {x})={\mathrm e}^{\vec {\xi }_j\cdot \vec {x}}$ for $\Vert \vec {\xi }_j\Vert _\infty \le \delta /\sqrt{d}$. By the argument of the previous proof, this time using that the ${\tilde{X}}_{n,j}$ have law *Q* on $(B_{n,k}\cap C_n)^c$, we find

8.11 $$\begin{aligned}&{\mathbb {E}}\left( \left. \prod _{j=1}^k g_j({\tilde{T}}_{n,j})h_j({\tilde{X}}_{n,j}) - \prod _{j=1}^k g_j({\tilde{T}}_{n,j}) {\hat{Q}}(\vec {\xi }_j)\,\right| {\mathscr {F}}_n\right) \nonumber \\&\quad = {\mathbb {1}}_{C_n} \sum _{\vec {i}} {\mathrm e}^{-\left| Z_{n,t_{i_k}}\right| } \left[ \prod _{j=1}^k g_j(t_{i_j}) \widehat{\Delta Z}_{n,i_j}(\vec {\xi }_j) - \prod _{j=1}^k g_j(t_{i_j})\left| \Delta Z_{n,i_j}\right| {\hat{Q}}(\vec {\xi }_j) \right] . \end{aligned}$$

By Condition 8.1 (a), the right-hand side of (8.11) converges to 0 in probability. Moreover, by the bound ${\mathrm e}^{\vec {\xi }_j\cdot \vec {x}}\le \sum _{\vec {\xi }_0\in \{\pm \delta /\sqrt{d}\}^d} {\mathrm e}^{\vec {\xi }_0\cdot \vec {x}}$ and the choice of $C_n$, it is bounded as well. Hence we may repeat the argument from the proof of Theorem 8.3 to find that $({\tilde{T}}_{n,j}, {\tilde{X}}_{n,j})_j$ satisfy the desired conclusions. But by construction, $\liminf _{n\rightarrow \infty } {\mathbb {P}}(X_{n,j} = {\tilde{X}}_{n,j}, |T_{n,j}-{\tilde{T}}_{n,j}|\le \varepsilon ) \ge 1-\varepsilon$. Since $\varepsilon >0$ was arbitrary, it follows that $X_{n,j}$ and $T_{n,j}$ themselves have the same convergence properties. $\square$

### Convergence of First Point of Cox Processes: Proof of Theorem 3.29

In this section, we use the above conditions for convergence of Cox processes to prove Theorem 3.29:

#### Proof of Theorem 3.29

For any $\varepsilon >0$ we may define

8.12 $$\begin{aligned} {\underline{t}}(\varepsilon )&=\max \left\{ t\in {\mathbb {Z}},t<-1/\varepsilon :q(t)<\varepsilon ^2\right\} , \end{aligned}$$

8.13 $$\begin{aligned} {\overline{t}}(\varepsilon )&=\min \left\{ t\in {\mathbb {Z}},t>1/\varepsilon :\liminf _{n\rightarrow \infty } {\mathbb {P}}(\vert Z^*_{n,t}\vert>1/\varepsilon )>1-\varepsilon \right\} , \end{aligned}$$

8.14 $$\begin{aligned} K_1(\varepsilon )&=\min \left\{ k_1\in {\mathbb {N}}:\liminf _{n\rightarrow \infty } {\mathbb {P}}\left( {\mathbb {E}}\left( \left. \big \vert Z^{\prime \scriptscriptstyle (K)}_{n,{\overline{t}}(\varepsilon )}\big \vert \,\right| {\mathscr {F}}'_n\right) \le 2q({\overline{t}}(\varepsilon )) \right) \ge 1-\varepsilon \text { for all } K\ge k_1\right\} \nonumber \\&\quad \vee \min \left\{ k_1\in {\mathbb {N}}:\liminf _{n\rightarrow \infty } {\mathbb {P}}\left( {\mathbb {E}}\left( \left. \big \vert Z^{\prime \prime \scriptscriptstyle (K)}_{n,{\overline{t}}(\varepsilon )}\big \vert \,\right| {\mathscr {F}}'_n\right) \le \varepsilon ^2 \right) \ge 1-\varepsilon \text { for all } K\ge k_1\right\} , \end{aligned}$$

by $\lim _{t\rightarrow -\infty }q(t)=0$, assumption (b), (3.55) and (3.57), respectively. Given any $\varepsilon >0$, let $\varepsilon '\in (0,\varepsilon )$ be arbitrary. By construction, ${\overline{t}}(\varepsilon ')\le {\overline{t}}(\varepsilon )$, so taking $K=K_1(\varepsilon ')$ shows that ${\mathbb {E}}\left( \left. \big \vert Z^*_{n,{\overline{t}}(\varepsilon )}\big \vert \,\right| {\mathscr {F}}'_n\right)$ is uniformly bounded apart from an event of probability at most $2\varepsilon '+o(1)$. Since $\varepsilon '$ was arbitrary, it follows that $\big \vert Z^*_{n,{\overline{t}}(\varepsilon )}\big \vert$ is tight as $n\rightarrow \infty$, so we may define

8.15 $$\begin{aligned} z_0(\varepsilon )=\min \left\{ z\in {\mathbb {N}}:\limsup _{n\rightarrow \infty } {\mathbb {P}}(\vert Z^*_{n,{\overline{t}}(\varepsilon )}\vert \le z)\ge 1-\varepsilon \right\} . \end{aligned}$$

For $\varepsilon >0,t,u\in {\mathbb {R}},\vec {\xi }\in {\mathbb {R}}^2$, let $K_0(\varepsilon ,t,u,\vec {\xi })$ denote the smallest integer exceeding $K_1(\varepsilon )$ such that (3.55)–(3.57) hold with probability at least $1-\varepsilon$ for $K\ge K_0(\varepsilon )$ and $n\ge n_0(K,\varepsilon ,t,u,\vec {\xi })$. Let $K_0(\varepsilon )$ and $n_0(\varepsilon )$ denote the maxima of $K_0(\varepsilon ,t,u,\vec {\xi })$ and $n_0(K_0(\varepsilon ),\varepsilon ,t,u,\vec {\xi })$, respectively, over all numbers $t,u\in [{\underline{t}}(\varepsilon ),{\overline{t}}(\varepsilon )]$ and $\xi _1,\xi _2\in [-1/\varepsilon ,1/\varepsilon ]$ that are dyadic rationals of the form $i2^{-\ell }$ ($i\in {\mathbb {Z}}$, $\ell \in {\mathbb {N}}$) with $\ell < 1/\varepsilon$.

The hypotheses imply that $K_0(\varepsilon )$ and $n_0(\varepsilon )$ are finite for each $\varepsilon >0$. Moreover, by construction, ${\overline{t}}(\varepsilon )\rightarrow \infty$, ${\underline{t}}(\varepsilon )\rightarrow -\infty$ as $\varepsilon \downarrow 0$. Therefore, by letting $\varepsilon =\varepsilon _n$ decrease to 0 sufficiently slowly as $n\rightarrow \infty$ and setting $K=K_0(\varepsilon _n)$, we can assume that $Z^*_n=Z^{\prime \scriptscriptstyle (K)}_n+Z^{\prime \prime \scriptscriptstyle (K)}_n$ where

8.16 $$\begin{aligned}&{\mathbb {E}}\left( \left. {\hat{Z}}^{\prime \scriptscriptstyle (K)}_{n,t}(\vec {\xi }) \,\right| {\mathscr {F}}'_n\right) {\mathop {\longrightarrow }\limits ^{\scriptscriptstyle {{\mathbb {P}}}}}q(t) {\hat{Q}}(\vec {\xi }), \end{aligned}$$

8.17 $$\begin{aligned}&{\mathbb {E}}\left( \left. \Big ( \frac{{\hat{Z}}^{\prime \scriptscriptstyle (K)}_{n,t}(\vec {\xi })}{q(t){\hat{Q}}(\vec {\xi })}-\frac{\left| Z^{\prime \scriptscriptstyle (K)}_{n,u}\right| }{q(u)} \Big )^2 \,\right| {\mathscr {F}}'_n \right) {\mathop {\longrightarrow }\limits ^{\scriptscriptstyle {{\mathbb {P}}}}}0, \end{aligned}$$

8.18 $$\begin{aligned}&{\mathbb {E}}\left( \left. \left| Z^{\prime \prime \scriptscriptstyle (K)}_{n,t}\right| \,\right| {\mathscr {F}}'_n\right) {\mathop {\longrightarrow }\limits ^{\scriptscriptstyle {{\mathbb {P}}}}}0, \end{aligned}$$

whenever $t,u,\xi _1,\xi _2$ are dyadic rationals.

Let ${\mathcal {P}}'_n,{\mathcal {P}}''_n$ denote the Cox processes with intensity measures $Z^{\prime \scriptscriptstyle (K)}_n,Z^{\prime \prime \scriptscriptstyle (K)}_n$, respectively. For any fixed $t\in {\mathbb {R}}$, (8.18) implies that the first point of ${\mathcal {P}}''_n$ does not occur by time *t* with high probability. By Proposition 8.5, it therefore suffices to show that $Z^{\prime \scriptscriptstyle (K)}_n$ satisfies Condition 8.1 for the family of functions $h(\vec {x})={\mathrm e}^{\vec {\xi }\cdot \vec {x}}$, $\vec {\xi }\in {\mathbb {R}}^2$.

Applying Chebyshev’s inequality to (8.17),

8.19 $$\begin{aligned} {\hat{Z}}^{\prime \scriptscriptstyle (K)}_{n,t}(\vec {\xi })-\frac{q(t)}{q(u)}{\hat{Q}}(\vec {\xi })\left| Z^{\prime \scriptscriptstyle (K)}_{n,u}\right| {\mathop {\longrightarrow }\limits ^{\scriptscriptstyle {{\mathbb {P}}}}}0 \end{aligned}$$

(a continuity argument extends the convergence from dyadic rationals to all $t,u,\vec {\xi }$). Taking $t=u$ verifies Condition 8.1 (a). For Condition 8.1 (b), fix $\varepsilon >0$ and choose ${\underline{t}}\in {\mathbb {R}}$ such that $q({\underline{t}})<\tfrac{1}{4}\varepsilon ^2$. Then (8.16) implies that ${\mathbb {E}}\left( \left. \left| Z^{\prime \scriptscriptstyle (K)}_{n,{\underline{t}}}\right| \,\right| {\mathscr {F}}_n\right) \le \tfrac{1}{2}\varepsilon ^2$ whp, and Markov’s inequality implies that ${\mathbb {P}}\left( \left. \left| Z^{\prime \scriptscriptstyle (K)}_{n,{\underline{t}}}\right| \ge \varepsilon \,\right| {\mathscr {F}}_n\right) \le \tfrac{1}{2}\varepsilon$ whp. Condition 8.1 (c) follows from (8.18) and assumption (b) in Theorem 3.29.

Finally, let $\varepsilon >0$ and a compact interval $[{\underline{t}},{\overline{t}}]$ be given. Expanding the interval if necessary, we may assume that ${\underline{t}},{\overline{t}}$ are dyadic rationals, and, decreasing $\varepsilon$ if necessary, we may assume that ${\overline{t}}\le {\overline{t}}(\varepsilon )$. Since *q* is continuous and non-decreasing, we may choose a partition $t_0<\cdots <t_N$ of $[{\underline{t}},{\overline{t}}]$ consisting of dyadic rationals such that

8.20 $$\begin{aligned} \sum _{i=1}^N \left( \frac{q(t_i)}{q(t_{i-1})}-1 \right) ^2 \le \frac{\varepsilon }{4z_0(\varepsilon )^2}. \end{aligned}$$

[it is enough to choose the partition finely enough that $\max _i (q(t_i)-q(t_{i-1})) \le q({\underline{t}})/4z_0(\varepsilon )^2 q({\overline{t}})$], and bound

8.21 $$\begin{aligned} \sum _{i=1}^N \left| \Delta Z^{\prime \scriptscriptstyle (K)}_{n,i}\right| ^2&= \sum _{i=1}^N \left( \big \vert Z^{\prime \scriptscriptstyle (K)}_{n,t_i}\big \vert -\frac{q(t_i)}{q(t_{i-1})} \big \vert Z^{\prime \scriptscriptstyle (K)}_{n,t_{i-1}}\big \vert + \Bigl ( \frac{q(t_i)}{q(t_{i-1})} - 1 \Bigr ) \big \vert Z^{\prime \scriptscriptstyle (K)}_{n,t_{i-1}}\big \vert \right) ^2 \nonumber \\&\le \sum _{i=1}^N 2 \left( \big \vert Z^{\prime \scriptscriptstyle (K)}_{n,t_i}\big \vert -\frac{q(t_i)}{q(t_{i-1})} \big \vert Z^{\prime \scriptscriptstyle (K)}_{n,t_{i-1}}\big \vert \right) ^2 + 2 \left( \frac{q(t_i)}{q(t_{i-1})} - 1 \right) ^2 \big \vert Z^{\prime \scriptscriptstyle (K)}_{n,t_{i-1}}\big \vert ^2 \nonumber \\&\le \frac{\varepsilon }{2z_0(\varepsilon )^2} \left| Z^*_{n,{\overline{t}}(\varepsilon )}\right| ^2 + 2 \sum _{i=1}^N \left( \big \vert Z^{\prime \scriptscriptstyle (K)}_{n,t_i}\big \vert -\frac{q(t_i)}{q(t_{i-1})} \big \vert Z^{\prime \scriptscriptstyle (K)}_{n,t_{i-1}}\big \vert \right) ^2 . \end{aligned}$$

The latter sum is $o_{\scriptscriptstyle {{\mathbb {P}}}}(1)$ by (8.19) with $\vec {\xi }=\vec {0}$, and the remaining term is at most $\varepsilon /2$ on the event $\big \{\vert Z^*_{n,{\overline{t}}(\varepsilon )}\vert \le z_0(\varepsilon )\big \}$. This event has probability at least $1-\varepsilon -o(1)$ by (8.15), which completes the proof. $\square$

## Moment Estimates and the Cluster After Unfreezing

While most other sections did not rely on the companion paper [21], in this section we *do* heavily rely on it. In particular, we make use of [Part I, Theorem 2.15], [Part I, Lemma 2.18], [Part I, Proposition 2.17] and [Part I, Lemma 6.4].

In this section we study ${\mathcal {B}}_t$ for $t\ge T_\mathrm{unfr}$, when the cluster resumes its CTBP behaviour. We will use moment methods to prove Lemma 3.32 and Theorem 3.31, completing the proof of our results.

This section is organised as follows. We start in Sect. 9.1 with some preparations concerning frozen intensity measures. In Sect. 9.2, we investigate the volume of the cluster after unfreezing and prove Lemma 3.32 (a). In Sect. 9.3 we use second moment methods on the collision edges to prove Theorem 3.31. In Sect. 9.4 we show that the collision edge whp does not originate from the frozen cluster, but rather from one of its descendants, to prove Lemma 3.32 (b). Finally, in Sect. 9.5, we study the freezing time and frozen cluster and use this to prove Theorem 3.18.

### Preparations: Frozen Intensity Measures

We introduce the frozen intensity measures

9.1 $$\begin{aligned} d\mu _{n,\mathrm{fr}}^{\scriptscriptstyle (j)}(y) = \sum _{v\in {\mathcal {B}}_\mathrm{fr}^{\scriptscriptstyle (j)}} {\mathbb {1}}_{\left\{ y\ge 0\right\} } \mu _n\bigl (T_\mathrm{fr}^{\scriptscriptstyle (j)}-T_v + dy\bigr ). \end{aligned}$$

Recall that the notation $\mu (t_0+dy)$ denotes the translation of the measure $\mu$ by $t_0$; thus (9.1) means that, for a test function $h\ge 0$,

9.2 $$\begin{aligned} \int h(y) d\mu _{n,\mathrm{fr}}^{\scriptscriptstyle (j)}(y) = \sum _{v\in {\mathcal {B}}_\mathrm{fr}^{\scriptscriptstyle (j)}} \int _{T_\mathrm{fr}^{\scriptscriptstyle (j)}-T_v}^\infty h\left( y-(T_\mathrm{fr}^{\scriptscriptstyle (j)}-T_v) \right) d\mu _n(y). \end{aligned}$$

#### Lemma 9.1

Almost surely, for $j=1,2,$

9.3 $$\begin{aligned} s_n\le \int {\mathrm e}^{-\lambda _n(1) y} d\mu _{n,\mathrm{fr}}^{\scriptscriptstyle (j)}(y) \le s_n+1. \end{aligned}$$

#### Proof

By (9.2),

9.4 $$\begin{aligned} \int {\mathrm e}^{-\lambda _n y} d\mu _{n,\mathrm{fr}}^{\scriptscriptstyle (j)}(y)=\sum _{v\in {\mathcal {B}}_\mathrm{fr}^{\scriptscriptstyle (j)}} \int _{T_\mathrm{fr}^{\scriptscriptstyle (j)}-T_v}^\infty {\mathrm e}^{-\lambda _n\left( y-(T_\mathrm{fr}^{\scriptscriptstyle (j)}-T_v)\right) } d\mu _n(y). \end{aligned}$$

The expression in (9.4) is the value of the process $\sum _{v\in \mathsf{BP}_t^{\scriptscriptstyle (j)}}\int _{t-T_v}^\infty {\mathrm e}^{-\lambda _n(y-(t-T_v))} d\mu _n(y)$ from Definition 3.17, stopped at $t=T_\mathrm{fr}^{\scriptscriptstyle (j)}$ (recall that ${\mathcal {B}}_\mathrm{fr}^{\scriptscriptstyle (j)}=\mathsf{BP}_{T_\mathrm{fr}^{\scriptscriptstyle (j)}}^{\scriptscriptstyle (j)}$). Since $\mu _n$ has no atoms, this process is continuous in *t* except for jumps at the birth times, and since the birth times are distinct a.s., the corresponding jump has size $\int _0^\infty {\mathrm e}^{-\lambda _ny}d\mu _n(y)=1$. By definition, $T_\mathrm{fr}^{\scriptscriptstyle (j)}$ is the first time the process in (9.4) exceeds $s_n$, so it can have value at most $s_n+1$ at that time. $\square$

For future reference, we now state a lemma, to be used in Sect. 9.3, showing that most of the mass of the frozen intensity measures $\mu _{n,\mathrm{fr}}^{\scriptscriptstyle (j)}$ comes from small times:

#### Lemma 9.2

Let $\delta ,\delta '>0$ and $a_0>0$ be given. Then there exists $K<\infty$ and $n_0 \in {\mathbb {N}}$ such that, for all $a\ge a_0$ and $n\ge n_0,$

9.5 $$\begin{aligned} {\mathbb {P}}\left( \int {\mathrm e}^{-\lambda _n(a^{1/s_n})y} {\mathbb {1}}_{\left\{ \lambda _n(1)y\ge K\right\} } d\mu _{n,\mathrm{fr}}^{\scriptscriptstyle (j)}(y) >\delta s_n \right) \le \delta '. \end{aligned}$$

#### Proof

Let $\varepsilon =a_0 {\mathrm e}^{-\gamma }/2$, where $\gamma$ denotes Euler’s constant. Using the definition of $\mu _{n,\mathrm{fr}}^{\scriptscriptstyle (j)}$ from (9.1), the monotonicity of $\lambda _n(\cdot )$ and (6.15), we obtain $n_0 \in {\mathbb {N}}$ such that for all $K<\infty$, $a\ge a_0$ and $n\ge n_0$,

9.6 $$\begin{aligned} \int {\mathrm e}^{-\lambda _n({\tilde{a}})y} {\mathbb {1}}_{\left\{ \lambda _n(1)y\ge K\right\} } d\mu _{n,\mathrm{fr}}^{\scriptscriptstyle (j)}(y) \le \sum _{v \in {\mathcal {B}}_\mathrm{fr}^{\scriptscriptstyle (j)}} \int {\mathrm e}^{-\varepsilon y/f_n(1)} {\mathbb {1}}_{\left\{ y\ge Kf_n(1)\right\} } \mu _n\left( T_\mathrm{fr}^{\scriptscriptstyle (j)}-T_v+dy\right) . \end{aligned}$$

According to Lemma 4.4, for any $\varepsilon '>0$ we can choose some $K<\infty$ such that, after possibly increasing $n_0$, the right-hand side of (9.6) is bounded from above by $\left| {\mathcal {B}}_\mathrm{fr}^{\scriptscriptstyle (j)}\right| \varepsilon '/s_n$. Since $\left| {\mathcal {B}}_\mathrm{fr}^{\scriptscriptstyle (j)}\right| =O_{\mathbb {P}}(s_n^2)$ by Theorem 3.18 (b), the proof is complete. $\square$

### A First Moment Estimate: Proof of Lemma 3.32 (a)

In this section we show how to express ${\mathcal {B}}_t\setminus {\mathcal {B}}_\mathrm{fr}$, $t\ge T_\mathrm{unfr}$, as a suitable union of branching processes. This representation leads to a simple proof of Lemma 3.32 (a). We will also use it in Sect. 9.3 to prove Theorem 3.31.

Consider the immediate children $v\in \partial {\mathcal {B}}_\mathrm{fr}$ of individuals in the frozen cluster ${\mathcal {B}}_\mathrm{fr}$. Then, for $t'\ge 0$,

9.7 $$\begin{aligned} {\mathcal {B}}_{T_\mathrm{unfr}+t'}\setminus {\mathcal {B}}_\mathrm{fr}= \bigcup _{v\in \partial {\mathcal {B}}_\mathrm{fr}:T_v^{\mathcal {B}}\le T_\mathrm{unfr}+t'} \left\{ vw:w\in \mathsf{BP}^{\scriptscriptstyle (v)}_{t'+T_\mathrm{unfr}-T_v^{\mathcal {B}}}\right\} , \end{aligned}$$

where $\mathsf{BP}^{\scriptscriptstyle (v)}$ denotes the branching process of descendants of *v*, re-rooted and time-shifted as in (6.1). Furthermore, conditionally on ${\mathcal {B}}_\mathrm{fr}$, the children $v\in \partial {\mathcal {B}}_\mathrm{fr}$ appear according to a Cox process. Formally, the point measures

9.8 $$\begin{aligned} {\mathcal {P}}_{n,\mathrm{unfr}}^{\scriptscriptstyle (j)}=\sum _{v\in \partial {\mathcal {B}}_\mathrm{fr}^{(j)}} \delta _{(T_v^{\mathcal {B}}-T_\mathrm{unfr},\mathsf{BP}^{(v)})} \end{aligned}$$

form Cox processes with intensities $d\mu _{n,\mathrm{fr}}^{\scriptscriptstyle (j)} \otimes d{\mathbb {P}}(\mathsf{BP}^{\scriptscriptstyle (1)}\in \cdot )$, $j=1,2$, where the frozen intensity measures $\mu _{n,\mathrm{fr}}^{\scriptscriptstyle (j)}$ were introduced in (9.1).

#### Proof of Lemma 3.32 (a)

By Theorem 3.18 (b), the volume $\left| {\mathcal {B}}_\mathrm{fr}\right|$ of the frozen cluster is $O_{\mathbb {P}}(s_n^2)$, and this is $o_{\mathbb {P}}(\sqrt{n s_n})$ since $n/s_n^3\rightarrow \infty$. It therefore suffices to show that $\left| {\mathcal {B}}_{{\overline{t}}}\setminus {\mathcal {B}}_\mathrm{fr}\right| =O_{\mathbb {P}}(\sqrt{n s_n})$ when ${\overline{t}}=T_\mathrm{unfr}+\lambda _n(1)^{-1}\left( \tfrac{1}{2}\log (n/s_n^3)+K\right)$.

Abbreviate $$t'={\overline{t}}-T_\mathrm{unfr}=\lambda _n(1)^{-1}\left( \tfrac{1}{2}\log (n/s_n^3)+K\right) $$. By (9.7)–(9.8),

9.9 $$\begin{aligned} \left| {\mathcal {B}}_{{\overline{t}}}\setminus {\mathcal {B}}_\mathrm{fr}\right|&= \sum _{v\in \partial {\mathcal {B}}_\mathrm{fr}:T_v^{\mathcal {B}}\le T_\mathrm{unfr}+t'} z^{1,\mathsf{BP}^{(v)}}_{t'+T_\mathrm{unfr}-T_v^{\mathcal {B}}}(1) = \sum _{j=1}^2 \int {\mathbb {1}}_{\left\{ t\le t'\right\} } z^{1,bp}_{t'-t}(1) d{\mathcal {P}}_{n,\mathrm{unfr}}^{\scriptscriptstyle (j)}(t,bp) , \end{aligned}$$

so that, by Theorem 3.13 and Lemma 9.1, there exists a $K'<\infty$ such that for sufficiently large *n*,

9.10 $$\begin{aligned} {\mathbb {E}}\left( \left. \big .\left| {\mathcal {B}}_{{\overline{t}}}\setminus {\mathcal {B}}_\mathrm{fr}\right| \,\right| {\mathcal {B}}_\mathrm{fr}\right)&= \sum _{j=1}^2 \int {\mathbb {1}}_{\left\{ t\le t'\right\} } {\mathrm e}^{\lambda _n(1)(t'-t)} {\bar{m}}_{t'-t}^1(1) d\mu _{n,\mathrm{fr}}^{\scriptscriptstyle (j)}(t) \nonumber \\&\le \sqrt{\frac{n}{s_n^3}}{\mathrm e}^K \sum _{j=1}^2 \int {\mathbb {1}}_{\left\{ t\le t'\right\} } K' s_n {\mathrm e}^{-\lambda _n(1)t} d\mu _{n,\mathrm{fr}}^{\scriptscriptstyle (j)}(t) \le K' {\mathrm e}^K \sqrt{\frac{n}{s_n^3}}s_n(s_n+1). \end{aligned}$$

Markov’s inequality completes the proof. $\square$

### Second Moment Estimates: Proof of Theorem 3.31

In this section we prove that ${\mathcal {P}}_n^*$ satisfies the assumptions of Theorem 3.29. Namely, we will split $\mu _n=\mu _n^{\scriptscriptstyle (K)}+(\mu _n-\mu _n^{\scriptscriptstyle (K)})$ into the truncated measure and a remainder, as in Sect. 7. This induces a splitting of the intensity measure into $Z^{\prime \scriptscriptstyle (K)}_n+Z^{\prime \prime \scriptscriptstyle (K)}_n$, and the hypothesis (a) will be verified using the estimates for the two-vertex characteristics $\chi _n^{\scriptscriptstyle (K)}$ and $\chi _n-\chi _n^{\scriptscriptstyle (K)}$ in Theorems 3.15 and 3.16. The remaining hypothesis (b) will be proved using a separate argument.

Throughout the proof, the times *t* and $t^*$ are related as in (3.60), and we recall from (3.54) that, for a measure *Q* on ${\mathbb {R}}^d$, we write ${\hat{Q}}$ for its moment generating function.

#### Proof of Theorem 3.31

Since ${\mathcal {P}}_n^*$ is the image of a Cox process under a mapping that is measurable with respect to ${\mathcal {B}}_\mathrm{fr}$, it is immediate that it itself is a Cox process, and its intensity measure is

9.11 $$\begin{aligned} Z_{n,t^*}^* {=} \sum _{v_1\in {\mathcal {B}}_t^{\scriptscriptstyle (1)}{\setminus }{\mathcal {B}}_\mathrm{fr}^{\scriptscriptstyle (1)}} \sum _{v_2\in {\mathcal {B}}_t^{\scriptscriptstyle (2)}\setminus {\mathcal {B}}_\mathrm{fr}^{\scriptscriptstyle (2)}} \tfrac{1}{n} \mu _n\bigl (\Delta R_{v_1,v_2},R_1(t)-R_1\left( T_{v_1}^{\mathcal {B}}\right) +R_2(t)-R_2\left( T_{v_2}^{\mathcal {B}}\right) \bigr ) \delta _{(\left| v_1\right| ^*,\left| v_2\right| ^*)}, \end{aligned}$$

where we recall $\left| v\right| ^*$ from (3.59) and $\left| v_j\right| ^*$ denotes $\left| v\right| ^*$ for ${\mathcal {B}}^{\scriptscriptstyle (j)}$. In the above sum, $T_{v_j}^{\mathcal {B}}\ge T_\mathrm{unfr}$, so that $R_{j'}(t)-R_{j'}(T_{v_j}^{\mathcal {B}})=t-T_{v_j}^{\mathcal {B}}$ whenever $t\ge T_{v_j}^{\mathcal {B}}$, $j,j'\in \left\{ 1,2\right\}$, and, recalling (3.49), $\Delta R_{v_1,v_2}=\left| T_{v_1}^{\mathcal {B}}-T_{v_2}^{\mathcal {B}}\right|$.

We begin by expressing ${\hat{Z}}^*_{n,t^*}(\vec {\xi })$ as a sum of two-vertex characteristics. As in (9.7), any vertex $v'\in {\mathcal {B}}_t\setminus {\mathcal {B}}_\mathrm{fr}$ is descended from a unique vertex $v=p^\mathrm{unfr}(v')\in \partial {\mathcal {B}}_\mathrm{fr}$ and can therefore be written as $v'=vw$ for some $w\in \mathsf{BP}^{\scriptscriptstyle (v)}_{t-T_v^{\mathcal {B}}}$. Hence $\left| v'\right| -\left| p^\mathrm{unfr}(v')\right| =\left| vw\right| -\left| v\right| =\left| w\right|$. Thus

9.12 $$\begin{aligned}&{\hat{Z}}^*_{n,t^*}(\vec {\xi })\nonumber \\&\quad = \sum _{v_1\in \partial {\mathcal {B}}_\mathrm{fr}^{\scriptscriptstyle (1)}} \sum _{w_1\in \mathsf{BP}^{\scriptscriptstyle (v_1)}_{t-T_{v_1}^{\mathcal {B}}}} \sum _{v_2\in \partial {\mathcal {B}}_\mathrm{fr}^{\scriptscriptstyle (2)}} \sum _{w_2\in \mathsf{BP}^{\scriptscriptstyle (v_2)}_{t-T_{v_2}^{\mathcal {B}}}}\nonumber \\&\qquad \exp \left( \frac{\xi _1 \left| w_1\right| +\xi _2\left| w_2\right| }{s_n\sqrt{\log (n/s_n^3)}} - (\xi _1+\xi _2)\frac{\phi _n}{2s_n}\sqrt{\log (n/s_n^3)}\right) \nonumber \\&\qquad \times \tfrac{1}{n}\mu _n\left( \left| T_{v_1 w_1}^{\mathcal {B}}-T_{v_2 w_2}^{\mathcal {B}}\right| , t-T_{v_1 w_1}^{\mathcal {B}}+t-T_{v_2 w_2}^{\mathcal {B}}\right) . \end{aligned}$$

We note that $T_{v_j w_j}^{\mathcal {B}}=T_{v_j}^{\mathcal {B}}+T^{\scriptscriptstyle (v_j)}_{w_j}$, where $T^{\scriptscriptstyle (v_j)}_{w_j}$ denotes the birth time of $w_j$ in the branching process $\mathsf{BP}^{\scriptscriptstyle (v_j)}$ defined in (6.1). (Note that $T_{v_j}^{{\mathcal {B}}}\ge T_\mathrm{unfr}$, so that freezing plays no role after $T_{v_j}^{\mathcal {B}}$ and we need not consider $T_{w_j}^{\mathcal {B}}$.) It follows that

9.13 $$\begin{aligned} \mu _n\left( \left| T_{v_1 w_1}^{\mathcal {B}}-T_{v_2 w_2}^{\mathcal {B}}\right| , t-T_{v_1 w_1}^{\mathcal {B}}+t-T_{v_2 w_2}^{\mathcal {B}}\right) = \chi _n\left( t-T_{v_1}^{\mathcal {B}}-T^{\scriptscriptstyle (v_1)}_{w_1}, t-T_{v_2}^{\mathcal {B}}-T^{\scriptscriptstyle (v_2)}_{w_2}\right) , \end{aligned}$$

where $\chi _n$ is the two-vertex characteristic from (3.24). Recalling the notation from (3.22),

9.14 $$\begin{aligned} {\hat{Z}}^*_{n,t^*}(\vec {\xi }) = \frac{1}{n}\exp \left( -(\xi _1+\xi _2)\frac{\phi _n}{2s_n}\sqrt{\log (n/s_n^3)} \right) \sum _{v_1\in \partial {\mathcal {B}}_\mathrm{fr}^{\scriptscriptstyle (1)}} \sum _{v_2\in \partial {\mathcal {B}}_\mathrm{fr}^{\scriptscriptstyle (2)}} z^{\chi _n,\mathsf{BP}^{(v_1)},\mathsf{BP}^{(v_2)}}_{t-T_{v_1}^{\mathcal {B}}\! , \, t-T_{v_2}^{\mathcal {B}}}({\tilde{\vec {a}}}) , \end{aligned}$$

where ${\tilde{\vec {a}}}=(a_1^{1/s_n},a_2^{1/s_n})$ as in (7.3) and

9.15 $$\begin{aligned} a_j=\exp \left( \frac{\xi _j}{\sqrt{\log (n/s_n^3)}} \right) \! . \end{aligned}$$

Note that $a_1,a_2$ depend implicitly on *n* and $a_j\rightarrow 1$ as $n\rightarrow \infty$.

As in Sect. 9.2, we express the sums over $\partial {\mathcal {B}}_\mathrm{fr}^{\scriptscriptstyle (1)}$, $\partial {\mathcal {B}}_\mathrm{fr}^{\scriptscriptstyle (2)}$ in (9.14) in terms of the point measures ${\mathcal {P}}_{n,\mathrm{unfr}}^{\scriptscriptstyle (j)}=\sum _{v\in \partial {\mathcal {B}}_\mathrm{fr}^{\scriptscriptstyle (j)}} \delta _{(T_v^{\mathcal {B}}-T_\mathrm{unfr},\mathsf{BP}^{\scriptscriptstyle (v)})}$ from (9.8):

9.16 $$\begin{aligned} {\hat{Z}}^*_{n,t^*}(\vec {\xi })&= \frac{1}{n}\exp \left( -(\xi _1+\xi _2)\frac{\phi _n}{2s_n}\sqrt{\log (n/s_n^3)} \right) \int d{\mathcal {P}}_{n,\mathrm{unfr}}^{\scriptscriptstyle (1)}(t_1,bp^{\scriptscriptstyle (1)}) \nonumber \\&\quad \times \int d{\mathcal {P}}_{n,\mathrm{unfr}}^{\scriptscriptstyle (2)}(t_2,bp^{\scriptscriptstyle (2)}) z^{\chi _n, bp^{(1)} \! , \, bp^{(2)}}_{t-T_\mathrm{unfr}-t_1, t-T_\mathrm{unfr}-t_2}({\tilde{\vec {a}}}) . \end{aligned}$$

Recalling the notation from (3.23) and (6.19),

9.17 $$\begin{aligned}&{\hat{Z}}^*_{n,t^*}(\vec {\xi }) = \frac{1}{n}\exp \left( -(\xi _1+\xi _2)\frac{\phi _n}{2s_n}\sqrt{\log (n/s_n^3)}+ (t-T_\mathrm{unfr})(\lambda _n({\tilde{a}}_1)+\lambda _n({\tilde{a}}_2)) \right) \nonumber \\&\qquad \times \int d{\mathcal {P}}_{n,\mathrm{unfr}}^{\scriptscriptstyle (1)}(t_1,bp^{\scriptscriptstyle (1)}) \int d{\mathcal {P}}_{n,\mathrm{unfr}}^{\scriptscriptstyle (2)}(t_2,bp^{\scriptscriptstyle (2)}) {\mathrm e}^{-\lambda _n({\tilde{a}}_1)t_1-\lambda _n({\tilde{a}}_2)t_2} {\bar{z}}^{\chi _n, bp^{(1)}, bp^{(2)}}_{t-T_\mathrm{unfr}-t_1 \! , \, t-T_\mathrm{unfr}-t_2}({\tilde{\vec {a}}}) \nonumber \\&\quad = \exp \left( \frac{\lambda _n({\tilde{a}}_1)+\lambda _n({\tilde{a}}_2)}{\lambda _n(1)}\left( t^*+\tfrac{1}{2}\log (n/s_n^3) \right) -(\xi _1+\xi _2)\frac{\phi _n}{2s_n}\sqrt{\log (n/s_n^3)} - \log (n/s_n^3) \right) \nonumber \\&\qquad \times \frac{1}{s_n^3} \int d{\mathcal {P}}_{n,\mathrm{unfr}}^{\scriptscriptstyle (1)}(t_1,bp^{\scriptscriptstyle (1)}) \int d{\mathcal {P}}_{n,\mathrm{unfr}}^{\scriptscriptstyle (2)}(t_2,bp^{\scriptscriptstyle (2)}) {\mathrm e}^{-\lambda _n({\tilde{a}}_1)t_1-\lambda _n({\tilde{a}}_2)t_2} {\bar{z}}^{\chi _n, bp^{(1)}, bp^{(2)}}_{t-T_\mathrm{unfr}-t_1 \! , \, t-T_\mathrm{unfr}-t_2}({\tilde{\vec {a}}}) . \end{aligned}$$

For the non-random factors on the right-hand side of (9.17), we use the asymptotics from Corollary 6.5:

9.18 $$\begin{aligned} \frac{\lambda _n({\tilde{a}}_j)}{\lambda _n(1)}&= 1+\frac{\phi _n}{s_n}\left( \exp \left( \xi _j/\sqrt{\log (n/s_n^3)} \right) -1\right) +o\left( \exp \left( \xi _j/\sqrt{\log (n/s_n^3)} \right) -1 \right) ^2 \nonumber \\&= 1+\frac{\phi _n}{s_n}\left( \xi _j/\sqrt{\log (n/s_n^3)} + \frac{1}{2}\xi _j^2/\log (n/s_n^3)\right) + o\left( 1/\log \left( n/s_n^3\right) \right) . \end{aligned}$$

Combining with the asymptotics $\lambda _n({\tilde{a}}_j)/\lambda _n(1)=1+o(1)$ and $s_n/\phi _n=1+o(1)$ [see (6.15) and Lemma 3.12], we conclude that, for any fixed $t^* \in {\mathbb {R}}$,

9.19 $$\begin{aligned} \frac{\lambda _n({\tilde{a}}_j)}{\lambda _n(1)}\bigl ( t^*+\tfrac{1}{2}\log (n/s_n^3) \bigr ) -\xi _j\frac{\phi _n}{2s_n}\sqrt{\log (n/s_n^3)}-\frac{1}{2}\log (n/s_n^3) =t^*+\tfrac{1}{4}\xi _j^2 +o(1) . \end{aligned}$$

[When $\xi _j=0$, $a_j=1$, the term *o*(1) in (9.19) is absent.] Combining (9.17) and (9.19),

9.20 $$\begin{aligned} {\hat{Z}}^*_{n,t^*}(\vec {\xi })&= \frac{{\mathrm e}^{2t^*+\frac{1}{4}\left\| \vec {\xi }\right\| ^2+o(1)}}{s_n^3} \int d{\mathcal {P}}_{n,\mathrm{unfr}}^{\scriptscriptstyle (1)}(t_1,bp^{\scriptscriptstyle (1)}) \int d{\mathcal {P}}_{n,\mathrm{unfr}}^{\scriptscriptstyle (2)}(t_2,bp^{\scriptscriptstyle (2)}) \nonumber \\&\quad \cdot {\mathrm e}^{-\lambda _n({\tilde{a}}_1)t_1} {\mathrm e}^{-\lambda _n({\tilde{a}}_2)t_2} {\bar{z}}_{t-T_\mathrm{unfr}-t_1 \! , \, t-T_\mathrm{unfr}-t_2}^{\chi _n,bp^{(1)},bp^{(2)}}({\tilde{\vec {a}}}) . \end{aligned}$$

This is the desired representation in terms of two-vertex characteristics.

Given $$K<\infty $$, define

9.21 $$\begin{aligned} Z^{\prime \scriptscriptstyle (K)}_{n,t^*} = \sum _{v_1\in {\mathcal {B}}_t^{\scriptscriptstyle (1)}\setminus {\mathcal {B}}_\mathrm{fr}^{\scriptscriptstyle (1)}} \sum _{v_2\in {\mathcal {B}}_t^{\scriptscriptstyle (2)}\setminus {\mathcal {B}}_\mathrm{fr}^{\scriptscriptstyle (2)}} \tfrac{1}{n} \mu _n^{\scriptscriptstyle (K)}\left( \left| T_{v_1}^{\mathcal {B}}-T_{v_2}^{\mathcal {B}}\right| ,t-T_{v_1}^{\mathcal {B}}+t-T_{v_2}^{\mathcal {B}}\right) \delta _{(\left| v_1\right| ^*,\left| v_2\right| ^*)}, \end{aligned}$$

similarly to Definition 3.30, and set $Z^{\prime \prime \scriptscriptstyle (K)}_{n,t^*}=Z_{n,t^*}^*-Z^{\prime \scriptscriptstyle (K)}_{n,t^*}$. (We suppress the dependence of $Z^{\prime \scriptscriptstyle (K)}_n,Z^{\prime \prime \scriptscriptstyle (K)}_n$ on *K*.) Clearly, (9.20) remains true when $Z_{n,t^*}^*$ and $\chi _n$ are replaced by $Z^{\prime \scriptscriptstyle (K)}_{n,t^*}$ and $\chi _n^{\scriptscriptstyle (K)}$, or by $Z^{\prime \prime \scriptscriptstyle (K)}_{n,t^*}$ and $\chi _n-\chi _n^{\scriptscriptstyle (K)}$, respectively. We will use (9.20) to control first and second moments of $Z^{\prime \scriptscriptstyle (K)}_{n,t^*}$, for arbitrary but fixed values $t^*,\xi _1,\xi _2\in {\mathbb {R}}$, and first moments of $Z^{\prime \prime \scriptscriptstyle (K)}_{n,t^*}$ with $\xi _1=\xi _2=0$.

**Verification of** (3.55): Writing $\lambda _n({\tilde{\vec {a}}})=(\lambda _n({\tilde{a}}_1),\lambda _n({\tilde{a}}_2))$, we obtain from (9.20),

9.22 $$\begin{aligned}&{\mathbb {E}}\left( \left. {\hat{Z}}'_{n,t^*}(\vec {\xi })\,\right| {\mathcal {B}}_\mathrm{fr}\right) = \frac{{\mathrm e}^{2t^*+\frac{1}{4}\left\| \vec {\xi }\right\| ^2+o(1)}}{s_n^2}\iint {\mathrm e}^{-\lambda _n({\tilde{\vec {a}}})\cdot \vec {t}} \frac{{\bar{m}}^{\chi _n^{\scriptscriptstyle (K)}}_{t-T_\mathrm{unfr}-\vec {t}}({\tilde{\vec {a}}})}{s_n} d\mu _{n,\mathrm{fr}}^{\scriptscriptstyle (1)}(t_1) d\mu _{n,\mathrm{fr}}^{\scriptscriptstyle (2)}(t_2) . \end{aligned}$$

Since $\lambda _n(1)(t-T_\mathrm{unfr})=\tfrac{1}{2}\log (n/s_n^3)+t^*\rightarrow \infty$ and $a_j\rightarrow 1$, Theorem 3.15 and Corollary 6.5 imply that for $\varepsilon \in (0,1)$ and $K<\infty$ sufficiently large, there exists $K'<\infty$ and $n_0 \in {\mathbb {N}}$ such that the integrand of (9.22) lies between ${\mathrm e}^{-\lambda _n(1)(t_1+t_2)-\varepsilon }(1-\varepsilon )$ and ${\mathrm e}^{-\lambda _n(1)(t_1+t_2)+\varepsilon }(1+\varepsilon )$ for $\lambda _n(1)[t_1 \vee t_2]\le K'$ and $n\ge n_0$, and there are constants $K''<\infty , n_0'\in {\mathbb {N}}$ such that it is bounded by $K'' {\mathrm e}^{-\lambda _n({\tilde{\vec {a}}})\cdot \vec {t}}$ for all $t_1,t_2\ge 0$, $n\ge n_0'$. Using Lemmas 9.1 and 9.2, it easily follows that

9.23 $$\begin{aligned} {\mathrm e}^{2t^*+\frac{1}{4}\left\| \vec {\xi }\right\| ^2}(1-2\varepsilon ) \le {\mathbb {E}}\left( \left. {\hat{Z}}'_{n,t^*}(\vec {\xi })\,\right| {\mathcal {B}}_\mathrm{fr}\right) \le {\mathrm e}^{2t^*+\frac{1}{4}\left\| \vec {\xi }\right\| ^2}(1+2\varepsilon ) \end{aligned}$$

whp. Since ${\mathrm e}^{\frac{1}{4}\left\| \vec {\xi }\right\| ^2}={\hat{Q}}(\vec {\xi })$, where *Q* is the law of two independent $N\left( 0,\tfrac{1}{2}\right)$ variables, we have therefore verified the first moment condition (3.55) of Theorem 3.29 with $q(t^*)={\mathrm e}^{2t^*}$.

**Verification of** (3.57): For $Z^{\prime \prime \scriptscriptstyle (K)}_{n,t^*}$, set $\vec {\xi }=\vec {0}:=(0,0)$. In (9.22), we can replace $Z^{\prime \scriptscriptstyle (K)}_{n,t^*}$ and $\chi _n^{\scriptscriptstyle (K)}$ by $Z^{\prime \prime \scriptscriptstyle (K)}_{n,t^*}$ and $\chi _n-\chi _n^{\scriptscriptstyle (K)}$, and Theorem 3.16 implies that, for any $\varepsilon \in (0,1)$ and large *K*, there is $K'<\infty$ such that the integrand of the resulting equation is at most $\varepsilon {\mathrm e}^{-\lambda _n(1)(t_1+t_2)}$, uniformly for $\lambda _n(1)[t_1 \vee t_2]\le K'$ for large *n*, and there is $K''<\infty$ such that it is bounded by $K''{\mathrm e}^{-\lambda _n(1)(t_1+t_2)}$ for all $t_1,t_2\ge 0$, *n* large. This verifies the first moment condition (3.57), as in the previous case.

**Verification of** (3.56): The second moment estimates for $Z^{\prime \scriptscriptstyle (K)}_{n,t^*}$, though somewhat more complicated, are similar in spirit. Note the importance of freezing, which is not so far apparent from the first moment calculations only: the freezing times $T_\mathrm{fr}^{\scriptscriptstyle (j)}$ and the rescaled times $t\approx T_\mathrm{unfr}+\tfrac{1}{2}\lambda _n(1)^{-1}\log (n/s_n^3)$ have exactly the scaling needed so that *both* the first and second moments of $Z^*_{n,t^*}$ will have order 1, even though ${\bar{m}}_{\vec {t}}^{\chi _n^{\scriptscriptstyle (K)}}({\tilde{\vec {a}}})\approx s_n$, ${\bar{M}}^{\chi _n^{\scriptscriptstyle (K)},\chi _n^{\scriptscriptstyle (K)}}_{\vec {t},\vec {u}}({\tilde{\vec {a}}},{\tilde{\vec {b}}})\approx s_n^4$ by Theorem 3.15.

Equation (9.20) for $Z_{n,t^*}'$ expresses ${\hat{Z}}'_{n,t^*}(\vec {\xi })$ in terms of a double integral with respect to a pair of Cox processes, whose intensity is measurable with respect to ${\mathcal {B}}_\mathrm{fr}$. An elementary calculation [applying (6.3) twice] shows that, for a pair ${\mathcal {P}}^{\scriptscriptstyle (1)},{\mathcal {P}}^{\scriptscriptstyle (2)}$ of Poisson point processes with intensities $\nu _1,\nu _2$,

9.24 $$\begin{aligned}&{\mathbb {E}}\left( \iint f(x_1,x_2) d{\mathcal {P}}^{\scriptscriptstyle (1)}(x_1)d{\mathcal {P}}^{\scriptscriptstyle (2)}(x_2) \iint g(y_1,y_2) d{\mathcal {P}}^{\scriptscriptstyle (1)}(y_1)d{\mathcal {P}}^{\scriptscriptstyle (2)}(y_2) \right) \nonumber \\&\quad = \iint f(x_1,x_2)g(x_1,x_2) d\nu _1(x_1)d\nu _2(x_2) + \iiint f(x_1,x_2) g(x_1,y_2) d\nu _1(x_1) d\nu _2(x_2) d\nu _2(y_2) \nonumber \\&\qquad + \iiint f(x_1,x_2) g(y_1,x_2) d\nu _1(x_1) d\nu _1(y_1) d\nu _2(x_2) \nonumber \\&\qquad + \iint f(x_1,x_2)d\nu _1(x_1)d\nu _2(x_2)\iint g(y_1,y_2)d\nu _1(y_1)d\nu _2(y_2). \end{aligned}$$

We apply (9.24) to (9.20). In the notation of (9.24), we have $x_j=(t_j,bp^{\scriptscriptstyle (j)})$, and $f(x_1,x_2)=g(x_1,x_2)={\mathrm e}^{-\lambda _n({\tilde{a}}_1)t_1} {\mathrm e}^{-\lambda _n({\tilde{a}}_2)t_2} {\bar{z}}_{t-T_\mathrm{unfr}-t_1 \! , \, t-T_\mathrm{unfr}-t_2}^{\chi ^{(K)}_n,bp^{(1)},bp^{(2)}}({\tilde{\vec {a}}})$. Integration against the intensity measure $d\nu _j=d\mu _{n,\mathrm{fr}}^{\scriptscriptstyle (j)} \otimes d{\mathbb {P}}(\mathsf{BP}^{\scriptscriptstyle (j)}\in \cdot )$ is equivalent to a branching process expectation together with an integration over $t_j$, and we will therefore obtain first or second moments of one- or two-vertex characteristics from the various terms in (9.24). Namely, for $\vec {\xi }, \vec {\zeta } \in {\mathbb {R}}^2$ and writing $b_j=\exp (\zeta _j/\sqrt{\log (n/s_n^3)})$, we obtain

9.25 $$\begin{aligned}&s_n^6 {\mathrm e}^{-2t^*-\frac{1}{4}\left\| \vec {\xi }\right\| ^2-2u^*-\frac{1}{4}\left\| \vec {\zeta }\right\| ^2-o(1)}{\mathbb {E}}\left( \left. {\hat{Z}}'_{n,t^*}(\vec {\xi }) {\hat{Z}}'_{n,u^*}(\vec {\zeta }) \,\right| {\mathcal {B}}_\mathrm{fr}\right) \nonumber \\&\quad = \iint {\mathrm e}^{-(\lambda _n({\tilde{a}}_1)+\lambda _n({\tilde{b}}_1))t_1} {\mathrm e}^{-(\lambda _n({\tilde{a}}_2)+\lambda _n({\tilde{b}}_2))t_2} {\bar{M}}_{t-T_\mathrm{unfr}-\vec {t},u-T_\mathrm{unfr}-\vec {t}}^{\chi _n^{\scriptscriptstyle (K)},\chi _n^{\scriptscriptstyle (K)}}\left( {\tilde{\vec {a}}},{\tilde{\vec {b}}}\right) d\mu _{n,\mathrm{fr}}^{\scriptscriptstyle (1)}(t_1) d\mu _{n,\mathrm{fr}}^{\scriptscriptstyle (2)}(t_2) \nonumber \\&\qquad + \iiint {\mathrm e}^{-(\lambda _n({\tilde{a}}_1)+\lambda _n({\tilde{b}}_1))t_1}{\mathrm e}^{-\lambda _n({\tilde{a}}_2)t_2}{\mathrm e}^{-\lambda _n({\tilde{b}}_2)u_2} {\bar{M}}_{t-T_\mathrm{unfr}-t_1,u-T_\mathrm{unfr}-t_1}^{\rho _{t-T_\mathrm{unfr}-t_2,{\tilde{a}}_2},\rho _{u-T_\mathrm{unfr}-u_2,{\tilde{b}}_2}}({\tilde{a}}_1,{\tilde{b}}_1) \nonumber \\&\qquad \qquad \qquad \qquad \qquad \times d\mu _{n,\mathrm{fr}}^{\scriptscriptstyle (1)}(t_1) d\mu _{n,\mathrm{fr}}^{\scriptscriptstyle (2)}(t_2) d\mu _{n,\mathrm{fr}}^{\scriptscriptstyle (2)}(u_2) \nonumber \\&\qquad +\iiint {\mathrm e}^{-(\lambda _n({\tilde{a}}_2)+\lambda _n({\tilde{b}}_2))t_2}{\mathrm e}^{-\lambda _n({\tilde{a}}_1)t_1}{\mathrm e}^{-\lambda _n({\tilde{b}}_1)u_1} {\bar{M}}_{t-T_\mathrm{unfr}-t_2,u-T_\mathrm{unfr}-t_2}^{\rho _{t-T_\mathrm{unfr}-t_1,{\tilde{a}}_1},\rho _{u-T_\mathrm{unfr}-u_1,{\tilde{b}}_1}}({\tilde{a}}_2,{\tilde{b}}_2) \nonumber \\&\qquad \qquad \qquad \qquad \qquad \times d\mu _{n,\mathrm{fr}}^{\scriptscriptstyle (1)}(t_2) d\mu _{n,\mathrm{fr}}^{\scriptscriptstyle (2)}(t_1) d\mu _{n,\mathrm{fr}}^{\scriptscriptstyle (2)}(u_1) \nonumber \\&\qquad + \iint {\mathrm e}^{-\lambda _n({\tilde{a}}_1)t_1} {\mathrm e}^{-\lambda _n({\tilde{a}}_2)t_2} {\bar{m}}_{t-T_\mathrm{unfr}-\vec {t}}^{\chi _n^{\scriptscriptstyle (K)}}({\tilde{\vec {a}}}) d\mu _{n,\mathrm{fr}}^{\scriptscriptstyle (1)}(t_1) d\mu _{n,\mathrm{fr}}^{\scriptscriptstyle (2)}(t_2) \nonumber \\&\qquad \qquad \qquad \qquad \qquad \times \iint {\mathrm e}^{-\lambda _n({\tilde{b}}_1)u_1} {\mathrm e}^{-\lambda _n({\tilde{b}}_2)u_2} {\bar{m}}_{u-T_\mathrm{unfr}-\vec {u}}^{\chi _n^{\scriptscriptstyle (K)}}({\tilde{\vec {b}}}) d\mu _{n,\mathrm{fr}}^{\scriptscriptstyle (1)}(u_1) d\mu _{n,\mathrm{fr}}^{\scriptscriptstyle (2)}(u_2) , \end{aligned}$$

where $\rho _{t_2,{\tilde{a}}_2}(t'_1)={\bar{m}}_{t_2}^{\chi _n^{\scriptscriptstyle (K)}(t'_1,\cdot )}({\tilde{a}}_2)$ is the characteristic from (7.5). Abbreviate

9.26 $$\begin{aligned} D={\mathrm e}^{-2t^*-\frac{1}{4}\left\| \vec {\xi }\right\| ^2}{\hat{Z}}'_{n,t^*}(\vec {\xi })-{\mathrm e}^{-2u^*}\left| Z^{\prime \scriptscriptstyle (K)}_{n,u^*}\right| ={\mathrm e}^{-2t^*-\frac{1}{4}\left\| \vec {\xi }\right\| ^2}{\hat{Z}}'_{n,t^*}(\vec {\xi })-{\mathrm e}^{-2u^*}{\hat{Z}}'_{n,u^*}(\vec {0}). \end{aligned}$$

Then

9.27 $$\begin{aligned} {\mathbb {E}}\left( \left. D^2\,\right| {\mathcal {B}}_\mathrm{fr}\right)&= {\mathrm e}^{-4t^*-\frac{2}{4}\left\| \vec {\xi }\right\| ^2}{\mathbb {E}}\left( \left. {\hat{Z}}_{n,t^*}'(\vec {\xi }) {\hat{Z}}_{n,t^*}'(\vec {\xi })\,\right| {\mathcal {B}}_\mathrm{fr}\right) -2{\mathrm e}^{-2t^*-\frac{1}{4}\left\| \vec {\xi }\right\| ^2-2u^*}{\mathbb {E}}\left( \left. {\hat{Z}}_{n,t^*}'(\vec {\xi }) {\hat{Z}}_{n,u^*}'(\vec {0})\,\right| {\mathcal {B}}_\mathrm{fr}\right) \nonumber \\&\quad +{\mathrm e}^{-4u^*}{\mathbb {E}}\left( \left. {\hat{Z}}_{n,u^*}'(\vec {0}) {\hat{Z}}_{n,u^*}'(\vec {0})\,\right| {\mathcal {B}}_\mathrm{fr}\right) . \end{aligned}$$

Applying (9.25) to each of the 3 terms in (9.27) gives 12 summands. From the first term in the right-hand side of (9.25), we obtain

9.28 $$\begin{aligned}&\frac{{\mathrm e}^{o(1)}}{s_n^2}\iint s_n^{-4}\left[ {\mathrm e}^{-2\lambda _n({\tilde{a}}_1)t_1} {\mathrm e}^{-2\lambda _n({\tilde{a}}_2)t_2} {\bar{M}}_{t-T_\mathrm{unfr}-\vec {t},t-T_\mathrm{unfr}-\vec {t}}^{\chi _n^{\scriptscriptstyle (K)},\chi _n^{\scriptscriptstyle (K)}}\left( {\tilde{\vec {a}}},{\tilde{\vec {a}}}\right) \right. \nonumber \\&\quad - 2{\mathrm e}^{-(\lambda _n({\tilde{a}}_1)+\lambda _n(1))t_1} {\mathrm e}^{-(\lambda _n({\tilde{a}}_2)+\lambda _n(1))t_2} {\bar{M}}_{t-T_\mathrm{unfr}-\vec {t},u-T_\mathrm{unfr}-\vec {t}}^{\chi _n^{\scriptscriptstyle (K)},\chi _n^{\scriptscriptstyle (K)}}\left( {\tilde{\vec {a}}},\vec {1}\right) \nonumber \\&\quad \left. + \, {\mathrm e}^{-2\lambda _n(1)t_1} {\mathrm e}^{-2\lambda _n(1)t_2} {\bar{M}}_{u-T_\mathrm{unfr}-\vec {t},u-T_\mathrm{unfr}-\vec {t}}^{\chi _n^{\scriptscriptstyle (K)},\chi _n^{\scriptscriptstyle (K)}}\left( \vec {1},\vec {1}\right) \right] d\mu _{n,\mathrm{fr}}^{\scriptscriptstyle (1)}(t_1) d\mu _{n,\mathrm{fr}}^{\scriptscriptstyle (2)}(t_2) . \end{aligned}$$

As in (9.22), Theorem 3.15 and Corollary 6.5 imply that the integrand in (9.28) is at most $(4\varepsilon +o(1)){\mathrm e}^{-2\lambda _n(1)t_1+o(1)} {\mathrm e}^{-2\lambda _n(1)t_2+o(1)}$ in absolute value, uniformly for $\lambda _n(1)[t_1 \vee t_2]\le K'$, and is otherwise bounded by $4K''{\mathrm e}^{-2\lambda _n(1\wedge {\tilde{a}}_1)t_1}{\mathrm e}^{-2\lambda _n(1\wedge {\tilde{a}}_2)t_2}$. Using Lemmas 9.1 and 9.2, it easily follows that the quantity in (9.28) is at most $5\varepsilon$ in absolute value, whp. From the second term in the right-hand side of (9.25), we obtain similarly

9.29 $$\begin{aligned}&\frac{{\mathrm e}^{o(1)}}{s_n^3}\iiint s_n^{-3}\left[ {\mathrm e}^{-2\lambda _n({\tilde{a}}_1)t_1} {\mathrm e}^{-2\lambda _n({\tilde{a}}_2)(t_2+u_2)} {\bar{M}}_{t-T_\mathrm{unfr}-t_1,t-T_\mathrm{unfr}-t_1}^{\rho _{t-T_\mathrm{unfr}-t_2,{\tilde{a}}_2},\rho _{t-T_\mathrm{unfr}-u_2,{\tilde{a}}_2}}\left( {\tilde{a}}_1,{\tilde{a}}_1\right) \right. \nonumber \\&\quad - 2{\mathrm e}^{-(\lambda _n({\tilde{a}}_1)+\lambda _n(1))t_1}{\mathrm e}^{-\lambda _n({\tilde{a}}_2)t_2}{\mathrm e}^{-\lambda _n(1)u_2} {\bar{M}}_{t-T_\mathrm{unfr}-t_1,u-T_\mathrm{unfr}-t_1}^{\rho _{t-T_\mathrm{unfr}-t_2,{\tilde{a}}_2},\rho _{u-T_\mathrm{unfr}-u_2,1}}({\tilde{a}}_1,1) \nonumber \\&\quad \left. + \, {\mathrm e}^{-2\lambda _n(1)t_1} {\mathrm e}^{-2\lambda _n(1)(t_2+u_2)} {\bar{M}}_{u-T_\mathrm{unfr}-t_1,u-T_\mathrm{unfr}-t_1}^{\rho _{u-T_\mathrm{unfr}-t_2,1},\rho _{u-T_\mathrm{unfr}-u_2,1}}\left( 1,1\right) \right] d\mu _{n,\mathrm{fr}}^{\scriptscriptstyle (1)}(t_1) d\mu _{n,\mathrm{fr}}^{\scriptscriptstyle (2)}(t_2) d\mu _{n,\mathrm{fr}}^{\scriptscriptstyle (2)}(u_2) . \end{aligned}$$

Arguing from Proposition 7.1 instead of Theorem 3.15, the quantity in (9.29) is again at most $5\varepsilon$ in absolute value, whp. The third and fourth terms from (9.25) are analogous. This verifies the second moment condition (3.56).

**Verification of** (3.58): Finally we verify condition (b) of Theorem 3.29. For $R\in (0,\infty )$, let $v_{j,R}$ be the first vertex in ${\mathcal {B}}^{\scriptscriptstyle (j)}$ born after time $T_\mathrm{unfr}$ with $\left| \mathsf{BP}_{f_n(1)}^{\scriptscriptstyle (v)}\right| \ge Rs_n^2$, so that *v* is $Rs_n^2$-lucky in the sense of [Part I, Definition 2.14]. (We will use a similar notion in Definition 9.3.) It follows from [Part I, Proposition 2.17 and Lemma 2.18] that

9.30 $$\begin{aligned} T_{v_{j,R}}^{\mathcal {B}}=T_\mathrm{unfr}+O_{\scriptscriptstyle {{\mathbb {P}}}}(f_n(1)). \end{aligned}$$

To see this, note that [Part I, Lemma 2.18] gives $T_{v_{j,R}}-T_{v_{j,r}}=O_{\scriptscriptstyle {{\mathbb {P}}}}(f_n(1))$ for each $r>0$, so it suffices to show that $T_{v_{j,r}}^{\mathcal {B}}=T_\mathrm{unfr}+O_{\scriptscriptstyle {{\mathbb {P}}}}(f_n(1))$ for some $r>0$ sufficiently small. Let $\varepsilon >0$ and use Lemmas 9.1 and 9.2 to find $K<\infty$ such that $\mu _{n,\mathrm{fr}}^{\scriptscriptstyle (j)}([0,Kf_n(1)])\ge \frac{1}{2}s_n$ with probability at least $1-\varepsilon$ for *n* sufficiently large. Assuming this event occurs, the number of vertices born in the time interval $\left( T_\mathrm{unfr},T_\mathrm{unfr}+Kf_n(1)\right]$ is conditionally Poisson with mean at least $\frac{1}{2}s_n$. By [Part I, Proposition 2.17] we can choose $r>0$ such that, for *n* sufficiently large, each such vertex has probability at least $1/\varepsilon s_n$ of being $r s_n^2$-lucky. Hence at least one vertex born by time $T_\mathrm{unfr}+Kf_n(1)$ will be $rs_n^2$-lucky, with probability at least $1-\varepsilon -\mathrm {e}^{-1/2\varepsilon }-o(1)$. This verifies $T_{v_{j,r}}^{\mathcal {B}}=T_\mathrm{unfr}+O_{\scriptscriptstyle {{\mathbb {P}}}}(f_n(1))$ and hence proves (9.30).

Now let $\varepsilon >0$ and choose $n_0 \in {\mathbb {N}}$ and $C' <\infty$ such that $\lambda _n(1)(f_n(1)+f_n(1+1/s_n)) \le C'$ for all $n\ge n_0$. Choose $R \in (0,\infty )$ such that $\frac{16}{(\log 2)^4}{\mathrm e}^{4C'} (1+2R)/R^2 \le \varepsilon$. After possibly increasing $n_0$, (9.30) and (6.15) yield a constant $C'' \in (0,\infty )$ such that with probability at least $1-\varepsilon$, $\lambda _n(1)(T_{v_{j,R}}^{\mathcal {B}}- T_\mathrm{unfr})\le C''$. Denote this event by ${\mathcal {A}}$ and assume for the remainder of the proof that ${\mathcal {A}}$ holds.

Set ${\mathcal {L}}_R^{\scriptscriptstyle (j)}$ to be the collection of descendants *w* of $v_{j,R}$ such that $T_w^{\mathcal {B}}-T_{v_{j,R}}^{\mathcal {B}}\le f_n(1)$ (thus $\left| {\mathcal {L}}_R^{\scriptscriptstyle (j)}\right| \ge Rs_n^2$ by definition). We will repeat the previous arguments used to analyze $Z_{n,t^*}$, with ${\mathcal {L}}_R^{\scriptscriptstyle (j)}$ playing the role of ${\mathcal {B}}_\mathrm{fr}^{\scriptscriptstyle (j)}$. Instead of $\partial {\mathcal {L}}_R^{\scriptscriptstyle (j)}$, we consider the subset

9.31 $$\begin{aligned} {\mathcal {U}}_R^{\scriptscriptstyle (j)}=\left\{ v\in \partial {\mathcal {L}}_R^{\scriptscriptstyle (j)}:T_{p\left( v\right) }^{\mathcal {B}}+f_n(1)<T_v^{\mathcal {B}}<T_{p\left( v\right) }^{\mathcal {B}}+f_n(1+1/s_n)\right\} \end{aligned}$$

[the set of immediate children of ${\mathcal {L}}_R^{\scriptscriptstyle (j)}$ born to a parent of age between times $f_n(1)$ and $f_n(1+1/s_n)$]. Since $v_{j,R}$ is born after $T_\mathrm{unfr}$, it follows immediately that ${\mathcal {B}}_\mathrm{fr}^{\scriptscriptstyle (j)} \cap {\mathcal {U}}_R^{\scriptscriptstyle (j)}=\varnothing$. Moreover the arrival time $T_v^{\mathcal {B}}$ of any $v\in {\mathcal {U}}_R^{\scriptscriptstyle (j)}$ satisfies

9.32 $$\begin{aligned} T_{v_{j,R}}^{{\mathcal {B}}}+ f_n(1)<T_{p\left( v\right) }^{\mathcal {B}}+f_n(1)<T_v^{\mathcal {B}}<T_{p\left( v\right) }^{\mathcal {B}}+f_n(1+1/s_n)\le T_{v_{j,R}}^{{\mathcal {B}}}+ f_n(1) + f_n(1+1/s_n). \end{aligned}$$

Let ${\tilde{{\mathcal {P}}}}^{\scriptscriptstyle (j)}_{n,R}=\sum _{v\in {\mathcal {U}}_R^{\scriptscriptstyle (j)}} \delta _{(T_v^{\mathcal {B}}-T_\mathrm{unfr}, \mathsf{BP}^{\scriptscriptstyle (v)})}$ [cf. (9.8)]. The definitions of $v_{j,R}$ and ${\mathcal {L}}_R^{\scriptscriptstyle (j)}$ depend only on descendants born to parents of age at most $f_n(1)$, whereas ${\mathcal {U}}_R^{\scriptscriptstyle (j)}$ consists of descendants born to parents of age greater than $f_n(1)$. It follows that ${\tilde{{\mathcal {P}}}}^{\scriptscriptstyle (j)}_{n,R}$ is a Cox process conditionally on ${\mathcal {L}}_R^{\scriptscriptstyle (j)}$ with intensity measure $d{\tilde{\mu }}_{n,R}^{\scriptscriptstyle (j)}\otimes d{\mathbb {P}}(\mathsf{BP}^{\scriptscriptstyle (1)}\in \cdot )$, where ${\tilde{\mu }}_{n,R}$ is the measure such that, for all measurable, nonnegative functions *h* on ${\mathbb {R}}$,

9.33 $$\begin{aligned} \int h(y) \, d{\tilde{\mu }}_{n,R}^{\scriptscriptstyle (j)}(y)= \sum _{w \in {\mathcal {L}}_R^{\scriptscriptstyle (j)}} \int _{f_n(1)}^{f_n(1+1/s_n)} h(y-(T_\mathrm{fr}^{\scriptscriptstyle (j)}-T_w)) \, d\mu _n(y). \end{aligned}$$

[These are the analogues of ${\mathcal {P}}_{n,\mathrm{unfr}}^{\scriptscriptstyle (j)}$ and $\mu _{n,\mathrm{fr}}^{\scriptscriptstyle (j)}$, see (9.2 and (9.8).] In particular, the total mass of ${\tilde{\mu }}_{n,R}^{\scriptscriptstyle (j)}$ is at least $Rs_n$. Using (9.32) we find that its support is in $[T_{v_{j,R}}^{\mathcal {B}}-T_\mathrm{unfr}+f_n(1), T_{v_{j,R}}^{\mathcal {B}}-T_\mathrm{unfr}+f_n(1)+f_n(1+1/s_n)]$ and, uniformly over $t_j$ in that set and $t^*\ge 0$,

9.34 $$\begin{aligned} \lambda _n(1)(T_{v_{j,R}}^{\mathcal {B}}- T_\mathrm{unfr})&\le \lambda _n(1) t_j \le \lambda _n(1)(T_{v_{j,R}}^{\mathcal {B}}- T_\mathrm{unfr}) +C', \end{aligned}$$

9.35 $$\begin{aligned} \lambda _n(1) (t-T_\mathrm{unfr}-t_j)&\ge \frac{1}{2}\log (n/s_n^3)-C''-C'. \end{aligned}$$

In particular, the fact that the right-hand side of (9.35) tends to infinity implies that, possibly after increasing $n_0$, ${\mathcal {U}}_R^{\scriptscriptstyle (j)} \subset {\mathcal {B}}_t^{\scriptscriptstyle (j)}$ whenever $n\ge n_0$ and $t^*\ge 0$. Furthermore, Theorem 3.15 and Proposition 7.1 yield a constant $K<\infty$ such that

9.36 $$\begin{aligned}&{\bar{m}}^{\chi _n^{(K)}}_{t-T_\mathrm{unfr}-t_1,t-T_\mathrm{unfr}-t_2}(\vec {1}) \ge s_n/2, \end{aligned}$$

9.37 $$\begin{aligned}&{\bar{M}}_{t-T_\mathrm{unfr}-\vec {t},u-T_\mathrm{unfr}-\vec {t}}^{\chi _n^{\scriptscriptstyle (K)},\chi _n^{\scriptscriptstyle (K)}}\left( \vec {1},\vec {1}\right) \le \frac{2}{(\log 2)^2} s_n^4, \end{aligned}$$

9.38 $$\begin{aligned}&{\bar{M}}_{t-T_\mathrm{unfr}-t_{j'},u-T_\mathrm{unfr}-t_i}^{\rho _{t-T_\mathrm{unfr}-t_j,1},\rho _{u-T_\mathrm{unfr}-u_j,1}}(1,1)\le \frac{2}{\log 2}s_n^3, \end{aligned}$$

for $\left\{ j,j'\right\} =\left\{ 1,2\right\}$, all $t_1,t_2$ in the support of ${\tilde{\mu }}_{n,R}^{\scriptscriptstyle (j)}$ and $n\ge n_0$. Using this *K* to truncate, let ${\tilde{Z}}^{\prime \scriptscriptstyle (K)}_{n,t^*,R}$ denote the restriction of $Z^{\prime \scriptscriptstyle (K)}_{n,t^*}$ to pairs $(v_1,v_2)$ for which each $v_j$ is a descendant of some vertex of ${\mathcal {U}}_R^{\scriptscriptstyle (j)}$. As in the argument leading to (9.20), we conclude that

9.39 $$\begin{aligned} \left| Z_{n,t^*}^*\right| \ge \big \vert {\tilde{Z}}^{\prime \scriptscriptstyle (K)}_{n,t^*,R}\big \vert&=\frac{{\mathrm e}^{2t^*}}{s_n^3} \int d{\tilde{{\mathcal {P}}}}^{\scriptscriptstyle (1)}_{n,R}(t_1,bp^{\scriptscriptstyle (1)})\nonumber \\&\quad \, \times \int d{\tilde{{\mathcal {P}}}}^{\scriptscriptstyle (2)}_{n,R}(t_2,bp^{\scriptscriptstyle (2)}) {\mathrm e}^{-\lambda _n(1)(t_1+t_2)} {\bar{z}}^{\chi _n^{(K)},bp^{(1)},bp^{(2)}}_{t-T_\mathrm{unfr}-t_1,t-T_\mathrm{unfr}-t_2}(\vec {1}). \end{aligned}$$

Hence, on ${\mathcal {A}}$, we may use (9.34) and (9.36) to obtain

9.40 $$\begin{aligned}&{\mathbb {E}}\left( \left. \big \vert {\tilde{Z}}^{\prime \scriptscriptstyle (K)}_{n,t^*,R}\big \vert \,\right| {\mathcal {L}}^{\scriptscriptstyle (1)}_R, {\mathcal {L}}^{\scriptscriptstyle (2)}_R\right) \end{aligned}$$

9.41 $$\begin{aligned}&\quad \ge \frac{{\mathrm e}^{2t^*}}{s_n^3} \int d{\tilde{\mu }}_{n,R}^{\scriptscriptstyle (1)}(t_1) \int d{\tilde{\mu }}_{n,R}^{\scriptscriptstyle (2)}(t_2) \exp \left( -\lambda _n(1)\left( T_{v_{1,R}}^{\mathcal {B}}-T_\mathrm{unfr}+T_{v_{2,R}}^{\mathcal {B}}-T_\mathrm{unfr}\right) -2C'\right) \frac{s_n}{2} \nonumber \\&\quad = \frac{1}{2}\exp \left( 2t^*-\lambda _n(1)\left( T_{v_{1,R}}^{\mathcal {B}}-T_\mathrm{unfr}+T_{v_{2,R}}^{\mathcal {B}}-T_\mathrm{unfr}\right) -2C'\right) \frac{\left| {\tilde{\mu }}_{n,R}^{\scriptscriptstyle (1)}\right| }{s_n} \frac{\left| {\tilde{\mu }}_{n,R}^{\scriptscriptstyle (2)}\right| }{s_n} \end{aligned}$$

9.42 $$\begin{aligned}&\quad \ge \frac{R^2}{2} \exp \left( 2t^*- 2 (C''+C')\right) . \end{aligned}$$

Similarly, using (9.24) to compute the variance of $\big \vert {\tilde{Z}}^{\prime \scriptscriptstyle (K)}_{n,t^*,R}\big \vert$ as in (9.25), and employing (9.34) and (9.37)–(9.38) for the estimation, we find that, on ${\mathcal {A}}$,

9.43 $$\begin{aligned} {{\,\mathrm{Var}\,}}\left( \left. \big \vert {\tilde{Z}}^{\prime \scriptscriptstyle (K)}_{n,t^*,R}\big \vert \,\right| {\mathcal {L}}^{\scriptscriptstyle (1)}_R, {\mathcal {L}}^{\scriptscriptstyle (2)}_R\right)&\le \frac{2}{(\log 2)^2}\exp \left( 4t^*-2\lambda _n(1)\left( T_{v_{1,R}}^{\mathcal {B}}- T_\mathrm{unfr}+T_{v_{2,R}}^{\mathcal {B}}- T_\mathrm{unfr}\right) \right) \nonumber \\&\quad \times \left( \frac{\left| {\tilde{\mu }}_{n,R}^{\scriptscriptstyle (1)}\right| \left| {\tilde{\mu }}_{n,R}^{\scriptscriptstyle (2)}\right| s_n^4 }{s_n^6} +\, \frac{\left| {\tilde{\mu }}_{n,R}^{\scriptscriptstyle (1)}\right| \left| {\tilde{\mu }}_{n,R}^{\scriptscriptstyle (2)}\right| ^2 s_n^3 + \left| {\tilde{\mu }}_{n,R}^{\scriptscriptstyle (1)}\right| ^2 \left| {\tilde{\mu }}_{n,R}^{\scriptscriptstyle (2)}\right| s_n^3 }{s_n^6} \right) . \end{aligned}$$

Abbreviate the conditional mean from (9.40) as *m*. Chebyshev’s inequality, (9.41) and (9.43) give

9.44 $$\begin{aligned} {\mathbb {P}}\left( \left. \big \vert {\tilde{Z}}^{\prime \scriptscriptstyle (K)}_{n,t^*,R}\big \vert \ge \tfrac{1}{2} m\,\right| {\mathcal {L}}^{\scriptscriptstyle (1)}_R, {\mathcal {L}}^{\scriptscriptstyle (2)}_R\right)&\ge 1-\frac{16}{(\log 2)^4}{\mathrm e}^{4C'} \left( \frac{s_n^2}{\left| {\tilde{\mu }}_{n,R}^{\scriptscriptstyle (1)}\right| \left| {\tilde{\mu }}_{n,R}^{\scriptscriptstyle (2)}\right| } + \frac{s_n}{\left| {\tilde{\mu }}_{n,R}^{\scriptscriptstyle (1)}\right| } + \frac{s_n}{\left| {\tilde{\mu }}_{n,R}^{\scriptscriptstyle (2)}\right| } \right) \nonumber \\&\ge 1-\frac{16}{(\log 2)^4}{\mathrm e}^{4C'}\left( \frac{1}{R^2} + 2\frac{1}{R} \right) \ge 1-\varepsilon , \end{aligned}$$

on ${\mathcal {A}}$. For given $C<\infty$, we use (9.42) to choose $t^*$ sufficiently large that $\frac{1}{2}m \ge C$. The claim now follows from $\left| Z_{n,t^*}^*\right| \ge \big \vert {\tilde{Z}}_{n,t^*,R}'\big \vert$ and (9.44). $\square$

### No Collisions from the Frozen Cluster: Proof of Lemma 3.32 (b)

In this section we prove Lemma 3.32 (b), which will show that whp the collision edge neither starts nor ends in the frozen cluster.

#### Definition 9.3

Let $\varepsilon _1$ denote the constant from Lemma 4.5 for $K=1$. Call a vertex $v \in {\mathcal {T}}^{\scriptscriptstyle (j)}$*lucky* if $\big \vert \mathsf{BP}^{\scriptscriptstyle (v)}_{f_n(1)}\big \vert \ge s_n^2/\varepsilon _1$, and set

9.45 $$\begin{aligned} T^{\scriptscriptstyle (j)}_\mathrm{lucky}=\inf \left\{ T_v:v\in {\mathcal {T}}^{\scriptscriptstyle (j)}\setminus \left\{ \varnothing _j\right\} \text { is lucky and }T_v> T_{p\left( v\right) }+f_n(1)\right\} , \end{aligned}$$

the first time that a lucky vertex is born to a parent of age greater than $f_n(1)$.

The notion that *v* is lucky is the same as the notion of *v* being $s_n^2/\varepsilon _1$-lucky in [Part I, Definition 2.14 and (2.24)]. We will use this fact later on, when we apply results from Part I.

In view of Definition 3.17 and Lemma 4.5, we have

9.46 $$\begin{aligned} v\in {\mathcal {T}}^{\scriptscriptstyle (j)}\text { is lucky} \; \implies \; T_\mathrm{fr}^{\scriptscriptstyle (j)}\le T_v+f_n(1). \end{aligned}$$

In other words, a lucky vertex has enough descendants in time $f_n(1)$ that the integral in the definition (3.31) of the freezing time must be at least $s_n$.

It has been proved in [Part I, Lemma 6.4 and Proposition 2.17] that the distribution of

9.47 $$\begin{aligned} \sum _{v\in \mathsf{BP}^{\scriptscriptstyle (j)}_{T^{\scriptscriptstyle (j)}_\mathrm{lucky}}} \left( f_n^{-1}\left( T^{\scriptscriptstyle (j)}_\mathrm{lucky}-T_v \right) -1 \right) ^+ \quad \text {is exponential with rate} \;{\mathbb {P}}(v~\text {is lucky}), \end{aligned}$$

and that there is a constant $\delta >0$ so that for sufficiently large *n*,

9.48 $$\begin{aligned} {\mathbb {P}}(v\text { is lucky}) \ge \delta /s_n. \end{aligned}$$

Now we are in the position to prove Lemma 3.32 (b):

#### Proof of Lemma 3.32 (b)

It suffices to show that the Cox intensity $Z_{n, {\overline{t}}}$ satisfies

9.49 $$\begin{aligned} \sum _{v_1 \in {\mathcal {B}}_{{\overline{t}}}^{\scriptscriptstyle (1)}} \sum _{v_2 \in {\mathcal {B}}_{{\overline{t}}}^{\scriptscriptstyle (2)}} {\mathbb {1}}_{\left\{ \left\{ v_1,v_2\right\} \cap {\mathcal {B}}_{\mathrm{fr}} \ne \varnothing \right\} } Z_{n, {\overline{t}}}(\left\{ v_1\right\} \times \left\{ v_2\right\} ) = o_{{\mathbb {P}}}(1), \end{aligned}$$

where we recall that $Z_{n,t}(\left\{ v_1\right\} \times \left\{ v_2\right\} )= \tfrac{1}{n} \mu _n\bigl (\Delta R_{v_1,v_2}, R_1(t)-R_1(T_{v_1}^{\mathcal {B}})+R_2(t)-R_2(T_{v_2}^{\mathcal {B}})\bigr )$.

We begin with the contribution to $Z_{n,{\overline{t}}}$ arising from the restriction of $\mu _n$ to $(f_n(1),\infty )$. Note that, by construction, $R_j({\overline{t}})-R_j(T_\mathrm{fr}^{\scriptscriptstyle (j)})=\lambda _n(1)^{-1}(\tfrac{1}{2}\log (n/s_n^3)+K)$ and $\left( R_j(T_\mathrm{fr}^{\scriptscriptstyle (j)})-R_j(T_{v_j}^{\mathcal {B}}) \right) ^+ = \left( T_\mathrm{fr}^{\scriptscriptstyle (j)}-T_{v_j}^{\mathcal {B}}\right) ^+$. Hence

9.50 $$\begin{aligned}&\tfrac{1}{n}\mu _n\big \vert _{(f_n(1),\infty )}\left( \Delta R_{v_1,v_2}, R_1({\overline{t}})-R_1(T_{v_1}^{\mathcal {B}})+R_2({\overline{t}})-R_2\left( T_{v_2}^{\mathcal {B}}\right) \right) \nonumber \\&\quad \le \tfrac{1}{n} \mu _n\left( f_n(1), \frac{\log (n/s_n^3)+2K}{\lambda _n(1)}+R_1(T_\mathrm{fr}^{\scriptscriptstyle (1)})-R_1(T_{v_1}^{\mathcal {B}})+R_2(T_\mathrm{fr}^{\scriptscriptstyle (2)})-R_2(T_{v_2}^{\mathcal {B}}) \right) \nonumber \\&\quad \le \tfrac{1}{n}\mu _n\left( f_n(1),3\frac{\log (n/s_n^3)+2K}{\lambda _n(1)} \right) + \sum _{j=1}^2 \tfrac{1}{n}\mu _n\left( f_n(1), f_n(1)\vee 3\left( T_\mathrm{fr}^{\scriptscriptstyle (j)}-T_{v_j}^{\mathcal {B}}\right) ^+ \right) . \end{aligned}$$

Lemma 4.3 and (6.15) imply that the first term in (9.50) is $O(1) (\log (n/s_n^3)+K)/(ns_n)$. In the second term, the *j*th summand is zero if $v_j \not \in {\mathcal {B}}_\mathrm{fr}^{\scriptscriptstyle (j)}$. For $v_j \in {\mathcal {B}}_\mathrm{fr}^{\scriptscriptstyle (j)}$, we consider separately the intervals

9.51 $$\begin{aligned} \begin{aligned} I_1&=\left( f_n(1), f_n(1) \vee \left( T_{\mathrm {lucky}}^{\scriptscriptstyle (j)}-T_{v_j}^{\mathcal {B}}\right) ^+\right) ,\\ I_2&=\left( f_n(1)\vee \left( T_{\mathrm {lucky}}^{\scriptscriptstyle (j)}-T_{v_j}^{\mathcal {B}}\right) ^+, f_n(1)\vee \left( T_\mathrm{fr}^{\scriptscriptstyle (j)}-T_{v_j}^{\mathcal {B}}\right) ^+ \right) , \\ I_3&= \left( f_n(1)\vee \left( T_\mathrm{fr}^{\scriptscriptstyle (j)}-T_{v_j}^{\mathcal {B}}\right) ^+, f_n(1)\vee 3\left( T_\mathrm{fr}^{\scriptscriptstyle (j)}-T_{v_j}^{\mathcal {B}}\right) ^+ \right) , \end{aligned} \end{aligned}$$

where $T_{\mathrm {lucky}}^{\scriptscriptstyle (j)}$ was defined in Definition 9.3. The definition of $\mu _n$ gives $\mu _n(I_1)=\bigl ( f_n^{-1}(T_{\mathrm {lucky}}^{\scriptscriptstyle (j)}-T_{v_j}^{\mathcal {B}})-1 \bigr )^+$. For $I_2$, note from (9.46) that $I_2$ is a subinterval of $(f_n(1),\infty )$ of length at most $f_n(1)$, so $\mu _n(I_2)\le O(1/s_n)$ by Lemma 4.3. For $I_3$, note from Lemma 4.1 that $f_n(3^{1/\varepsilon _0s_n} m)\ge 3f_n(m)$ for any $m\ge 1$. It follows that $f_n^{-1}(3y)\le 3^{1/\varepsilon _0s_n} f_n^{-1}(y)=(1+O(1/s_n))f_n^{-1}(y)$ uniformly over $y\ge f_n(1)$, so that $\mu _n(I_3)\le O(1/s_n)f_n^{-1}(T_\mathrm{fr}^{\scriptscriptstyle (j)})$. We conclude that

9.52 $$\begin{aligned}&\tfrac{1}{n}\mu _n\big \vert _{(f_n(1),\infty )}\left( \Delta R_{v_1,v_2}, R_1({\overline{t}})-R_1(T_{v_1}^{\mathcal {B}})+R_2({\overline{t}})-R_2(T_{v_2}^{\mathcal {B}}) \right) \nonumber \\&\quad \le O(1) \left( \frac{\log (n/s_n^3)}{n s_n} + \sum _{j=1}^2 {\mathbb {1}}_{\left\{ v_j \in {\mathcal {B}}_\mathrm{fr}^{\scriptscriptstyle (j)}\right\} }\left( \tfrac{1}{n}\left( f_n^{-1}\left( T_{\mathrm {lucky}}^{\scriptscriptstyle (j)}-T_{v_j}^{\mathcal {B}}\right) -1 \right) ^+ + \frac{1+f_n^{-1}(T_\mathrm{fr}^{\scriptscriptstyle (j)})}{n s_n} \right) \right) . \end{aligned}$$

By Theorem 3.18 (a), $f_n^{-1}(T_\mathrm{fr}^{\scriptscriptstyle (j)})=O_{\mathbb {P}}(1)$. Sum (9.52) over $v_1 \in {\mathcal {B}}_{{\overline{t}}}^{\scriptscriptstyle (1)}, v_2 \in {\mathcal {B}}_{{\overline{t}}}^{\scriptscriptstyle (2)}$ with $\left\{ v_1,v_2\right\} \cap {\mathcal {B}}_{\mathrm{fr}} \ne \varnothing$ and use $T_{v_j}^{\mathcal {B}}=T_{v_j}$ for $v_j \in {\mathcal {B}}_\mathrm{fr}^{\scriptscriptstyle (j)}$ to obtain

9.53 $$\begin{aligned} O(1) \sum _{\left\{ j,j'\right\} =\left\{ 1,2\right\} } \left| {\mathcal {B}}^{\scriptscriptstyle (\smash {j'})}_{{\overline{t}}}\right| \Big (\frac{\log (n/s_n^3)+O_{\mathbb {P}}(1)}{n s_n}\left| {\mathcal {B}}^{\scriptscriptstyle (j)}_\mathrm{fr}\right| + \frac{1}{n} \sum _{v_j\in {\mathcal {B}}^{\scriptscriptstyle (j)}_\mathrm{fr}} \left( f_n^{-1}\left( T_{\mathrm {lucky}}^{\scriptscriptstyle (j)}-T_{v_j} \right) -1 \right) ^+ \Big ) . \end{aligned}$$

By Lemma 3.32 (a), $\left| {\mathcal {B}}^{\scriptscriptstyle (\smash {j'})}_{{\overline{t}}}\right| =O_{\mathbb {P}}(\sqrt{n s_n})$, and by Theorem 3.18 (b), $\left| {\mathcal {B}}^{\scriptscriptstyle (j)}_\mathrm{fr}\right| =O_{\mathbb {P}}(s_n^2)$. In the sum over $v_j\in {\mathcal {B}}^{\scriptscriptstyle (j)}_\mathrm{fr}$, only terms with $v_j\in {\mathcal {B}}^{\scriptscriptstyle (j)}_{T_{\mathrm {lucky}}^{\scriptscriptstyle (j)}}$ can contribute, so (9.46)–(9.48) imply that the inner sum in (9.53) is $O_{\mathbb {P}}(s_n)$. Hence (9.53) is $O_{\mathbb {P}}(\log (n/s_n^3)/\sqrt{n/s_n^3})$, which is $o_{\mathbb {P}}(1)$ since $n/s_n^3\rightarrow \infty$.

We now turn to the contribution to $Z_{n,{\overline{t}}}$ arising from the restriction of $\mu _n$ to $[0,f_n(1)]$ and split the sum into three groups of vertex pairs. Let $(J,J')$ denote the random ordering of $\left\{ 1,2\right\}$ for which $T_\mathrm{fr}^{\scriptscriptstyle (J)} < T_\mathrm{fr}^{\scriptscriptstyle (J')}$. The first group of vertex pairs are those with $v_J \in {\mathcal {B}}_{{\overline{t}}}^{\scriptscriptstyle (J)}$ and $v_{J'} \in {\mathcal {B}}_{T_\mathrm{fr}^{\scriptscriptstyle (J)}}^{\scriptscriptstyle (J')}$. That is, the vertex in the slower-growing cluster is born before the faster cluster freezes. We show that the number $\big \vert {\mathcal {B}}_{T_\mathrm{fr}^{\scriptscriptstyle (J)}}^{\scriptscriptstyle (J')}\big \vert$ of such choices for $v_{J'}$ is $O_{\mathbb {P}}(1)$. By Theorem 3.18 (a), $f_n^{-1}(T_\mathrm{fr}^{\scriptscriptstyle (j)}) {\mathop {\longrightarrow }\limits ^{\scriptscriptstyle {{\mathbb {P}}}}}M^{\scriptscriptstyle (j)}$ for $j\in \left\{ 1,2\right\}$ and $M^{\scriptscriptstyle (1)} \ne M^{\scriptscriptstyle (2)}$ a.s. Hence $T_\mathrm{fr}^{\scriptscriptstyle (J)} < f_n(M^{\scriptscriptstyle (J')})$ whp. A vertex $v'\in {\mathcal {B}}^{\scriptscriptstyle (J')}_\mathrm{fr}$ with $T_{v'}^{\mathcal {B}}<f_n(M^{\scriptscriptstyle (J')})$ must be connected to the root $\varnothing _{J'}$ by edges that all have PWIT edge weight less than $M^{\scriptscriptstyle (J')}$. The number of such vertices is finite and independent of *n*, and is in particular $O_{\mathbb {P}}(1)$. Since the measure $\mu _n\big \vert _{[0,f_n(1)]}$ has total mass 1 by construction, the total contribution to $Z_{n,{\overline{t}}}$ of this group of vertex pairs is at most $\tfrac{1}{n}\left| {\mathcal {B}}_{{\overline{t}}}^{\scriptscriptstyle (J)}\right| O_{\mathbb {P}}(1)$, which is $o_{\mathbb {P}}(1)$ by Lemma 3.32 (a).

The second group are pairs $(v_1,v_2)$ with $T_{v_{J'}}^{\mathcal {B}}\ge T_\mathrm{fr}^{\scriptscriptstyle (J)}\ge T_{v_J}^{\mathcal {B}}$. For these pairs, by (3.49),

9.54 $$\begin{aligned} \Delta R_{v_1,v_2} = R_J(T_{v_{J'}}^{\mathcal {B}})-R_J(T_{v_J}^{\mathcal {B}})\ge R_J(T_\mathrm{fr}^{\scriptscriptstyle (J)})-R_J(T_{v_J}^{\mathcal {B}})=T_\mathrm{fr}^{\scriptscriptstyle (J)}-T_{v_J}^{\mathcal {B}}. \end{aligned}$$

We can therefore bound the contribution to $Z_{n,{\overline{t}}}$ in terms of the contribution to $\mu _{n,\mathrm{fr}}^{\scriptscriptstyle (J)}$:

9.55 $$\begin{aligned}&\frac{1}{n}\sum _{v_1,v_2:T_{v_{J'}}^{\mathcal {B}}\ge T_\mathrm{fr}^{\scriptscriptstyle (J)}\ge T_{v_J}^{\mathcal {B}}} \mu _n\big \vert _{[0,f_n(1)]}\left( \Delta R_{v_1,v_2}, R_1({\overline{t}})-R_1(T_{v_1}^{\mathcal {B}})+R_2({\overline{t}})-R_2(T_{v_2}^{\mathcal {B}}) \right) \nonumber \\&\quad \le \tfrac{1}{n}\left| {\mathcal {B}}^{\scriptscriptstyle (\smash {J'})}_{{\overline{t}}}\right| \sum _{v_J\in {\mathcal {B}}^{\scriptscriptstyle (J)}_\mathrm{fr}} \mu _n\left( f_n(1)\wedge \left( T_\mathrm{fr}^{\scriptscriptstyle (J)}-T_{v_J}^{\mathcal {B}}\right) , f_n(1) \right) \nonumber \\ {}&\quad \le \tfrac{1}{n} O_{\mathbb {P}}(\sqrt{n s_n}) \sum _{v_J\in {\mathcal {B}}^{\scriptscriptstyle (J)}_\mathrm{fr}} {\mathrm e}^{\lambda _n(1)f_n(1)} \int _0^\infty {\mathrm e}^{-\lambda _n(1)y} \mu _n\left( T_\mathrm{fr}^{\scriptscriptstyle (J)}-T_{v_J}^{\mathcal {B}}+dy \right) \le O_{\mathbb {P}}(\sqrt{s_n^3/n}) \end{aligned}$$

by Lemma 3.32 (a), Lemma 9.1 and (6.15). The last group of vertex pairs are those with $T_{v_J}^{\mathcal {B}}\ge T_\mathrm{fr}^{\scriptscriptstyle (J')} \ge T_{v_{J'}}^{\mathcal {B}}$. These pairs satisfy $\Delta R_{v_1,v_2} \ge T_\mathrm{fr}^{\scriptscriptstyle (J')}-T_{v_{J'}}^{\mathcal {B}}$ instead of (9.54), and their contribution can therefore be handled as in (9.55) with *J* and $J'$ interchanged. $\square$

### Properties of the Freezing Time and Frozen Cluster: Proof of Theorem 3.18

#### Proof of Theorem 3.18

The proof uses the results of [Part I, Theorem 2.15], which applies because the assumptions in [Part I, Conditions 2.2–2.3] are weaker than those of Conditions 2.1–2.3. We adopt the terminology of [Part I, Theorem 2.15], adding superscripts in the obvious way to indicate the two copies of the PWIT.

Let $\varepsilon _1$ denote the constant from Lemma 4.5 with $K=1$. Then Lemma 4.5 asserts that any vertex $v\in \mathsf{BP}^{\scriptscriptstyle (j)}_t$ of age at most 1, i.e. $t-T_v\le f_n(1)$, contributes at least $\varepsilon _1/s_n$ to the sum in the formula (3.31) defining $T_\mathrm{fr}^{\scriptscriptstyle (j)}$, for *n* sufficiently large. In particular, if there are at least $s_n^2/\varepsilon _1$ such vertices at time *t*, then the sum in (3.31) will be at least $s_n$. We conclude that then *v* is lucky, as in Definition 9.3. Thus,

9.56 $$\begin{aligned} T^{\scriptscriptstyle (j)}_\mathrm{fr}\le T^{\scriptscriptstyle (j)}_{\text {lucky}} \end{aligned}$$

for *n* sufficiently large.

On the other hand, by Lemma 4.4 [with ${\bar{\varepsilon }}=1$ and $\varepsilon$ chosen sufficiently small that $\varepsilon /f_n(1)\le \lambda _n(1)$; this is possible, for *n* sufficiently large, by Lemma 3.12], a single vertex can contribute at most

9.57 $$\begin{aligned}&\int _t^\infty {\mathrm e}^{-\lambda _n(1) (y-t)}d\mu _n(y) \le \mu _n(0,Kf_n(1)) + \int {\mathrm e}^{-\varepsilon y'/f_n(1)} {\mathbb {1}}_{\left\{ y'\ge Kf_n(1)\right\} } \mu _n(t+dy')\nonumber \\&\le 1+O(1/s_n) + 1/s_n \end{aligned}$$

to the sum in (3.31). In particular, this contribution is *O*(1). It follows that ${\mathcal {B}}_\mathrm{fr}$ must contain at least $\delta s_n$ vertices, for some $\delta >0$ chosen sufficiently small, and thus that

9.58 $$\begin{aligned} T^{\scriptscriptstyle (j)}_\mathrm{fr}\ge T^{\scriptscriptstyle (j)}_{\text {size}\,\delta s_n}. \end{aligned}$$

The proof now follows from (9.56), (9.58) and [Part I, Theorem 2.15] (with $\sigma _n=s_n/\sqrt{\varepsilon _1}$ and $\sigma _n = \sqrt{\delta s_n}$, respectively). $\square$

**A short guide to notation:**

- $\mathsf{SWT}^{\scriptscriptstyle (j)}_t$ is SWT from vertex $j\in \left\{ 1,2\right\}$.
- ${\mathcal {S}}^{\scriptscriptstyle (j)}_t$ is the SWT from vertex $j\in \left\{ 1,2\right\}$ such that ${\mathcal {S}}^{\scriptscriptstyle (1)}$ and ${\mathcal {S}}^{\scriptscriptstyle (2)}$ cannot merge and with an appropriate freezing procedure.
- $${\mathcal {S}}_t={\mathcal {S}}_t^{\scriptscriptstyle (1)}\cup {\mathcal {S}}_t^{\scriptscriptstyle (2)}$$.
- $\mathsf{BP}^{\scriptscriptstyle (j)}$ is branching process copy number *j* where $j\in \left\{ 1,2\right\}$, without freezing.
- ${\mathcal {B}}^{\scriptscriptstyle (j)}$ is branching process copy number *j* where $j\in \left\{ 1,2\right\}$, with freezing.
- ${\mathcal {B}}_t$ is the union of 2 CTBPs with the appropriate freezing of one of them.
- ${\widetilde{{\mathcal {B}}}}_t$ is the union of 2 CTBPs with the appropriate freezing of one of them, and the resulting thinning. Thus, ${\widetilde{{\mathcal {B}}}}_t$ has the same law as the frozen ${\mathcal {S}}_t$.
- $f_n$ is the function with $Y_e^{\scriptscriptstyle (K_n)}\overset{d}{=} f_n(nE)$, where *E* is exponential with mean 1.
- $\mu _n$ is the image of the Lebesgue measure on $(0,\infty )$ under $f_n$.
- $\lambda _n(a)$ is the exponential growth rate of the CTBP, cf. (3.16).
- $z_t^{\chi }(a)$ and $z_{\vec {t}}^{\chi }(\vec {a})$ are the generation-weighted vertex characteristics from one and two vertices, respectively, cf. (3.14) and (3.22).
- $\bar{m}_t^{\chi }(a)$ and $\bar{m}_{\vec {t}}^{\chi }(\vec {a})$ are the expected, rescaled vertex characteristics, cf. (3.19) and (3.23), respectively.

## Footnotes

1 [Part I, Theorem 1.5 (a)] in fact deals with powers of uniform random variables, but for this discussion that makes no difference.

2 Alternatively, we could retain the definition of ${\mathcal {B}}$ and set the edge weights $Y^{\scriptscriptstyle (K_n)}_{\left\{ i_1,i_2\right\} }$ for $i_1\in {\mathcal {S}}^{\scriptscriptstyle (1)},i_2\in {\mathcal {S}}^{\scriptscriptstyle (2)}$ using an additional source of randomness. We prefer to avoid this option because it would entail proving a more complicated version of Theorem 5.3.
